# LogiKEy workbench: Deontic logics, logic combinations and expressive ethical and legal reasoning (Isabelle/HOL dataset)

**DOI:** 10.1016/j.dib.2020.106409

**Published:** 2020-10-15

**Authors:** Christoph Benzmüller, Ali Farjami, David Fuenmayor, Paul Meder, Xavier Parent, Alexander Steen, Leendert van der Torre, Valeria Zahoransky

**Affiliations:** a University of Luxembourg, Esch sur Alzette, Luxembourg; b Freie Universität Berlin, Berlin, Germany; c Zhejiang University, Hangzhou, China; d University of Oxford, Oxford, UK

**Keywords:** Trustworthy and responsible AI, Knowledge representation and reasoning, Automated theorem proving, Model finding, Normative reasoning, Normative systems, Semantical embedding, Higher-order logic

## Abstract

The LogiKEy workbench and dataset for ethical and legal reasoning is presented. This workbench simultaneously supports development, experimentation, assessment and deployment of formal logics and ethical and legal theories at different conceptual layers. More concretely, it comprises, in form of a dataset (Isabelle/HOL theory files), formal encodings of multiple deontic logics, logic combinations, deontic paradoxes and normative theories in the higher-order proof assistant system Isabelle/HOL. The data were acquired through application of the LogiKEy methodology, which supports experimentation with different normative theories, in different application scenarios, and which is not tied to specific logics or logic combinations. Our workbench consolidates related research contributions of the authors and it may serve as a starting point for further studies and experiments in flexible and expressive ethical and legal reasoning. It may also support hands-on teaching of non-trivial logic formalisms in lecture courses and tutorials.

The LogiKEy methodology and framework is discussed in more detail in the companion research article titled “Designing Normative Theories for Ethical and Legal Reasoning: LogiKEy Framework, Methodology, and Tool Support” [5].

## Specifications Table

**Table:** 

Subject	Computer Science
Specific subject area	Artificial intelligence; Knowledge representation and reasoning; Normative reasoning
Type of data	formal theories (.thy files) encoded in Isabelle/HOL syntax, readable (.png or .pdf) views of this data
How data were acquired	The data were acquired through manual encoding of various deontic logics, logic combinations, examples of contrary-to-duty paradoxes, excerpts of legal texts and exemplary ethical theories utilizing the LogiKEy methodology [5], which is based on shallow semantical embeddings (SSEs) of logics and theories in classical higher-order logic. The concrete encodings were conducted in the higher-order proof assistant system Isabelle/HOL (https://isabelle.in.tum.de); however, they are conceptually transferable to other expressive reasoning systems.
Data format	Raw, processed, analyzed and cleaned data. The dataset is provided in the syntax format of the Isabelle/HOL proof assistant, which has been used to process, analyze and verify it; the data files were also annotated by hand. Isabelle/HOL is freely available: https://isabelle.in.tum.de
Parameters for data collection	One objective was to empirically assess the expressiveness and proof automation capabilities of Isabelle/HOL and its integrated tools in normative reasoning when utilizing the LogiKEy methodology and the SSE approach. Another objective was to provide a reusable foundation for further experiments in expressive ethical and legal reasoning.
Description of data collection	The data were written by hand. As part of the data collection process it has been demonstrated that non-trivial normative reasoning is supported in the provided framework. This in particular included studies of paradoxes in normative reasoning [6] and whether and how they can eventually be avoided. An integral aspect of the data collection process has been also to provide evidence for the practical normative reasoning performance of the various reasoning tools integrated with Isabelle/HOL when utilizing the LogiKEy approach. Useful comments were added to the data files. The practical performance of the logic encodings can be independently assessed by users in combination with the Isabelle/HOL system. It has also been demonstrated how deontic logics can be flexibly combined with other logic formalisms within the LogiKEy approach.
Data source location	The data is hosted on github.com.
Data accessibility	The data is accessible via logikey.org, which redirects to the repository https://github.com/cbenzmueller/LogiKEy on https://github.com, where the data is hosted and maintained. The two subdirectories 2020-DataInBrief-Article and 2020-DataInBrief-Data are associated with this article; the latter contains the dataset.
Related research article	C. Benzmüller, X. Parent, and L. van der Torre. Designing normative theories for ethical and legal reasoning: LogiKEy framework, methodology, and tool support. Artificial Intelligence, 287(103348) 2020. Doi: 10.1016/j.artint.2020.103348.

## Value of the Data

• The provided data can be reused, independently of the related research article(s), as a starting point for further studies and experiments in expressive ethical and legal reasoning. Moreover, it can be reused, extended and adapted to support also other application directions, including, e.g., the study of deontic modality and quantifiers in linguistics.
• The data collection is beneficial for research and application in a range of areas, including but not limited to: machine ethics (ethico-legal governor systems), explainable and trustworthy AI, regulatory technologies, argumentation, natural language semantics. To that end the data includes reusable SSEs of a portfolio of deontic logics, logic combinations, paradoxes in normative reasoning and ethical theories in classical higher-order logic (HOL), interpretable in the Isabelle/HOL proof assistant system. The dataset may also be used to support the teaching of expressive, classical and non-classical logic formalisms and their combinations in lecture courses and tutorials.
• To reuse the data interested researchers, students and practitioners only need to download the provided data files, include them in their formalization projects and suitably extend or adapt them. For example, the contributed data includes a sample encoding of selected statements from the GDPR (General Data Protection Regulation) and an encoding of Gewirth's ethical argument and principle, known as the Principle of Generic Consistency (PGC) [12], in a suitable extension of higher-order deontic logic. These are two examples in the area of knowledge representation and reasoning with an emphasis on regulatory and ethical aspects. They can be reused as a starting point for the encoding and automated solution of similar ethico-legal theories.
• The dataset advances the state of the art in deontic logic [11] as follows. Sixty years after Von Wright's invention of deontic logic, the question has always been how deontic logic and normative theories can be used in computer science applications. The LogiKEy workbench and associated methodology addresses this challenge. The dataset is useful also for stimulation of cross-fertilization effects between different research communities including the deontic logics and normative reasoning communities, the area of higher-order logics, and the area of interactive and automated theorem proving with its various sub-communities targeting very different logic formalisms.
• The presented encodings put a particular emphasis on the modeling of (regulative) norms. We agree with, e.g., Jones and Sergot [14] that deontic logic is needed when it is necessary to make explicit, and then reason about, the distinction between what ought to be the case and what is the case. Furthermore, the adequate handling of the deontic paradoxes (like in particular Chisholm's paradox of contrary-to-duty (CTD) obligation, which deals with norm violation) posed a core challenge for knowledge representation frameworks. This problem motivated the design of deontic logics (and logic combinations) more sophisticated and finer-grained than the traditional ones, like modal logic. Such frameworks are automatized in our work. It is demonstrated that a computer or a machine can reason about norm violation during run-time.

## 1 Data Description

The data are provided in form of Isabelle/HOL source files, which are hosted at logikey.org. The individual data files belong to different categories.

**Fig. 1:**
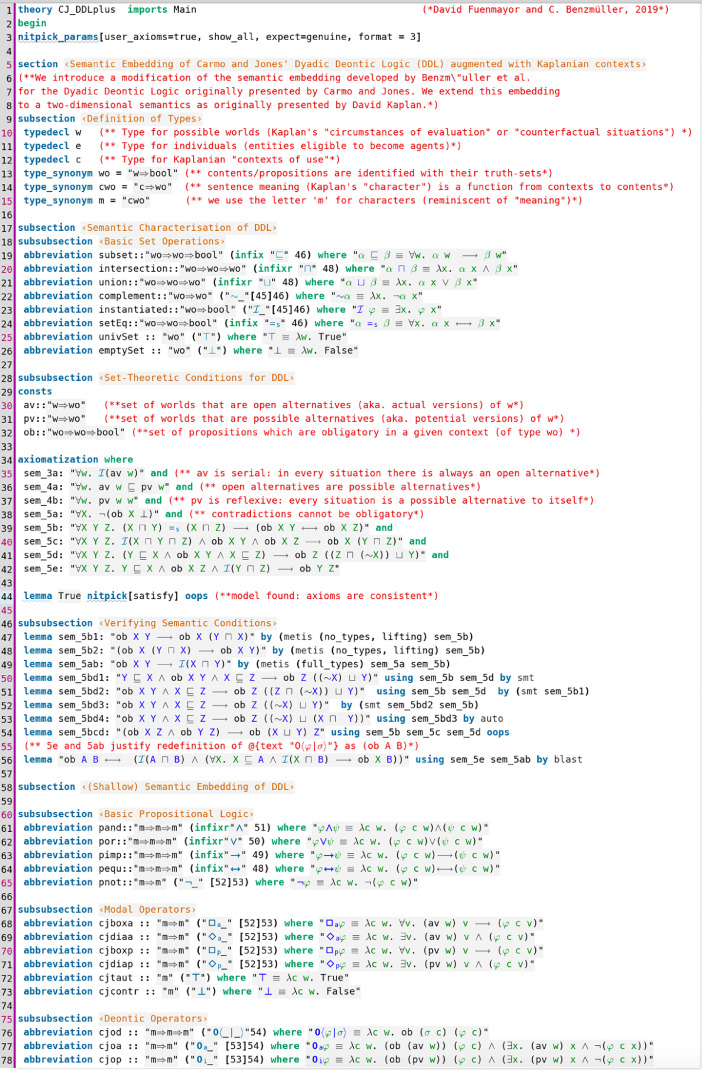
Data file CJ_DDLplus.thy; in lines 29–84 the SSE of the DDL by Carmo and Jones [6] in HOL is presented.

**Fig. 2:**
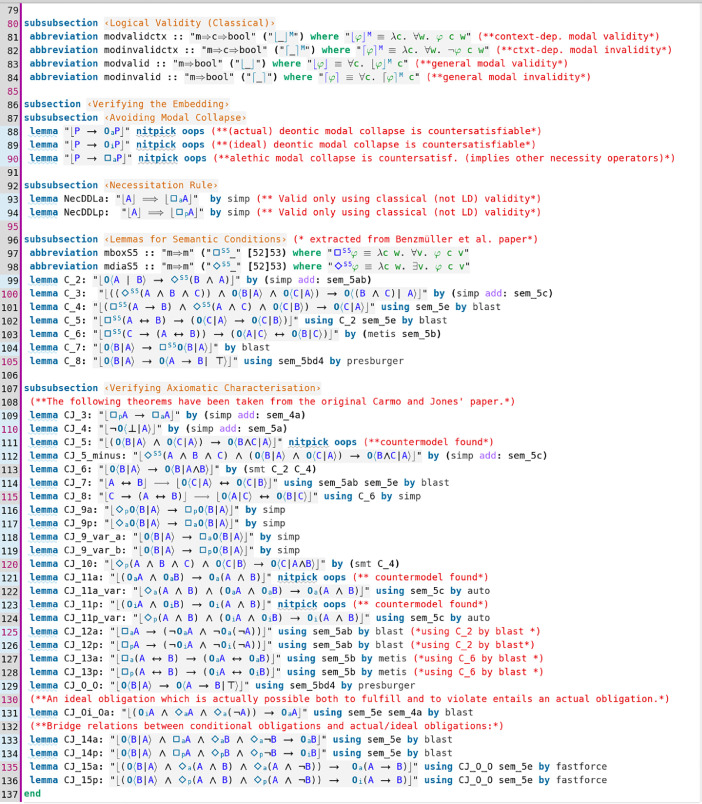
File CJ_DDLplus.thy (cont'd); in lines 87–136 lemmata from Carmo and Jones's paper [6] are verified by automated reasoning tools.

Contributed data files in category I are listed in Table 1. They provide encodings of SSEs, and associated tests, of various deontic logics in meta-logic HOL. A category I example file is displayed in Figs. 1 and 2; this data file contains (an extension of) the SSE of a dyadic deontic logic (DDL) by Carmo and Jones [6] in HOL and studies, resp. verifies, its properties.

**Table 1:** Category I data files—deontic logics, extensions of deontic logics and logic combinations.

File (dependency) reading	Description
SDL.thy (Main.thy)[7]	Provides a SSE of standard deontic logic (SDL) in HOL. An unary deontic operator is defined. The D axiom is postulated and correspondence to seriality of the accessibility is proved. The added first-order and higher-order quantifiers are constant domain (possibilist notion of quantification). This is verified by proving the Barcan formula and its converse.
CJ_DDL.thy (Main.thy)[3]	Provides a SSE of a dyadic deontic logic (DDL) by Carmo and Jones [6] in HOL. Different modal operators are introduced: dyadic deontic obligation, monadic deontic operator for actual obligation, monadic deontic operator for primary obligation, and further alethic modalities. Moreover, constant domain first-order and higher-order quantifiers are added.
CJ_DDL_Tests.thy (CJ_DDL.thy)[3]	Contains soundness and proof automation tests for the embedding of DDL in HOL given in CJ_DDL.thy. For example, the monadic modal operators *O, O_p_* and *O_a_* are identified as S5, KT and KD modalities, respectively. Relevant lemmata from the original work of Carmo and Jones are automated.
E.thy (Main.thy)[4], [5, Fig. 6]	Provides a SSE of a quantified extension of Aqvist's System E in HOL. The file also runs a number of reasoning tasks (validity checking, refutation, correspondence theory).
Lewis_DDL.thy (Main.thy)[16]	Provides a SSE of Lewis's DDL. The file runs a number of reasoning tasks (validity checking, refutation, correspondence theory). The relationship with Åqvist's dyadic deontic operator is also studied.
IOL_out2.thy (Main.thy)[2]	Provides a SSE of a quantified extension of Input/Output (I/O) logic (out_2_) [17,18]. The file also contains an analysis of a benchmark example discussed in the literature on moral luck.
IO_out2_STIT.thy (Main.thy)[2]	Provides a SSE of a quantified extension of I/O logic (out_2_) [17,18] and elements of STIT logics [13] in HOL. The file also contains proof automation tests and soundness checks.
CJ_DDLplus.thy (Main.thy)[9]	A modification of the SSE developed in file CJ_DDL.thy is presented; see Figs. 1 and 2. This theory provides the starting point for an extension of a higher-order variant of DDL into a two-dimensional semantics as originally presented by Kaplan for his logic of demonstratives [15]. The logic extension is completed in file Extended_CJ_DDL.thy. The displayed lines in Fig. 2 show automations of various lemmata from the original paper of Carmo and Jones [6], where they were proved manually with pen and paper.
Extended_CJ_DDL.thy (CJ_DDLplus.thy)[9]	Contains a further extension and combination of the higher-order DDL encoded in file CJ_DDLplus.thy with relevant parts (for the work presented in the related research article [5]) of Kaplan's logic of demonstratives.

**Fig. 3:**
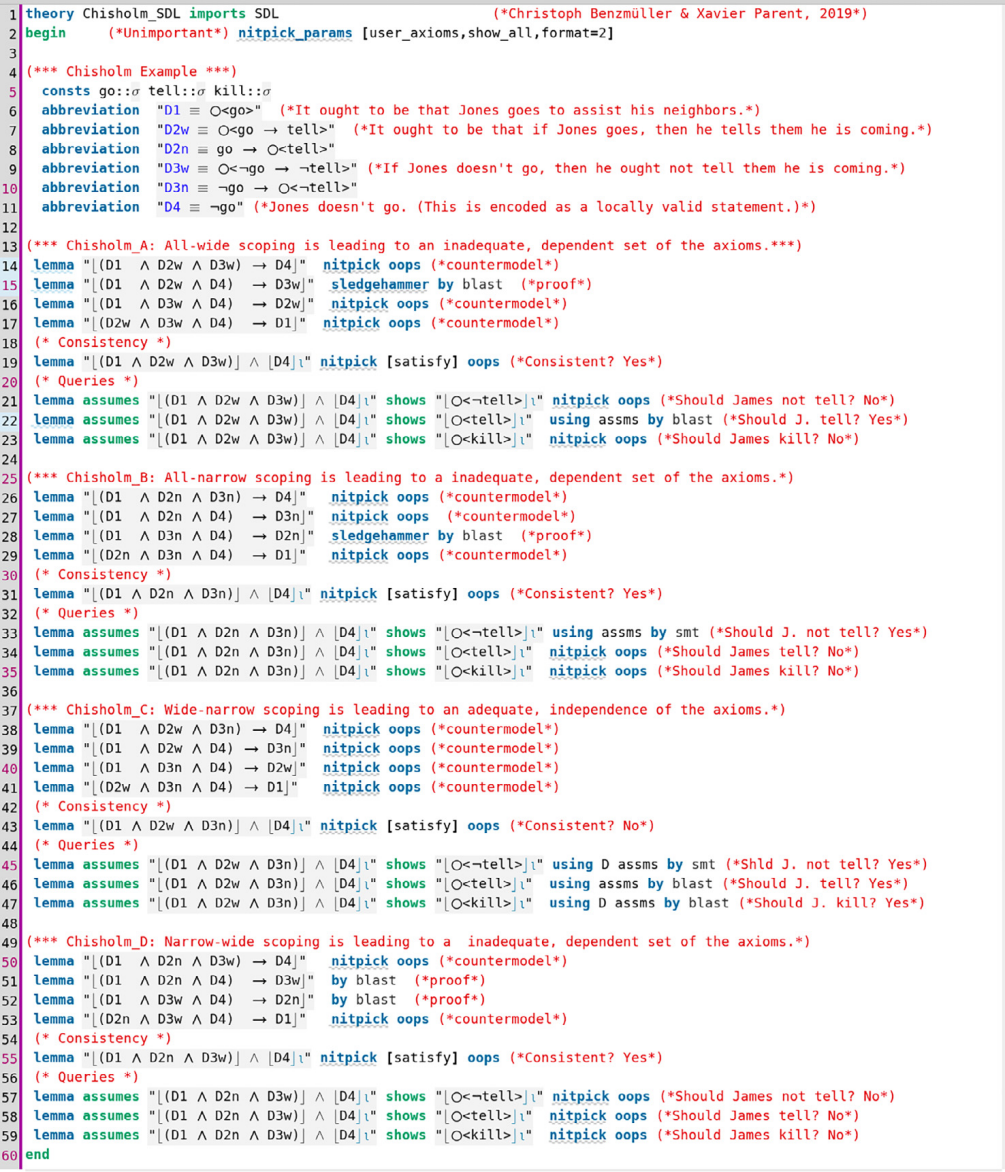
Data file Chisholm_SDL.thy studies Chisholm's paradox in combination with wide-narrow scoping issues.

Contributed data files in category II are listed in Table 2. They study paradoxes and smaller examples of normative reasoning. An example is displayed in Fig. 3, which presents an analysis of Chisholm's paradox of CTD obligation [8] in Standard Deontic Logic (SDL). The known fact that SDL cannot handle CTD scenarios is confirmed by the computer.

**Table 2:** Category II data files: paradoxes and examples of normative reasoning.

File (dependency) reading	Description
Chisholm_SDL.thy (SDL.thy)[18]	The well-known analysis of Chisholm's CTD paradox in SDL is automated. The formalization uses both the wide-scope interpretation of conditional “ought” and the narrow-scope one; see Fig. 3.
Chisholm_CJ_DDL_Monadic.thy (CJ_DDL.thy)	Contains a study analogous to Chisholm_SDL.thy for monadic obligation in DDL.
Chisholm_CJ_DDL_Dyadic.thy (CJ_DDL.thy)	Contains a study analogous to Chisholm_SDL.thy for dyadic obligation in DDL.
Chisholm_E.thy (E.thy)	Contains a study analogous to Chisholm_SDL.thy for deontic logic E.
IO_Experiments (IO_out2_STIT.thy)	Contains a study of different paradoxes from the literature in I/O logic (out_2_); the file imports IO_out2_STIT.thy.

Contributed data files in category III are listed in Table 3. They provide an encoding of (excerpt of) legal and ethical theories and arguments formalized using the deontic logics as provided in category I files and further examined in the category II files.

**Table 3:** Category III data files: (excerpts of) legal and ethical theories and arguments.

File (dependency) reading	Description
GDPR_SDL.thy (SDL.thy) [5, Fig. 7]	Contains a modeling of selected statements from GDPR in first-order SDL. It is confirmed by automated means that first-order SDL cannot handle CTD scenarios.
GDPR_CJ_DDL.thy (CJ_DDL.thy)	Contains a modeling of selected statements from the GDPR in first-order DDL. It is confirmed by automated means that the logic can handle CTD scenarios. The problems identified in GDPR_SDL.thy, i.e., inconsistency and explosion, are avoided. The reasoners return “intuitive” answers to queries.
GDPR_E.thy (E.thy) [5, Fig. 8]	Contains a modeling of selected statements from the GDPR in first-order DDL. It is confirmed by automated means that the logic can handle CTD scenarios. The problems identified in GDPR_SDL.thy, i.e., inconsistency and explosion, are avoided. The reasoners return “intuitive” answers to queries.
GewirthArgument.thy (Extended_CJ_DDL.thy) [9, 5, Fig. 10]	Contains a formalization and partial automation of Gewirth's supporting argument for his *Principle of Generic Consistency*. This principle constitutes, loosely speaking, an emendation of the *Golden Rule*, i.e., the principle of treating others as one's self would wish to be treated. Gewirth's argument and theory is assessed, emended (minor corrections) and verified.

In addition to data listed in Tables 1–3 the dataset provided at http://logikey.org also includes the following:

• Subdirectory 2020-DataInBrief-Data/Course-Material-1 contains Isabelle/HOL data files stemming from a lecture course on deontic logic at the University of Luxembourg based on [18].
• Subdirectory 2020-DataInBrief-Data/Climate-Engineering contains Isabelle/HOL data files related to the formalization and assessment of selected arguments in climate engineering [10].
• Subdirectory 2020-DataInBrief-Data/US-Constitution-Loophole contains Isabelle/HOL data files related to a formalization and assessment of Kurt Gödel's claim that the US Constitution contains a loophole for establishing a dictatorship.
• Subdirectory 2020-DataInBrief-Data/WiseMenPuzzle contains Isabelle/HOL data files related to a formalization and study of the well known Wise Men Puzzle; this dataset, which has been published before [1], is included here to make it better available for http://logikey.org users.

Further related datasets, including selected formalizations in computational metaphysics, will be added to logikey.org as we think fit.

## 2 Experimental Design, Materials and Methods

**Fig. 4:**
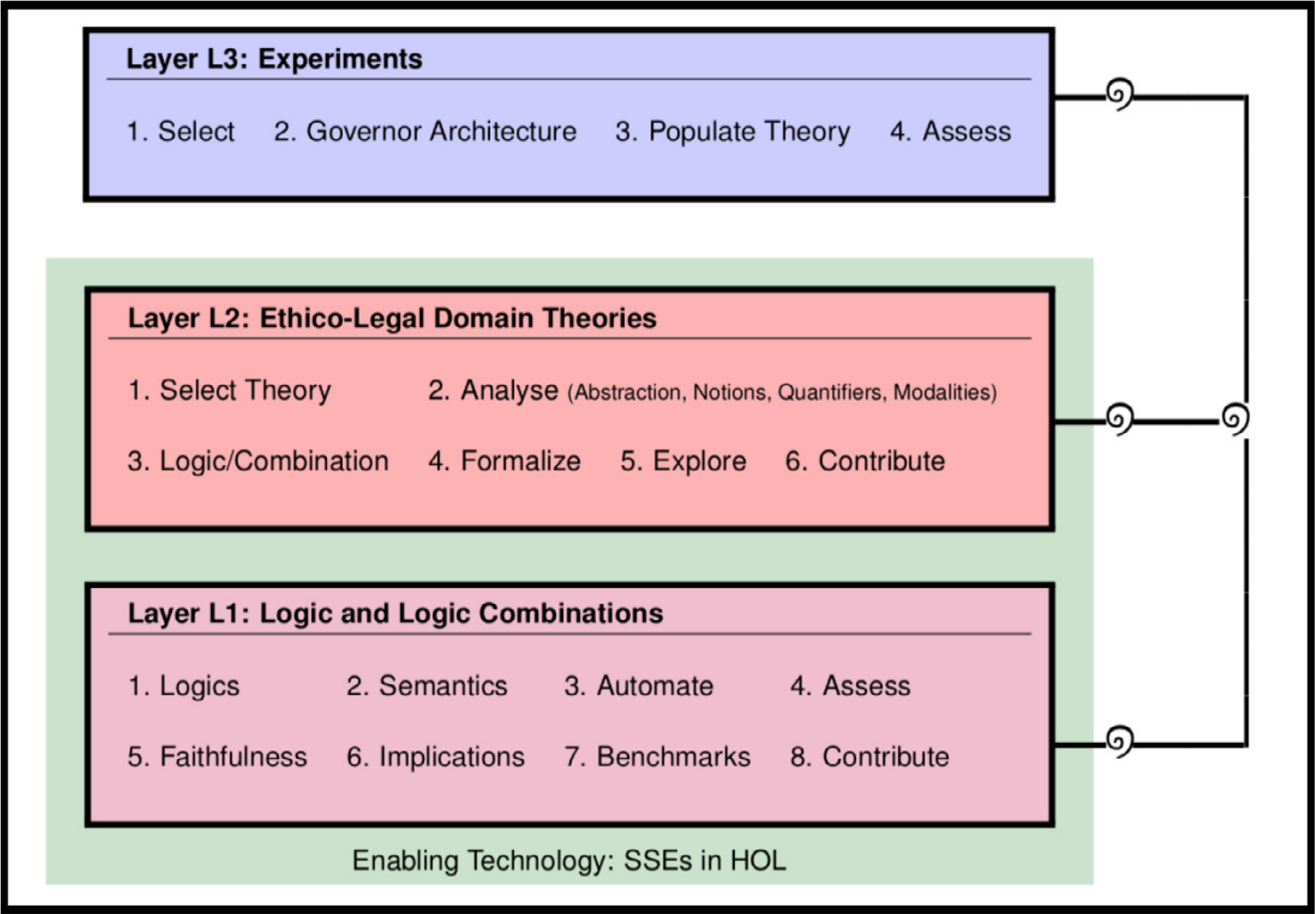
The LogiKEy logic and knowledge development methodology; adapted from [5].

The data were acquired through manual encodings of logics, theories and arguments in the Isabelle/HOL proof assistant system. The modeling process followed the LogiKEy methodology depicted in Fig. 4. This methodology supports formalization projects in the area of ethical and legal reasoning at different layers of abstraction. The spirals in Fig. 4 indicate that the formalization work may proceed in cycles – at each layer and overall. The LogiKEy methodology is briefly explained below at hand of selected examples from our contributed dataset; we address all three different layers and discuss examples.^1^

*Layer L1 example development (files CJ_DDL.thy and CJ_DDL_Tests.thy):* File CJ_DDL.thy contains the encoding (of a quantified extension) of the DDL of Carmo and Jones in HOL. This encoding of DDL in HOL is exemplary for *Layer L1* developments in LogiKEy. First, the desired object *logic* was selected (Step 1); Carmo and Jones's DDL in the given case. A *semantics* (Step 2) for this object logic was sought and found in the original papers by Carmo and Jones [6]; such a mathematical description of a semantics, a neighborhood semantics in the given case, constitutes the ideal starting point for the definition of a SSE of the object logic in HOL, which in turn enables its *automation* (Step 3) with off-the-shelf reasoning tools for HOL. The automation of DDL was subsequently assessed (Step 4) with automated theorem provers and model finders integrated with Isabelle/HOL. Then, by pen and paper means on a theoretical level, the *faithfulness* (Step 4) of the embedding of DDL in HOL was studied and proved; this proof has been published [3]. Furthermore, *implications* of the embedding of DDL in HOL were studied (Step 5); see for example the additional theorems in file CJ_DDL_Tests.thy and the analysis of CTD scenarios conducted in files Chisholm_DDL_Monadic.thy and Chisholm_DDL_Dyadic.thy. Since the DDL of Carmo and Jones has not been automated before with other systems or approaches, there are no *benchmarks* (Step 7) available that we could use to properly assess and compare the competitiveness of our solution. The publication of this dataset can be seen as a first step towards the built-up and *contribution* (Step 8) of such a benchmark suite to the community.

*Layer L2 example development (file GDPR_CJ_DDL.thy):* In file GDPR_CJ_DDL.thy we *selected* (Step 1) statements from the General Data Protection Regulation (GDPR) for formalization. The *analysis* (Step 2) of these statements revealed that obligation aspects in the context of data processing needed to be addressed. Natural language phrases in the studied parts of the GDPR indeed contains occurrences of the deontic modalities. This motivated the choice of a suitable deontic *logic* (Step 3), such as DDL, for the formal encoding of these challenging aspects. In the given case it also became apparent that a propositional encoding would hardly suffice in practical applications, so the selected deontic logic DDL needed to be extended by a notion of quantification, which led to the addition of quantifiers to the file CJ_DDL.thy. Subsequently the two GDPR articles were *formalized* (Step 4) using logical connectives as provided in the imported file CJ_DDL.thy, and then some *exploration* (Step 5) and assessment studies were conducted. This included the analysis of the CTD scenario as reported in related research articles [5,3]. With our dataset we *contribute* (Step 6) this work to the wider research community and enable its reuse.

*Layer L3 example development:* Layer L3 example developments have just started. The idea is to populate regulatory governor architectures [5] with ethical and legal theories from Layer L2, so that reasoning with the theories can be utilized to explain and control the behavior of (autonomous) AI systems. To realize such applications it is required to *select* (Step 1) some ethical and/or legal theory from Layer L2, to devise and implement (or reuse) a respective *governor architecture* (Step 2), to *populate* (Step 3) this governor system with the selected ethical and/or legal theory, and to *assess* (Step 4) the well-functioning of this system in empirical studies.

## Ethics Statement

Our work did not involve the use of human subjects, and it did not involve animal experiments.

## Declaration of Competing Interest

Benzmüller was funded by the VolkswagenStiftung under grant CRAP (Consistent Rational Argumentation in Politics). Parent and van der Torre were supported by the European Union's Horizon 2020 research and innovation programme under the Marie Skłodowska-Curie grant agreement MIREL (MIning and REasoning with Legal texts) No 690974.

## Appendix Supplementary materials

Image, application 1

## Footnotes

1 For a general description of the LogiKEy framework, methodology and tool support see the related research article [5].
