# Supplementary material for: LogiKEy workbench: Deontic logics, logic combinations and expressive ethical and legal reasoning (Isabelle/HOL dataset)
Source: Data Brief. 2020 Oct 15;33:106409. doi: 10.1016/j.dib.2020.106409 (PMC7586073; doi:10.1016/j.dib.2020.106409)
Supplement: Supplementary file 1 [file mmc1.zip › 2020-DataInBrief-Data/GDPR_E.html]

xml version="1.0" encoding="utf-8"?


Theory GDPR\_E (Isabelle2019: June 2019)


# Theory GDPR\_E

theory GDPR\_E  
imports E

```
theory GDPR_E imports E       (*GDPR CTD Example. C. Benzmüller & X. Parent, 2019*)
begin
(*Unimportant.*) sledgehammer_params [max_facts=20,timeout=20] 
(*Unimportant.*) nitpick_params [user_axioms,expect=genuine,show_all,dont_box]

datatype data = d1 | d2   (*We introduce concrete data objects d1 and d2.*)
datatype indiv = Mary | Peter (*We introduce individuals Mary and Peter.*)
consts process_lawfully::"data⇒σ" erase::"data⇒σ" is_protected_by_GDPR::"data⇒σ" 
             belongs_to::"data⇒indiv⇒σ" is_european::"indiv=>σ " kill::"indiv⇒σ"

axiomatization where
(*Data belonging to Europeans is protected by the GDPR.*)
 A0: "⌊❙∀x. ❙∀d. (is_european x ❙∧ belongs_to d x) ❙→ is_protected_by_GDPR d⌋" and
(*Data d1 is belonging to the European Peter.*)
 F1: "⌊belongs_to d1 Peter ❙∧ is_european Peter⌋" and

(*It is an obligation to process data lawfully.*)
 A1: "⌊❙∀d. ○<process_lawfully d | is_protected_by_GDPR d>⌋"  and
(*If data was not processed lawfully, then it is an obligation to erase the data.*)
 A2: "⌊❙∀d. ○<erase d | is_protected_by_GDPR d  ❙∧ ❙¬process_lawfully d>⌋" and
(*Implicit: It is an obligation to keep the data if it was processed lawfully.*)
 A3: "⌊❙∀d. ○<❙¬erase d | is_protected_by_GDPR d ❙∧ process_lawfully d>⌋" and
(*Given a situation where data is processed unlawfully.*) 
 Situation: "⌊❙¬process_lawfully d1⌋⇩l" 


(***Some Experiments***) 
 lemma True nitpick [satisfy] oops (*Consistency-check: Nitpick finds a model.*) 
 lemma False sledgehammer oops (*Inconsistency-check: Can Falsum be derived? No.*)
 lemma "⌊❙○<kill Mary>⌋⇩l" nitpick oops (*Should Mary be killed? —
                                                                   Answer is no. Countermodel by Nitpick.*)
end
```
