# Supplementary material for: LogiKEy workbench: Deontic logics, logic combinations and expressive ethical and legal reasoning (Isabelle/HOL dataset)
Source: Data Brief. 2020 Oct 15;33:106409. doi: 10.1016/j.dib.2020.106409 (PMC7586073; doi:10.1016/j.dib.2020.106409)
Supplement: Supplementary file 1 [file mmc1.zip › 2020-DataInBrief-Data/CJ_DDLplus.html]

xml version="1.0" encoding="utf-8"?


Theory CJ\_DDLplus (Isabelle2019: June 2019)


# Theory CJ\_DDLplus

theory CJ\_DDLplus  
imports Main

```
theory CJ_DDLplus  imports Main                                       (*David Fuenmayor and C. Benzmüller, 2019*)
begin
nitpick_params[user_axioms=true, show_all, expect=genuine, format = 3]

section ‹Semantic Embedding of Carmo and Jones' Dyadic Deontic Logic (DDL) augmented with Kaplanian contexts›
(**We introduce a modification of the semantic embedding developed by Benzm\"uller et al.
for the Dyadic Deontic Logic originally presented by Carmo and Jones. We extend this embedding
to a two-dimensional semantics as originally presented by David Kaplan.*)
subsection ‹Definition of Types›
 typedecl w   (** Type for possible worlds (Kaplan's "circumstances of evaluation" or "counterfactual situations") *)
 typedecl e   (** Type for individuals (entities eligible to become agents)*)
 typedecl c   (** Type for Kaplanian "contexts of use"*)
 type_synonym wo = "w⇒bool" (** contents/propositions are identified with their truth-sets*)
 type_synonym cwo = "c⇒wo"  (** sentence meaning (Kaplan's "character") is a function from contexts to contents*)
 type_synonym m = "cwo"      (** we use the letter 'm' for characters (reminiscent of "meaning")*)

subsection ‹Semantic Characterisation of DDL›
subsubsection ‹Basic Set Operations›
 abbreviation subset::"wo⇒wo⇒bool" (infix "⊑" 46) where "α ⊑ β ≡ ∀w. α w  ⟶ β w"
 abbreviation intersection::"wo⇒wo⇒wo" (infixr "⊓" 48) where "α ⊓ β ≡ λx. α x ∧ β x"
 abbreviation union::"wo⇒wo⇒wo" (infixr "⊔" 48) where "α ⊔ β ≡ λx. α x ∨ β x"
 abbreviation complement::"wo⇒wo" ("∼_"[45]46) where "∼α ≡ λx. ¬α x"
 abbreviation instantiated::"wo⇒bool" ("ℐ_"[45]46) where "ℐ φ ≡ ∃x. φ x"
 abbreviation setEq::"wo⇒wo⇒bool" (infix "=⇩s" 46) where "α =⇩s β ≡ ∀x. α x ⟷ β x"
 abbreviation univSet :: "wo" ("⊤") where "⊤ ≡ λw. True"
 abbreviation emptySet :: "wo" ("⊥") where "⊥ ≡ λw. False"

subsubsection ‹Set-Theoretic Conditions for DDL›
consts
 av::"w⇒wo"   (**set of worlds that are open alternatives (aka. actual versions) of w*)
 pv::"w⇒wo"   (**set of worlds that are possible alternatives (aka. potential versions) of w*)
 ob::"wo⇒wo⇒bool" (**set of propositions which are obligatory in a given context (of type wo) *)

axiomatization where
 sem_3a: "∀w. ℐ(av w)" and (** av is serial: in every situation there is always an open alternative*)
 sem_4a: "∀w. av w ⊑ pv w" and (** open alternatives are possible alternatives*)
 sem_4b: "∀w. pv w w" and (** pv is reflexive: every situation is a possible alternative to itself*)
 sem_5a: "∀X. ¬(ob X ⊥)" and (** contradictions cannot be obligatory*)
 sem_5b: "∀X Y Z. (X ⊓ Y) =⇩s (X ⊓ Z) ⟶ (ob X Y ⟷ ob X Z)" and
 sem_5c: "∀X Y Z. ℐ(X ⊓ Y ⊓ Z) ∧ ob X Y ∧ ob X Z ⟶ ob X (Y ⊓ Z)" and
 sem_5d: "∀X Y Z. (Y ⊑ X ∧ ob X Y ∧ X ⊑ Z) ⟶ ob Z ((Z ⊓ (∼X)) ⊔ Y)" and
 sem_5e: "∀X Y Z. Y ⊑ X ∧ ob X Z ∧ ℐ(Y ⊓ Z) ⟶ ob Y Z"

 lemma True nitpick[satisfy] oops (**model found: axioms are consistent*)

subsubsection ‹Verifying Semantic Conditions›
 lemma sem_5b1: "ob X Y ⟶ ob X (Y ⊓ X)" by (metis (no_types, lifting) sem_5b)
 lemma sem_5b2: "(ob X (Y ⊓ X) ⟶ ob X Y)" by (metis (no_types, lifting) sem_5b) 
 lemma sem_5ab: "ob X Y ⟶ ℐ(X ⊓ Y)" by (metis (full_types) sem_5a sem_5b)
 lemma sem_5bd1: "Y ⊑ X ∧ ob X Y ∧ X ⊑ Z ⟶ ob Z ((∼X) ⊔ Y)" using sem_5b sem_5d by smt
 lemma sem_5bd2: "ob X Y ∧ X ⊑ Z ⟶ ob Z ((Z ⊓ (∼X)) ⊔ Y)"  using sem_5b sem_5d  by (smt sem_5b1)  
 lemma sem_5bd3: "ob X Y ∧ X ⊑ Z ⟶ ob Z ((∼X) ⊔ Y)"  by (smt sem_5bd2 sem_5b) 
 lemma sem_5bd4: "ob X Y ∧ X ⊑ Z ⟶ ob Z ((∼X) ⊔ (X ⊓  Y))" using sem_5bd3 by auto
 lemma sem_5bcd: "(ob X Z ∧ ob Y Z) ⟶ ob (X ⊔ Y) Z" using sem_5b sem_5c sem_5d oops
 (** 5e and 5ab justify redefinition of @{text "O⟨φ|σ⟩"} as (ob A B)*)
 lemma "ob A B ⟷  (ℐ(A ⊓ B) ∧ (∀X. X ⊑ A ∧ ℐ(X ⊓ B) ⟶ ob X B))" using sem_5e sem_5ab by blast

subsection ‹(Shallow) Semantic Embedding of DDL›

subsubsection ‹Basic Propositional Logic›
 abbreviation pand::"m⇒m⇒m" (infixr"❙∧" 51) where "φ❙∧ψ ≡ λc w. (φ c w)∧(ψ c w)"
 abbreviation por::"m⇒m⇒m" (infixr"❙∨" 50) where "φ❙∨ψ ≡ λc w. (φ c w)∨(ψ c w)"
 abbreviation pimp::"m⇒m⇒m" (infix"❙→" 49) where "φ❙→ψ ≡ λc w. (φ c w)⟶(ψ c w)"
 abbreviation pequ::"m⇒m⇒m" (infix"❙↔" 48) where "φ❙↔ψ ≡ λc w. (φ c w)⟷(ψ c w)"
 abbreviation pnot::"m⇒m" ("❙¬_" [52]53) where "❙¬φ ≡ λc w. ¬(φ c w)"

subsubsection ‹Modal Operators›
 abbreviation cjboxa :: "m⇒m" ("❙□⇩a_" [52]53) where "❙□⇩aφ ≡ λc w. ∀v. (av w) v ⟶ (φ c v)"
 abbreviation cjdiaa :: "m⇒m" ("❙◇⇩a_" [52]53) where "❙◇⇩aφ ≡ λc w. ∃v. (av w) v ∧ (φ c v)"
 abbreviation cjboxp :: "m⇒m" ("❙□⇩p_" [52]53) where "❙□⇩pφ ≡ λc w. ∀v. (pv w) v ⟶ (φ c v)"
 abbreviation cjdiap :: "m⇒m" ("❙◇⇩p_" [52]53) where "❙◇⇩pφ ≡ λc w. ∃v. (pv w) v ∧ (φ c v)"
 abbreviation cjtaut :: "m" ("❙⊤") where "❙⊤ ≡ λc w. True"
 abbreviation cjcontr :: "m" ("❙⊥") where "❙⊥ ≡ λc w. False"

subsubsection ‹Deontic Operators›
 abbreviation cjod :: "m⇒m⇒m" ("❙O⟨_|_⟩"54) where "❙O⟨φ|σ⟩ ≡ λc w. ob (σ c) (φ c)"
 abbreviation cjoa :: "m⇒m" ("❙O⇩a_" [53]54) where "❙O⇩aφ ≡ λc w. (ob (av w)) (φ c) ∧ (∃x. (av w) x ∧ ¬(φ c x))"
 abbreviation cjop :: "m⇒m" ("❙O⇩i_" [53]54) where "❙O⇩iφ ≡ λc w. (ob (pv w)) (φ c) ∧ (∃x. (pv w) x ∧ ¬(φ c x))"

subsubsection ‹Logical Validity (Classical)›
 abbreviation modvalidctx :: "m⇒c⇒bool" ("⌊_⌋⇧M") where "⌊φ⌋⇧M ≡ λc. ∀w. φ c w" (**context-dep. modal validity*)
 abbreviation modinvalidctx :: "m⇒c⇒bool" ("⌈_⌉⇧M") where "⌈φ⌉⇧M ≡ λc. ∀w. ¬φ c w" (**ctxt-dep. modal invalidity*)
 abbreviation modvalid :: "m⇒bool" ("⌊_⌋") where "⌊φ⌋ ≡ ∀c. ⌊φ⌋⇧M c" (**general modal validity*)
 abbreviation modinvalid :: "m⇒bool" ("⌈_⌉") where "⌈φ⌉ ≡ ∀c. ⌈φ⌉⇧M c" (**general modal invalidity*)

subsection ‹Verifying the Embedding›
subsubsection ‹Avoiding Modal Collapse›
 lemma "⌊P ❙→ ❙O⇩aP⌋" nitpick oops (**(actual) deontic modal collapse is countersatisfiable*)
 lemma "⌊P ❙→ ❙O⇩iP⌋" nitpick oops (**(ideal) deontic modal collapse is countersatisfiable*)
 lemma "⌊P ❙→ ❙□⇩aP⌋" nitpick oops (**alethic modal collapse is countersatisf. (implies other necessity operators)*)

subsubsection ‹Necessitation Rule›
 lemma NecDDLa: "⌊A⌋ ⟹ ⌊❙□⇩aA⌋"  by simp (** Valid only using classical (not LD) validity*)
 lemma NecDDLp:  "⌊A⌋ ⟹ ⌊❙□⇩pA⌋" by simp (** Valid only using classical (not LD) validity*)

subsubsection ‹Lemmas for Semantic Conditions› (* extracted from Benzmüller et al. paper*)
 abbreviation mboxS5 :: "m⇒m" ("❙□⇧S⇧5_" [52]53) where "❙□⇧S⇧5φ ≡ λc w. ∀v. φ c v"
 abbreviation mdiaS5 :: "m⇒m" ("❙◇⇧S⇧5_" [52]53) where "❙◇⇧S⇧5φ ≡ λc w. ∃v. φ c v"
 lemma C_2: "⌊❙O⟨A | B⟩ ❙→ ❙◇⇧S⇧5(B ❙∧ A)⌋" by (simp add: sem_5ab)
 lemma C_3:  "⌊((❙◇⇧S⇧5(A ❙∧ B ❙∧ C)) ❙∧ ❙O⟨B|A⟩ ❙∧ ❙O⟨C|A⟩) ❙→ ❙O⟨(B ❙∧ C)| A⟩⌋" by (simp add: sem_5c)
 lemma C_4: "⌊(❙□⇧S⇧5(A ❙→ B) ❙∧ ❙◇⇧S⇧5(A ❙∧ C) ❙∧ ❙O⟨C|B⟩) ❙→ ❙O⟨C|A⟩⌋" using sem_5e by blast
 lemma C_5: "⌊❙□⇧S⇧5(A ❙↔ B) ❙→ (❙O⟨C|A⟩ ❙→ ❙O⟨C|B⟩)⌋" using C_2 sem_5e by blast
 lemma C_6: "⌊❙□⇧S⇧5(C ❙→ (A ❙↔ B)) ❙→ (❙O⟨A|C⟩ ❙↔ ❙O⟨B|C⟩)⌋" by (metis sem_5b)
 lemma C_7: "⌊❙O⟨B|A⟩ ❙→ ❙□⇧S⇧5❙O⟨B|A⟩⌋" by blast 
 lemma C_8: "⌊❙O⟨B|A⟩ ❙→ ❙O⟨A ❙→ B| ❙⊤⟩⌋" using sem_5bd4 by presburger

subsubsection ‹Verifying Axiomatic Characterisation›
 (**The following theorems have been taken from the original Carmo and Jones' paper.*)
 lemma CJ_3: "⌊❙□⇩pA ❙→ ❙□⇩aA⌋" by (simp add: sem_4a)
 lemma CJ_4: "⌊❙¬❙O⟨❙⊥|A⟩⌋" by (simp add: sem_5a)
 lemma CJ_5: "⌊(❙O⟨B|A⟩ ❙∧ ❙O⟨C|A⟩) ❙→ ❙O⟨B❙∧C|A⟩⌋" nitpick oops (**countermodel found*)
 lemma CJ_5_minus: "⌊❙◇⇧S⇧5(A ❙∧ B ❙∧ C) ❙∧ (❙O⟨B|A⟩ ❙∧ ❙O⟨C|A⟩) ❙→ ❙O⟨B❙∧C|A⟩⌋" by (simp add: sem_5c)
 lemma CJ_6: "⌊❙O⟨B|A⟩ ❙→ ❙O⟨B|A❙∧B⟩⌋" by (smt C_2 C_4)
 lemma CJ_7: "⌊A ❙↔ B⌋ ⟶ ⌊❙O⟨C|A⟩ ❙↔ ❙O⟨C|B⟩⌋" using sem_5ab sem_5e by blast 
 lemma CJ_8: "⌊C ❙→ (A ❙↔ B)⌋ ⟶ ⌊❙O⟨A|C⟩ ❙↔ ❙O⟨B|C⟩⌋" using C_6 by simp 
 lemma CJ_9a: "⌊❙◇⇩p❙O⟨B|A⟩ ❙→ ❙□⇩p❙O⟨B|A⟩⌋" by simp
 lemma CJ_9p: "⌊❙◇⇩a❙O⟨B|A⟩ ❙→ ❙□⇩a❙O⟨B|A⟩⌋" by simp
 lemma CJ_9_var_a: "⌊❙O⟨B|A⟩ ❙→ ❙□⇩a❙O⟨B|A⟩⌋" by simp
 lemma CJ_9_var_b: "⌊❙O⟨B|A⟩ ❙→ ❙□⇩p❙O⟨B|A⟩⌋" by simp
 lemma CJ_10: "⌊❙◇⇩p(A ❙∧ B ❙∧ C) ❙∧ ❙O⟨C|B⟩ ❙→ ❙O⟨C|A❙∧B⟩⌋" by (smt C_4)
 lemma CJ_11a: "⌊(❙O⇩aA ❙∧ ❙O⇩aB) ❙→ ❙O⇩a(A ❙∧ B)⌋" nitpick oops (** countermodel found*)
 lemma CJ_11a_var: "⌊❙◇⇩a(A ❙∧ B) ❙∧ (❙O⇩aA ❙∧ ❙O⇩aB) ❙→ ❙O⇩a(A ❙∧ B)⌋" using sem_5c by auto
 lemma CJ_11p: "⌊(❙O⇩iA ❙∧ ❙O⇩iB) ❙→ ❙O⇩i(A ❙∧ B)⌋" nitpick oops (** countermodel found*)
 lemma CJ_11p_var: "⌊❙◇⇩p(A ❙∧ B) ❙∧ (❙O⇩iA ❙∧ ❙O⇩iB) ❙→ ❙O⇩i(A ❙∧ B)⌋" using sem_5c by auto
 lemma CJ_12a: "⌊❙□⇩aA ❙→ (❙¬❙O⇩aA ❙∧ ❙¬❙O⇩a(❙¬A))⌋" using sem_5ab by blast (*using C_2 by blast *)
 lemma CJ_12p: "⌊❙□⇩pA ❙→ (❙¬❙O⇩iA ❙∧ ❙¬❙O⇩i(❙¬A))⌋" using sem_5ab by blast (*using C_2 by blast*) 
 lemma CJ_13a: "⌊❙□⇩a(A ❙↔ B) ❙→ (❙O⇩aA ❙↔ ❙O⇩aB)⌋" using sem_5b by metis (*using C_6 by blast *)
 lemma CJ_13p: "⌊❙□⇩p(A ❙↔ B) ❙→ (❙O⇩iA ❙↔ ❙O⇩iB)⌋" using sem_5b by metis (*using C_6 by blast *)
 lemma CJ_O_O: "⌊❙O⟨B|A⟩ ❙→ ❙O⟨A ❙→ B|❙⊤⟩⌋" using sem_5bd4 by presburger
 (**An ideal obligation which is actually possible both to fulfill and to violate entails an actual obligation.*)
 lemma CJ_Oi_Oa: "⌊(❙O⇩iA ❙∧ ❙◇⇩aA ❙∧ ❙◇⇩a(❙¬A)) ❙→ ❙O⇩aA⌋" using sem_5e sem_4a by blast
 (**Bridge relations between conditional obligations and actual/ideal obligations:*)
 lemma CJ_14a: "⌊❙O⟨B|A⟩ ❙∧ ❙□⇩aA ❙∧ ❙◇⇩aB ❙∧ ❙◇⇩a❙¬B ❙→ ❙O⇩aB⌋" using sem_5e by blast
 lemma CJ_14p: "⌊❙O⟨B|A⟩ ❙∧ ❙□⇩pA ❙∧ ❙◇⇩pB ❙∧ ❙◇⇩p❙¬B ❙→ ❙O⇩iB⌋" using sem_5e by blast
 lemma CJ_15a: "⌊(❙O⟨B|A⟩ ❙∧ ❙◇⇩a(A ❙∧ B) ❙∧ ❙◇⇩a(A ❙∧ ❙¬B)) ❙→  ❙O⇩a(A ❙→ B)⌋" using CJ_O_O sem_5e by fastforce 
 lemma CJ_15p: "⌊(❙O⟨B|A⟩ ❙∧ ❙◇⇩p(A ❙∧ B) ❙∧ ❙◇⇩p(A ❙∧ ❙¬B)) ❙→  ❙O⇩i(A ❙→ B)⌋" using CJ_O_O sem_5e by fastforce 
end
```
