# Supplementary material for: LogiKEy workbench: Deontic logics, logic combinations and expressive ethical and legal reasoning (Isabelle/HOL dataset)
Source: Data Brief. 2020 Oct 15;33:106409. doi: 10.1016/j.dib.2020.106409 (PMC7586073; doi:10.1016/j.dib.2020.106409)
Supplement: Supplementary file 1 [file mmc1.zip › 2020-DataInBrief-Data/CJ_DDL_Tests.html]

xml version="1.0" encoding="utf-8"?


Theory CJ\_DDL\_Tests (Isabelle2019: June 2019)


# Theory CJ\_DDL\_Tests

theory CJ\_DDL\_Tests  
imports CJ\_DDL

```
theory CJ_DDL_Tests imports CJ_DDL           (* Christoph Benzmüller, Ali Farjami, Xavier Parent, 2020  *)
begin (* Some Tests on the Meta-Theory of DDL*)
lemma True nitpick [satisfy,user_axioms,expect=genuine] oops  (* Consistency confirmed by Nitpick *)  
 
lemma MP: "⟦⌊A⌋; ⌊A ❙→ B⌋⟧ ⟹ ⌊B⌋" by simp
lemma Nec: "⌊A⌋ ⟹ ⌊❙□A⌋" by simp
lemma Neca: "⌊A⌋ ⟹ ⌊❙□⇩aA⌋"  by simp
lemma Necp: "⌊A⌋ ⟹ ⌊❙□⇩p A⌋"  by simp

(* "❙□" is an S5 modality *)
lemma C_1_refl: "⌊❙□A ❙→ A⌋" by simp
lemma C_1_trans: "⌊❙□A ❙→ (❙□(❙□A))⌋"  by simp
lemma C_1_sym: "⌊A ❙→ (❙□(❙◇A))⌋"  by simp

(* "❙□⇩p" is an KT modality *)
lemma C_9_p_refl: "⌊❙□⇩pA ❙→ A⌋"  by (simp add: ax_4b)
lemma "⌊❙□⇩pA ❙→ (❙□⇩p(❙□⇩pA))⌋" nitpick [user_axioms] oops (* countermodel *)
lemma "⌊A ❙→ (❙□⇩p(❙◇⇩pA))⌋"   nitpick [user_axioms] oops (* countermodel *)

(* "❙□⇩a" is an KD modality *)
lemma C_10_a_serial: "⌊❙□⇩aA ❙→ ❙◇⇩aA⌋"  by (simp add: ax_3a) 
lemma "⌊❙□⇩aA ❙→ A⌋" nitpick [user_axioms] oops (* countermodel *)
lemma "⌊❙□⇩aA ❙→ (❙□⇩a(❙□⇩aA))⌋" nitpick [user_axioms] oops (* countermodel *)
lemma "⌊A ❙→ (❙□⇩a(❙◇⇩aA))⌋" nitpick [user_axioms] oops (* countermodel *)

(* Relationship between "❙□,❙□⇩a,❙□⇩p" *)
lemma C_11: "⌊❙□A ❙→ ❙□⇩pA⌋" sledgehammer by simp 
lemma C_12: "⌊❙□⇩pA ❙→ ❙□⇩aA⌋"  using ax_4a by auto

(* Observation II-2-1 *)
lemma ax_5b': "ob X Y ⟷ ob X (λz. X z ∧ Y z)" by (metis (no_types, lifting) ax_5b) 
lemma ax_5b'': "ob X Y ⟷ ob X (λz. Y z ∧ X z)" by (metis (no_types, lifting) ax_5b) 

(* Characterisation of "❙O" *)
lemma C_2: "⌊❙O❙⟨A❙|B❙⟩ ❙→ ❙◇(B ❙∧ A)⌋"  by (metis ax_5a ax_5b)  
lemma C_3: "⌊(❙◇(A ❙∧ B ❙∧ C) ❙∧ ❙O❙⟨B❙|A❙⟩ ❙∧ ❙O❙⟨C❙|A❙⟩ ) ❙→ ❙O❙⟨(B ❙∧ C)❙|A❙⟩⌋" using ax_5c by auto 
lemma C_4: "⌊(❙□(A ❙→ B) ❙∧ (❙◇(A ❙∧ C)) ❙∧ ❙O❙⟨C❙|B❙⟩) ❙→ ❙O❙⟨C❙|A❙⟩⌋"   using ax_5e by blast
lemma C_5: "⌊❙□(A ❙↔ B) ❙→ (❙O❙⟨C❙|A❙⟩ ❙↔ ❙O❙⟨C❙|B❙⟩)⌋"  by presburger 
lemma C_6: "⌊❙□(C ❙→ (A ❙↔ B)) ❙→ (❙O❙⟨A❙|C❙⟩ ❙↔ ❙O❙⟨B❙|C❙⟩)⌋"  by (smt ax_5b) 
lemma C_7: "⌊❙O❙⟨B❙|A❙⟩ ❙→ ❙□(❙O❙⟨B❙|A❙⟩)⌋"  by blast 
lemma C_8: "⌊❙O❙⟨B❙|A❙⟩ ❙→ ❙O❙⟨(A ❙→ B)❙|❙⊤❙⟩⌋"  
 proof -   
  have  "∀X Y Z. (ob X Y ∧ (∀w. X w  ⟶ Z w)) ⟶ ob Z (λw. (Z w ∧ ¬X w) ∨ Y w)" by  (smt ax_5d  ax_5b ax_5b'')
  thus ?thesis  using ax_5b by fastforce qed

(* Relationship between "❙O⇩a,❙O⇩p,❙□⇩a,❙□⇩p" *)
lemma C_13_a: "⌊❙□⇩aA ❙→ (❙¬❙O⇩aA ❙∧ ❙¬❙O⇩a(❙¬A))⌋"  by (metis (full_types) ax_5a ax_5b)
lemma C_13_b: "⌊❙□⇩pA ❙→ (❙¬❙O⇩pA ❙∧ ❙¬❙O⇩p(❙¬A))⌋"   by (metis (full_types) ax_5a ax_5b) 
lemma C_14_a: "⌊❙□⇩a(A ❙↔ B) ❙→ (❙O⇩aA ❙↔ ❙O⇩aB)⌋"  by (metis ax_5b)
lemma C_14_b: "⌊❙□⇩p(A ❙↔ B) ❙→ (❙O⇩pA ❙↔ ❙O⇩pB)⌋"  by (metis ax_5b)

(* Relationship between "❙O⇩,❙O⇩a,❙O⇩p,❙□⇩a,❙□⇩p" *)
lemma C_15_a: "⌊(❙O❙⟨B❙|A❙⟩ ❙∧ ❙□⇩aA ❙∧ ❙◇⇩aB ❙∧ ❙◇⇩a(❙¬B)) ❙→ ❙O⇩aB⌋"  using ax_5e by blast
lemma C_15_b: "⌊(❙O❙⟨B❙|A❙⟩ ❙∧ ❙□⇩pA ❙∧ ❙◇⇩pB ❙∧ ❙◇⇩p(❙¬B)) ❙→ ❙O⇩pB⌋"  using ax_5e by blast

(* Soundness and consistency *)
lemma II_3_1: "((⌊❙O❙⟨B❙|A❙⟩⌋) ∧ (∃x. Z(x) ∧ A(x) ∧ B(x))) ⟶ ob(Z)(A ❙→ B)" 
  proof 
    assume "(⌊❙O❙⟨B❙|A❙⟩⌋) ∧ (∃x. Z(x) ∧ A(x) ∧ B(x))"
     hence "ob (λz. A z ∧ Z z) (λz. A z ∧ Z z ∧ B z)" using ax_5e ax_5b  ax_5b' ax_5d by smt
     hence "ob (λz. Z z ∧ A z) (λz. Z z ∧ A z ∧ B z)" using ax_5e ax_5b  ax_5b' ax_5d by smt
     hence "ob Z (λw. (Z w ∧ ¬(Z w ∧ A w)) ∨ (Z w ∧ A w ∧ B w))" by (metis (mono_tags) ax_5d) 
     from this show  L19: "ob(Z)(A ❙→ B)" by (smt ax_5b)  qed

(* Some theorems and derived (proof) rules *)
lemma II_4_1: "⌊❙□(A ❙↔ B) ❙→ (C(A) ❙↔ C(B))⌋"  using ext by blast
lemma obs_II_4_1_a  : "⌊A ❙↔ B⌋ ⟹ ⌊C(A) ❙↔ C(B)⌋"  using ext by blast 
lemma obs_II_4_1_b  : "⌊A ❙↔ B⌋ ⟹ ⌊(❙◇(A ❙∧ C) ❙∧ ❙O❙⟨C❙|B❙⟩) ❙→ ❙O❙⟨C❙|A❙⟩⌋"  using ax_5e by blast
lemma obs_II_4_1_c_1: "⌊❙◇(❙O❙⟨B❙|A❙⟩) ❙→ ❙◇(❙□(❙O❙⟨B❙|A❙⟩))⌋"  by blast
lemma obs_II_4_1_c_2: "⌊❙◇(❙□(❙O❙⟨B❙|A❙⟩)) ❙→ ❙◇(❙O❙⟨B❙|A❙⟩)⌋"  by auto
lemma obs_II_4_1_c_3: "⌊❙◇(❙O❙⟨B❙|A❙⟩) ❙→ ❙□(❙O❙⟨B❙|A❙⟩)⌋"  by blast
lemma obs_II_4_1_c_4: "⌊❙◇(❙¬(❙O❙⟨B❙|A❙⟩)) ❙→ ❙□(❙¬(❙O❙⟨B❙|A❙⟩))⌋"  by blast
lemma res_II_4_1_a_1: "⌊❙¬(❙O❙⟨❙⊥❙|A❙⟩)⌋"  by (simp add: ax_5a)  
lemma res_II_4_1_a_2: "⌊(❙◇⇩p(A ❙∧ B ❙∧ C) ❙∧ ❙O❙⟨B❙|A❙⟩ ❙∧ ❙O❙⟨C❙|A❙⟩) ❙→ ❙O❙⟨(B ❙∧ C)❙|A❙⟩⌋"  using C_3  by auto
lemma res_II_4_1_a_3: "⌊❙O❙⟨B❙|A❙⟩ ❙→ ❙O❙⟨B❙|(A ❙∧ B)❙⟩⌋"  by (smt ax_5a ax_5b ax_5e)
lemma res_II_4_1_a_4: "⌊❙◇⇩p(❙O❙⟨B❙|A❙⟩) ❙→ ❙□⇩p(❙O❙⟨B❙|(A ❙∧ B)❙⟩)⌋"  by (smt ax_5a ax_5b ax_5e)
lemma res_II_4_1_a_5: "⌊(❙◇⇩p(A ❙∧ B ❙∧ C) ❙∧ ❙O❙⟨C❙|A❙⟩) ❙→ ❙O❙⟨C❙|(A ❙∧ B)❙⟩⌋"  by (smt ax_5a ax_5b ax_5e)
lemma res_II_4_1_b_1:  "⌊A ❙↔ B⌋ ⟹ ⌊❙O❙⟨C❙|A❙⟩ ❙↔ ❙O❙⟨C❙|B❙⟩⌋"  by (smt ax_5a ax_5b ax_5e)
lemma res_II_4_1_b_2:  "⌊C ❙→ (A ❙↔ B)⌋ ⟹ ⌊❙O❙⟨A❙|C❙⟩ ❙↔ ❙O❙⟨B❙|C❙⟩⌋"  by (smt ax_5b)
lemma obs_II_4_2_1: "⌊(❙O❙⟨B❙|A❙⟩ ❙∧ ❙◇⇩a(A ❙∧ B) ❙∧ ❙◇⇩a(A ❙∧ ❙¬B)) ❙→ (❙O❙⟨B❙|A❙⟩ ❙∧ ❙◇⇩a(A ❙→ B) ❙∧ ❙◇⇩a(❙¬(A ❙→ B)))⌋"  by blast
lemma obs_II_4_2_2: "⌊❙O❙⟨B❙|A❙⟩ ❙→ ❙O❙⟨(A ❙→ B)❙|❙⊤❙⟩⌋" by (simp add: C_8)
lemma obs_II_4_2_3: "⌊(❙O❙⟨(A ❙→ B)❙|❙⊤❙⟩ ❙∧ ❙□⇩a❙⊤ ❙∧ ❙◇⇩a(A ❙→ B) ❙∧ ❙◇⇩a(❙¬(A ❙→ B))) ❙→ ❙O⇩a(A ❙→ B)⌋"  by (smt ax_5e)
lemma obs_II_4_2_4: "⌊❙□⇩a❙⊤⌋"  by simp
lemma obs_II_4_2_5: "⌊(❙O❙⟨(A ❙→ B)❙|❙⊤❙⟩ ❙∧ ❙◇⇩a(A ❙→ B) ❙∧ ❙◇⇩a(❙¬(A ❙→ B))) ❙→ ❙O⇩a(A ❙→ B)⌋"  by (smt ax_5e)
lemma obs_II_4_2_6: "⌊(❙O❙⟨B❙|A❙⟩ ❙∧ ❙◇⇩a(A ❙∧ B) ❙∧ ❙◇⇩a(A ❙∧ ❙¬B)) ❙→ ❙O⇩a(A ❙→ B)⌋"   by (simp add: II_3_1)  
lemma obs_II_4_2_6_p: "⌊(❙O❙⟨B❙|A❙⟩ ❙∧ ❙◇⇩p(A ❙∧ B) ❙∧ ❙◇⇩p(A ❙∧ ❙¬B)) ❙→ ❙O⇩p(A ❙→ B)⌋"   by (simp add: II_3_1)  

lemma Oa_C: "⌊❙◇⇩a(A ❙∧ B) ❙∧ ❙O⇩aA ❙∧ ❙O⇩aB ❙→  ❙O⇩a(A ❙∧ B)⌋" using ax_5c by auto
lemma Op_C: "⌊❙◇⇩p(A ❙∧ B) ❙∧ ❙O⇩pA ❙∧ ❙O⇩pB ❙→  ❙O⇩p(A ❙∧ B)⌋" using ax_5c by auto
lemma Oa_DD: "⌊(❙O⇩aA ❙∧ ❙O❙⟨B❙|A❙⟩ ❙∧ ❙◇⇩a(A ❙∧ B)) ❙→ ❙O⇩a(A ❙∧ B)⌋" using ax_5b ax_5c obs_II_4_2_6 by smt 
declare [[smt_timeout=300]]
lemma Op_DD: "⌊(❙O⇩pA ❙∧ ❙O❙⟨B❙|A❙⟩ ❙∧ ❙◇⇩p(A ❙∧ B)) ❙→ ❙O⇩p(A ❙∧ B)⌋" using ax_5b ax_5c obs_II_4_2_6_p by smt
end
```
