# Supplementary material for: LogiKEy workbench: Deontic logics, logic combinations and expressive ethical and legal reasoning (Isabelle/HOL dataset)
Source: Data Brief. 2020 Oct 15;33:106409. doi: 10.1016/j.dib.2020.106409 (PMC7586073; doi:10.1016/j.dib.2020.106409)
Supplement: Supplementary file 1 [file mmc1.zip › 2020-DataInBrief-Data/index.html]

xml version="1.0" encoding="utf-8"?


Session MySession (Isabelle2019: June 2019)


# Session MySession

View theory dependencies

## Theories

- CJ\_DDL
- CJ\_DDL\_Tests
- CJ\_DDLplus
- Chisholm\_CJ\_DDL\_Dyadic
- Chisholm\_CJ\_DDL\_Monadic
- E
- Chisholm\_E
- SDL
- Chisholm\_SDL
- Extended\_CJ\_DDL
- GDPR\_CJ\_DDL
- GDPR\_E
- GDPR\_SDL
- GewirthArgument
- IOL\_out2
- IO\_out2\_STIT
- IO\_Experiments
- Lewis\_DDL
