# Supplementary material for: LogiKEy workbench: Deontic logics, logic combinations and expressive ethical and legal reasoning (Isabelle/HOL dataset)
Source: Data Brief. 2020 Oct 15;33:106409. doi: 10.1016/j.dib.2020.106409 (PMC7586073; doi:10.1016/j.dib.2020.106409)
Supplement: Supplementary file 1 [file mmc1.zip › 2020-DataInBrief-Data/Extended_CJ_DDL.html]

xml version="1.0" encoding="utf-8"?


Theory Extended\_CJ\_DDL (Isabelle2019: June 2019)


# Theory Extended\_CJ\_DDL

theory Extended\_CJ\_DDL  
imports CJ\_DDLplus

```
theory Extended_CJ_DDL                    (*David Fuenmayor and C. Benzmüller, 2019*)
  imports CJ_DDLplus
begin
nitpick_params[user_axioms=true, show_all, expect=genuine, format = 3]


section ‹Extending the Carmo and Jones DDL Logical Framework›

subsection ‹Context Features›

(**To keep things simple (and relevant for our task) we restrict ourselves to representing a context c as the pair: @{text "⟨Agent(c), World(c)⟩"}.
For this purpose we represent the functional concepts "Agent" and "World" as logical constants.*)
consts Agent::"c⇒e"  (**function retrieving the agent corresponding to context c*)   
consts World::"c⇒w"  (**function retrieving the world corresponding to context c*)

subsection ‹Logical Validity›

(**Kaplan's notion of (context-dependent) logical truth for a sentence corresponds to its (context-sensitive) formula
(of type @{text "c⇒w⇒bool"} i.e. m) being true in the given context and at its corresponding world.*)
abbreviation ldtruectx::"m⇒c⇒bool" ("⌊_⌋⇩_") where "⌊φ⌋⇩c ≡ φ c (World c)"

(**Kaplan's LD notion of logical validity for a sentence corresponds to its being true in all contexts.
This notion is also known as indexical validity.*)
abbreviation ldvalid::"m⇒bool" ("⌊_⌋⇧D") where "⌊φ⌋⇧D ≡ ∀c. ⌊φ⌋⇩c"

(**Here we show that indexical validity is indeed weaker than its classical modal counterpart (truth at all worlds for all contexts):*)
lemma "⌊A⌋ ⟹ ⌊A⌋⇧D" by simp
lemma "⌊A⌋⇧D ⟹ ⌊A⌋" nitpick oops (**countermodel found*)

(**Here we show that the interplay between indexical validity and the DDL modal and deontic operators does not
result in modal collapse.*)
lemma "⌊P ❙→ ❙O⇩aP⌋⇧D" nitpick oops
lemma "⌊P ❙→ ❙□⇩aP⌋⇧D" nitpick oops

(**Next we show that the necessitation rule does not work for indexical validity (in contrast to classical modal validity as defined for DDL).*)
lemma "⌊A⌋⇧D ⟹ ⌊❙□⇩aA⌋⇧D"  nitpick oops
lemma "⌊A⌋⇧D ⟹ ⌊❙□⇩pA⌋⇧D" nitpick oops

(**The following operator above is not part of the original Kaplan's LD and has been added
by us in order to better highlight some semantic features of our formalisation of Gewirth's argument in the next section and to being able to
use the necessitation rule for some inference steps. This is used to model a kind of logical-indexical necessity in contrast
to the alethic necessity shown above.*)
abbreviation ldvalidbox :: "m⇒m" ("❙□⇧D_" [52]53) where "❙□⇧Dφ ≡ λc w. ⌊φ⌋⇧D" (**note the D superscript*)
lemma "⌊❙□⇧Dφ⌋⇩C ≡ ∀c.⌊φ⌋⇩c" by simp (**works analogously to the box operator in modal logic S5*)

(**The necessitation rule works for the combination of indexical validity with the previous operator, as intended.*)
lemma "⌊A⌋⇧D ⟹ ⌊❙□⇧DA⌋⇧D"  by simp

subsection ‹Quantification›
(** We also enrich our logic with (higher-order) quantifiers (using parameterized types).*)
abbreviation mforall::"('t⇒m)⇒m" ("❙∀") where "❙∀Φ ≡ λc w.∀x. (Φ x c w)"
abbreviation mexists::"('t⇒m)⇒m" ("❙∃") where "❙∃Φ ≡ λc w.∃x. (Φ x c w)"    
abbreviation mforallBinder::"('t⇒m)⇒m" (binder"❙∀"[8]9) where "❙∀x. (φ x) ≡ ❙∀φ"  
abbreviation mexistsBinder::"('t⇒m)⇒m" (binder"❙∃"[8]9) where "❙∃x. (φ x) ≡ ❙∃φ"

(*<*)
end
(*>*)
```
