# Supplementary material for: LogiKEy workbench: Deontic logics, logic combinations and expressive ethical and legal reasoning (Isabelle/HOL dataset)
Source: Data Brief. 2020 Oct 15;33:106409. doi: 10.1016/j.dib.2020.106409 (PMC7586073; doi:10.1016/j.dib.2020.106409)
Supplement: Supplementary file 1 [file mmc1.zip › 2020-DataInBrief-Data/GDPR_SDL.html]

xml version="1.0" encoding="utf-8"?


Theory GDPR\_SDL (Isabelle2019: June 2019)


# Theory GDPR\_SDL

theory GDPR\_SDL  
imports SDL

```
theory GDPR_SDL  imports SDL  (*GDPR CTD Example. Benzmüller & Parent, 2019*)
begin
(*Unimportant.*) sledgehammer_params [max_facts=20,timeout=20,verbose] 
(*Unimportant.*) nitpick_params [user_axioms,show_all,dont_box] 

datatype data = d1 | d2  (*We exemplarily introduce concrete data objects d1 and d2.*)
datatype indiv = Mary | Peter  (*We exemplarily introduce individuals Mary and Peter.*)
consts process_lawfully::"data⇒σ" erase::"data⇒σ" is_protected_by_GDPR::"data⇒σ" 
            belongs_to::"data⇒indiv⇒σ" is_european::"indiv=>σ" kill::"indiv⇒σ"

axiomatization where   
(*Data belonging to Europeans is protected by the GDPR.*)
 A0: "⌊❙∀x. ❙∀d. (is_european x ❙∧ belongs_to d x) ❙→ is_protected_by_GDPR d⌋" and
(*Data d1 is belonging to the European Peter.*)
 F1: "⌊belongs_to d1 Peter ❙∧ is_european Peter⌋" and

(*It is an obligation to process data lawfully.*)
 A1: "⌊❙∀d. is_protected_by_GDPR d ❙→ ❙○<process_lawfully d>⌋"  and
(*If data was not processed lawfully, then it is an obligation to erase the data.*)
 A2: "⌊❙∀d.  (is_protected_by_GDPR d  ❙∧ ❙¬process_lawfully d) ❙→ ❙○<erase d>⌋" and
(*Implicit: It is an obligation to keep the data if it was processed lawfully.*)
 A3: "⌊❙∀d. ❙○<(is_protected_by_GDPR d ❙∧ process_lawfully d) ❙→ ❙¬erase d>⌋" and
(*Given a situation where data is processed unlawfully.*) 
 Situation: "⌊❙¬process_lawfully d1⌋⇩l" 

(***Some Experiments***) 
 lemma True nitpick [satisfy] oops (*Consistency-check: Nitpick finds no model.*)
 lemma False by (metis A0 F1 A1 A2 A3 Situation D) (*Prove of Falsum.*)

(*Should the data be erased? — Yes, proof found by ATPs*)
 lemma "⌊❙○<erase d1>⌋⇩l"  sledgehammer by (metis A0 A2 F1 Situation) 
(*Should the data be kept? — Yes, proof found by ATPs*)
 lemma "⌊❙○<❙¬erase d1>⌋⇩l" sledgehammer by (meson A0 A1 F1 A3)
(*Should Mary be killed? — Yes, proof found by ATPs*)
 lemma "⌊❙○<kill Mary>⌋⇩l" sledgehammer by (meson A0 A1 A2 F1 A3 Situation)
end
```
