# Supplementary material for: LogiKEy workbench: Deontic logics, logic combinations and expressive ethical and legal reasoning (Isabelle/HOL dataset)
Source: Data Brief. 2020 Oct 15;33:106409. doi: 10.1016/j.dib.2020.106409 (PMC7586073; doi:10.1016/j.dib.2020.106409)
Supplement: Supplementary file 1 [file mmc1.zip › 2020-DataInBrief-Data/IO_out2_STIT.html]

xml version="1.0" encoding="utf-8"?


Theory IO\_out2\_STIT (Isabelle2019: June 2019)


# Theory IO\_out2\_STIT

theory IO\_out2\_STIT  
imports Main

```
theory IO_out2_STIT imports Main                (*Paul Meder, 2018*)
begin
  typedecl i (* "type for possible worlds" *)
  type_synonym e = "(i⇒bool)"

  consts a1::"i ⇒ i ⇒ bool" a2::"i ⇒ i ⇒ bool"   (* Relation for agents: a1 & a2 *)
  consts r_box :: "i⇒i⇒bool"  (infixr "rbox" 70)  (* Relation R_Box *)
  consts r_agt :: "i⇒i⇒bool" (infixr "ragt" 70)  (* Relation R_Agt *)
  consts r_G :: "i⇒i⇒bool"    (infixr "rG" 70 )  (* Relation R_G *)
  consts r_H :: "i⇒i⇒bool"    (infixr "rH" 70 )   (* Relation R_H *)
  consts r_45 :: "i⇒i⇒bool" (infixr "r45" 70)   (* relation for a modal logic K45 *)
  consts aw :: i   (*actual world *)

  definition knot :: "e⇒e" ("❙¬_"[52]53) where "❙¬φ ≡ λw. ¬φ(w) " (* Negation *)
  definition kor :: "e⇒e⇒e" (infixr "❙∨"50) where "φ❙∨ψ ≡ λw. φ(w) ∨ ψ(w)"  (* Disjunction *)
  definition kand :: "e⇒e⇒e" (infixr "❙∧"51) where "φ❙∧ψ ≡ λw. φ(w) ∧ ψ(w)" (* Conjunction *)
  definition kimp :: "e⇒e⇒e" (infixr "❙⊃" 49) where "φ❙⊃ψ ≡ λw. φ(w) ⟶ ψ(w)" (* Implication *)
 
  definition k45box :: "e⇒e" ("❙□⇩l") where " ❙□⇩l φ ≡ λw. ∀v. w r45 v ⟶ φ(v)" (*□ for Modal logic K45*)
  definition k45dia :: "e⇒e" ("❙◇⇩l") where "❙◇⇩l φ ≡ ❙¬ ❙□⇩l( ❙¬ φ) " (* Dual operator for □ *)

  definition kbox :: "e⇒e" ("❙□") where "❙□φ ≡ λw. ∀v. w rbox v ⟶ φ(v)" (* □ for T-STIT logic*)
  definition kdia :: "e⇒e" ("❙◇") where "❙◇ φ ≡ ❙¬ ❙□( ❙¬ φ) " (* Dual *)
  definition kcstit :: "(i⇒i⇒bool)⇒e⇒e" ("_ cstit _") where "r cstit φ ≡ λw. ∀v. r w v ⟶ φ(v) " (* STIT [i]  *)
  definition kgstit :: "e⇒e" ("gstit _") where "gstit φ ≡ λw. ∀v. w ragt v ⟶ φ(v)" (*Group STIT [Agt]*)

  definition kvalid :: "e⇒bool" ("⌊_⌋" [8]109) where "⌊p⌋ ≡ ∀w. p(w)" (* Validity *)
  definition actual :: "e ⇒ bool" ("⌊_⌋⇩l"[7]105) where "⌊φ⌋⇩l ≡ φ(aw)" (* Validity for actual world *)


  definition ktrue  :: "e" ("❙⊤") where "❙⊤ ≡ λw. True" (* Tautology *)
  definition kfalse :: "e" ("❙⊥") where "❙⊥ ≡ λw. False"  (* Falsum *)

  definition kfuture :: "e⇒e" ("F _") where "F φ ≡ λw. ∀v. w rG v ⟶ φ(v)"
  definition kpast :: "e⇒e" ("H _") where "H φ ≡ λw. ∀v. w rH v ⟶ φ(v)"
 (* P - dual of the past tense operator H
    Pφ stands for 'φ has been true at some point in the past ' *)
  definition kpastpoint :: "e⇒e" ("P _")
    where "P φ ≡ ❙¬ H ❙¬ φ"
  definition kdstit :: "(i⇒i⇒bool)⇒e⇒e" ("_ dstit _") where "r dstit φ ≡ (r cstit φ)  ❙∧ ❙¬ ❙□ φ"

  definition union_set :: "(e⇒bool)⇒(e⇒bool)⇒(e⇒bool)"  ("_ ❙∪ _")
  where "A ❙∪ B ≡ λw. (A w) ∨ (B w)"

  named_theorems Defs
  declare knot_def[Defs] kor_def[Defs] kand_def[Defs] kimp_def[Defs] kvalid_def[Defs]
          kbox_def[Defs] kdia_def[Defs] kcstit_def[Defs] kdstit_def[Defs] kgstit_def[Defs] 
          kfuture_def[Defs] kpast_def[Defs] kpastpoint_def[Defs] ktrue_def[Defs] kfalse_def[Defs]
             union_set_def[Defs] 
          k45box_def[Defs] k45dia_def[Defs]

 (* Subset for relations *)
 abbreviation subset_rel :: "(i⇒i⇒bool)⇒(i⇒i⇒bool)⇒bool"  ("_ ❙⊆ _")
   where "r1 ❙⊆ r2 ≡ ∀w. ∀v. (r1 w v) ⟶ (r2 w v) "
 (* Composition of 2 relations *)
 abbreviation comp_rel :: "(i⇒i⇒bool)⇒(i⇒i⇒bool)⇒(i⇒i⇒bool)"  ("_ ❙∘ _")
   where "r1 ❙∘ r2 ≡ λw. λv. ∃u.( (r1 w u) ∧ (r2 u v))"
 (* Intersection between 2 relations *)
 abbreviation intersection_rel :: "(i⇒i⇒bool)⇒(i⇒i⇒bool)⇒(i⇒i⇒bool)"  ("_ ❙∩ _")
  where "r1 ❙∩ r2 ≡ λw. λv. (r1 w v) ∧ (r2 w v)"
 (* Reflexive relation *)
 abbreviation reflexive 
   where "reflexive r ≡ (∀x. r x x)"
 (* Symmetric relation *)
 abbreviation symmetric 
   where "symmetric r ≡ (∀x y. r x y ⟶ r y x)"
 (* Transitive relation *)
 abbreviation transitive 
   where "transitive r ≡ (∀x y z. ((r x y) ∧ (r y z) ⟶ (r x z)))"
 (* Serial relation *)
 abbreviation serial 
   where "serial r ≡ (∀w. ∃v. r w v)"
 (* Inverse relation *)
 abbreviation inverse :: "(i⇒i⇒bool)⇒(i⇒i⇒bool)⇒bool"
  where "inverse r s ≡ ∀w. ∀v. (r w v) ⟷ (s v w)"
 abbreviation euclidean
   where "euclidean rel ≡ (∀x y z. ((rel x y) ∧ (rel x z) ⟶ (rel y z)))"


 (* Abbrevations for conditions on the relations *)
 abbreviation axC1
   where "axC1 r ≡ r ❙⊆ r_box"
 abbreviation axC2 :: "(i⇒i⇒bool)⇒(i⇒i⇒bool)⇒bool" 
   where "axC2 r s ≡ ( ∀w. ∀v. ((w rbox w) ∧ (v rbox v) 
                                  ∧ (w rbox v) ∧ (v rbox w))
                         ⟶ (∃x. r w x ∧ s v x))"
 abbreviation axC3 :: "(i⇒i⇒bool)⇒(i⇒i⇒bool)⇒bool"
   where "axC3 r s ≡ (∀w.∀v. (w ragt v) = (r w v ∧ s w v))" 

axiomatization where
  (* r45 fulfills transitivity and euclideaness *)
 (* ax_reflex_r45 : "reflexive r_45" *)
  ax_trans_r45 : "transitive r_45" and
  ax_euclidean_r45 : "euclidean r_45" 

lemma  "⌊ ( ❙□⇩lφ)  ❙⊃ ( ❙□⇩l ( ❙□⇩l φ))⌋" 
  by (smt ax_trans_r45 k45box_def kimp_def kvalid_def)

lemma  "⌊ (❙◇⇩lφ)  ❙⊃ ( ❙□⇩l(❙◇⇩l φ))⌋"
  by (smt ax_euclidean_r45 k45box_def k45dia_def kimp_def knot_def kvalid_def)

axiomatization where
  (* rbox is an equivalence relation between the worlds in W *)
  ax_refl_rbox: "reflexive r_box" and
  ax_sym_rbox : "symmetric r_box" and
  ax_trans_rbox : "transitive r_box" and

  (* ragt is an equivalence relation between the worlds in W *)
  ax_refl_ragt: "reflexive r_agt" and
  ax_sym_ragt: "symmetric r_agt" and
  ax_trans_ragt: "transitive r_agt" and

  (* every r is an equivalence relation between the worlds in W *)
  ax_refl_a1:  "reflexive a1" and
  ax_sym_a1: "symmetric a1" and
  ax_trans_a1:  "transitive a1" and

  ax_refl_a2:  "reflexive a2" and
  ax_sym_a2: "symmetric a2" and
  ax_trans_a2:  "transitive a2" and

  (*  rG and rH are binary relations between worlds in W  such that 
    rG is serial and transitive, 
    rH is the inverse relation of rG *)
 (* ax_ser_rG: "serial r_G" and *)
  ax_trans_rG: "transitive r_G" and
  ax_inverse_rG_rH: "inverse r_G r_H" 
lemma ax_1: "∃w. w=w" by simp (* W is a nonempty set of possible worlds *)

axiomatization where
  axC1_a1: "axC1 a1" 

axiomatization where
  axC1_a2: "axC1 a2" 

axiomatization where
  axC2_a1_a2 : "axC2 a1 a2 " 

axiomatization where
  axC3_a1_a2 : "axC3 a1 a2 "

lemma True nitpick [satisfy,user_axioms,show_all] oops

axiomatization where
  ax_C4 : "∀w. ∀v. ∀u. ((w rG u) ∧ (w rG v))
           ⟶ ((u rG v) ∨ (v rG u) ∨ (u=v))" 

lemma True nitpick [satisfy,user_axioms,show_all] oops

axiomatization where
  ax_C5 : "∀w. ∀v. ∀u. ((w rH u) ∧ (w rH v))
           ⟶ ((u rH v) ∨ (v rH u) ∨ (u=v))" 

lemma True nitpick [satisfy,user_axioms,show_all] oops

axiomatization where
  ax_C6 : "(r_G ❙∘ r_box) ❙⊆ (r_agt ❙∘ r_G)" 

axiomatization where
  ax_C7 : "∀w.∀v. (w rbox v) ⟶ ¬ (w rG v)" 

lemma True nitpick [satisfy,user_axioms,show_all] oops

(* Some Test *)

consts a::e b::e x::e y::e
(* K = {(a, [a1 cstit x])}, A = {a} *)
(* [a1 cstit x] ∈ out2(K,a) *)
lemma "⌊ ((a ❙⊃ ❙□⇩l(a1 cstit x)) ❙∧ (a)) ❙⊃ ❙□⇩l(a1 cstit x) ⌋ ∧ ⌊(a1 cstit x)❙⊃ (a1 cstit x) ⌋" 
  by (simp add: kand_def kimp_def kvalid_def)

(* WO rule of I/O STIT logic with out2 *)
(*  x ∈ out2(K,A) *)
lemma "⌊ ((a ❙⊃ ❙□⇩l(a1 cstit x)) ❙∧ (a)) ❙⊃ ❙□⇩l(x) ⌋ ∧ ⌊(a1 cstit x)❙⊃ (x) ⌋" 
  using ax_refl_a1 k45box_def kand_def kcstit_def kimp_def kvalid_def by auto 
(* ❙◇[a1 cstit x] ∈ out2(K,A) *)
lemma "⌊ ((a ❙⊃ ❙□⇩l(a1 cstit x)) ❙∧ (a)) ❙⊃ ❙□⇩l(❙◇(a1 cstit x)) ⌋ ∧ ⌊(a1 cstit x)❙⊃ (❙◇(a1 cstit x)) ⌋"
   using ax_refl_rbox k45box_def kand_def kbox_def kdia_def kimp_def knot_def kvalid_def by auto
(* [a1 cstit (x∨y)] ∈ out2(K,A) *)
lemma "⌊((a ❙⊃ ❙□⇩l(a1 cstit (x)))❙∧(a)) ❙⊃ ❙□⇩l(a1 cstit (x❙∨y)) ⌋ ∧ ⌊(a1 cstit x)❙⊃ (a1 cstit (x❙∨y))⌋" 
  by (simp add: k45box_def kand_def kcstit_def kimp_def kor_def kvalid_def)

(* SI rule for I/O STIT with out2 *)
(* [a1 cstit x] ∈ out2(K,{a∧b}) *)
lemma "⌊ ((a ❙⊃ ❙□⇩l(a1 cstit x)) ❙∧ (a ❙∧ b)) ❙⊃ ❙□⇩l(a1 cstit x) ⌋ ∧ ⌊(a1 cstit x)❙⊃ (a1 cstit x) ⌋" 
  by (simp add: kand_def kimp_def kvalid_def)

definition "G ≡ (λX. X=(a, (a1 cstit x)))" declare G_def[Defs]
definition "A ≡(λX. X=a)" declare A_def[Defs]
definition "G_Box ≡ λx. ∃y z. ( (x=(y ❙⊃ ❙□⇩lz)) ∧ G(y,z)) " declare G_Box_def[Defs]

lemma "G_Box(a ❙⊃ ❙□⇩l(a1 cstit x))" 
  by (simp add: G_Box_def G_def)  

lemma "(G_Box ❙∪ A)(a)" 
  by (simp add: A_def union_set_def)

(*lemma "G_L G (a1 cstit x)"
  by (simp add: G_L_def G_def)*)

(* Sledgehammer times out / Nitpick no counter model *)
lemma "out2 G (a1 cstit x)" 
  nitpick[user_axioms,show_all] oops

lemma "⌊ ((a ❙⊃ ❙□⇩l(a1 cstit x)) ❙∧ (a)) ❙⊃ ❙□⇩l(a1 cstit x) ⌋"
  by (simp add: kand_def kimp_def kvalid_def)
consts z::e

(* K = {(a, [a1 cstit x])} A = {a} *)
definition "K ≡ (λX. X= (a, (a1 cstit x))) " declare K_def[Defs] 
(* WO rule of I/O STIT logic with out1 *)
(*out1 *)
(* [a1 cstit x] ∈ out1(K,a)  - timeout*)
lemma "out1 K a (a1 cstit x)"   nitpick[user_axioms,show_all] oops
(*  x ∈ out1(K,a)  - timeout*)
lemma "out1 K a x" nitpick[user_axioms,show_all] oops 
(* ❙◇[a1 cstit x] ∈ out1(K,a)  - timeout*)
lemma "out1 K a (❙◇(a1 cstit x))"  nitpick[user_axioms,show_all] oops 
(* [a1 cstit (x∨y)] ∈ out1(K,a)  - timeout*)
lemma "out1 K a (a1 cstit (x❙∨y))" nitpick[user_axioms,show_all] oops 

(* SI rule for I/O STIT with out1 *)
(* [a1 cstit x] ∈ out1(K,{a∧b})  - timeout*)
lemma "out1 K (a ❙∧ b) (a1 cstit x)" nitpick[user_axioms,show_all] oops 

(* M = {(a, [a1 cstit x]), (a, [a1 cstit y])} *)
definition "M ≡ (λX. X= (a, (a1 cstit x)) ∨ X=(a, (a1 cstit y))) " declare M_def[Defs] 
(* AND rule for I/O STIT  with out1*)
(* [a1 cstit x] ∈ out1(M,a)  - timeout*)
lemma "out1 M a ((a1 cstit x)❙∧(a1 cstit y))"  nitpick[user_axioms,show_all] oops 
(* [a1 cstit (x∧y)] ∈ out2(M,a) - timeout  *)
lemma "out1 M a (a1 cstit (x❙∧y))"  nitpick[user_axioms,show_all] oops 

(* OR rule for I/O STIT with out1*)
(* T = {(a, [a1 cstit x]), (b, [a1 cstit x]))} *)
definition "T ≡ (λX. X= (a, (a1 cstit x)) ∨ X= (b, (a1 cstit x)))" declare T_def[Defs]

(* [a1 cstit x] ∈ out1(T,{a ∨ b})? No, countermodel*)
lemma "out1 T (a ❙∨ b) (a1 cstit x)"  nitpick[user_axioms,show_all] oops


(* K = {(a, [a1 cstit x])}, A = {a} *)
(* [a1 cstit x] ∈ out2(K,a) *)
lemma "⌊ ((a ❙⊃ ❙□⇩l(a1 cstit x)) ❙∧ (a)) ❙⊃ ❙□⇩l(a1 cstit x) ⌋ ∧ ⌊(a1 cstit x)❙⊃ (a1 cstit x) ⌋" 
  by (simp add: kand_def kimp_def kvalid_def)

(* WO rule of I/O STIT logic with out2 *)
(*  x ∈ out2(K,A) *)
lemma "⌊ ((a ❙⊃ ❙□⇩l(a1 cstit x)) ❙∧ (a)) ❙⊃ ❙□⇩l(x) ⌋ ∧ ⌊(a1 cstit x)❙⊃ (x) ⌋" 
  using ax_refl_a1 k45box_def kand_def kcstit_def kimp_def kvalid_def by auto 
(* ❙◇[a1 cstit x] ∈ out2(K,A) *)
lemma "⌊ ((a ❙⊃ ❙□⇩l(a1 cstit x)) ❙∧ (a)) ❙⊃ ❙□⇩l(❙◇(a1 cstit x)) ⌋ ∧ ⌊(a1 cstit x)❙⊃ (❙◇(a1 cstit x)) ⌋"
   using ax_refl_rbox k45box_def kand_def kbox_def kdia_def kimp_def knot_def kvalid_def by auto
(* [a1 cstit (x∨y)] ∈ out2(K,A) *)
lemma "⌊((a ❙⊃ ❙□⇩l(a1 cstit (x)))❙∧(a)) ❙⊃ ❙□⇩l(a1 cstit (x❙∨y)) ⌋ ∧ ⌊(a1 cstit x)❙⊃ (a1 cstit (x❙∨y))⌋" 
  by (simp add: k45box_def kand_def kcstit_def kimp_def kor_def kvalid_def)

(* SI rule for I/O STIT with out2 *)
(* [a1 cstit x] ∈ out2(K,{a∧b}) *)
lemma "⌊ ((a ❙⊃ ❙□⇩l(a1 cstit x)) ❙∧ (a ❙∧ b)) ❙⊃ ❙□⇩l(a1 cstit x) ⌋ ∧ ⌊(a1 cstit x)❙⊃ (a1 cstit x) ⌋" 
  by (simp add: kand_def kimp_def kvalid_def)

(* AND rule for I/O STIT with out2 *)
(* M = {(a, [a1 cstit x]), (a, [a1 cstit y])} *)
(* ([a1 cstit (x)] ∧ [a1 cstit (y)]) ∈ out2(M,a) *)
lemma "⌊ ((a ❙⊃ ❙□⇩l(a1 cstit (x))) ❙∧ (a ❙⊃ ❙□⇩l(a1 cstit (y))) ❙∧(a)) ❙⊃ ❙□⇩l((a1 cstit x)❙∧(a1 cstit y)) ⌋
         ∧ ⌊((a1 cstit x)❙∧(a1 cstit y)) ❙⊃ ((a1 cstit x)❙∧(a1 cstit y)) ⌋"
  by (simp add: k45box_def kand_def kimp_def kvalid_def)
(* [a1 cstit (x∧y)] ∈ out2(M,a) *)
lemma "⌊ ((a ❙⊃ ❙□⇩l(a1 cstit (x))) ❙∧ (a ❙⊃ ❙□⇩l(a1 cstit (y))) ❙∧(a)) ❙⊃ ❙□⇩l(a1 cstit (x❙∧y)) ⌋
         ∧ ⌊((a1 cstit x)❙∧(a1 cstit y)) ❙⊃ (a1 cstit (x❙∧y)) ⌋"
  by (simp add: k45box_def kand_def kcstit_def kimp_def kvalid_def)

(* OR rule for the I/O STIT with out2 *)
(* T = {(a, [a1 cstit x]), (b, [a1 cstit x]))} *)
(* [a1 cstit x] ∈ out2(T,{a ∨ b}) *)
lemma "⌊ ((a ❙⊃ ❙□⇩l(a1 cstit x)) ❙∧ (b ❙⊃ ❙□⇩l(a1 cstit x)) ❙∧ (a ❙∨ b)) ❙⊃ ❙□⇩l(a1 cstit x) ⌋
       ∧ ⌊(a1 cstit x)❙⊃ (a1 cstit x) ⌋"
  using kand_def kimp_def kor_def kvalid_def by auto

(* ID
(*out1 *)
(* [a1 cstit x] ∈ out1(T,[a1 cstit x])? No, countermodel*)
lemma "out1 T (a1 cstit x) (a1 cstit x)"  nitpick[user_axioms,show_all] oops *)
(*out2 *)
(* [a1 cstit x] not ∈ out2(T,{[a1 cstit x]} *)
lemma  "⌊ ((a ❙⊃ ❙□⇩l(a1 cstit x)) ❙∧ (b ❙⊃ ❙□⇩l(a1 cstit x)) ❙∧ (a1 cstit x)) ❙⊃ ❙□⇩l(a1 cstit x) ⌋
       ∧ ⌊(a1 cstit x)❙⊃ (a1 cstit x) ⌋"   nitpick[user_axioms,show_all] oops
(* Cumulative Transitivity *)
definition "T1 ≡ (λX. X= (a1 cstit a, a1 cstit x) ∨ X = ((a1 cstit a) ❙∧ (a1 cstit x),a1 cstit y))"
(*out1 *)
(* [a1 cstit y] ∈ out1(T1, [a1 cstit a])? No, countermodel*)
lemma "out1 T1 (a1 cstit a) (a1 cstit y)"  nitpick[user_axioms,show_all] oops
(*out2 *)
(* [a1 cstit y] ∈ out2(T1, [a1 cstit a]) *)
lemma "⌊ (( (a1 cstit a ) ❙⊃ ❙□⇩l(a1 cstit x)) ❙∧ (((a1 cstit a) ❙∧ (a1 cstit x)) ❙⊃ ❙□⇩l(a1 cstit y))
           ❙∧ (a1 cstit a)) ❙⊃ ❙□⇩l(a1 cstit y) ⌋
       ∧ ⌊((a1 cstit x) ❙∧ (a1 cstit y))❙⊃ (a1 cstit y) ⌋"  nitpick[user_axioms,show_all] oops

definition "T2 ≡ (λX. X= (a1 cstit x, a1 cstit y) ∨ X = ((a1 cstit y),a1 cstit z))"
(*out1 *)
(* [a1 cstit z] ∈ out1(TT, [a1 cstit x])? No, countermodel*)
lemma "out1 T2 (a1 cstit x) (a1 cstit z)"  nitpick[user_axioms,show_all] oops
(*out2 *)
(* [a1 cstit z] ∈ out2(T2, [a1 cstit x]) *)
lemma "⌊ (( (a1 cstit x ) ❙⊃ ❙□⇩l(a1 cstit y)) ❙∧ ((a1 cstit y) ❙⊃ ❙□⇩l(a1 cstit z))
           ❙∧ (a1 cstit x)) ❙⊃ ❙□⇩l(a1 cstit z) ⌋
       ∧ ⌊((a1 cstit y) ❙∧ (a1 cstit z))❙⊃ (a1 cstit z) ⌋"  nitpick[user_axioms,show_all] oops

definition "S ≡ (λX. X= (a, (a1 cstit x)) ∨ X=(a, (a2 cstit y))) " 
declare S_def[Defs] 
(*out1 *)
(* [gstit (x ∧ y)] ∈ out1(S,a)? Timeout*)
lemma "out1 S (a) (gstit (x ❙∧ y))" nitpick[user_axioms,show_all] oops
(*out2 *)
(* [gstit (x ∧ y)] ∈ out2(S,a) *)
lemma "⌊ ((a ❙⊃ ❙□⇩l(a1 cstit (x) )) ❙∧ (a ❙⊃ ❙□⇩l(a2 cstit (y) )) ❙∧(a)) ❙⊃ ❙□⇩l(gstit (x ❙∧ y) ) ⌋
         ∧ ⌊((a1 cstit x)❙∧(a2 cstit y)) ❙⊃ (gstit (x ❙∧ y)) ⌋"
  by (simp add: axC3_a1_a2 k45box_def kand_def kcstit_def kgstit_def kimp_def kvalid_def) 


definition "W ≡ (λX. X= (a,gstit z))" declare W_def[Defs]
(*out1 *)
(* [a1 cstit z] ∧ [a2 cstit z] ∈ out1(W,a)? No*)
lemma "out1 W (a) ((a1 cstit z) ❙∧ (a2 cstit z))" nitpick[user_axioms,show_all] oops
(*out2 *)
(* [a1 cstit z] ∧ [a2 cstit z] ∈ out2(W,a) ? 
  No, we dont have  [gstit z] ⊃ ([a1 cstit z] ∧ [a2 cstit z])   *)
lemma "⌊ ((a ❙⊃ ❙□⇩l(gstit (z)))❙∧(a)) ❙⊃ ❙□⇩l((a1 cstit z)❙∧(a2 cstit z)) ⌋
         ∧ ⌊(gstit z) ❙⊃ ((a1 cstit z) ❙∧ (a2 cstit z)) ⌋" unfolding Defs nitpick[user_axioms,show_all] oops 

definition "L ≡ (λX. X= (a, (a1 cstit x)) ∨ X=(a, (a2 cstit x))) " declare L_def[Defs] 
(* [gstit x] ∈ out2(L,a) *)
lemma "⌊ ((a ❙⊃ ❙□⇩l(a1 cstit (x) )) ❙∧ (a ❙⊃ ❙□⇩l(a2 cstit (x) )) ❙∧(a)) ❙⊃ ❙□⇩l(gstit x) ⌋
         ∧ ⌊((a1 cstit x)❙∧(a2 cstit x)) ❙⊃ (gstit x) ⌋" 
  by (simp add: axC3_a1_a2 k45box_def kand_def kcstit_def kgstit_def kimp_def kvalid_def)

(* Some test with ❙□⇩l and ❙□ *)
definition "R ≡ (λX. X = (a, ❙□x))" declare R_def[Defs]
(* ❙□ x ∈ out1(R,a) - Timeout *)
lemma "out1 R (a) (❙□x)" nitpick[user_axioms,show_all] oops
(* ❙□ x ∈ out2(R,a) *)
lemma "⌊ ((a ❙⊃ ❙□⇩l(❙□ x)) ❙∧ (a))❙⊃ ❙□⇩l(❙□ x)  ⌋ ∧ ⌊ (❙□ x)❙⊃ (❙□ x) ⌋"
  by (simp add: kand_def kimp_def kvalid_def)
(* ◇x ∈ out1(R,a) - Timeout *)
lemma "out1 R (a) (❙◇x)" nitpick[user_axioms,show_all] oops
(* ◇x ∈ out2(R,a) *)
lemma "⌊ ((a ❙⊃ ❙□⇩l(❙□ x)) ❙∧ (a))❙⊃ ❙□⇩l(❙◇ x)  ⌋ ∧ ⌊ (❙□ x)❙⊃ (❙◇ x) ⌋"
  using ax_refl_rbox k45box_def kand_def kbox_def kdia_def kimp_def knot_def kvalid_def by auto
(* x ∈ out1(R,a) - Timeout *)
lemma "out1 R (a) (x)" nitpick[user_axioms,show_all] oops
(* x ∈ out2(R,a) *)
lemma "⌊ ((a ❙⊃ ❙□⇩l(❙□ x)) ❙∧ (a))❙⊃ ❙□⇩l( x)  ⌋ ∧ ⌊ (❙□ x)❙⊃ ( x) ⌋" 
  by (simp add: ax_refl_rbox k45box_def kand_def kbox_def kimp_def kvalid_def)
(* (x ❙∨ y) ∈ out1(R,a) - Timeout *)
lemma "out1 R (a) (x ❙∨ y)" nitpick[user_axioms,show_all] oops
(* (x ∨ y) ∈ out2(R,a) *)
lemma "⌊ ((a ❙⊃ ❙□⇩l(❙□ x)) ❙∧ (a))❙⊃ ❙□⇩l( x ❙∨ y)  ⌋ ∧ ⌊ (❙□ x)❙⊃ (x ❙∨ y) ⌋" 
  by (simp add: ax_refl_rbox k45box_def kand_def kbox_def kimp_def kor_def kvalid_def)
(* [a1 cstit x] ∈ out1(R,a) - Timeout *)
lemma "out1 R (a) ( a1 cstit x)" nitpick[user_axioms,show_all] oops
(* [a1 cstit x] ∈ out2(R,a) *)
lemma "⌊ ((a ❙⊃ ❙□⇩l(❙□ x)) ❙∧ (a))❙⊃ ❙□⇩l( a1 cstit x)  ⌋"
  by (simp add: axC1_a1 k45box_def kand_def kbox_def kcstit_def kimp_def kvalid_def)
lemma "⌊ (❙□ x)❙⊃ (a1 cstit x) ⌋" 
  by (simp add: axC1_a1 kbox_def kcstit_def kimp_def kvalid_def) 
(* [a2 cstit x] ∈ out1(R,a) - Timeout *)
lemma "out1 R (a) ( a2 cstit x)" nitpick[user_axioms,show_all] oops
(* [a2 cstit x] ∈ out2(R,a) *)
lemma "⌊ ((a ❙⊃ ❙□⇩l(❙□ x)) ❙∧ (a))❙⊃ ❙□⇩l( a2 cstit x)  ⌋"
  by (simp add: axC1_a2 k45box_def kand_def kbox_def kcstit_def kimp_def kvalid_def)
lemma "⌊ (❙□ x)❙⊃ (a2 cstit x) ⌋"
  by (simp add: axC1_a2 kbox_def kcstit_def kimp_def kvalid_def)
(* ([a1 cstit x] ∧ [a2 cstit x]) ∈ out1(R,a) - Timeout *)
lemma "out1 R (a) ((a1 cstit x) ❙∧ (a2 cstit x))" nitpick[user_axioms,show_all] oops
(* ([a1 cstit x] ∧ [a2 cstit x]) ∈ out2(R,a) *)
lemma "⌊ ((a ❙⊃ ❙□⇩l(❙□ x)) ❙∧ (a))❙⊃ ❙□⇩l((a1 cstit x) ❙∧ (a2 cstit x))  ⌋"
  by (simp add: axC1_a1 axC1_a2 k45box_def kand_def kbox_def kcstit_def kimp_def kvalid_def)
lemma "⌊ (❙□ x)❙⊃ ((a1 cstit x) ❙∧ (a2 cstit x)) ⌋" 
  by (simp add: axC1_a1 axC1_a2 kand_def kbox_def kcstit_def kimp_def kvalid_def)
(* [gstit x] ∈ out1(R,a) - Timeout *)
lemma "out1 R (a) (gstit x)" nitpick[user_axioms,show_all] oops
(* [gstit x] ∈ out2(R,a) *)
lemma "⌊ ((a ❙⊃ ❙□⇩l(❙□ x)) ❙∧ (a))❙⊃ ❙□⇩l(gstit x)  ⌋"
  by (simp add: axC1_a2 axC3_a1_a2 k45box_def kand_def kbox_def kgstit_def kimp_def kvalid_def)
lemma "⌊ (❙□ x)❙⊃ (gstit x) ⌋"
  by (simp add: axC1_a1 axC3_a1_a2 kbox_def kgstit_def kimp_def kvalid_def)
end
```
