# Supplementary material for: LogiKEy workbench: Deontic logics, logic combinations and expressive ethical and legal reasoning (Isabelle/HOL dataset)
Source: Data Brief. 2020 Oct 15;33:106409. doi: 10.1016/j.dib.2020.106409 (PMC7586073; doi:10.1016/j.dib.2020.106409)
Supplement: Supplementary file 1 [file mmc1.zip › 2020-DataInBrief-Data/IO_Experiments.html]

xml version="1.0" encoding="utf-8"?


Theory IO\_Experiments (Isabelle2019: June 2019)


# Theory IO\_Experiments

theory IO\_Experiments  
imports IO\_out2\_STIT

```
theory IO_Experiments imports IO_out2_STIT             (*Paul Meder, 2018*)
begin
(* Xin Paper - Example proof theory *)
consts e::e f::e
(* G = (a ∨ b, [a1 cstit e]) *)     (* [a1 cstit (e ∨ f)] ∈ out1(G, {a2 cstit b } *)
lemma "out1 (λX. X=(a ❙∨ b, (a1 cstit e))) (a2 cstit b) (a1 cstit (e ❙∨ f))" nitpick[user_axioms,show_all] oops

(* [a1 cstit (e ∨ f)] ∈ out2(G, {a2 cstit b } *) (* [a1 cstit (e ∨ f)] ∈ Cn(G(L))  *)
(* old version - times out *)
lemma " out2 (λX. X=(a ❙∨ b, (a1 cstit e))) (a1 cstit (e ❙∨ f)) " oops
(* new version - finds proof *)
lemma "⌊(a1 cstit e)❙⊃(a1 cstit (e ❙∨ f)) ⌋" 
  using kcstit_def kimp_def kor_def kvalid_def by auto

(* Modal logic part *)
lemma "⌊ (((a ❙∨ b) ❙⊃ ❙□⇩l(a1 cstit e)) ❙∧ (a2 cstit b)) ❙⊃ ❙□⇩l(a1 cstit (e ❙∨ f)) ⌋" 
  by (simp add: ax_refl_a2 k45box_def kand_def kcstit_def kimp_def kor_def kvalid_def)

(* together *)
lemma "⌊ (((a ❙∨ b) ❙⊃ ❙□⇩l(a1 cstit e)) ❙∧ (a2 cstit b)) ❙⊃ ❙□⇩l(a1 cstit (e ❙∨ f)) ⌋ ∧
       ⌊(a1 cstit e)❙⊃(a1 cstit (e ❙∨ f)) ⌋ " unfolding Defs 
  using ax_refl_a2 by blast

(* Xin Paper - Ross Paradox *)
(* We have that [a1 dstit (e ∨ f)] not in out2({(⊤, [a1 dstit e])}, {⊤}) *)
lemma "out1 (λX. X=(❙⊤, (a1 dstit e))) ❙⊤  (a1 dstit (e ❙∨ f))" nitpick[user_axioms,show_all] oops
(* We have that [a1 dstit (e ∨ f)] not in out2({(⊤, [a1 dstit e])}, {⊤}) *)
lemma "⌊ (❙⊤ ❙⊃ ❙□⇩l(a1 dstit e)) ❙⊃ ❙□⇩l(a1 dstit (e ❙∨ f))  ⌋"
  nitpick[user_axioms,show_all] oops

(* With Chellas's STIT, the paradax is not solved *)
lemma "out1 (λX. X=(❙⊤, (a1 cstit e))) ❙⊤  (a1 cstit (e ❙∨ f))" nitpick[user_axioms,show_all] oops
lemma "⌊ (❙⊤ ❙⊃ ❙□⇩l(a1 cstit e)) ❙⊃ ❙□⇩l(a1 cstit (e ❙∨ f))  ⌋ "
  by (simp add: k45box_def kcstit_def kimp_def kor_def ktrue_def kvalid_def)

(* out2 *)
(* G = {(a,[a1 cstit e]), (a,[a2 cstit f]),(b,e ∧ f)} *)
(* [a1 cstit e] ∈ out2(G,a) *)
lemma  "⌊ ((a ❙⊃ ❙□⇩l(a1 cstit e)) ❙∧ (a ❙⊃ ❙□⇩l(a1 cstit f))❙∧(b ❙⊃ ❙□⇩l(e❙∧f)) ❙∧ (a)) ❙⊃ ❙□⇩l(a1 cstit (e)) ⌋ ∧
       ⌊((a1 cstit e) ❙∧ (a1 cstit f) ❙∧ (e ❙∧ f))❙⊃(a1 cstit (e)) ⌋ "  unfolding Defs by simp
(*  e ∈ out2(G,a) *)
lemma  "⌊ ((a ❙⊃ ❙□⇩l(a1 cstit e)) ❙∧ (a ❙⊃ ❙□⇩l(a1 cstit f))❙∧(b ❙⊃ ❙□⇩l(e❙∧f)) ❙∧ (a)) ❙⊃ ❙□⇩l(e) ⌋ ∧
       ⌊((a1 cstit e) ❙∧ (a1 cstit f) ❙∧ (e ❙∧ f))❙⊃e ⌋ "  unfolding Defs 
  by (simp add: ax_refl_a1)
(*  (e∧f) ∈ out2(G,a) *)
lemma "⌊ ((a ❙⊃ ❙□⇩l(a1 cstit e)) ❙∧ (a ❙⊃ ❙□⇩l(a1 cstit f)) ❙∧(b ❙⊃ ❙□⇩l(e❙∧f)) ❙∧ (a)) ❙⊃ ❙□⇩l(e❙∧f)⌋ ∧
       ⌊((a1 cstit e) ❙∧ (a1 cstit f) ❙∧ (e ❙∧ f))❙⊃(e❙∧f)⌋" unfolding Defs
  by (simp add: ax_refl_a1) 

lemma "⌊ ((a ❙⊃ ❙□⇩l(a1 cstit e)) ❙∧ (a ❙⊃ ❙□⇩l(a1 cstit f)) ❙∧(b ❙⊃ ❙□⇩l(e❙∧f)) ❙∧ (a1 cstit b)) ❙⊃ ❙□⇩l(e❙∧f)⌋ ∧
       ⌊((a1 cstit e) ❙∧ (a1 cstit f) ❙∧ (e ❙∧ f))❙⊃(e❙∧f)⌋" 
  using ax_refl_a1 kand_def kcstit_def kimp_def kvalid_def by auto

(* Moral Luck example *)
consts Drunk::e Drive::e DriveCarefully::e Jump::e Kill::e Hurt::e Stay::e
(* ◇(a1 cstit DriveCarefully) ∈ out2(N,A) *)
lemma "⌊ ((❙⊤ ❙⊃ ❙□⇩l(❙¬Kill ❙∧ ❙¬Hurt)) ❙∧ (❙⊤  ❙⊃ ❙□⇩l(❙□DriveCarefully))
           ❙∧ (❙¬❙◇(a1 cstit DriveCarefully) ❙⊃ ❙□⇩l(a1 cstit Stay)) 
           ❙∧ (Drunk ❙∧ (a1 cstit Drive) ❙∧ (Jump) ❙∧ (Drunk ❙⊃❙¬❙◇(a1 cstit DriveCarefully)
           ❙∧ (❙¬❙◇(a1 cstit DriveCarefully) ❙∧ Jump ❙∧ (a1 cstit Drive)❙⊃(Kill ❙∨ Hurt)))
          )) ❙⊃ ❙□⇩l(❙◇(a1 cstit DriveCarefully))⌋  " 
  by (smt axC1_a1 ax_refl_rbox k45box_def kand_def kbox_def
      kcstit_def kdia_def kimp_def knot_def ktrue_def kvalid_def)
(*(a1 cstit Stay) ∈ out2(N,A) *)
lemma "⌊ ((❙⊤ ❙⊃ ❙□⇩l(❙¬Kill ❙∧ ❙¬Hurt)) ❙∧ (❙⊤  ❙⊃ ❙□⇩l(❙□DriveCarefully))
           ❙∧ (❙¬❙◇(a1 cstit DriveCarefully) ❙⊃ ❙□⇩l(a1 cstit Stay)) 
           ❙∧ (Drunk ❙∧ (a1 cstit Drive) ❙∧ (Jump) ❙∧ (Drunk ❙⊃❙¬❙◇(a1 cstit DriveCarefully)
           ❙∧ (❙¬❙◇(a1 cstit DriveCarefully) ❙∧ Jump ❙∧ (a1 cstit Drive)❙⊃(Kill ❙∨ Hurt)))
         )) ❙⊃ ❙□⇩l(a1 cstit Stay)⌋"
  by (simp add: kand_def kimp_def kvalid_def)
(*(¬Kill ❙∧ ❙¬Hurt) ∈ out2(N,A) *)
lemma "⌊ ((❙⊤ ❙⊃ ❙□⇩l(❙¬Kill ❙∧ ❙¬Hurt)) ❙∧ (❙⊤  ❙⊃ ❙□⇩l(❙□DriveCarefully))
           ❙∧ (❙¬❙◇(a1 cstit DriveCarefully) ❙⊃ ❙□⇩l(a1 cstit Stay)) 
           ❙∧ (Drunk ❙∧ (a1 cstit Drive) ❙∧ (Jump) ❙∧ (Drunk ❙⊃❙¬❙◇(a1 cstit DriveCarefully)
           ❙∧ (❙¬❙◇(a1 cstit DriveCarefully) ❙∧ Jump ❙∧ (a1 cstit Drive)❙⊃(Kill ❙∨ Hurt)))
         )) ❙⊃ ❙□⇩l(❙¬Kill ❙∧ ❙¬Hurt)⌋" 
  by (simp add: kand_def kimp_def ktrue_def kvalid_def)
end
```
