# Supplementary material for: LogiKEy workbench: Deontic logics, logic combinations and expressive ethical and legal reasoning (Isabelle/HOL dataset)
Source: Data Brief. 2020 Oct 15;33:106409. doi: 10.1016/j.dib.2020.106409 (PMC7586073; doi:10.1016/j.dib.2020.106409)
Supplement: Supplementary file 1 [file mmc1.zip › 2020-DataInBrief-Data/E.html]

xml version="1.0" encoding="utf-8"?


Theory E (Isabelle2019: June 2019)


# Theory E

theory E  
imports Main

```
theory E imports Main       (* Aqvist's System E: C. Benzmüller & X. Parent, 2019 *)
begin       
typedecl i (*Possible worlds.*) type_synonym σ = "(i⇒bool)" 
consts aw::i (*Actual world.*)  
abbreviation etrue  :: "σ" ("❙⊤") where "❙⊤ ≡ λw. True" 
abbreviation efalse :: "σ" ("❙⊥")  where "❙⊥ ≡ λw. False"   
abbreviation enot :: "σ⇒σ" ("❙¬_"[52]53)  where "❙¬φ ≡ λw. ¬φ(w)" 
abbreviation eand :: "σ⇒σ⇒σ" (infixr"❙∧"51) where "φ❙∧ψ ≡ λw. φ(w)∧ψ(w)"   
abbreviation eor  :: "σ⇒σ⇒σ" (infixr"❙∨"50) where "φ❙∨ψ ≡ λw. φ(w)∨ψ(w)"   
abbreviation eimp :: "σ⇒σ⇒σ" (infixr"❙→"49) where "φ❙→ψ ≡ λw. φ(w)⟶ψ(w)"  
abbreviation eequ :: "σ⇒σ⇒σ" (infixr"❙↔"48) where "φ❙↔ψ ≡ λw. φ(w)⟷ψ(w)" 

(*Possibilist--constant domain--quantification.*)
abbreviation eforall ("❙∀") where "❙∀Φ ≡ λw.∀x. (Φ x w)"
abbreviation eforallB (binder"❙∀"[8]9) where "❙∀x. φ(x) ≡ ❙∀φ"  
abbreviation eexists ("❙∃") where "❙∃Φ ≡ λw.∃x. (Φ x w)"   
abbreviation eexistsB (binder"❙∃"[8]9) where "❙∃x. φ(x) ≡ ❙∃φ" 

abbreviation ebox :: "σ⇒σ" ("□") where "□ ≡ λφ w.  ∀v. φ(v)"  
consts R :: "i⇒σ" (infixr "R" 70) (*Betterness relation, cf. def. of ○<_|_>.*) 
abbreviation eopt  :: "σ⇒σ" ("opt<_>") 
  where "opt<φ> ≡ (λv. ( (φ)(v) ∧ (∀x. ((φ)(x)  ⟶  v R x) )) )" 
abbreviation esubset :: "σ⇒σ⇒bool" (infix "❙⊆" 53)
  where "φ ❙⊆ ψ ≡ ∀x. φ x ⟶ ψ x"
abbreviation econd  :: "σ⇒σ⇒σ" ("○<_|_>")
  where "○<ψ|φ> ≡  λw. opt<φ> ❙⊆ ψ"
abbreviation euncobl :: "σ⇒σ" ("❙○<_>")   
  where "❙○<φ> ≡ ○<φ|❙⊤>" 

abbreviation evalid :: "σ⇒bool" ("⌊_⌋"[8]109)  (*Global validity.*)
  where "⌊p⌋ ≡ ∀w. p w"
abbreviation ecjactual :: "σ⇒bool" ("⌊_⌋⇩l"[7]105) (*Local validity — in world aw.*)  
  where "⌊p⌋⇩l ≡ p(aw)"

lemma True nitpick [satisfy,user_axioms,expect=genuine] oops (*Consistency conf.*)

(*Correspondence theory.*)
lemma assumes "∀x y z. x R y ∧ y R z ⟶ x R z" 
      shows "⌊((○<ψ|φ>) ❙∧ ❙¬(○<❙¬ξ|φ>)) ❙→ ○<ψ|φ❙∧ξ>⌋" 
  using assms by blast

lemma assumes "⌊((○<ψ|φ>) ❙∧ ❙¬(○<❙¬ξ|φ>)) ❙→ ○<ψ|φ❙∧ξ>⌋" 
      shows "∀x y z. x R y ∧ y R z ⟶ x R z" 
  nitpick [show_all,format=2] oops (*Countermodel presented by Nitpick.*)
end
```
