# Supplementary material for: LogiKEy workbench: Deontic logics, logic combinations and expressive ethical and legal reasoning (Isabelle/HOL dataset)
Source: Data Brief. 2020 Oct 15;33:106409. doi: 10.1016/j.dib.2020.106409 (PMC7586073; doi:10.1016/j.dib.2020.106409)
Supplement: Supplementary file 1 [file mmc1.zip › 2020-DataInBrief-Data/US-Constitution-Loophole/BA_VZahoransky.pdf]

Freie Universität Berlin

Bachelor's Thesis

Modelling the US Constitution in HOL

On establishing a dictatorship with Gödel

Valeria Zahoransky

supervised and examined by
Prof. Dr. Christoph Benzmüller

examined by
Prof. Dr. Jan von Plato
(University of Helsinki)

23rd August 2019

Contents

1 Introduction **1**

2 On the framework used **1**
2.1 The components . . . 1
2.2 Short introduction to Isabelle/HOL . . . 2

3 On the argument used **3**
3.1 Finding Gödel's argument . . . 3
3.2 Argument according to Guerra-Pujol . . . 6

4 Modelling the argument **9**
4.1 Finding a suitable theoretical model . . . 10
4.1.1 On representing dictatorship . . . 10
4.1.2 On representing time . . . 10
4.1.3 On representing Art. V . . . 12
4.2 HOTL - Higher order temporal logic . . . 13
4.2.1 Data types . . . 13
4.2.2 Lifted operators . . . 14
4.2.3 Validity . . . 15
4.3 Simulation . . . 16
4.3.1 Preliminaries . . . 16
4.3.2 Time instance t_1 . . . 19
4.3.3 Time instance t_2 . . . 22
4.3.4 Time instance t_3 . . . 25
4.4 What to avoid when modelling . . . 27
4.4.1 Data type *time* as *int* . . . 27
4.4.2 Functions instead of relations . . . 28
4.4.3 Numerous type declarations . . . 28
4.4.4 Polymorphism . . . 30
4.4.5 Higher order quantification and the Frame Problem . . . 30

5 Outlook **35**

6 Conclusion **36**

7 Declaration of Authorship **37**

8 References **38**

1 Introduction

This thesis aims at modelling parts of the US Constitution with higher order logic (HOL) in theorem prover ISABELLE/HOL in order to verify the possibility of a legal dictatorship in the USA. The basis for the argument is a notorious anecdote on how, at his US citizenship hearing, logician Kurt Gödel informed the judge that the US Constitution was in fact faulty and allowed for the erection of a constitutional dictatorship. We shall explore both the argument Gödel might have had in mind when saying this and a verified version of the supposed argument, modelled on the computer.

Before delving into the argument, we give a short overview on the tools used, including an introduction to Isabelle/HOL and the manner in which we are going to use it.

The ensuing section of this work is concerned with Gödel's supposed argument on the Constitution's shortcomings. This also encompasses a quick overview of the Constitution and a more detailed consideration of the articles most relevant to the argument.

After having laid a theoretical foundation, we will devise and implement a HOL model for the argument in the main part of this work. Being mindful of the technical restrictions, we shall choose a suitable logic embedded into Isabelle's HOL-language and map the relevant parts of the Constitution to their equivalents in the proposed logic. Having succeeded in this, we shall prove that it is possible to build a dictatorship without violating the Constitution, thus verifying Gödel's argument. The main part concludes with a few remarks on what to avoid when modelling a concept with Isabelle/HOL.

The last section will present a few further problematic properties of the US Constitution in addition to the one modelled in the main part. We then name a few questions not yet addressed and conclude the thesis.

For convenience, the terms "US Constitution" and "(the) Constitution" shall be used interchangeably.

2 On the framework used

To start off, we shall give an overview on the framework this thesis was written in, that is, enlist the relevant software components used and explain the core features of Isabelle/HOL since it is the most important component.

2.1 The components

There are three main tools used to write this thesis:

Isabelle/HOL A proof assistant that provides an environment to axiomatize and utilize deduction systems with which one can formulate the-

orems and prove them. It was used to represent the Constitution's model on the computer and conduct reasoning with it.

LaTeX A typesetting software. It is very convenient for mathematical formulas. Isabelle has an inbuilt tool to use LaTeX. This was used to typeset the code written with Isabelle.

Git Version control software to keep track of changes and return to older versions where necessary.

2.2 Short introduction to Isabelle/HOL

Depending on the context, the way Isabelle is used can vary greatly. The following shall illustrate how we will be using it.[1] First we give an outline of the steps taken and then illustrate those with examples.
Here is the outline:

1. Provide definitions of types, concepts, operators used.
2. State all assumptions in the form of axioms.
3. State theorems.
4. Test your theory by trying to refute/prove the theorems. Use tools Sledgehammer and Nitpick to do so.[2]

1. Provide definitions of types, concepts, operators used.
We can define our own data types or work with predefined ones. Here we introduce a data type *bvg* (*beverage*) and a type *temp* (*temperature*). The latter is just a synonym for predefined type *int*.

datatype *bvg* = *tea* | *coffee* | *juice*
type-synonym *temp*=*int*

Next we define predicates and operators.

consts *tempOf*::*bvg*⇒*temp*
— determines temperature of bvg

definition *totalTemp*::*temp*
where *totalTemp* ≡ (*tempOf tea*) + (*tempOf coffee*) + (*tempOf juice*)
— determines total temperature of all bvg-instances

definition *tooHot*::*bvg*⇒*bool*
where *tooHot b* ≡ *if*(*b*=*juice*)*then*(*tempOf b* > *5*) *else* (*tempOf b* > *20*)

[1]For a general introduction see the manual [Wen19]
[2]See also the manuals on Sledgehammer [BP19] and on Nitpick [Bla19]

— determines if a beverage is too hot. For *juice* this is the case iff its temperature is >*5*, for *tea* and *coffee* iff the temperature is >*20*

2. State all assumptions in the form of axioms.
Note that we introduce a contradiction with the first two axioms *teaHot tea* and *tempOf tea* = *10*.

axiomatization where
teaHot: *tooHot tea* **and**
teaTemp10: (*tempOf tea*)=*10* **and**
coffeeTemp5:(*tempOf coffee*)=*5* **and**
juiceTemp2: (*tempOf juice*)=*2*

3. State theorems and 4. Test your theory by trying to refute/prove the theorems.
These steps have to be conducted together since Isabelle requires theorems to be proven. It is not possible to enumerate theorems without proofs unless you use keyword **oops** to signify that a theorem has not been proven yet.

Sledgehammer can be used to find proofs and *Nitpick* to find counter models and satisfying models.

theorem *totalTemp17*:*totalTemp* = *17* **sledgehammer**
by (*simp add*: *coffeeTemp5 juiceTemp2 teaTemp10 totalTemp-def*)

lemma *basic-unsat*:*False* **using** *teaHot teaTemp10 tooHot-def* **sledgehammer**
by *simp*

lemma *basic-sat*:*True* **nitpick**[*show-all*,*user-axioms*] **oops**

Note that our axioms are inconsistent, so we can prove *basic-unsat*. Nitpick can neither find a counter model nor a satisfying one. However, if we remove axiom *teaHot* or *teaTemp10* lemma *basic-unsat* becomes unprovable and Nitpick will find a satisfying model for lemma *basic-sat*. To look for a satisfying model rather than a refuting one, we simply add option *satifsy*.

3 On the argument used

3.1 Finding Gödel's argument

Following his permanent employment at the *Institute for Advanced Study* (IAS) in Princeton, Gödel applied for US citizenship in 1947.[3]

As part of his naturalization process he had to attend a hearing during which a judge would ask questions on topics such as the governmental system or the history of the United States. Gödel was accompanied by two fellow scientists at the IAS: economist Oskar Morgenstern and physicist Albert Einstein. The two served as character witnesses.

[3] See [Daw97], p.159 a. p.179f

There are different accounts of this event. Biographers Dawson, Wang and Yourgrau all present the anecdote, albeit somewhat differently.[4] Dawson refers to an interview with Morgenstern's wife and a diary entry, but also mentions that he hoped to refer to an account by Morgenstern himself but that he couldn't locate it.[5] Wang refers to an obituary by Zemanek[6] and Yourgrau refers to Dawson[7].

Gödel himself mentions the hearing in letters to his mother but doesn't go into much detail.[8]

Morgenstern's account has since been published by the IAS. The following will recount the incident according to Morgenstern since, unlike Dawson, Morgenstern attended the hearing himself.[9]

Being a very thorough person, Gödel prepared for his citizenship hearing months in advance. He studied US history and law from the first settlers and Native American tribes to the exact border between Princeton Borough and Princeton Township to the US Constitution. Apparently Gödel would address several of his questions to Morgenstern and the two of them would discuss these matters together.

Morgenstern also mentions conversations about these topics in his diaries from 1947, unfortunately without going into much detail.[10] For example, in his entry from February 26 he says he would be with the Gödels the following day and that most certainly Gödel would have his notebook and a lot of questions waiting. The next diary entry from March 3 tells us that Morgenstern had in fact been with the Gödels twice but only mentions conversations about other topics, nothing about the pending hearing.

Eventually Gödel seemed to have found a fault with the Constitution, a fault that would allow for the erection of a fascist regime. He was most distressed and could not be calmed by either Morgenstern or Einstein. They told him that questions asked would not require an in-depth analysis of the Constitution and tried to dissuade him from mentioning the matter altogether.

At the hearing the judge first asked Einstein and Morgenstern whether they considered Gödel to be a good potential citizen. Being his character witnesses they confirmed. The judge then turned to Gödel and the following

[4]See [Daw97],p.179f and [Wan87], p.115f and [You06], p.98f

[5]S. [Daw97], p.300

[6]S. [Wan87], p.115

[7]S. [You06], p.190

[8]S. [Göd78], *Dec 9 1947*: Gödel mentions that he will soon be US citizen. *Jan 11 1948*: Gödel mentions the hearing and explains shortly who was there and why. *16 Mar 1948*: Gödel mentions that he hasn't gotten a response concerning his application yet. *May 10 1948*: Gödel describes his citizenship oath. This is more detailed than the information on the preceding hearing.

[9]The following paragraphs on the hearing all refer to [Mor71] unless clearly stated otherwise

[10]S. [Mor16], *Feb 26 1947*, *Mar 3 1947* and *Dec 7 1947*

dialogue unfolded:[11]

> The Examinor : "Now, Mr. Godel, where do you come from?"
>
> Gödel : "Where I come from? Austria."
>
> The Examinor : "What kind of government do you have in Austria?"
>
> Gödel : "It was a republic, but the constitution was such that it finally was changed into a dictatorship."
>
> The Examinor : "Oh! This is very bad. This could not happen in this country."
>
> Gödel : "Oh, yes, I can prove it."
> [. . .]
>
> [The Examinor :]"Oh God, let's not go into this."

Evidently, the examining judge was not interested in hearing Gödel's reasoning behind such a statement. What was probably good for Gödel's successful naturalization is rather inconvenient for us since it leaves us without a record of Gödel's argument.

As mentioned above, while Morgenstern does write about conversations with Gödel in his diaries of 1947, the topics he mentions do not expand to Gödel's reservations about the Constitution.
Furthermore none of the biographers seem to have found a record of the argument. We are thus forced to speculate on what it might have been.

We shall use an argument provided by legal scholar Enrique Guerra-Pujol as basis for our further reasoning. In his article "Gödel's Loophole" he names a few problems with the US Constitution and goes into detail on one of them that is concerned with self-referentiality.[12]

Considering that self-referentiality was at the very heart of the proof for the Incompleteness Theorem[13] Guerra-Pujol's argument shares at least one feature with Gödel's work even if it is not what Gödel himself had in mind.

Also, as will be shown below, the argument requires very unlikely conditions to be fulfilled which might be what Morgenstern is referring to when he writes

> I told him that it was most unlikely that such events would ever occur, even assuming that he was right[...].[14]

With this in mind, we choose to work with Guerra-Pujol's argument. Having decided on a basis for the Consitution's model, we shall now take a closer look at that argument.

[11] This direct quote from [Mor71] contains some misspellings. For better readability they are listed here: *Godel-Gödel,examinor-examiner*

[12] S. [GP13]

[13] S. [Smu92], chapter "I The General Idea Behind Gödel's Proof"

[14] S. [Mor71]

3.2 Argument according to Guerra-Pujol

To understand Guerra-Pujol's reasoning, let us first consider the US Constitution and its structure.

The Constitution is made up of seven original articles written in 1787 and 27 amendments that followed later. Note that at the time of Gödel's hearing in 1947 there were only 21 amendments with the twenty-second having been proposed but not yet ratified.[15] Here is a broad overview on the original articles' contents:

Article	Content
I	Legislative
II	Executive
III	Judiciary
IV	States' relations
V	Amendments
VI	Prior Debts and National Supremacy
VII	Ratification of the Constitution

Articles I-III specifiy the rights of the governmental branches, by which institutions they are represented, how elections are to be held and so forth.
Article IV sets up the federalistic system by which each state has legislative sovereignty over its own affairs, but also manages interstate relations. In this context, state is to mean a member of the United States of America.
Article V describes the process of changing or amending the Constitution. This article is of particular importance to the argument. It will therefore be considered in more detail later.
Article VI determines that any state's debts are not changed by the ratification of the Constitution. Furthermore, it states that the Constitution shall be the "supreme Law of the Land" and any official representative has to swear an oath of support.
Article VII finally stipulates that the Constitution will be ratified through the ratification of nine states. The ratification by all thirteen original states is not necessary for the Constitution to take effect.

Here is the outline of the actual argument:
As it is, the Constitution does not allow for a dictatorship under one dictator since there is a division of powers into legislative, executive and judiciary, all of which have unique rights and responsibilities.

This means that in order to set up a dictatorship the Constitution needs to be amended first. How can this be done? To answer this question one

[15] S. [Bal17] for a list of all articles and amendments, as well as notes on which articles were affected by which amendments.

needs to consider Article V:[16]

(1) The Congress, whenever two thirds of both Houses shall deem it necessary, shall propose Amendments to this Constitution,

(2) or, on the Application of the Legislatures of two thirds of the several States, shall call a Convention for proposing Amendments,

(3) which, in either Case, shall be valid to all Intents and Purposes, as Part of this Constitution, when ratified by the Legislatures of three fourths of the several States,

(4) or by Conventions in three fourths thereof, as the one or the other Mode of Ratification may be proposed by the Congress;

(5) Provided that no Amendment which may be made prior to the Year One thousand eight hundred and eight shall in any Manner affect the first and fourth Clauses in the Ninth Section of the first Article;

(6) and that no State, without its Consent, shall be deprived of its equal Suffrage in the Senate.

There are three concepts addressed in this article:

- The **proposition** of amendments (paragraphs 1 and 2)
- The **ratification** of amendments (paragraphs 3 and 4)
- **Entrenchments** of other parts of the Constitution (paragraphs 5 and 6)

An amendment may be proposed either by Congress or by a specifically held Convention. In the first case at least two thirds of both houses of the Congress, i.e. the House of Representatives and the Senate, need to support the proposition. In the second case, at least two thirds of all states' legislatures need to request such a Convention.

As to the ratification, the conditions for ratification are similar to but not quite the same as for proposition. There are two possible methods and Congress decides which method shall be used. Either a proposed amendment is ratified by at least three fourths of all states' legislatures or special State Conventions are held for each state, three fourths of which need to ratify the amendment.

With regard to "entrenchments", let us first clarify what is meant by the term. There are different definitions of what "entrenchment" of a rule means. In the broadest sense an entrenched rule is "any rule that is difficult

[16] This is a literal quote, including dated spelling. The arrangement in separate paragraphs was added for better readability. The original is one paragraph.

to alter".[17] Therefore an "entrenchment rule" is a rule causing some rule to be entrenched. Art. V is thus an entrenchment both of Art. I, §9, cl.1, cl.4 and of the article regulating states' votes in Senate, namely Art. I, §3, cl.1.[18]

The first two clauses regulate slavery[19] and how taxes are raised[20] but shall not concern us since they were only entrenched up until 1808. We are interested in the Constitution Gödel was working with and that is the Constitution from 1947. Hence the entrenchments concerning these clauses were not valid anymore.

Art. I, §3, cl.1 was amended by Amend. XXVII[21] , ratified in 1913, which is thus relevant for us as well. The articles determine that each State shall have two representatives in Senate, each having one vote. So, according to Art. V, an amendment may not change either of these clauses.

In summary, Art V. gives instructions on how to propose and ratify an amendment with the additional condition of an amendment not infringing on a state's votes in Senate.

This means that Art. V poses an obstacle on the path to legal dictatorship via amendments. Luckily, Art. V does not protect itself from amendment.
One could thus institute a dictatorship by following these steps:

1. Propose an amendment to remove the entrenchment clause from Art. V.

2. Ratify this amendment.

3. Propose an amendment to institute a dictatorship, e.g. by depriving Congress and all courts of their rights and granting those rights to the President.

4. Ratify this amendment and behold the marvellous institution that is presidential dictatorship created at your hands.

---

[17] S. [Bar16], p.327

[18] U.S. Const. art.I, §3., cl.1.: "The Senate of the United States shall be composed of two Senators from each State, chosen by the Legislature thereof, for six Years; and each Senator shall have one Vote."

[19] U.S. Const. art.I, §9., cl.1.: "The Migration or Importation of such Persons as any of the States now existing shall think proper to admit, shall not be prohibited by the Congress prior to the Year one thousand eight hundred and eight, but a Tax or duty may be imposed on such Importation, not exceeding ten dollars for each Person."

[20] U.S. Const. art.I, §9., cl.4.: "No Capitation, or other direct, Tax shall be laid, unless in Proportion to the Census or Enumeration herein before directed to be taken."

[21] U.S. Const. amend.XXVII, §1.: "The Senate of the United States shall be composed of two Senators from each State, elected by the people thereof, for six years; and each Senator shall have one vote. The electors in each State shall have the qualifications requisite for electors of the most numerous branch of the State legislatures"

Now, while this sounds rather simple, there are a few remarks that should be made at this point.

Firstly, both proposition and ratification require large majorities in Congress and states' legislatures. It his highly unlikely that any state legislature would ratify an amendment depriving Art. V of its entrenchment clause. After all, it protects all states' suffrage in Senate. So, while this is, in theory, a feasible method of setting up a dictatorship, it would most likely not come to pass. This is the improbable condition mentioned above in 3.1, which Morgenstern may have been referring to in his account of the hearing.

Secondly, if we assume that a majority of Congress and state legislatures do support the anti-entrenchment amendment, then the amendment is actually unnecessary. This is because Art. V only prohibits the decrease of a state's suffrage when said state does not give its consent. Assuming that all states support the anti-entrenchment amendment, they would probably also support an amendment that attacks their suffrage directly.

Having made these remarks, we do choose to work with the argument presented above. Albeit very unlikely, it provides a possible path to constitutional dictatorship. Also it makes use of the fact that Art. V. does not entrench itself. So, even if this was not Gödel's own argument, it will at least share one characteristic with another work of Gödel's, the Incompleteness Theorem, which has self-referentiality at its heart.[22]

4 Modelling the argument

This section comprises the actual modelling and simulation of the theoretical argument presented above.

We shall first look at how to best map the relevant concepts to higher order logic, i.e. answer questions as to which kind of logic(s) to use, how to represent dictatorship and non-dictatorship, which axioms to use and so forth.

After having answered these questions, we will simulate the institution of dictatorship starting with a rough model of the Constitution as set out in 1947 and transitioning to a model that allows for dictatorship without creating inconsistencies along the way. This will be done in HOTL and Simulation. HOTL (higher order temporal logic) contains basic definitions of the logical framework used. Simulation holds the actual content on the Constitution.

The section closes with a few remarks on what to avoid when modelling a concept in Isabelle/HOL. Throughout the process of this work many ideas had to be discarded and providing some insight on the difficulties involved, especially when it comes to weaknesses of Isabelle, might help others with similar tasks.

[22] S. [Smu92], chapter "I The General Idea Behind Gödel's Proof"

4.1 Finding a suitable theoretical model

4.1.1 On representing dictatorship

As mentioned above, once Art. V has been adequately changed, we want to introduce an amendment that implements a dictatorship. In order to do this, it is necessary to first determine what is meant by dictatorship.

Depending on the angle you consider this question from, the answer may turn out quite differently. In this context we are not interested in the negative connotation of despotism that comes with the term "dictatorship" but rather in the technical question of how a government has to be structured in order to be called a dictatorship.

According to Levinson and Balkin in a dictatorship the dictator "combines elements of judicial, legislative, and executive power" with dictators being individuals or institutions.[23]

Note that this definition does not require the dictator to consolidate all power but only some in each branch. There could, in fact, be several dictators of different types. The authors call this "special-purpose dictatorships" and name dictatorship with respect to war as one such type where the dictator might have the "power to initiate war, commandeer funds and resources for war, and conduct war at any time for any reason in any manner he pleases".[24]

For convenience, dictatorship in our case shall be simplified to be an "all-purpose dictatorship" where a dictator is required to have all judicial, legislative, and executive power. If a person or institution does not combine all of them, they are not a dictator.

Note that we have only considered a horizontal distribution of power. Being a federal union of states, the USA also implements a vertical distribution of power. The fact that at least 75% of state legislatures have to support ratification for it to be successful is an example of this.[25]

Since federalism shall not play a big part in our model, we choose to distinguish between horizontal and vertical distribution of power and define dictatorship to be the consolidation of all power on a national level, disregarding any lower levels such as states, counties or cities.

4.1.2 On representing time

Since the basic idea of the argument is to introduce amendments to change the Constitution, we have to be able to express the notion of change.

We choose to do this via temporal logic and more specifically with an instant-based model of time as opposed to an interval-based one. That is, we introduce different points in time and an operator to connect those. Since

[23] S. [LB09], p.1805
[24] S. [LB09], p.1806
[25] S. 3.2

we are not interested in the concept of duration, the discrete approach is enough.

Now, generally, this kind of logic would be expressed by a set T of instances of time and a precedence relation $\prec$ on $T \times T$, such that $\prec$ is both irreflexive and transitive.[26]

We shall not require a relation to be transitive, however. Neither will we use modal operators to express that certain events will *always* occur in the future or that an event will occur *at some point* in the future. The same goes for events in the past. We only require an operator **X** that refers to the immediate successor of an instance of time. The operator is denoted by **X** for the "x" in "next".

To understand why this is sensible in our case, consider the following outline of what we would like to express. Assume that $T = \{t_1, t_2, t_3\}$ and $t_1 \prec t_2$,$t_2 \prec t_3$ and $t_i \not\prec t_j$ for all other combinations of t_i and t_j in T :

t_1:
- The Constitution from 1947 is valid.
- There is a division of powers and thus no dictatorship.
- An amendment to change Art. V (*amd1*) is proposed, but not yet ratified.
  Content of *amd1*: Remove the condition that only amendments can be proposed that do not alter a state's suffrage.

t_2:
- *amd1* is ratified and therefore valid.
- The Constitution from 1947 is valid, except for Art. V.
- There is a division of powers and thus no dictatorship.
- Art. V does not require proposed amendments to leave states' voting rights untouched.
- An amendment to introduce dictatorship (*amd2*) is proposed, but not yet ratified.
  Content of *amd2*: Give all rights of Congress and the Courts to the President.

t_3:
- *amd2* is ratified and therefore valid.
- All power is with the President. With the abolished division of powers there is a dictatorship.

The basis for changes in t_2 is set out with *amd1* at t_1. Likewise the basis for changes in t_3 is set out with *amd2* at t_2. At each $t_i \in T$ the furthest we look into the future is the immediate successor, thus we do not need $\prec$ to be transitive.

In addition to it not being necessary, there is another reason to omit transitivity as requirement for the precedence relation. For a formula φ, we

[26] S. [GG15], "2.1 Instant-based models of the flow of time"

would like $\mathbf{X}\varphi$ to be valid at some point of time t iff for any t', s.t. $t \prec t'$, holds: φ is valid at t'.
If $\prec$ were transitive, then $\mathbf{X}\varphi$ would not mean "φ is valid at the next instance after t", but "φ is valid at all instances after t". If not used very carefully, this could easily lead to inconsistencies. After all, amendments do not necessarily stay valid once ratified.[27] Since we do not need a transitive relation $\prec$, it is advisable to avoid it altogether.

4.1.3 On representing Art. V

The concepts actually mapped onto HOL are only a fraction of what is written in the Constitution. This is because representing everything would go beyond the scope of this work. Since Art. V is of particular importance to the argument, it will not be omitted but we shall concentrate on the relevant bits.
Recall that the three concepts addressed are:

1. **proposition of amendments**
   with support of
   - 1.1. two thirds of both houses of Congress
   - 1.2. two thirds of State Legislatures requesting a Convention
2. **ratification of amendments**
   with support of
   - 2.1. three fourths of State Legislatures
   - 2.2. three fourths of State Conventions
3. **entrenchment**
   protecting
   - 3.1. until 1808: Art. I, §9, cl.1 3 , cl.4
   - 3.2. Art. I,§3, cl.1, Amend. XXVII, cl.1

We shall not represent 1.2.,2.1.,2.2., or 3.1. for the following reasons:

- 1.2.,2.1.,2.2. are part of the federal system which is not essential to the argument.
- 3.1. may be ignored since 1808 had long since passed when Gödel studied the Constitution.

The remaining points will be represented as follows:

[27] S. [Bal17]: Amend.XVII, the prohibition of intoxicating liquors, was repealed by Amend. XXI, §.1

- For 1. and 1.1. there will be a predicate *is-prop* for potential amendments that will only be true if the amendments have the support of Congress to be proposed.

- In analogy to *is-prop*, there will by a predicate *is-rat* that can only be true if the amendments have support to be ratified. What this support looks like shall not be specified further. Predicate *is-rat* will serve to express 2.

- To express 3. and 3.2. there will be a predicate *maint-suf* for amendments which shall be true iff the amendments would maintain equal suffrage in Senate for each state.

4.2 HOTL - Higher order temporal logic

This section introduces the logical operators and data types we will be working with in Simulation.

4.2.1 Data types

There are two new data types g *time* and one derived data type σ.

Type g is for governmental institutions, with *Congress* being Congress, P being the President and *Courts* being the Supreme Court as well as other courts set up by Congress. The legislative, executive and judicial powers shall later be bestowed upon these three instances of g. We use *Courts* rather than just the Supreme Court since Art. III, §1. states that the "judicial Power [...] shall be vested in one supreme Court, and in such inferior Courts as the Congress may [...] ordain and establish."

datatype $g = \mathit{Congress} \mid P \mid \mathit{Courts}$

There are four instances of time: t_1-t_3 as in 4.1.2 and t_e, the instance that marks the end of time. At t_1 the 1947 version of the Constitution is valid. t_2 holds the version with an amended Art. V that allows for amendments that do not maintain states' suffrage and t_3 with the Constitution upholding dictatorship.

Note that there is a fourth instance t_e. We need this for technical reasons. Since we want to use an operator **X** that carries a formula from one instance to its successor, it is convenient to have a successor for each instance of time used. Unless we define a circular successor relation we need a further instance of time that can be the successor of t_3 to avoid inconsistencies. We shall point out where t_e prevents inconsistencies when it becomes relevant below.

datatype $\mathit{time} = t_1 \mid t_2 \mid t_3 \mid t_e$

Since we will only consider a formula's validity at a certain point in time we need a time dependant type for them, as well as operators lifted to that type.

See the following definition of a time dependant formula's type. We will use it to explain what a lifted operator is and as a basis for our lifted operators.

type-synonym $\sigma{=}(time{\Rightarrow}bool)$

Assume you have operator

$$op{::}'a{\Rightarrow}bool$$

where $'a$ is an arbitrary type and *bool* is Isabelle's version of the boolean type. A lifted version

$$op_l{::}'a{\Rightarrow}\sigma$$

of *op* would be an operator such that for any argument $arg{::}'a$

$$op_l\ arg \equiv \Phi(op)(arg)$$

with $\Phi{::}('a{\Rightarrow}bool){\Rightarrow}'a{\Rightarrow}\sigma$ being a suitable function to translate the notion of what *op* does to a notion of what it does at a particular instance of time. What this function Φ looks like depends on *op*. See below how it is done for the operators we require.

4.2.2 Lifted operators

The following are lifted versions for standard logical operators $\{\neg,\wedge,\vee,\longrightarrow.\longleftrightarrow\}$, as well as for $\{=,!=\}$ and for quantifiers $\{\forall,\exists\}$.

Observe that the quantifiers lifted may each only be used for one type of argument. We shall go into detail about polymorphism in 4.4.4.

Note also that they need an additional binding for the form we are used to. This is because the initial definition actually refers to operator $\Pi_{(\alpha\Rightarrow bool)\Rightarrow bool}$ which allows us to define a lifted $\forall$ using only lambda abstraction.[28]

definition *tneg* :: $\sigma{\Rightarrow}\sigma$ ($\neg$-[52]53) **where** $\neg\varphi \equiv \lambda t.\ \neg\varphi(t)$
definition *tand* :: $\sigma{\Rightarrow}\sigma{\Rightarrow}\sigma$ (**infixr**$\wedge$51) **where** $\varphi{\wedge}\psi \equiv \lambda t.\ \varphi(t){\wedge}\psi(t)$
definition *tor* :: $\sigma{\Rightarrow}\sigma{\Rightarrow}\sigma$ (**infixr**$\vee$50) **where** $\varphi{\vee}\psi \equiv \lambda t.\ \varphi(t){\vee}\psi(t)$
definition *timp* :: $\sigma{\Rightarrow}\sigma{\Rightarrow}\sigma$ (**infixr**$\longrightarrow$49) **where** $\varphi{\longrightarrow}\ \psi \equiv \lambda t.\ \varphi(t){\longrightarrow}\psi(t)$
definition *tequ* :: $\sigma{\Rightarrow}\sigma{\Rightarrow}\sigma$ (**infixr**$\longleftrightarrow$48) **where** $\varphi{\longleftrightarrow}\psi \equiv \lambda t.\ \varphi(t){\longleftrightarrow}\psi(t)$

definition *teq* :: $g{\Rightarrow}g{\Rightarrow}\sigma$ (**infixr=**40) **where** $\varphi{=}\psi \equiv \lambda t.\ \varphi{=}\psi$
definition *tneq* :: $g{\Rightarrow}g{\Rightarrow}\sigma$ (**infixr!=**40) **where** $\varphi\mathbf{!}{=}\psi \equiv \lambda t.\neg(\varphi{=}\psi)$

definition *tall-g* :: $(g{\Rightarrow}\sigma){\Rightarrow}\sigma$ ($\forall_{\boldsymbol{g}}$) **where** $\forall_{\boldsymbol{g}}\Phi \equiv \lambda t.\forall x.\ \Phi(x)(t)$
definition *tallB-g*:: $(g{\Rightarrow}\sigma){\Rightarrow}\sigma$ (**binder**$\forall_{\boldsymbol{g}}$[8]9) **where** $\forall_{\boldsymbol{g}}x.\ \varphi(x) \equiv \forall_{\boldsymbol{g}}\varphi$

[28] S. [BA19],"1.1 Fundamental Ideas"

definition *texi-g* :: $(g{\Rightarrow}\sigma){\Rightarrow}\sigma$ $(\exists_{\boldsymbol{g}})$ **where** $\exists_{\boldsymbol{g}}\Phi \equiv \lambda t.\exists x.\ \Phi(x)(t)$
definition *texiB-g*:: $(g{\Rightarrow}\sigma){\Rightarrow}\sigma$ (**binder**$\exists_{\boldsymbol{g}}$[*8*]*9*) **where** $\exists_{\boldsymbol{g}}x.\ \varphi(x) \equiv \exists_{\boldsymbol{g}}\varphi$

definition *tall-s* :: $(\sigma{\Rightarrow}\sigma){\Rightarrow}\sigma$ $(\forall_{\boldsymbol{\sigma}})$ **where** $\forall_{\boldsymbol{\sigma}}\Phi \equiv \lambda t.\forall \varphi.\ \Phi(\varphi)(t)$
definition *tallB-s*:: $(\sigma{\Rightarrow}\sigma){\Rightarrow}\sigma$ (**binder**$\forall_{\boldsymbol{\sigma}}$[*8*]*9*) **where** $\forall_{\boldsymbol{\sigma}}\varphi.\ \Phi(\varphi) \equiv \forall_{\boldsymbol{\sigma}}\Phi$

definition *texi-s* :: $(\sigma{\Rightarrow}\sigma){\Rightarrow}\sigma$ $(\exists_{\boldsymbol{\sigma}})$ **where** $\exists_{\boldsymbol{\sigma}}\Phi \equiv \lambda t.\ \exists \varphi.\ \Phi(\varphi)(t)$
definition *texiB-s*:: $(\sigma{\Rightarrow}\sigma){\Rightarrow}\sigma$ (**binder**$\exists_{\boldsymbol{\sigma}}$[*8*]*9*) **where** $\exists_{\boldsymbol{\sigma}}\varphi.\ \Phi(\varphi) \equiv \exists_{\boldsymbol{\sigma}}\Phi$

The last operator we want to introduce is **X**. This requires a precedence relation. To stress the fact that we are talking about a *future* instance of time when using **X** we call the relation *succ* for successor, rather than *pred* for predecessor.

consts *succ*::*time*⇒*time*⇒*bool*
axiomatization where
t1-s-t2: *succ* t_1 t_2 **and**
t2-s-t3: *succ* t_2 t_3 **and**
t3-s-te: *succ* t_3 t_e **and**
te-s-te: *succ* t_e t_e **and**
Nt1-s-t1: $\neg(succ\ t_1\ t_1)$ **and**
Nt1-s-t3: $\neg(succ\ t_1\ t_3)$ **and**
Nt1-s-te: $\neg(succ\ t_1\ t_e)$ **and**
Nt2-s-t1: $\neg(succ\ t_2\ t_1)$ **and**
Nt2-s-t2: $\neg(succ\ t_2\ t_2)$ **and**
Nt2-s-te: $\neg(succ\ t_2\ t_e)$ **and**
Nt3-s-t1: $\neg(succ\ t_3\ t_1)$ **and**
Nt3-s-t2: $\neg(succ\ t_3\ t_2)$ **and**
Nt3-s-t3: $\neg(succ\ t_3\ t_3)$ **and**
Nte-s-t1: $\neg(succ\ t_e\ t_1)$ **and**
Nte-s-t2: $\neg(succ\ t_e\ t_2)$ **and**
Nte-s-t3: $\neg(succ\ t_e\ t_3)$

So in Kripke semantics[29] a visualisation of the instances with *succ* as accessibility relation would look as follows:

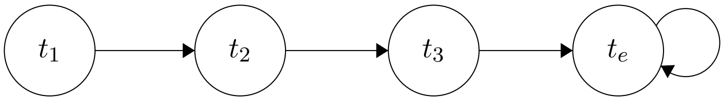

Based on *succ* we can then define **X**.

definition *tnext* :: $\sigma{\Rightarrow}\sigma$ (**X**-) **where** $\mathbf{X}\varphi \equiv (\lambda t.\ \forall t'.\ ((succ\ t\ t') \longrightarrow \varphi\ t'))$

4.2.3 Validity

Lastly, we want to define a notion of *validity*. We distinguish between global and local validity.

[29] S. [Gar18]

A formula shall be globally valid when it is valid independently of the the current time. This is useful for universally valid definitions such as what we mean by *dictatorship*. A formula shall be locally valid for a specific t if it is valid at that instance of time.

definition *global-valid* :: $\sigma \Rightarrow$ *bool* ($\lfloor$-$\rfloor$[7]8) **where** $\lfloor\varphi\rfloor \equiv \forall\, t.\ \varphi\ t$
definition *local-valid* :: $\sigma\Rightarrow$*time* $\Rightarrow$ *bool* ($\lfloor$-$\rfloor$-[9]10) **where** $\lfloor\varphi\rfloor_t \equiv \varphi\ t$

We conclude this section with checking satisfiability and enlisting all definitions in *Defs* so we may access them conveniently in proofs later on.

Lemmas used to test the modelling begin with a T to signify that they are testing lemmas. The check for satisfiability is one such testing lemma.

lemma *T-basic-sat-HOTL*: *True* **nitpick**[*satisfy*,*user-axioms*,*show-all*]**oops**

named-theorems *Defs* **declare**
tneg-def [*Defs*] *tand-def* [*Defs*]
tor-def [*Defs*] *timp-def* [*Defs*] *tequ-def* [*Defs*]
teq-def [*Defs*] *tneq-def* [*Defs*]
tall-g-def [*Defs*] *tallB-g-def* [*Defs*]
texi-g-def [*Defs*] *texiB-g-def* [*Defs*]
tall-s-def [*Defs*] *tallB-s-def* [*Defs*]
texi-s-def [*Defs*] *texiB-s-def* [*Defs*]
tnext-def [*Defs*]
global-valid-def [*Defs*] *local-valid-def* [*Defs*]

4.3 Simulation

This section is made up of four parts. In 4.3.1 basic notions are defined that will be used throughout the remainder of the section. Part 4.3.2 gives an axiomatization of what is valid at t_1 and some proofs on the basis of those axioms. The same holds for 4.3.3 and 4.3.4, only they describe the state at t_2 and t_3 respectively.

4.3.1 Preliminaries

We begin with definitions for governmental institutions g. They express that g is a certain branch of government.

This is a very simplified version of the Constitution, stripped off anything not relevant to the argument. For instance, rather than saying that some g has executive powers and is thus entitled to command the army, grant and reprieve pardons[30], we simply state that g *is* the executive.

consts
is-leg::$g\Rightarrow\sigma$ — g is the legislative
is-exe::$g\Rightarrow\sigma$ — g is the executive

[30] S. U.S. Const. art.2, §2.

$is\text{-}jud{::}g{\Rightarrow}\sigma$ — g is the judiciary

We require the branches to be unique, i.e. each branch has to have a unique governmental institution associated with it.

One could imagine a distribution of one branch over several governmental institutions. In fact, governmental institution *Courts* represents a collection of courts and thus several different governmental institutions. To Isabelle, however, it is a single instance of type g. Only we know that *Courts* isn't just one institution.

We choose to demand uniqueness because it keeps the model simple without taking away any concepts necessary to the argument. If we didn't demand uniqueness, we would have to explicitly state which institutions represent which branches *and* which they do not represent. Otherwise, the fact that for example *Congress* is legislative would not imply that P isn't. So P could be both legislative and executive. To then prove non-dictatorship for t_1 and t_2 would be impossible since any institution could be all of the branches.

Given that we do not need to model an institution representing several branches we may as well simplify things and demand uniqueness.

axiomatization where
$unique\text{-}is\text{-}leg$: $\lfloor\forall_{\boldsymbol{g}} g1.\ \forall_{\boldsymbol{g}} g2.\ (((is\text{-}leg\ g1)\wedge(is\text{-}leg\ g2))\longrightarrow(g1 = g2))\rfloor$ **and**
$unique\text{-}is\text{-}exe$: $\lfloor\forall_{\boldsymbol{g}} g1.\ \forall_{\boldsymbol{g}} g2.\ (((is\text{-}exe\ g1)\wedge(is\text{-}exe\ g2))\longrightarrow(g1 = g2))\rfloor$ **and**
$unique\text{-}is\text{-}jud$: $\lfloor\forall_{\boldsymbol{g}} g1.\ \forall_{\boldsymbol{g}} g2.\ (((is\text{-}jud\ g1)\wedge(is\text{-}jud\ g2))\longrightarrow(g1 = g2))\rfloor$

There is a dictatorship at t if at that instance of time a dictator d exists that represents all branches of government.

definition $Dictatorship{::}\sigma$
where $Dictatorship \equiv \lambda t.\ \exists\, d.\ \lfloor(is\text{-}leg\ d) \wedge (is\text{-}exe\ d) \wedge (is\text{-}jud\ d)\rfloor_t$

Below follow some predicates for formulas $\varphi{::}\sigma$. Based on these we also define predicates that are only dependant on time, and thus are either valid or not valid for a certain instance of time. These will serve as properties of the Constitution at different points in time.

consts
$is\text{-}amd{::}\sigma{\Rightarrow}\sigma$ — φ is an amendment
$is\text{-}prop{::}\sigma{\Rightarrow}\sigma$ — φ is proposed
$is\text{-}rat{::}\sigma{\Rightarrow}\sigma$ — φ is ratified
$sup\text{-}prop{::}g{\Rightarrow}\sigma{\Rightarrow}\sigma$ — φ has support by g to be proposed
$sup\text{-}rat{::}\sigma{\Rightarrow}\sigma$ — φ has support to be ratified
$maint\text{-}suf{::}\sigma{\Rightarrow}\sigma$ — φ maintains suffrage in Senate for all states

We shall now define the following concepts:

oap Only amendments may be proposed.
This time dependant formula is used for technical reasons. It helps to distinguish between generic formulas φ of type σ and what we call amendment. For example *oap* itself may not be proposed if it isn't also declared an amendment.

osp Only if an amendment has the support of the legislative, can it be proposed.
This is a simplified version of what Art. V says. Basically *osp* requires an amendment to have support by two thirds of both houses of Congress. As mentioned above we omit the option of support by a specific convention, so we can concentrate solely on horizontal division of power.
Another reason why this simplified version is preferable is because it is more generic and allows for a change of interpretation. That is, we can make a statement about the legislative supporting an amendment, no matter if the current constitution stipulates Congress to be the legislative or not.

omsp Only amendments that maintain suffrage may be proposed.

opr Only proposed amendments may be ratified at the next time instance.

osr Only if an amendment has the support for ratification, can it be ratified in the future.

psr If an amendment is proposed and has the support for ratification, it will be ratified at the next time instance.
This will be used to show that an amendment proposed at t_i is ratified and thus valid at t_{i+1}, given that it also has support for ratification at t_i.
Note that together with *opr* this makes proposition and ratification of an amendment a two-step process.

rv If an amendment is ratified, it is also valid.
Here the framework for reasoning about amendments is entwined with the the content of the amendments. In combination with *psr* this property is a precarious one to work with for, as soon as *rv* is declared to be valid for some *t*, it will be possible to prove anything as long as it has been proposed with support for ratification in the preceding instance of time.

abbreviation $oap::\sigma$
where $oap \equiv \forall_{\boldsymbol{\sigma}}\varphi.\ (\neg(\textit{is-amd}\ \varphi))\longrightarrow(\neg(\textit{is-prop}\ \varphi))$

abbreviation $osp::\sigma$
where $osp \equiv \forall_{\boldsymbol{\sigma}}\varphi.\ \forall_{\boldsymbol{g}}g.(\textit{is-leg}\ g)\longrightarrow((\neg(\textit{sup-prop}\ g\ \varphi))\longrightarrow(\neg(\textit{is-prop}\ \varphi)))$

abbreviation $omsp::\sigma$
where $omsp \equiv \forall_{\boldsymbol{\sigma}}\varphi.\ (\neg(\textit{maint-suf}\ \varphi))\longrightarrow(\neg(\textit{is-prop}\ \varphi))$

abbreviation $opr::\sigma$
where $opr \equiv \forall_{\boldsymbol{\sigma}}\varphi.\ (\neg(\textit{is-prop}\ \varphi))\longrightarrow(\neg(\mathbf{X}(\textit{is-rat}\ \varphi)))$

abbreviation *osr*::σ
 where $osr \equiv \forall_{\boldsymbol{\sigma}}\varphi.\ \forall_{\boldsymbol{g}} g.\ (\neg(\textit{sup-rat}\ \varphi)) \longrightarrow (\neg(\mathbf{X}(\textit{is-rat}\ \varphi)))$

abbreviation *psr*::σ
 where $psr \equiv \forall_{\boldsymbol{\sigma}}\varphi.\ (\textit{is-prop}\ \varphi \wedge (\textit{sup-rat}\ \varphi)) \longrightarrow (\mathbf{X}(\textit{is-rat}\ \varphi))$

abbreviation *rv*::σ
 where $rv \equiv \forall_{\boldsymbol{\sigma}}\varphi.\ (\textit{is-rat}\ \varphi) \longrightarrow \varphi$

4.3.2 Time instance t_1

The following section starts with an axiomatic description of the Constitution's state at t_1. This also includes some preparation for t_2, namely defining amendment *amd1* and giving axioms on what ought to be valid at t_2.

Before proceeding to t_2, we will prove some properties valid at t_1, in particular that there is no dictatorship at t_1 with the given axioms.

At t_1 *Congress* is the legislative, the *President* is the executive and the *Courts* are the judiciary. We write *President* for constant P in continuous text for better readability but use P in commands to keep names short.

axiomatization where
 Con-Leg-t1: $\lfloor \textit{is-leg Congress} \rfloor_{t1}$ **and**
 P-Exe-t1: $\lfloor \textit{is-exe}\ P \rfloor_{t1}$ **and**
 Cou-Jud-t1: $\lfloor \textit{is-jud Courts} \rfloor_{t1}$

All of the above defined properties for an instance of time are valid at t_1.

axiomatization where
 oap-t1: $\lfloor oap \rfloor_{t1}$ **and**
 osp-t1: $\lfloor osp \rfloor_{t1}$ **and**
 omsp-t1:$\lfloor omsp \rfloor_{t1}$ **and**
 opr-t1: $\lfloor opr \rfloor_{t1}$ **and**
 rv-t1: $\lfloor rv \rfloor_{t1}$ **and**
 osr-t1: $\lfloor osr \rfloor_{t1}$ **and**
 psr-t1: $\lfloor psr \rfloor_{t1}$

Here are two suggestions of what *amd1* might look like.

definition *amd1a*::σ
 where $amd1a \equiv \exists_{\boldsymbol{\sigma}}\varphi.\ (\neg(\textit{maint-suf}\ \varphi)) \wedge ((\textit{is-prop}\ \varphi))$
definition *amd1b*::σ
 where $amd1b \equiv \forall_{\boldsymbol{\sigma}}\varphi.\ (\textit{is-prop}\ \varphi) \longrightarrow ((\textit{maint-suf}\ \varphi) \vee \neg(\textit{maint-suf}\ \varphi))$

Neither are optimal solutions. Indeed, there is no optimal solution for the presented framework.

This is because what we want *amd1* to say is that it is not necessary for all proposed amendments to maintain all states' suffrage in Senate. In other words we want condition

$$omsp \equiv \forall_{\boldsymbol{\sigma}}\varphi.\ (\neg(\textit{maint-suf}\ \varphi)) \longrightarrow (\neg(\textit{is-prop}\ \varphi))$$

to be omitted at t_2. This, however is not the same as requiring the amendment to be the negation of *omsp* as *amd1a* does. The negation would require at least one φ::σ to expressly *not* maintain suffrage rights for some state *and* be proposed. Yet, it were acceptable both if such a φ existed and if it didn't. We do not want to demand such a φ into existence.

One could therefore choose to use *amd1b* that states a proposed φ may either satisfy the *maint-suf* condition or it may not. Unfortunately, this is a tautology since $a \longrightarrow b$ is always true if b is always true. The b in this case is tautology $(\textit{maint-suf}\ \varphi) \vee \neg(\textit{maint-suf}\ \varphi)$ and thus always true.

Although the suggested amendments do not constitute ideal amendments for the desired outcome, we shall still use them. They help to illustrate how one can reason about amendments within this framework.

Notice that one could introduce a logic like *deontic logic*[31] to reason about the necessity of *omsp*, either requiring it to be necessarily true or not. We choose not to do this in order to avoid inadvertent errors due to a mixture of temporal logic and other logics.

Next there are a few axioms that pave the way for the state at t_2.

Amendments *amd1a* and *amd1b* are both proposed and have support for ratification at t_1, so they may be ratified at the next instance.

axiomatization where
amd1a-prop-t1: $\lfloor \textit{is-prop amd1a} \rfloor_{t1}$ **and**
amd1a-sup-rat-t1: $\lfloor \textit{sup-rat amd1a} \rfloor_{t1}$ **and**
amd1b-prop-t1: $\lfloor \textit{is-prop amd1b} \rfloor_{t1}$ **and**
amd1b-sup-rat-t1: $\lfloor \textit{sup-rat amd1b} \rfloor_{t1}$

The distribution of powers stays the same at the next instance: *Congress* is the legislative, the *President* the executive and *Courts* are the judiciary.

axiomatization where
XCon-Leg-t1: $\lfloor \mathbf{X}(\textit{is-leg Congress}) \rfloor_{t1}$ **and**
XP-Exe-t1: $\lfloor \mathbf{X}(\textit{is-exe P}) \rfloor_{t1}$ **and**
XCou-Jud-t1: $\lfloor \mathbf{X}(\textit{is-jud Courts}) \rfloor_{t1}$

All properties defined in 4.3.1 are valid next time, except for *maint-suf*. This is to ensure that we can introduce an amendment at t_2 that does not satisfy *maint-suf*.
In a way the amendment to Art. V is implemented by simply not using $\lfloor \mathbf{X}\ \textit{omsp} \rfloor_{t1}$ as axiom, rather than by working with one of the above suggested amendments *amd1a* and *amd1b*.

One could criticize two aspects of this approach. **Firstly**, the fact that not an actual amendment is used to bring about the change but rather the lack of an axiom. As argued above this is not possible, however. **Secondly**, it shouldn't be necessary for us to explicitly state which axioms to keep and which to give up when transitioning to the next time point. It would be

[31] For an overview of deontic logic, see [McN19]

preferable if the logical system automatically kept all axioms that do not lead to contradictions and discarded the problematic ones. We will see in 4.4.5 why this is not easily done and content ourselves with the solution at hand.

Observe that our logic is suitable to express this problem in the sense that we would run into inconsistencies, were we to keep condition *omsp* for t_2 and also introduce an amendment *amd2* with ¬(*maint-suf amd2*).

axiomatization where
Xoap-t1: $\lfloor \mathbf{X}\ oap \rfloor_{t1}$ **and**
Xosp-t1: $\lfloor \mathbf{X}\ osp \rfloor_{t1}$ **and**
Xopr-t1: $\lfloor \mathbf{X}\ opr \rfloor_{t1}$ **and**
Xrv-t1: $\lfloor \mathbf{X}\ rv \rfloor_{t1}$ **and**
Xosr-t1: $\lfloor \mathbf{X}\ osr \rfloor_{t1}$ **and**
Xpsr-t1: $\lfloor \mathbf{X}\ psr \rfloor_{t1}$

Using the axioms provided above, we shall prove that there is no dictatorship at t_1. This requires the proof of facts *only-g-power-t1* meaning that g is the only governmental institution with *power*(legislative, executive, judicial) at t_1. Since g is different for each *power* no dictatorship can be in place at t_1.

lemma *only-Con-Leg-t1*: $\lfloor \forall_{\boldsymbol{g}} g.\ (\textit{is-leg}\ g) \longrightarrow (g = \textit{Congress}) \rfloor_{t1}$
unfolding *Defs* **using** *unique-is-leg Con-Leg-t1*
by (*simp add*: *global-valid-def local-valid-def tallB-g-def tall-g-def tand-def teq-def timp-def*)

lemma *only-P-Exe-t1*: $\lfloor \forall_{\boldsymbol{g}} g.\ (\textit{is-exe}\ g) \longrightarrow (g = P) \rfloor_{t1}$
unfolding *Defs* **using** *unique-is-exe P-Exe-t1*
by (*simp add*: *global-valid-def local-valid-def tallB-g-def tall-g-def tand-def teq-def timp-def*)

lemma *only-Cou-Jud-t1*: $\lfloor \forall_{\boldsymbol{g}} g.\ (\textit{is-jud}\ g) \longrightarrow (g = \textit{Courts}) \rfloor_{t1}$
unfolding *Defs* **using** *unique-is-jud Cou-Jud-t1*
by (*simp add*: *global-valid-def local-valid-def tallB-g-def tall-g-def tand-def teq-def timp-def*)

With these we can prove theorem *noDictatorship-t1*.

theorem *noDictatorship-t1*: $\lfloor \neg\ \textit{Dictatorship} \rfloor_{t1}$
unfolding *Defs* **using** *only-Con-Leg-t1 only-P-Exe-t1 only-Cou-Jud-t1*
by (*metis* (*no-types*, *lifting*) *Dictatorship-def g.distinct*(*1*) *local-valid-def tallB-g-def tall-g-def tand-def teq-def timp-def*)

Finally we check whether the axioms so far are even satisfiable by asking Nitpick to find a satisfying model for *True*. Note that we will repeat this test for time instances t_2 and t_3. Since we only ever add axioms and don't remove any, proceeding from one time instance to the next, it is sufficient to only consider the last model provided. We will present this when checking for satisfiability at t_3.

lemma *T-basic-sat-t1*: *True* **nitpick**[*satisfy*,*user-axioms*,*show-all*,*card time* = *4*]**oops**

4.3.3 Time instance t_2

As before there are three parts to t_2. This time they differ a little in structure.

The description of the current state is not given by a set of axioms but rather conclusions drawn from the preparation at t_1. This also includes proofs for the validity of *amd1a* and *amd1b.* The preparation for the next instance of time introduces the new amendment *amd2.* The proof for the non-existence of dictatorship at t_2 is practically the same as in t_1.

Based on axioms *XCon-Leg-t1*, *XP-Exe-t1* and *XCou-Jud-t1* we can now deduct that *Congress* is still the legislative, the *President* the executive and *Courts* are the judiciary.

lemma *Con-Leg-t2*:$\lfloor$*is-leg Congress*$\rfloor_{t2}$
  unfolding *Defs*
  using *XCon-Leg-t1 local-valid-def tnext-def t1-s-t2* **by** *auto*

lemma *P-Exe-t2*:$\lfloor$*is-exe P*$\rfloor_{t2}$
  unfolding *Defs* **using** *tnext-def XP-Exe-t1*
  using *XP-Exe-t1 local-valid-def tnext-def t1-s-t2* **by** *auto*

lemma *Cou-Jud-t2*:$\lfloor$*is-jud Courts*$\rfloor_{t2}$
  using *XCou-Jud-t1 local-valid-def tnext-def t1-s-t2* **by** *auto*

Analogously, we can refer to axioms *Xproperty-t1* to conclude that *property* is valid at t_2. These are the same properties we had for t_1 with the exception of *omsp.*

lemma *oap-t2*:$\lfloor$*oap*$\rfloor_{t2}$
  using *Xoap-t1 local-valid-def tnext-def t1-s-t2* **by** *auto*
lemma *osp-t2*:$\lfloor$*osp*$\rfloor_{t2}$
  using *Xosp-t1 local-valid-def tnext-def t1-s-t2* **by** *auto*
lemma *opr-t2*:$\lfloor$*opr*$\rfloor_{t2}$
  using *Xopr-t1 local-valid-def tnext-def t1-s-t2* **by** *auto*
lemma *rv-t2*:$\lfloor$*rv*$\rfloor_{t2}$
  using *Xrv-t1 local-valid-def tnext-def t1-s-t2* **by** *auto*
lemma *osr-t2*:$\lfloor$*osr*$\rfloor_{t2}$
  using *Xosr-t1 local-valid-def tnext-def t1-s-t2* **by** *auto*
lemma *psr-t2*:$\lfloor$*psr*$\rfloor_{t2}$
  using *Xpsr-t1 local-valid-def tnext-def t1-s-t2* **by** *auto*

Below are proofs for the amendments proposed previously.

As discussed above, the outline for a validity proof where an amendment *amd* is concerned is as follows:

t_i	t_{i+1}			
psr-t_i	*rv*-t_{i+1}			
is-prop amd<br>*sup-rat amd*	$\Big\}\underset{psr\text{-}t_i}{\Rightarrow}$	*is-rat amd*	$\underset{rv\text{-}t_{i+1}}{\Rightarrow}$	*amd*

This is exactly what we do with *amd1a*. Using *amd1a-prop-t1*, *amd1a-sup-rat-t1* and *psr-t1* we get that $\lfloor \mathbf{X}(\textit{is-rat amd1a}) \rfloor_{t1}$. By definition of **X** this means that $\lfloor \textit{is-rat amd1a} \rfloor_{t2}$ is true and by *rv-t2* that $\lfloor \textit{amd1a} \rfloor_{t2}$ is true.

lemma *amd1a-val-t2*: $\lfloor \textit{amd1a} \rfloor_{t2}$
proof –
  have $\lfloor \mathbf{X}(\textit{is-rat amd1a}) \rfloor_{t1}$
    using *amd1a-prop-t1 amd1a-sup-rat-t1 psr-t1 local-valid-def tallB-s-def tall-s-def tand-def timp-def tnext-def*
    by *auto*
  thus $\lfloor \textit{amd1a} \rfloor_{t2}$
    using *local-valid-def tallB-s-def tall-s-def timp-def tnext-def rv-t2 t1-s-t2*
    by *auto*
qed

See below that we can prove $\lfloor \textit{amd1b} \rfloor_{t2}$ with or without these axioms. We do not need to use the deduction rules provided by our axioms because *amd1b* is a tautology. Indeed, we can also show *amd1b*'s validity for t_1 and its global validity. This is not possible with *amd1a*.

lemma *amd1b-val-t2*: $\lfloor \textit{amd1b} \rfloor_{t2}$
  unfolding *Defs*
  by (*simp add*: *amd1b-def tallB-s-def tall-s-def timp-def tneg-def tor-def*)

lemma *amd1b-val-t2-2*: $\lfloor \textit{amd1b} \rfloor_{t2}$
  unfolding *Defs* **using** *amd1b-sup-rat-t1 amd1b-prop-t1 psr-t1 rv-t2*
  by (*simp add*: *amd1b-def tallB-s-def tall-s-def timp-def tneg-def tor-def*)

lemma *amd1b-val-t1*: $\lfloor \textit{amd1b} \rfloor_{t1}$
  unfolding *Defs*
  by (*simp add*: *amd1b-def tallB-s-def tall-s-def timp-def tneg-def tor-def*)

lemma *amd1b-val*: $\lfloor \textit{amd1b} \rfloor$
  unfolding *Defs*
  by (*simp add*: *amd1b-def tallB-s-def tall-s-def timp-def tneg-def tor-def*)

Now we introduce *amd2* which will transfer all governmental power to the *President*. Technically *amd2* does not bereave any state of its votes in Senate and would thus satifsy *maint-suf*. However, if Congress does not have any real power any more, then neither do its members, which would render any state's votes inane. So, in effect, we have that $\neg(\textit{maint-suf amd2})$.

Notice that we cannot declare $\neg(\textit{maint-suf amd2})$ to be globally valid since a state's votes in Senate depend on what the Constitution currently looks like. Were we to consider predicate *maint-suf* for *amd2* at a time when states have no suffrage in Senate ($\textit{maint-suf amd2}$) would be true.

definition *amd2*::σ **where** *amd2* $\equiv$ *is-leg P* $\wedge$ *is-exe P* $\wedge$ *is-jud P*
axiomatization where
  amd2-prop-t2: $\lfloor \textit{is-prop amd2} \rfloor_{t2}$ **and**
  amd2-sup-rat-t2: $\lfloor \textit{sup-rat amd2} \rfloor_{t2}$ **and**

amd2-not-maint-suf-t2: $\lfloor\neg(\textit{maint-suf amd2})\rfloor_{t2}$

As before we intend to keep all time dependant conditions except for *omsp* when transitioning to t_3.

axiomatization where
Xoap-t2: $\lfloor\mathbf{X}\ \textit{oap}\rfloor_{t2}$ **and**
Xosp-t2: $\lfloor\mathbf{X}\ \textit{osp}\rfloor_{t2}$ **and**
Xopr-t2: $\lfloor\mathbf{X}\ \textit{opr}\rfloor_{t2}$ **and**
Xrv-t2: $\lfloor\mathbf{X}\ \textit{rv}\rfloor_{t2}$ **and**
Xosr-t2: $\lfloor\mathbf{X}\ \textit{osr}\rfloor_{t2}$ **and**
Xpsr-t2: $\lfloor\mathbf{X}\ \textit{psr}\rfloor_{t2}$

In 4.2.1 we mentioned that we needed t_e for technical reasons. This is because we want to use above given axiom $\lfloor\mathbf{X}\textit{opr}\rfloor_{t2}$ without creating inconsistencies due to a missing successor for t_3.

$$\begin{aligned}
\lfloor\mathbf{X}\textit{opr}\rfloor_{t2} &\Rightarrow \lfloor\textit{opr}\rfloor_{t3}\\
&\Leftrightarrow \lfloor\forall_{\boldsymbol{\sigma}}\varphi.\ (\neg(\textit{is-prop}\ \varphi))\longrightarrow(\neg(\mathbf{X}(\textit{is-rat}\ \varphi)))\rfloor_{t3}\\
&\Leftrightarrow \lfloor\forall_{\boldsymbol{\sigma}}\varphi.\ (\mathbf{X}(\textit{is-rat}\ \varphi))\longrightarrow(\textit{is-prop}\ \varphi)\rfloor_{t3}\\
&\Leftrightarrow \forall\,\varphi.\ ((\mathbf{X}(\textit{is-rat}\ \varphi))t_3)\longrightarrow(\textit{is-prop}\ \varphi)\ t_3\\
&\Leftrightarrow \forall\,\varphi.\ \forall\,t'.((\textit{succ}\ t_3\ t')\longrightarrow(\textit{is-rat}\ \varphi)\ t')\longrightarrow(\textit{is-prop}\ \varphi)\ t_3
\end{aligned}$$

If t_3 does not have a successor $(\textit{succ}\ t_3\ t')$ will always be false, making $(\textit{succ}\ t_3\ t')\longrightarrow(\textit{is-rat}\ \varphi)\ t'$ always true which it shouldn't be. As soon as term $(\textit{is-prop}\ \varphi)\ t_3$ is not true for some φ, axiom $\lfloor\mathbf{X}\textit{opr}\rfloor_{t2}$ will cause an inconsistency.

We therefore want t_3 to have a successor. In order to avoid circular succession we introduce dummy instance t_e.

Analogously to the proof at 4.3.2, we prove properties *only-g-power-t2* for g, governmental institution and *power* $\in$ {*legilslative power*, *executive power*, *judicial power*} to use them in the proof for *noDictatorship-t2*.

lemma *only-Con-Leg-t2*: $\lfloor\forall_{\boldsymbol{g}}g.\ (\textit{is-leg}\ g)\longrightarrow(g = \textit{Congress})\rfloor_{t2}$
using *unique-is-leg Con-Leg-t2 global-valid-def local-valid-def tallB-g-def tall-g-def tand-def teq-def timp-def*
by *simp*

lemma *only-P-Exe-t2*: $\lfloor\forall_{\boldsymbol{g}}g.\ (\textit{is-exe}\ g)\longrightarrow(g = P)\rfloor_{t2}$
unfolding *Defs* **using** *unique-is-exe P-Exe-t2*
by (*simp add*: *global-valid-def local-valid-def tallB-g-def tall-g-def tand-def teq-def timp-def*)

lemma *only-Cou-Jud-t2*: $\lfloor\forall_{\boldsymbol{g}}g.\ (\textit{is-jud}\ g)\longrightarrow(g = \textit{Courts})\rfloor_{t2}$
unfolding *Defs* **using** *unique-is-jud Cou-Jud-t2*
by (*simp add*: *global-valid-def local-valid-def tallB-g-def tall-g-def tand-def teq-def timp-def*)

theorem *noDictatorship-t2*: $\lfloor\neg$ *Dictatorship*$\rfloor_{t2}$
unfolding *Defs* **using** *only-Con-Leg-t2 only-P-Exe-t2 only-Cou-Jud-t2 Dictatorship-def*
by (*metis* (*mono-tags*, *lifting*) *g.distinct*(*3*) *local-valid-def tallB-g-def tall-g-def tand-def teq-def timp-def*)

Lastly, we make sure that Nitpick can still find a satisfiable model for our axioms.

lemma *T-basic-sat-t2*: *True* **nitpick**[*satisfy*,*user-axioms*,*show-all*,*card time* = *4*]**oops**

4.3.4 Time instance t_3

The remainder of this section is rather simple. We prove properties for new time instance t_3 using previously provided axioms *Xproperty-t2*. We then proceed to show that *amd2* is valid with the reasoning given above and use it to prove that there is now a dictatorship.

lemma *oap-t3*:$\lfloor$*oap*$\rfloor_{t3}$
using *Xoap-t2 local-valid-def tnext-def t2-s-t3* **by** *auto*
lemma *osp-t3*:$\lfloor$*osp*$\rfloor_{t3}$
using *Xosp-t2 local-valid-def tnext-def t2-s-t3* **by** *auto*
lemma *opr-t3*:$\lfloor$*opr*$\rfloor_{t3}$
using *Xopr-t2 local-valid-def tnext-def t2-s-t3* **by** *auto*
lemma *rv-t3*:$\lfloor$*rv*$\rfloor_{t3}$
using *Xrv-t2 local-valid-def tnext-def t2-s-t3* **by** *auto*
lemma *osr-t3*:$\lfloor$*osr*$\rfloor_{t3}$
using *Xosr-t2 local-valid-def tnext-def t2-s-t3* **by** *auto*
lemma *psr-t3*:$\lfloor$*psr*$\rfloor_{t3}$
using *Xpsr-t2 local-valid-def tnext-def t2-s-t3* **by** *auto*

lemma *amd2-val-t3*:$\lfloor$*amd2*$\rfloor_{t3}$
proof –
have $\lfloor$**X**(*is-rat amd2*)$\rfloor_{t2}$
using *amd2-prop-t2 amd2-sup-rat-t2 local-valid-def tallB-s-def tall-s-def tand-def timp-def tnext-def psr-t2*
by *auto*
thus $\lfloor$*amd2*$\rfloor_{t3}$
using *local-valid-def tallB-s-def tall-s-def timp-def tnext-def rv-t3 t2-s-t3*
by *auto*
qed

Since *amd2* $\equiv$ *is-leg P* $\wedge$ *is-exe P* $\wedge$ *is-jud P* we can easily show that the condition for *Dictatorship* is satisfied.

theorem *Dictatorship-t3*:$\lfloor$*Dictatorship*$\rfloor_{t3}$
proof –
have $\lfloor$*is-leg P* $\wedge$ *is-exe P* $\wedge$ *is-jud P*$\rfloor_{t3}$
using *amd2-val-t3 amd2-def*
by (*simp add*: *local-valid-def tand-def*)
thus $\lfloor$*Dictatorship*$\rfloor_{t3}$

by (*meson Dictatorship-def local-valid-def*)
qed

To conclude we check for satisfiability again.

lemma *T-basic-sat-t3*: *True* **nitpick**[*satisfy*,*user-axioms*,*show-all*,*format* = *2*,*card time* = *4*]**oops**

The following satisfying model is the result:

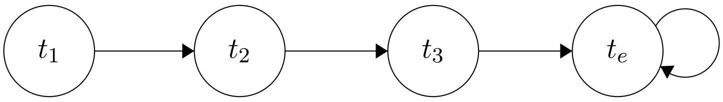

t_1	t_2	t_3	t_e
is-exe P	*is-exe P*	*is-exe P*	*is-exe P*
is-jud Courts	*is-jud Courts*	*is-jud P*	*is-jud P*
is-leg Congress	*is-leg Congress*	*is-leg P*	*is-leg P*
maint-suf amd1a			
sup-prop Congress amd1a			
is-prop amd1a			
sup-rat amd1a	*is-rat amd1a*		
maint-suf amd1b			
sup-prop Congress amd1b			
is-prop amd1b			
sup-rat amd1b	*is-rat amd1b*		
	¬ (*maint-suf amd2*)		
	sup-prop Congress amd2		
	is-prop amd2		
	sup-rat amd2	*is-rat amd2*	

Here are a few notes on the presentation of the model.

Firstly, the graph expresses which instances of time succeed which. Arrows point from the instances of time to their respective successors. We have already seen this graph in 4.2, when we introduced instances of time.

Secondly, Nitpick refers to all properties that we introduce with a definition by λ-expressions since they depend on the properties introduced with constants. For example predicate *amd1b* is referred to by term

$$(\lambda x.\ \text{-})(t_1 := \mathit{True},\ t_2 := \mathit{True},\ t_3 := \mathit{True},\ t_e := \mathit{True})$$

As a tautology, *amd1b* is true at every instance of time. Hence, its λ-term evaluates to *True* for all instances of time.
Likewise *amd2* and *Dictatorship* are represented by

$$(\lambda x.\ \text{-})(t_1 := \mathit{False},\ t_2 := \mathit{False},\ t_3 := \mathit{True},\ t_e := \mathit{True})$$

With the given values for constants *is-exe*, *is-leg* and *is-jud*, the value of *amd2* and *Dictatorship* will be *False* at t_1 and t_2 and *True* at the remaining points in time. This also means that we could add a term *property Dictatorship* whenever we have *property amd2* in the above table.

Thirdly, the presented model is a truncated version of Nitpick's model since we choose to omit points that are not relevant to the argument. For instance, we have that *sup-prop Courts amd1a* at t_1 but since the legislative, *Congress*, supports the proposition of *amd1a* and thus makes it possible for *amd1a* to be proposed it is not relevant to us that *Courts* support the proposition of *amd1a*, too.

4.4 What to avoid when modelling

4.4.1 Data type *time* as *int*

The initial idea for the Constitution's model was to map everything possible to the computer, including concepts like

> The House of Representatives shall be [...] chosen every second Year[...].[32]

Isabelle offers a rich theory on *integers*. It thus seemed to be a good idea to work with *int* as basis for data type *time* defined thus:

type-synonym *time=int*

One could then have identified year n with *int* n and expressed a two-year election cycle the following way:

elections-2yearCycle: $\lfloor \forall\, t.(\textit{T-lastE}\ t) \longrightarrow (\textit{T-nextE}\ (t+2))\rfloor$
where *T-lastE* and *T-nextE* are predicates on whether or not the last election was at t and whether or not the next election will be at $t+2$ respectively.

Unfortunately working with integers like this renders Isabelle's tools more or less unusable. This is because Isabelle must then provide a theory to work with integers which makes the tools very slow to respond, if they do not run out of time, altogether. Especially Nitpick[33] is not helpful anymore since now it has to provide infinite models at which it generally fails.

Since neither the rich theory of integers nor an infinite number of time instances were necessary in our case, dispensing with command *time=int* in favour of helpful versions of Nitpick and Sledgehammer[34] was the appropriate choice.

[32] See U.S. Const. art.I, §2., cl.1.
[33] S. [Bla19]
[34] S. [BP19]

4.4.2 Functions instead of relations

In order to be able to introduce next operator **X**, a successor function is necessary that provides exactly one successor for each time instance. This was done with a *relation* in our model. Given that the mapping of (*succ* t) to t is unique, one could also consider using a function, rather than a relation. This way the definition would be shorter as one wouldn't have to specify whether two instances are related or not for each pair of time instances.
A function and corresponding **X** could be defined as follows:

function *succ*::*time*⇒*time* **where**
succ $t_1 = t_2$
|*succ* $t_2 = t_3$
|*succ* $t_3 = t_e$
|*succ* $t_e = t_e$
by *pat-completeness auto*

definition *tnext* :: $\sigma \Rightarrow \sigma$ (**X**-)
where $\mathbf{X}\varphi \equiv (\lambda t.\ \forall t'.\ ((succ(t) = t') \longrightarrow \varphi\ t'))$

Observe that requirement ($succ\ t\ t'$) has now been replaced with $succ(t) = t'$ in the definition of *tnext*.

Now why is the function not a desirable option? As with *int* for *time* the tools grew very slow or not usable at all when using the function. Presumably, that is because of the comprehensive theory that comes with functions. Its provision makes Isabelle slow.

Furthermore, the functions themselves are somewhat cumbersome to work with. For example, consider line **by** *pat-completeness auto*. If the domain is a recursively defined data type the definition of a function requires a proof that it will terminate. Of course, our data type *time* has not been defined recursively. However, its definition uses the same syntax as a recursive data type would. Therefore, a proof of the function's termination on arbitrary elements of the domain is necessary for the function to be well-defined.

Taking into account that we do not need this theory, we might as well dispense with it.

4.4.3 Numerous type declarations

As mentioned in 4.4.1 the original goal was to represent as many notions of the Constitution as possible. This meant that distinguishing a fair amount of different types of topics was necessary. The most straightforward way to do this seemed to be to introduce various data types. See below for an example.

typedecl *h* — Type for humans

typedecl *s* — Type for states
typedecl *g* — Type for government institutions
typedecl *r* — Type for rights
typedecl *e* — Type for elections
typedecl *time* — Type for time
type-synonym σ=(*time*⇒*bool*)

Unfortunately, this made the domain for finding (counter-)models a complex field to navigate. While the numerous data types made it easy to accurately distinguish between different notions and express formulas, the computer did not know about appropriate cardinalities for the declared data types. Take for example the following proposition:

$$\lfloor \forall\, h.\ (h\ \mathit{memOf}\ \mathit{Senate}) \leftrightarrow \\ ((\exists\, el.(\ el\ \mathit{elecFor}\ \mathit{E\text{-}Senate}) \wedge (\mathit{elects}\ el\ h\ \mathit{E\text{-}Senate})))\rfloor$$

based on constants

Senate :: *g*
E-Senate :: *e*
memOf :: *h* ⇒ *g* ⇒ σ (- *memOf* -)
elecFor :: *h*⇒*e*⇒σ (- *elecFor* -)
elects :: *h*⇒*h*⇒*e*⇒σ (*elects* - - -)

It is meant to express that senators are elected by electors[35] or put differently, that a human *h* is a member of *Senate* iff there is another human *el* that is an elector for election of type *E-Senate* and elects *h* at that election.

This proposition requires reasoning on data types *g*,*e*,*h* and *time* (because of σ=(*time*⇒*bool*)). If no cardinalities are given for the respective types, Nitpick will try to combine all kinds of combinations of cardinalities when looking for a model. The number of different cardinality combinations grows exponentially with the number of data types rendering Nitpick useless.

A partial solution for this is to introduce finite data types where possible and determine the cardinalites when calling Nitpick, as it will try to cover all of these combinations with the limited computation time it has. See for example:

typedecl *h* — Type for humans
typedecl *s* — Type for states
datatype *g* = — Type for government institutions
Congress — Congress of the US
| *HoR* — House of Representatives
| *Senate* — Senate

typedecl *r* — Type for rights
datatype *e* = — Type for elections
E-HoR — elections for the HoR

[35] cf. U.S. Const. amend.XVII, §1.

| *E-Senate* — elections for the Senate
typedecl *time* — Type for time
type-synonym σ=(*time*⇒*bool*)

with *Nitpick*[*card g= 3*,*card e=2*].
The disadvantage here is that introducing finite types is not always possible which means the problem can only be solved partially.

Furthermore, the finite data types reduce flexibility in the modelling or at least require repeated adjustments when they do not suffice for the currently modelled concepts anymore. This makes their use prone to inconsistencies and errors in general.

4.4.4 Polymorphism

A similar problem to the one discussed in 4.4.3 is the one polymorphism poses. Take for example the following alternative definition of *memOf*.

memOf :: $'a \Rightarrow\ 'b \Rightarrow \sigma$ (- *memOf* -)
It is elegant since it would allow for an instance of any type $'a$ to be declared a member of an instance of any type $'b$. It is not necessary to specify types $'a$ and $'b$ upon defining *memOf*.

As with the unknown cardinalities in 4.4.3 however, Isabelle's tools need to guess the required specific type. This means trying all possibilities until a suitable one is found. This takes time and thus makes this theoretically elegant concept an inconvenient one in practice.

Notice that the problem becomes even more pronounced when working with quantifiers, such as

tall :: $('a \Rightarrow \sigma) \Rightarrow \sigma$ ($\forall$) **where** $\forall\, \Phi \equiv \lambda t. \forall\, x.\ \Phi(x)(t)$
In this case Isabelle has to find the right type for $'a$ as well as check for all instances of the presumable type whether Φ holds true of it.

Incidentally, this is why we introduce operators $\forall_{\boldsymbol{g}}$ and $\forall_{\boldsymbol{\sigma}}$ in 4.2. Knowing that types g and σ are the only ones that will be quantified over, it is sensible to introduce these instead of a polymorphic version as given above. This spares Isabelle the work of searching for the right type and thus leads to quicker response times of its tools. Also, it forces us to be precise with our formulas, which in turn contributes to cleaner code and a better understanding of the concepts involved.

4.4.5 Higher order quantification and the Frame Problem

This section is somewhat more extensive than its predecessors of 4.4 since we are going to look into two different topics.

In the first part the eponymous higher order quantification will be discussed. The reason there is a second part is that the exemplary formulas

given are an attempt at solving the so called *Frame Problem*[36]. This Frame Problem is of significance not only to AI in general, but for finding a good model of our time dependant Constitution in particular and shall therefore be presented as well. This will be the second part.

As mentioned in 4.3.2 it would be convenient to only have to make statements about what changes when transitioning from one time instance to the next without mentioning everything that stays the same. This would allow us to introduce amendments and thus make changes to the Constitution without having to state explicitly which of the currently valid properties will still be valid.

A possible way to realize this, is by using an axiom that states the following two properties:

- If φ is valid at t_i with successor t_{i+1} and there is no ψ contradicting φ, valid at t_{i+1}, then φ stays valid at t_{i+1}.
- If φ is valid at t_i with successor t_{i+1} and there is a ψ contradicting φ, valid at t_{i+1}, then φ is not valid at t_{i+1}.

With a very rudimentary notion of what it means when "ψ contradicts φ", we could express these points as follows:

definition *isNeg* $::\sigma\Rightarrow\sigma\Rightarrow bool$
where *isNeg* $\varphi\ \psi \equiv \forall t.\ (\varphi\ t) \longleftrightarrow (\neg(\psi\ t))$

axiom1:
$\forall \varphi.\ \forall t2.\ ((\exists t1.\ (succ\ t1\ t2) \land (\varphi\ t1) \land (\forall \psi.\ \neg((isNeg\ \varphi\ \psi) \land (\psi\ t2)))) \longrightarrow (\varphi\ t2))$
and
axiom2:
$\forall \varphi.\ \forall t2.\ ((\exists t1.\ (succ\ t1\ t2) \land (\varphi\ t1) \land (\exists \psi.\ ((isNeg\ \varphi\ \psi) \land (\psi\ t2)))) \longrightarrow \neg(\varphi\ t2))$

Unfortunately, these axioms are not well suited to actually help with modelling the amendment process.

One reason is, of course, that *isNeg* is a very simple way of checking for contradictions in φ and ψ. It only verifies whether the negation of one evaluates the same way as the other does. This does not take their respective composition into account. What if $\psi \equiv \psi_1 \land \psi_2$ and only ψ_2 contradicted φ or what if φ was a tautology and ψ inherently contradictory? While not a trivial task, one could improve *isNeg* e.g. by analysing φ and ψ in a recursive manner and comparing their respective components. The check for contradictions shall not be the centre of our attention, however. Let us instead turn to *axiom1* and *axiom2*.

Another reason why these axioms are only marginally helpful, is that they quantify over formulas. This makes them rather strong axioms which,

[36] S. [Sha16]

in theory, should make them very useful, but in practice makes them inconvenient to work with.

The reason is that in order to use them for a proof, Isabelle will have to check the premises of the implications given, including the verification of

- $\forall \psi.\ \neg\ (\mathit{isNeg}\ \varphi\ \psi \wedge \psi\ \mathit{t2})$ and
- $\exists \psi.\ \mathit{isNeg}\ \varphi\ \psi \wedge \psi\ \mathit{t2}$

respectively. This a very difficult task since the verification of neither of these terms is decidable.
For the former to be verified, Isabelle would have to check all possible formulas ψ for contradictions to φ with the number of formulas being infinite. For the latter, potentially infinitely many formulas have to be assessed with termination once a suitable ψ has been found. Hence, checking the second term is at least semi-decidable, but not decidable.

In both cases, reasoning will be very slow since Isabelle will try to check all available candidates for ψ. Since *axiom1* and *axiom2* are given as axioms, Isabelle will try to use them whenever trying to find a proof and in attempting to verify the axioms' left-hand sides run out of computation time. One could, of course, increase the available computation time for tools such as Sledgehammer but given that the verification is not decidable, this is not likely to help.

Consequently, it is best to avoid axioms like the above. One should note here that higher order quantification per se is not an evil to be avoided at all costs. Higher order logic is very expressive and can make it easy to formulate concepts in a very concise manner. This is why we have used it throughout the simulation. There are two differences between the above quantification and the ones we have used.

The first is that quantifiers used, almost always occurred at the beginning of a formula, not as just a component of a bigger formula. So, unless there was reasoning about the entire formula "$\forall \varphi.\ \Phi(\varphi)$", there was no need to reason about the quantifiers.

The second is that all quantification over formulas, as opposed to quantification over constants, was always time-dependant. So if such quantification was used in an axiom, it still wasn't universally valid but only for certain instances of time. This resulted in these axioms not being used in proofs by default.

All in all, higher order quantification can be very helpful but has to be used adequately.

Let us now turn to the Frame Problem. We shall determine what it is, how it is related to our model and how it is connected to legal texts in general.

There are different notions of what the Frame Problem is. Its more narrow, technical version originated in logic-based AI and was then taken

up by philosophers who interpreted it in a more general way and extended the question.[37] We will only be concerned with the former.

The Frame Problem was first presented by McCarthy and Hayes in 1969.[38] They observed that for any program to successfully interact with its environment, it needed an internal representation of its environment. This would allow for example a robot to judge whether an action was successful or not. Simply stating which actions resulted in which changes of the environment was not enough, however. To see why, consider the following example.
There is a tea cup *cup* on which the following actions can be performed:

1. *fill*(*cup*)
2. *move*(*cup*,*position*)

You could describe the state of *cup* by giving information on its position and on whether or not it is full. Upon conducting an action the parameters of *cup* are changed as follows:

1. After *fill*(*cup*) the cup is full.
2. After *move*(*cup*,*position*) the cup is at *position*.

Assume that *cup* is empty and at position x. If *fill*(*cup*) and *move*(*cup*,y) are conducted consecutively, we assume *cup* to be full and at y. However, this does not have to be the outcome. What if moving the cup also meant tilting it, so that something poured out? Both states "*full* and y" and "*empty* and y" are conceivable. It is therefore necessary to also consider the *non-effects* of each action. We could determine them as follows:

1. After *fill*(*cup*) the cup is at position x, if its original position was x.
2. After *move*(*cup*,*position*) the cup is full, if it was full before the move and empty otherwise.
   Here we assume that *move*(*cup*,*position*) will not result in spilled tea.

These additional assumptions would allow for only one state after conducting *fill*(*cup*) and *move*(*cup*,y). It is the expected "*full* and y".

Stating all effects and non-effects requires many statements. Assuming that there are n actions to be conducted in the environment and m properties of the environment, we would have to state $n \cdot m$ assumptions. Solving the Frame Problem means to give an adequate description of the environment without having to use $n \cdot m$ statements. "Frame" refers to the description of the environment.

[37] S. [Sha16]-"Introduction"
[38] S. [MH69],"4.3. The Frame Problem"

In the following, we shall determine how the Frame Problem is connected to modelling the Constitution in HOL.

Since we are interested in verifying an argument about amending and thus changing the Constitution, we are faced with finding a solution to the Frame Problem. If there were no changes, we wouldn't need to consider their effects, after all. In our case, an *action* is the introduction of an amendment and the *environment* to be described is the Constitution itself.

There are two factors that make our task more complicated than the standard Frame Problem.

The **first** is that the ratification of each amendment poses a different action since the amendments are all different and thus warrant different changes. Here we assume that the legislative would not make the effort of ratifying an amendment more than once. With each amendment representing a different action, one can only describe the Constitution and its changes if all amendments are known from the beginning. If that is not the case the Frame Problem extends to also accommodate changing actions.

In our specific case, the amendments were known from the beginning. Nonetheless this was only partially helpful due to the **second** factor. As mentioned in 4.3.1 when defining *rv* we blend the *contents* of amendments with the logic used to *argue about* them. This results in an action not only changing the environment, but also the scope for actions. Hence, even with the actions known, we cannot state their effects globally, i.e. for all time instances since the effects themselves depend on the instance.

To solve the Frame Problem we chose to explicitly state all effects and non-effects by determining which properties will be valid at which time instance. This was feasible as the number of parameters to describe each instance was low, as were the number of actions performed and the number of points in time. Recall that we only had the properties defined at 4.3.1, such as *oap*, *rv* or *Dictatorship*. The actions we conducted were the respective ratifications of *amd1a*, *amd1b* and *amd2*.

To conclude this section, let us consider one final connection between modelling the Constitution and the Frame Problem. It is the so called legal convention of *lex posterior derogat legi priori* (*lex posterior*).
According to Parry & Grant it is the principle "that a later legal rule prevails over a prior inconsistent legal rule".[39] This is in effect what *axiom1* and *axiom2* in the first part of this section were meant to express.

Finding a good representation of *lex posterior* is another facet of finding a solution to the Frame Problem. Because, if we do not want to manually go through all rules that are potentially valid at a certain time to then discard the ones that contradict newer rules, we are forced to use suitable meta rules for reasoning about the rules in question. Solutions to the Frame Problem

[39] S. [BG09], p.346

might provide such meta rules.

We mention this last point since finding a good representation of *lex posterior* is an important task for any automated legal reasoning based on legal texts that are inconsistent due to their gradual development over time. This also holds for the Constitution and its amendments. For example U.S. Const. art.I, §3., cl.1. states that senators shall be elected by the *legislatives* of their home states while U.S. Const. amend.XVII, cl.1. states that senators shall be elected by the *people* of their home states. Today only Amend. XVII is used as basis for state senator elections and yet Art. I remains unchanged.

With this in mind the narrow version of the Frame Problem needs to be extended to accommodate *lex posterior* in legal contexts.

5 Outlook

This section aims at providing an outlook on which questions might be studied next as well as presenting the limits of this work by addressing unanswered questions.

The first point to mention here is that no records were found that attest to the argument Gödel himself devised. It seems that there simply are no first-hand records on Gödel's reservations concerning the Constitution. Nonetheless, the author has not exhausted all sources due to a lack of availability of some of them in Berlin. Most notably, there are the "Kurt Gödel Papers"[40] and the "Kurt Gödel Papers on microfilm"[41] respectively. The latter are a selection of the former but more widely available since they can be accessed wherever the microfilm is available. The full collection is only available at the Princeton University Library. They contain personal notes amongst other documents. These might help in retracing Gödel's thoughts. It should be noted that fellow scientists with access to the microfilms could not find a sketch of the argument. However, the collection at the Princeton University Library also contains his correspondence with Morgenstern and as we have seen in 3.1 Gödel would turn to Morgenstern with questions concerning the hearing.[42] Since their correspondence was not published in "Kurt Gödel - Collected Works"[43] one would have to turn to the collection in Princeton to examine those.

In addition to faults that Gödel himself might have found with the Constitution, it would be interesting to study and potentially formalize other problems of the Constitution from a logician's perspective. There seem to be both logical problems[44] and problems with respect to content, for example

[40] S. [GA85]

[41] S. [Edi99]

[42] S. [Mor71]

[43] This is a collection of Gödel's scientific work in five volumes, the fifth containing his correspondence with persons of surnames starting with H-Z, see [Göd03].

[44] S. [Bel04]

when it comes to ensuring a balanced distribution of powers[45]. In terms of logical problems, the above mentioned formalization of *lex posterior* would be of particular interest, given that it is a widely used principal in law.

With respect to the argument formalized in this work and its connection to the Frame Problem, we chose to enlist all necessary axioms on effects and non-effects. Formalizing the argument with the currently available solutions for the Frame Problem[46] remains to be done as it might lead to new insights.

This work only dealt with the contents of the Constitution relevant to the argument formalized. Analysing and representing more of its contents will be the next step in meeting growing demands in automated legal reasoning.

When it comes to formalizing legal concepts in general the collaboration of logicians and legal scholars is essential to achieve better results. Given that the problems presented above are in nature interdisciplinary they should also be solved in an interdisciplinary context.

6 Conclusion

In the course of this work we delved into an argument on how a legal dictatorship could be instantiated on the basis of the US Constitution.

The starting point was an anecdote on how Gödel tried to teach his examiner at the citizenship hearing about the potential for a dictatorship in the USA based on a fault of the Constitution.

Not being able to locate a document on Gödel's own thoughts concerning this flaw, we concentrated on an argument by Guerra-Pujol[47]. The basic idea is to amend Art. V which entrenches some parts of the Constitution to enable the introduction of another amendment that dissolves the separation of powers and installs a dictator.

This argument was then formalized with Isabelle/HOL using a simple temporal logic with different instances of time to represent the stages the Constitution passes through until it allows for a dictatorship.

Having successfully verified the validity of the argument, we turned to lessons learned throughout the process. Among these, there were some directly connected to Isabelle and its weaknesses and thus of a technical nature, but also some that were of theoretical nature and in part warrant further research.

The author was a little sad to not have found Gödel's original argument but greatly enjoyed looking for it in letters and diary entries and definitely learned a lot throughout the process of modelling and verifying the argument.

---

[45] S. [GP13], IV.
[46] S. [Sha16], "5.The Frame Problem Today"
[47] S. [GP13]

7 Declaration of Authorship

I declare that the bachelor's thesis I am submitting is entirely my own work except where otherwise indicated. I declare that I have clearly indicated the presence of all material I have quoted from other sources, including any diagrams, charts, tables or graphs and that I have clearly indicated the presence of all paraphrased material with appropriate references. This thesis has not been submitted, either partially or in full, for a qualification at any other university.

Berlin, 23rd August 2019

Valeria Zahoransky

8 References

References

[BA19] Christoph Benzmüller and Peter Andrews. Church's type theory. In Edward N. Zalta, editor, *The Stanford Encyclopedia of Philosophy*. Metaphysics Research Lab, Stanford University, summer 2019 edition, 2019.

[Bal17] George W. Baltzell. The constitution of the united states, the bill of rights & all amendments. https://constitutionus.com/, 2017. Accessed: 2019-08-20.

[Bar16] Nicholas W. Barber. Why entrench? *International Journal Of Constitutional Law*, 14:325–350, 2016.

[Bel04] Alexander S. Belenky. The solvability of a set partitioning problem and a logical mistake in article 2 of the u.s. constitution. *Mathematical and Computer Modelling*, 40(1):1 – 3, 2004.

[BG09] J Craig Barker and John P Grant. *Parry & Grant encyclopaedic dictionary of international law*. Oxford University Press, 3rd ed. edition, 2009.

[Bla19] Jasmin Christian Blanchette. *Picking Nits–A user's guide to nitpick for Isabelle/HOL*, 2019. http://isabelle.in.tum.de/dist/doc/nitpick.pdf.

[BP19] Jasmin Christian Blanchette and Lawrence C. Paulson. *Hammering away– A user's guide to Sledgehammer for Isabelle/HOL*, 2019. https://isabelle.in.tum.de/dist/doc/sledgehammer.pdf.

[Con87] U.S. Const. Constitution of the united states, 1787.

[Daw97] John W. Dawson. *Logical dilemmas: The life and work of Kurt Gödel*. AK Peters/CRC Press, 1997.

[Edi99] Various Authors & Editors. *Kurt Gödel Papers on microfilm*. Brill, Leiden, The Netherlands, 1999.

[GA85] Kurt Gödel and Various Authors. *Kurt Gödel Papers*. Manuscripts Division, Department of Rare Books and Special Collections, Princeton University Library, 1985.

[Gar18] James Garson. Modal logic. In Edward N. Zalta, editor, *The Stanford Encyclopedia of Philosophy*. Metaphysics Research Lab, Stanford University, fall 2018 edition, 2018.

[GG15] Valentin Goranko and Antony Galton. Temporal logic. In Edward N. Zalta, editor, *The Stanford Encyclopedia of Philosophy*. Metaphysics Research Lab, Stanford University, winter 2015 edition, 2015.

[Göd78] Kurt Gödel. Briefe an marianne gödel 1906-1978. https://www.digital.wienbibliothek.at/wbr/nav/classification/2559756, 1906-1978. Accessed: 2019-08-20.

[Göd03] Kurt Gödel. *Kurt Gödel Collected Works: Volume V: Correspondence, H-Z.* 2003.

[GP13] FE Guerra-Pujol. Gödel's loophole. *Cap. UL Rev.*, 41:637, 2013.

[LB09] Sanford Levinson and Jack M Balkin. Constitutional dictatorship: Its dangers and its design. *Minn. L. Rev.*, 94:1789, 2009.

[McN19] Paul McNamara. Deontic logic. In Edward N. Zalta, editor, *The Stanford Encyclopedia of Philosophy*. Metaphysics Research Lab, Stanford University, summer 2019 edition, 2019.

[MH69] John McCarthy and Patrick J. Hayes. Some philosophical problems from the standpoint of artificial intelligence. In B. Meltzer and D. Michie, editors, *Machine Intelligence 4*, pages 463–502. Edinburgh University Press, 1969. reprinted in McC90.

[Mor71] Oskar Morgenstern. Oskar morgenstern's account of kurt gödel's naturalization. *Dorothy Morgenstern Thomas collection*, 1971.

[Mor16] Oskar Morgenstern. Tagebuch. digitale edition: 1917 bis 1977. http://gams.uni-graz.at/archive/objects/o:ome.b47-47/methods/sdef:TEI/get?mode=b47-47, 2016. Accessed: 2019-08-07.

[Sha16] Murray Shanahan. The frame problem. In Edward N. Zalta, editor, *The Stanford Encyclopedia of Philosophy*. Metaphysics Research Lab, Stanford University, spring 2016 edition, 2016.

[Smu92] Raymond M Smullyan. *Gödel's incompleteness theorems*. Oxford University Press, 1992.

[Wan87] Hao Wang. *Reflections on Kurt Gödel*. MIT Press, 1987.

[Wen19] Makarius Wenzel. *The Isabelle/Isar Reference Manual*, 2019. https://isabelle.in.tum.de/doc/isar-ref.pdf.

[You06] Palle Yourgrau. *A world without time: The forgotten legacy of Gödel and Einstein*. Basic Books, 2006.
