# Supplementary material for: LogiKEy workbench: Deontic logics, logic combinations and expressive ethical and legal reasoning (Isabelle/HOL dataset)
Source: Data Brief. 2020 Oct 15;33:106409. doi: 10.1016/j.dib.2020.106409 (PMC7586073; doi:10.1016/j.dib.2020.106409)
Supplement: Supplementary file 1 [file mmc1.zip › 2020-DataInBrief-Data/GewirthArgument.html]

xml version="1.0" encoding="utf-8"?


Theory GewirthArgument (Isabelle2019: June 2019)


# Theory GewirthArgument

theory GewirthArgument  
imports Extended\_CJ\_DDL

```
theory GewirthArgument                    (* by David Fuenmayor and C. Benzmüller, 2019 *)
   imports Extended_CJ_DDL     
begin
nitpick_params[user_axioms=true, show_all, expect=genuine, format = 3] 


section ‹Gewith's Ethical Theory›

type_synonym p = "e⇒m" (**Type for properties (function from individuals to sentence meanings)*)

(**ActsOnPurpose is a relational constant. (ActsOnPurpose A E) reads as "A is acting on purpose E":*)
consts ActsOnPurpose::"e⇒m⇒m" 
(**(NeedsForPurpose A P E) reads as "A needs to have property P in order to reach purpose E":*)
consts NeedsForPurpose::"e⇒p⇒m⇒m"

definition PPA:: "p" where "PPA a ≡ ❙∃E. ActsOnPurpose a E" (** Definition of PPA*)
axiomatization where essentialPPA: "⌊❙∀a.(PPA a ❙→ ❙□⇧D(PPA a))⌋⇧D" (**PPA is an essential property*)
lemma recognizeOtherPPA: "∀c d. (⌊PPA (Agent d)⌋⇩d) ⟶ ⌊PPA (Agent d)⌋⇩c" using essentialPPA by blast

consts Good::"e⇒m⇒m"
axiomatization where explGoodness1: "⌊❙∀a P. ActsOnPurpose a P ❙→ Good a P⌋⇧D"
axiomatization where explGoodness2: "⌊❙∀P M a. Good a P ❙∧ NeedsForPurpose a M P ❙→ Good a (M a)⌋⇧D"
axiomatization where explGoodness3: "⌊❙∀φ a. ❙◇⇩pφ ❙→ ❙O⟨φ | ❙□⇧DGood a φ⟩⌋⇧D"

consts FWB::"p" (**Enjoying freedom and well-being (FWB) is a property (i.e.~has type @{text "e⇒m"})*)

axiomatization where
explicationFWB1: "⌊❙∀P a. NeedsForPurpose a FWB P⌋⇧D"
axiomatization where explicationFWB2: "⌊❙∀a. ❙◇⇩p FWB a⌋⇧D"  
axiomatization where explicationFWB3: "⌊❙∀a. ❙◇⇩p ❙¬FWB a⌋⇧D"  

lemma "⌊❙O⇩iφ ❙→ ❙◇⇩pφ⌋" using sem_5ab by simp
axiomatization where OIOAC: "⌊❙O⇩iφ ❙→ ❙O⇩i(❙◇⇩aφ)⌋⇧D"

consts InterferesWith::"e⇒m⇒m"
axiomatization where explicationInterference: "⌊(❙∃b. InterferesWith b φ) ❙↔ ❙¬❙◇⇩aφ⌋"
lemma "⌊❙∀a. (❙∃b. InterferesWith b (FWB a)) ❙↔ ❙¬❙◇⇩a(FWB a)⌋" using explicationInterference by blast
lemma InterferenceWithFWB: "⌊❙∀a.  ❙◇⇩a(FWB a) ❙↔ (❙∀b. ❙¬InterferesWith b (FWB a))⌋" using explicationInterference by blast

definition RightTo::"e⇒(e⇒m)⇒m" where "RightTo a φ ≡ ❙O⇩i(❙∀b. ❙¬InterferesWith b (φ a))"

(**Axiom consistency checked: Nitpick finds a two-world model (card w=2)*)
lemma True nitpick[satisfy, card c = 1, card e = 1, card w = 2] oops

section ‹Gewirth's Argument for the PGC›

(**Following Beyleveld's summary, the main steps of the argument are (with original numbering): *)
(**(1) I act voluntarily for some (freely chosen) purpose E (equivalent --by definition-- to: I am a PPA).*)
(**(2) E is (subjectively) good (i.e.~I value E proactively).*)
(**(3) My freedom and well-being (FWB) are generically necessary conditions of my agency (i.e.~I need them to achieve any purpose whatsoever).*)
(**(4) My FWB are necessary goods (at least for me).*)
(**(5) I have (maybe nobody else does) a claim right to my FWB.*)
(**(13) Every PPA has a claim right to their FWB.*)


(**The following is a formalized proof for the main conclusion of Gewirth's argument, which 
asserts that the following sentence is valid from every PPA's standpoint: "Every PPA has a 
claim right to its freedom and well-being (FWB)" *)
theorem PGC: shows "⌊❙∀x. PPA x ❙→ (RightTo x FWB)⌋⇧D"
proof - 
 {fix C::c (**'C' is some arbitrarily chosen context (agent's perspective)*)
   {fix I::"e" (**'I' is some arbitrarily chosen individual (agent's perspective)*)
     {fix E::m (**'E' is some arbitrarily chosen purpose*)
      { assume P1: "⌊ActsOnPurpose I E⌋⇩C" (**(1) I act voluntarily on purpose E:*)
           (**(1a) I am a PPA:*)
        from P1 have P1a: "⌊PPA I⌋⇩C" using PPA_def by auto
           (**(2) purpose E is good for me:*)
        from P1 have C2: "⌊Good I E⌋⇩C" using explGoodness1 essentialPPA by meson       
           (**(3) I need FWB for any purpose whatsoever:*)
        from explicationFWB1 have C3: "⌊❙∀P. NeedsForPurpose I FWB P⌋⇧D" by simp
        hence "∃P.⌊Good I P ❙∧ NeedsForPurpose I FWB P⌋⇧D" 
             using explicationFWB2 explGoodness3 sem_5ab by blast
           (**FWB is (a priori) good for me (in a kind of definitional sense):*)
        hence "⌊Good I (FWB I)⌋⇧D" using explGoodness2 by blast       
           (**(4) FWB is an (a priori) necessary good for me:*)
        hence C4: "⌊❙□⇧D(Good I (FWB I))⌋⇩C" by simp  
         (**I ought to pursue my FWB on the condition that I consider it a necessary good:*)
        have "⌊❙O⟨FWB I | ❙□⇧D(Good I) (FWB I)⟩⌋⇩C" using explGoodness3 explicationFWB2 by blast
           (**There is an (other-directed) obligation to my FWB:*)
        hence "⌊❙O⇩i(FWB I)⌋⇩C" using explicationFWB2 explicationFWB3 C4 CJ_14p by fastforce 
           (**It must therefore be the case that my FWB is possible:*)
        hence "⌊❙O⇩i(❙◇⇩a(FWB I))⌋⇩C" using OIOAC by simp       
           (**There is an obligation for others not to interfere with my FWB:*)
        hence "⌊❙O⇩i(❙∀a. ❙¬InterferesWith a (FWB I))⌋⇩C" using InterferenceWithFWB by simp        
           (**(5) I have a claim right to my FWB:*)
        hence C5: "⌊RightTo I FWB⌋⇩C" using RightTo_def by simp }
      (**I have a claim right to my FWB (since I act on some purpose E):*) 
    hence "⌊ActsOnPurpose I E ❙→ RightTo I FWB⌋⇩C" by (rule impI) }
      (** "allI" is the logical generalization rule: all-quantifier introduction*)
    hence "⌊❙∀P. ActsOnPurpose I P ❙→ RightTo I FWB⌋⇩C" by (rule allI)    
      (**I have a claim right to my FWB since I am a PPA:*)
    hence "⌊PPA I ❙→ RightTo I FWB⌋⇩C" using PPA_def by simp }
    (**Every agent has a claim right to its FWB since it is a PPA:*)
  hence "∀x. ⌊PPA x ❙→ RightTo x FWB⌋⇩C" by simp }
    (**(13) For every perspective C: every agent has a claim right to its FWB:*)
  thus C13: "∀C. ⌊❙∀x. PPA x ❙→ (RightTo x FWB)⌋⇩C" by (rule allI)  
qed

(**The following is a weaker variant of PGC: the agent of some context claims rights to FWB if it holds itself as a PPA.*)
lemma PGC_weak: "∀C. ⌊PPA (Agent C) ❙→ (RightTo (Agent C) FWB)⌋⇩C" using PGC by simp

section ‹An example›

(**In the following, we illustrate how to draw some inferences building upon Gewirth's PGC.
Note that all theorems below can be proven using Isabelle's term rewriting engine.*)

consts X::c (**Context of use X (to which a certain speaker agent corresponds)*)
consts Y::c (**Context of use Y (to which another speaker agent corresponds)*)

(**The agent (of context) X holds itself as a PPA:*)
axiomatization where AgentX_X_PPA: "⌊PPA (Agent X)⌋⇩X"

(**The agent (of another context) Y holds the agent (of context) X  as a PPA:*)
lemma AgentY_X_PPA: "⌊PPA (Agent X)⌋⇩Y" using AgentX_X_PPA recognizeOtherPPA by simp

(**Now the agent (of context) Y holds itself as a PPA: *)
axiomatization where AgentY_Y_PPA: "⌊PPA (Agent Y)⌋⇩Y"

(**The agent Y claims a right to FWB:*)
lemma AgentY_Y_FWB: "⌊RightTo (Agent Y) FWB⌋⇩Y" using AgentY_Y_PPA PGC_weak by simp

(**The agent Y accepts X claiming a right to FWB:*)
lemma AgentY_X_FWB: "⌊RightTo (Agent X) FWB⌋⇩Y" using AgentY_X_PPA PGC by simp

(**The agent Y accepts an (other-directed) obligation of non-interference with X's FWB:*)
lemma AgentY_NonInterference_X_FWB: "⌊❙O⇩i(❙∀z. ❙¬InterferesWith z (FWB (Agent X)))⌋⇩Y" using AgentY_X_FWB RightTo_def by simp

(**Axiom consistency checked: Nitpick finds a two-world model (card w=2).*)
lemma True nitpick[satisfy, card c = 1, card e = 1, card w = 2] oops

end
```
