# Supplementary material for: LogiKEy workbench: Deontic logics, logic combinations and expressive ethical and legal reasoning (Isabelle/HOL dataset)
Source: Data Brief. 2020 Oct 15;33:106409. doi: 10.1016/j.dib.2020.106409 (PMC7586073; doi:10.1016/j.dib.2020.106409)
Supplement: Supplementary file 1 [file mmc1.zip › 2020-DataInBrief-Data/Chisholm_CJ_DDL_Dyadic.html]

xml version="1.0" encoding="utf-8"?


Theory Chisholm\_CJ\_DDL\_Dyadic (Isabelle2019: June 2019)


# Theory Chisholm\_CJ\_DDL\_Dyadic

theory Chisholm\_CJ\_DDL\_Dyadic  
imports CJ\_DDL\_Tests

```
theory Chisholm_CJ_DDL_Dyadic imports CJ_DDL  CJ_DDL_Tests (*Christoph Benzmüller & Xavier Parent, 2019*)

begin (* Chisholm Example *)
consts go::τ tell::τ kill::τ

 nitpick_params [user_axioms,show_all,format=2] (*Settings for model finder.*)

(*It ought to be that Jones goes to assist his neighbors.*)
  definition  "D1 ≡ ❙O❙⟨go❙|❙⊤❙⟩"  
(*It ought to be that if Jones goes, then he tells them he is coming.*)
  definition  "D2 ≡ ❙O❙⟨tell❙|go❙⟩"  
(*If Jones doesn't go, then he ought not tell them he is coming.*)
  definition  "D3 ≡ ❙O❙⟨❙¬tell❙|❙¬go❙⟩"
(*Jones doesn't go. (This is encoded as a locally valid statement.)*)
  definition  "D4 ≡ ❙¬go" 


(*** Chisholm ***)
 (* All-wide scoping is not leading to a dependent set of the axioms.*)
 lemma "⌊(D1 ❙∧ D2 ❙∧ D3) ❙→ D4⌋"  nitpick oops (*countermodel*)
 lemma "⌊(D1 ❙∧ D2 ❙∧ D4) ❙→ D3⌋"  nitpick oops (*countermodel*)
 lemma "⌊(D1 ❙∧ D3 ❙∧ D4) ❙→ D2⌋"  nitpick oops (*countermodel*)
 lemma "⌊(D2 ❙∧ D3 ❙∧ D4) ❙→ D1⌋"  nitpick oops (*countermodel*)
 (* Chisholm is thus an adequate modeling. *)

 (* Consistency *)
 lemma "⌊(D1 ❙∧ D2 ❙∧ D3)⌋ ∧ ⌊D4⌋⇩l" nitpick [satisfy] oops (*Consistent? Yes*) 
 lemma assumes "⌊(D1 ❙∧ D2 ❙∧ D3)⌋ ∧ ⌊D4⌋⇩l" shows False nitpick oops (*Inconsistent? No*)
 (* Queries *)
 lemma assumes "⌊(D1 ❙∧ D2 ❙∧ D3)⌋ ∧ ⌊D4⌋⇩l" shows "⌊❙O❙⟨❙¬tell❙|❙⊤❙⟩⌋⇩l" nitpick oops (*Should James not tell? No*) 
 lemma assumes "⌊(D1 ❙∧ D2 ❙∧ D3)⌋ ∧ ⌊D4⌋⇩l" shows "⌊❙O❙⟨tell❙|❙⊤❙⟩⌋⇩l"
   sledgehammer nitpick oops (*Should James tell? Timeout*)
 lemma assumes "⌊(D1 ❙∧ D2 ❙∧ D3)⌋ ∧ ⌊D4⌋⇩l" shows "⌊❙O❙⟨kill❙|❙⊤❙⟩⌋⇩l"  nitpick oops (*Should James kill? No*)

end
```
