# Supplementary material for: LogiKEy workbench: Deontic logics, logic combinations and expressive ethical and legal reasoning (Isabelle/HOL dataset)
Source: Data Brief. 2020 Oct 15;33:106409. doi: 10.1016/j.dib.2020.106409 (PMC7586073; doi:10.1016/j.dib.2020.106409)
Supplement: Supplementary file 1 [file mmc1.zip › 2020-DataInBrief-Data/SDL.html]

xml version="1.0" encoding="utf-8"?


Theory SDL (Isabelle2019: June 2019)


# Theory SDL

theory SDL  
imports Main

```
theory SDL        (* SDL: Standard Deontic Logic. C. Benzmüller & X. Parent, 2019 *)
  imports Main 
begin
 typedecl i (*Type for possible worlds.*)  
 type_synonym σ = "(i⇒bool)"
 type_synonym γ = "σ⇒σ" 
 type_synonym ρ = "σ⇒σ⇒σ"

 consts R::"i⇒i⇒bool" (infixr "R" 70) (*Accessibility relation.*)  
        aw::i (*Actual world.*)  

 abbreviation SDLtop::σ ("❙⊤") where "❙⊤ ≡ λw. True" 
 abbreviation SDLbot::σ ("❙⊥") where "❙⊥ ≡ λw. False" 
 abbreviation SDLnot::γ ("❙¬_"[52]53) where "❙¬φ ≡ λw. ¬φ(w)" 
 abbreviation SDLand::ρ (infixr"❙∧"51) where "φ❙∧ψ ≡ λw. φ(w) ∧ ψ(w)"   
 abbreviation SDLor::ρ (infixr"❙∨"50) where "φ❙∨ψ ≡ λw. φ(w) ∨ ψ(w)"   
 abbreviation SDLimp::ρ (infixr"❙→"49) where "φ❙→ψ ≡ λw. φ(w) ⟶ ψ(w)"  
 abbreviation SDLequ::ρ (infixr"❙↔"48) where "φ❙↔ψ ≡ λw. φ(w) ⟷ ψ(w)"  

 abbreviation SDLobligatory::γ ("OB") where "OB φ ≡ λw. ∀v.  w R v ⟶ φ(v)"
 abbreviation SDLpermissible::γ ("PE") where "PE φ ≡ ❙¬(OB(❙¬φ))"
 abbreviation SDLimpermissible::γ ("IM") where "IM φ ≡ OB(❙¬φ)"
 abbreviation SDLomissible::γ ("OM") where "OM φ ≡ ❙¬(OB φ)"
 abbreviation SDLoptional::γ ("OP") where "OP φ ≡ (❙¬(OB φ) ❙∧  ❙¬(OB(❙¬φ)))"

 abbreviation SDLvalid::"σ⇒bool" ("⌊_⌋"[7]105)  where "⌊A⌋ ≡ ∀w. A w"       (*Global validity.*)
 abbreviation SDLvalidcw::"σ⇒bool" ("⌊_⌋⇩l"[7]105)    where "⌊A⌋⇩l ≡ A aw"   (*Validity in actual world.*)

(*Possibilist Quantification.*)
 abbreviation SDLforall ("❙∀") where "❙∀Φ ≡ λw.∀x. (Φ x w)"
 abbreviation SDLforallB (binder"❙∀"[8]9) where "❙∀x. φ(x) ≡ ❙∀φ"  
 abbreviation SDLexists ("❙∃") where "❙∃Φ ≡ λw.∃x. (Φ x w)"   
 abbreviation SDLexistsB (binder"❙∃"[8]9) where "❙∃x. φ(x) ≡ ❙∃φ" 

 axiomatization where D: "⌊❙¬ ((OB φ) ❙∧ (OB (❙¬ φ)))⌋" (*Axiom D: seriality of r.*)
 lemma seriality: "(∀w. ∃v. w R v)" using D by blast

 abbreviation SDLobl::γ ("❙○<_>") where "❙○<φ> ≡  OB φ"  (*New syntax: A is obligatory.*)

(*Consistency confirmed by model finder Nitpick.*) 
 lemma True nitpick[satisfy,user_axioms,expect=genuine] oops 
 
(*Barcan formulas.*) 
 lemma Barcan:         "⌊(❙∀d. ❙○<φ(d)>) ❙→ (❙○<❙∀d. φ(d)>)⌋" by simp  
 lemma ConverseBarcan: "⌊(❙○<❙∀d. φ(d)>) ❙→ (❙∀d. ❙○<φ(d)>)⌋" by simp 
end
```
