# Supplementary material for: LogiKEy workbench: Deontic logics, logic combinations and expressive ethical and legal reasoning (Isabelle/HOL dataset)
Source: Data Brief. 2020 Oct 15;33:106409. doi: 10.1016/j.dib.2020.106409 (PMC7586073; doi:10.1016/j.dib.2020.106409)
Supplement: Supplementary file 1 [file mmc1.zip › 2020-DataInBrief-Data/Lewis_DDL.html]

xml version="1.0" encoding="utf-8"?


Theory Lewis\_DDL (Isabelle2019: June 2019)


# Theory Lewis\_DDL

theory Lewis\_DDL  
imports Main

```
(*X. Parent, 25/08/2019, and C. Benzmüller, 2020 *)
(*This file introduces David Lewis DDL and studies its
relationship with the Aqvist-Hansson DDL  *)
(*This uses material from a chapter for the second volume of the
handbook of deontic logic  *)

theory Lewis_DDL  imports Main  
begin       
typedecl i ―‹type for possible worlds›  
type_synonym τ = "(i⇒bool)" 
consts  aw::i ―‹actual world›  
 
abbreviation(input) mtrue  :: "τ" ("❙⊤") where "❙⊤ ≡ λw. True" 
abbreviation(input) mfalse :: "τ" ("❙⊥")  where "❙⊥ ≡ λw. False"   
abbreviation(input) mnot   :: "τ⇒τ" ("❙¬_"[52]53)  where "❙¬φ ≡ λw. ¬φ(w)" 
abbreviation(input) mand   :: "τ⇒τ⇒τ" (infixr"❙∧"51) where "φ❙∧ψ ≡ λw. φ(w)∧ψ(w)"   
abbreviation(input) mor    :: "τ⇒τ⇒τ" (infixr"❙∨"50) where "φ❙∨ψ ≡ λw. φ(w)∨ψ(w)"   
abbreviation(input) mimp   :: "τ⇒τ⇒τ" (infixr"❙→"49) where "φ❙→ψ ≡ λw. φ(w)⟶ψ(w)"  
abbreviation(input) mequ   :: "τ⇒τ⇒τ" (infixr"❙↔"48) where "φ❙↔ψ ≡ λw. φ(w)⟷ψ(w)"  

abbreviation(input) mbox :: "τ⇒τ" ("□") where "□φ ≡ λw.  ∀v. φ(v)" 
definition ddediomond  :: "τ⇒τ" ("◇") where "◇φ ≡ λw. ∃v. φ(v)"

consts r :: "i⇒τ"  (infixr "r" 70)
 ―‹the betterness relation r, used in definition of ○<_|_>  ›  
abbreviation(input) mopt  :: "τ⇒τ" ("opt<_>") 
  where "opt<φ> ≡ (λv. ( (φ)(v) ∧ (∀x. ((φ)(x)  ⟶  v r x) )) )" 
abbreviation(input) msubset :: "τ⇒τ⇒bool" (infix "❙⊆" 53)
  where "φ ❙⊆ ψ ≡ ∀x. φ x ⟶ ψ x"

(*David Lewis's evaluation rule for the conditional *)
abbreviation(input) mcond  :: "τ⇒τ⇒τ" ("○<_|_>")
  where "○<ψ|φ> ≡ (λv. (¬(∃x. (φ)(x))∨  (∃x. ((φ)(x)∧(ψ)(x) ∧ (∀y. ((y r x) ⟶ (φ)(y)⟶(ψ)(y)))))))"
definition ddeperm :: "τ⇒τ⇒τ" ("P<_|_>") 
  where "P<ψ|φ> ≡❙¬○<❙¬ψ|φ>"

(*Hansson-Aqvist's evaluation rule for the conditional *)
abbreviation(input) mcond2  :: "τ⇒τ⇒τ" ("○o<_|_>")
  where "○o<ψ|φ> ≡ (λv. opt<φ> ❙⊆ ψ)"

abbreviation limitedness  where "limitedness ≡ (∀φ. (∃x. (φ)x) ⟶ (∃x. opt<φ>x))"
abbreviation transitivity where "transitivity ≡ (∀x y z. (x r y ∧ y r z) ⟶ x r z)"
abbreviation totalness where "totalness ≡ (∀x y. (x r y ∨ y r x))"
abbreviation reflexivity where "reflexivity ≡ (∀x. (x r x))"

abbreviation(input) valid :: "τ⇒bool" ("⌊_⌋"[8]109)
  where "⌊p⌋ ≡ ∀w. p w"
definition cjactual :: "τ⇒bool" ("⌊_⌋⇩l"[7]105) 
  where "⌊p⌋⇩l ≡ p(aw)"
     
lemma True nitpick [satisfy, user_axioms, show_all, expect=genuine] oops     

(*relation with optimality*)
(*The lewis rule is stronger*)
lemma "⌊○o<ψ|φ>❙→○<ψ|φ>⌋" nitpick oops  (*countermodel*)
lemma "⌊○<ψ|φ>❙→○o<ψ|φ>⌋" by blast

lemma
assumes "limitedness"
  assumes "transitivity" 
  shows "⌊○o<ψ|φ>❙→○<ψ|φ>⌋"
  by (smt assms(1) assms(2))

(*axioms of E holding irrespective of the properties of r*)
lemma Abs:"⌊○<ψ|φ> ❙→ □○<ψ|φ>⌋"  by auto
lemma Nec:"⌊□ψ ❙→ ○<ψ|φ>⌋" by blast 
lemma Ext:"⌊□(φ⇩1❙↔φ⇩2) ❙→ (○<ψ|φ⇩1> ❙↔ ○<ψ|φ⇩2>)⌋" by simp 
lemma Id:"⌊○<φ|φ>⌋" by auto 
lemma Sh:"⌊○<ψ|φ⇩1❙∧φ⇩2> ❙→ ○<(φ⇩2❙→ψ)|φ⇩1>⌋" by blast
lemma MP:"(⌊φ⌋∧⌊φ❙→ψ⌋)⟹⌊ψ⌋" by blast
lemma N:"⌊φ⌋⟹⌊□φ⌋" by auto 
 
(*axioms of E holding if r transitive and totale*)

lemma COK:"⌊○<(ψ⇩1❙→ψ⇩2)|φ> ❙→ (○<ψ⇩1|φ> ❙→ ○<ψ⇩2|φ>)⌋" nitpick oops (*countermodel*)

lemma 
  assumes "transitivity" 
  shows COK:"⌊○<(ψ⇩1❙→ψ⇩2)|φ> ❙→ (○<ψ⇩1|φ> ❙→ ○<ψ⇩2|φ>)⌋" nitpick oops  (*countermodel*) 

  lemma 
  assumes "totalness"
  shows COK:"⌊○<(ψ⇩1❙→ψ⇩2)|φ> ❙→ (○<ψ⇩1|φ> ❙→ ○<ψ⇩2|φ>)⌋" nitpick oops (*countermodel*)
  
lemma 
  assumes "transitivity" 
  assumes "totalness"
  shows COK:"⌊○<(ψ⇩1❙→ψ⇩2)|φ> ❙→ (○<ψ⇩1|φ> ❙→ ○<ψ⇩2|φ>)⌋" by (smt assms(1) assms(2))
  
lemma D : "⌊◇φ ❙→ (○<ψ|φ> ❙→ P<ψ|φ>)⌋"  nitpick oops (*countermodel*)

lemma
 assumes "totalness"
  shows D : "⌊◇φ ❙→ (○<ψ|φ> ❙→ P<ψ|φ>)⌋"  by (smt assms ddediomond_def ddeperm_def) 

lemma  Sp: "⌊( P<ψ|φ> ❙∧ ○<(ψ❙→χ)|φ>) ❙→ ○<χ|(φ❙∧ψ)>⌋" nitpick oops (*countermodel*)

lemma
 assumes "transitivity"
 shows Sp: "⌊( P<ψ|φ> ❙∧ ○<(ψ❙→χ)|φ>) ❙→ ○<χ|(φ❙∧ψ)>⌋" by (smt assms ddeperm_def) 

lemma CM: "⌊(○<ψ|φ>❙∧○<χ|φ>)❙→ ○<χ|φ❙∧ψ>⌋" nitpick oops (*countermodel*)

lemma
  assumes "transitivity"
  shows CM: "⌊(○<ψ|φ>❙∧○<χ|φ>)❙→ ○<χ|φ❙∧ψ>⌋" nitpick oops (*countermodel*)

lemma
  assumes "transitivity"
  assumes "totalness"
  shows CM: "⌊(○<ψ|φ>❙∧○<χ|φ>)❙→ ○<χ|φ❙∧ψ>⌋" by (metis assms(1) assms(2)) 

lemma SA:"⌊○<ψ|φ> ❙→  ○<ψ|φ❙∧χ>⌋" nitpick oops (*countermodel*)
end
```
