# Supplementary material for: LogiKEy workbench: Deontic logics, logic combinations and expressive ethical and legal reasoning (Isabelle/HOL dataset)
Source: Data Brief. 2020 Oct 15;33:106409. doi: 10.1016/j.dib.2020.106409 (PMC7586073; doi:10.1016/j.dib.2020.106409)
Supplement: Supplementary file 1 [file mmc1.zip › 2020-DataInBrief-Data/Chisholm_E.html]

xml version="1.0" encoding="utf-8"?


Theory Chisholm\_E (Isabelle2019: June 2019)


# Theory Chisholm\_E

theory Chisholm\_E  
imports E

```
theory Chisholm_E imports E                 (*Christoph Benzmüller & Xavier Parent, 2019*)

begin (* Chisholm Example *)
consts go::σ tell::σ kill::σ

  nitpick_params [user_axioms,expect=genuine,show_all,format=2] (*settings for the model finder*)

(*It ought to be that Jones goes to assist his neighbors.*)
  abbreviation  "D1 ≡ ❙○<go>"  
(*It ought to be that if Jones goes, then he tells them he is coming.*)
  abbreviation  "D2w ≡ ❙○<go ❙→ tell>"  
  abbreviation  "D2n ≡ go ❙→ ❙○<tell>"  
(*If Jones doesn't go, then he ought not tell them he is coming.*)
  abbreviation  "D3w ≡ ❙○<❙¬go ❙→ ❙¬tell>" 
  abbreviation  "D3n ≡ ❙¬go ❙→ ❙○<❙¬tell>" 
(*Jones doesn't go. (This is encoded as a locally valid statement.)*)
  abbreviation  "D4 ≡ ❙¬go" 


(*** Chisholm_A ***)
 (* All-wide scoping is not leading to a dependent set of the axioms.*)
 lemma "⌊(D1  ❙∧ D2w ❙∧ D3w) ❙→ D4⌋"   nitpick oops (*countermodel*)
 lemma "⌊(D1  ❙∧ D2w ❙∧ D4)  ❙→ D3w⌋"  by blast  (*proof*)
 lemma "⌊(D1  ❙∧ D3w ❙∧ D4)  ❙→ D2w⌋"  nitpick oops (*countermodel*)
 lemma "⌊(D2w ❙∧ D3w ❙∧ D4)  ❙→ D1⌋"   nitpick oops (*countermodel*)
 (* Chisholm_A is thus an inadequate modeling. *)

 (* Consistency *)
 lemma "⌊(D1 ❙∧ D2w ❙∧ D3w)⌋ ∧ ⌊D4⌋⇩l" nitpick [satisfy] oops (*Consistent? Yes*) 
 lemma assumes "⌊(D1 ❙∧ D2w ❙∧ D3w)⌋ ∧ ⌊D4⌋⇩l" shows False nitpick oops (*Inconsistent? No*)
 (* Queries *)
 lemma assumes "⌊(D1 ❙∧ D2w ❙∧ D3w)⌋ ∧ ⌊D4⌋⇩l" shows "⌊❙○<❙¬tell>⌋⇩l" nitpick oops (*Should James not tell? No*) 
 lemma assumes "⌊(D1 ❙∧ D2w ❙∧ D3w)⌋ ∧ ⌊D4⌋⇩l" shows "⌊❙○<tell>⌋⇩l"  using assms by auto (*Should James tell? Yes*)
 lemma assumes "⌊(D1 ❙∧ D2w ❙∧ D3w)⌋ ∧ ⌊D4⌋⇩l" shows "⌊❙○<kill>⌋⇩l"  nitpick oops (*Should James kill? No*)


(*** Chisholm_B ***)
 (* All-narrow scoping is leading to a dependent set of the axioms.*)
 lemma "⌊(D1  ❙∧ D2n ❙∧ D3n) ❙→ D4⌋"   nitpick oops (*countermodel*)
 lemma "⌊(D1  ❙∧ D2n ❙∧ D4)  ❙→ D3n⌋"  nitpick oops  (*countermodel*)
 lemma "⌊(D1  ❙∧ D3n ❙∧ D4)  ❙→ D2n⌋"  by blast  (*proof*)
 lemma "⌊(D2n ❙∧ D3n ❙∧ D4)  ❙→ D1⌋"   nitpick oops (*countermodel*)
 (* Chisholm_B is thus an inadequate modeling. *)

 (* Consistency *)
 lemma "⌊(D1 ❙∧ D2n ❙∧ D3n)⌋ ∧ ⌊D4⌋⇩l" nitpick [satisfy] oops (*Consistent? Yes*) 
 lemma assumes "⌊(D1 ❙∧ D2n ❙∧ D3n)⌋ ∧ ⌊D4⌋⇩l" shows False nitpick oops (*Inconsistent? No*)
 (* Queries *)
 lemma assumes "⌊(D1 ❙∧ D2n ❙∧ D3n)⌋ ∧ ⌊D4⌋⇩l" shows "⌊❙○<❙¬tell>⌋⇩l" using assms by auto (*Should James not tell? Yes*) 
 lemma assumes "⌊(D1 ❙∧ D2n ❙∧ D3n)⌋ ∧ ⌊D4⌋⇩l" shows "⌊❙○<tell>⌋⇩l"  using assms by auto (*Should James tell? Yes*)
 lemma assumes "⌊(D1 ❙∧ D2n ❙∧ D3n)⌋ ∧ ⌊D4⌋⇩l" shows "⌊❙○<kill>⌋⇩l"  using assms by smt (*Should James kill? Yes*)


(*** Chisholm_C ***)
 (* Wide-narrow scoping is leading to independence of the axioms.*)
 lemma "⌊(D1  ❙∧ D2w ❙∧ D3n) ❙→ D4⌋"   nitpick oops (*countermodel*)
 lemma "⌊(D1  ❙∧ D2w ❙∧ D4)  ❙→ D3n⌋"  nitpick oops (*countermodel*)
 lemma "⌊(D1  ❙∧ D3n ❙∧ D4)  ❙→ D2w⌋"  nitpick oops (*countermodel*)
 lemma "⌊(D2w ❙∧ D3n ❙∧ D4)  ❙→ D1⌋"   nitpick oops (*countermodel*)
 (* Chisholm_C is thus fine from this perspective. *)

 (* Consistency *)
 lemma "⌊(D1 ❙∧ D2w ❙∧ D3n)⌋ ∧ ⌊D4⌋⇩l" nitpick [satisfy] oops (*Consistent? Yes*) 
 lemma assumes "⌊(D1 ❙∧ D2w ❙∧ D3n)⌋ ∧ ⌊D4⌋⇩l" shows False nitpick oops (*Inconsistent? No*)
 (* Queries *)
 lemma assumes "⌊(D1 ❙∧ D2w ❙∧ D3n)⌋ ∧ ⌊D4⌋⇩l" shows "⌊❙○<❙¬tell>⌋⇩l" using assms by auto (*Should James not tell? Yes*) 
 lemma assumes "⌊(D1 ❙∧ D2w ❙∧ D3n)⌋ ∧ ⌊D4⌋⇩l" shows "⌊❙○<tell>⌋⇩l"  using assms by blast (*Should James tell? Yes*)
 lemma assumes "⌊(D1 ❙∧ D2w ❙∧ D3n)⌋ ∧ ⌊D4⌋⇩l" shows "⌊❙○<kill>⌋⇩l"  using assms by blast (*Should James kill? Yes*)


(*** Chisholm_D ***)
 (* Narrow-wide scoping is leading to a dependent set of the axioms.*)
 lemma "⌊(D1  ❙∧ D2n ❙∧ D3w) ❙→ D4⌋"   nitpick oops (*countermodel*)
 lemma "⌊(D1  ❙∧ D2n ❙∧ D4)  ❙→ D3w⌋"  by blast  (*proof*)
 lemma "⌊(D1  ❙∧ D3w ❙∧ D4)  ❙→ D2n⌋"  by blast  (*proof*)
 lemma "⌊(D2n ❙∧ D3w ❙∧ D4)  ❙→ D1⌋"   nitpick oops (*countermodel*)
 (* Chisholm_D is thus an inadequate modeling. *)

 (* Consistency *)
 lemma "⌊(D1 ❙∧ D2n ❙∧ D3w)⌋ ∧ ⌊D4⌋⇩l" nitpick [satisfy] oops (*Consistent? Yes*) 
 lemma assumes "⌊(D1 ❙∧ D2n ❙∧ D3w)⌋ ∧ ⌊D4⌋⇩l" shows False nitpick oops (*Inconsistent? No*)
 (* Queries *)
 lemma assumes "⌊(D1 ❙∧ D2n ❙∧ D3w)⌋ ∧ ⌊D4⌋⇩l" shows "⌊❙○<❙¬tell>⌋⇩l" nitpick oops (*Should James not tell? No*) 
 lemma assumes "⌊(D1 ❙∧ D2n ❙∧ D3w)⌋ ∧ ⌊D4⌋⇩l" shows "⌊❙○<tell>⌋⇩l"  using assms by blast (*Should James tell? Yes*)
 lemma assumes "⌊(D1 ❙∧ D2n ❙∧ D3w)⌋ ∧ ⌊D4⌋⇩l" shows "⌊❙○<kill>⌋⇩l"  nitpick oops (*Should James kill? No*)
end
```
