# Supplementary material for: LogiKEy workbench: Deontic logics, logic combinations and expressive ethical and legal reasoning (Isabelle/HOL dataset)
Source: Data Brief. 2020 Oct 15;33:106409. doi: 10.1016/j.dib.2020.106409 (PMC7586073; doi:10.1016/j.dib.2020.106409)
Supplement: Supplementary file 1 [file mmc1.zip › 2020-DataInBrief-Data/CJ_DDL.html]

xml version="1.0" encoding="utf-8"?


Theory CJ\_DDL (Isabelle2019: June 2019)


# Theory CJ\_DDL

theory CJ\_DDL  
imports Main

```
theory CJ_DDL imports Main            (* Christoph Benzmüller & Xavier Parent & Ali Farjami, 2018  *)

begin (* DDL: Dyadic Deontic Logic by Carmo and Jones *)
 typedecl i (*type for possible worlds*)
 type_synonym τ = "(i⇒bool)"
 type_synonym γ = "τ⇒τ" 
 type_synonym ρ = "τ⇒τ⇒τ"

 consts av::"i⇒τ" pv::"i⇒τ" ob::"τ⇒(τ⇒bool)" (*accessibility relations*)
            cw::i (*current world*)

 axiomatization where
  ax_3a: "∀w.∃x. av(w)(x)" and ax_4a: "∀w x. av(w)(x) ⟶ pv(w)(x)" and ax_4b: "∀w. pv(w)(w)" and
  ax_5a: "∀X.¬ob(X)(λx. False)" and
  ax_5b: "∀X Y Z. (∀w. ((Y(w) ∧ X(w)) ⟷ (Z(w) ∧ X(w)))) ⟶ (ob(X)(Y) ⟷ ob(X)(Z))" and
  ax_5ca: "∀X β. ((∀Z. β(Z) ⟶ ob(X)(Z)) ∧ (∃Z. β(Z))) ⟶ 
      (((∃y. ((λw. ∀Z. (β Z) ⟶ (Z w))(y) ∧ X(y))) ⟶ ob(X)(λw. ∀Z. (β Z) ⟶ (Z w))))" and
  ax_5c: "∀X Y Z. (((∃w. (X(w) ∧ Y(w) ∧ Z(w))) ∧  ob(X)(Y)  ∧  ob(X)(Z))  ⟶  ob(X)(λw. Y(w) ∧ Z(w)))" and
  ax_5d: "∀X Y Z. ((∀w. Y(w) ⟶ X(w)) ∧ ob(X)(Y) ∧ (∀w. X(w) ⟶ Z(w)))
                   ⟶ ob(Z)(λw. (Z(w) ∧ ¬X(w)) ∨ Y(w))" and
  ax_5e: "∀X Y Z. ((∀w. Y(w) ⟶ X(w)) ∧ ob(X)(Z) ∧ (∃w. Y(w) ∧ Z(w))) ⟶ ob(Y)(Z)"

 abbreviation ddlneg::γ ("❙¬_"[52]53) where "❙¬A ≡ λw. ¬A(w)" 
 abbreviation ddland::ρ (infixr"❙∧"51) where "A❙∧B ≡ λw. A(w)∧B(w)"   
 abbreviation ddlor::ρ (infixr"❙∨"50) where "A❙∨B ≡ λw. A(w)∨B(w)"   
 abbreviation ddlimp::ρ (infixr"❙→"49) where "A❙→B ≡ λw. A(w)⟶B(w)"  
 abbreviation ddlequiv::ρ (infixr"❙↔"48) where "A❙↔B ≡ λw. A(w)⟷B(w)"  
 abbreviation ddlbox::γ ("❙□") where "❙□A ≡ λw.∀v. A(v)"  (*A = (λw. True)*) 
 abbreviation ddlboxa::γ ("❙□⇩a") where "❙□⇩aA ≡ λw. (∀x. av(w)(x) ⟶ A(x))"  (*in all actual worlds*)
 abbreviation ddlboxp::γ ("❙□⇩p") where "❙□⇩pA ≡ λw. (∀x. pv(w)(x) ⟶ A(x))" (*in all potential worlds*)
 abbreviation ddldia::γ ("❙◇") where "❙◇A ≡ ❙¬❙□(❙¬A)"
 abbreviation ddldiaa::γ ("❙◇⇩a") where "❙◇⇩aA ≡ ❙¬❙□⇩a(❙¬A)"
 abbreviation ddldiap::γ ("❙◇⇩p") where "❙◇⇩pA ≡ ❙¬❙□⇩p(❙¬A)" 
 abbreviation ddlo::ρ ("❙O❙⟨_❙|_❙⟩"[52]53) where "❙O❙⟨B❙|A❙⟩ ≡ λw. ob(A)(B)"  (*it ought to be ψ, given φ *)
 abbreviation ddloa::γ  ("❙O⇩a") where "❙O⇩aA ≡ λw. ob(av(w))(A) ∧ (∃x. av(w)(x) ∧ ¬A(x))" (*actual obligation*)
 abbreviation ddlop::γ  ("❙O⇩p") where "❙O⇩pA ≡ λw. ob(pv(w))(A) ∧ (∃x. pv(w)(x) ∧ ¬A(x))"  (*primary obligation*)
 abbreviation ddltop::τ ("❙⊤") where "❙⊤ ≡ λw. True"
 abbreviation ddlbot::τ ("❙⊥") where "❙⊥ ≡ λw. False"

(*Possibilist Quantification.*)
 abbreviation ddlforall ("❙∀") where "❙∀Φ ≡ λw.∀x. (Φ x w)"
 abbreviation ddlforallB (binder"❙∀"[8]9) where "❙∀x. φ(x) ≡ ❙∀φ"  
 abbreviation ddlexists ("❙∃") where "❙∃Φ ≡ λw.∃x. (Φ x w)"   
 abbreviation ddlexistsB (binder"❙∃"[8]9) where "❙∃x. φ(x) ≡ ❙∃φ" 

 abbreviation ddlvalid::"τ ⇒ bool" ("⌊_⌋"[7]105) where "⌊A⌋ ≡ ∀w. A w"   (*Global validity*)
 abbreviation ddlvalidcw::"τ ⇒ bool" ("⌊_⌋⇩l"[7]105) where "⌊A⌋⇩l ≡ A cw" (*Local validity (in cw)*)

(* A is obliagtory *)
 abbreviation ddlobl::γ ("❙○<_>") where "❙○<A> ≡  ❙O❙⟨A❙|❙⊤❙⟩"  (*New syntax: A is obligatory.*)

(* Consistency *) 
 lemma True nitpick [satisfy,user_axioms,show_all] oops 
end
```
