# Supplementary material for: LogiKEy workbench: Deontic logics, logic combinations and expressive ethical and legal reasoning (Isabelle/HOL dataset)
Source: Data Brief. 2020 Oct 15;33:106409. doi: 10.1016/j.dib.2020.106409 (PMC7586073; doi:10.1016/j.dib.2020.106409)
Supplement: Supplementary file 1 [file mmc1.zip › 2020-DataInBrief-Data/Course-Material-1/Deontic.pdf]

Intelligent Systems II

Contents

1 Introduction to Isabelle **2**

1.1 Basics 2

1.2 Exercises 4

2 Proving first theorems **5**

2.1 Hilbert Calculus for Classical Logic 5

2.2 Exercises 6

3 Hilbert proofs for MDL **7**

3.1 MDL Proof Examples 7

3.2 Exercises 7

4 MDL Reasoning – A Semantical Approach **8**

4.1 Some properties of MDL 9

4.2 Chisholm's Paradox in MDL 10

5 Forrester's Paradox **12**

6 About using Cups. **14**

1 Introduction to Isabelle

1.1 Basics

Text blocks for in-line documentation (in a way related to literal programming) are started with the **text** command, followed by the text to write in the ⟨ ... ⟩ brackets. You can also structure the document using commands such as **section** or **subsection** etc., just like in Latex.

Comments (like in programming languages) are written in Isabelle between (* and *). As an example, the following comment will not be interpreted by Isabelle in any sense:

See? Probably you did not see anything, since comments are also not processed by the PDF generation. Visible in-line comments can be written using the comment symbol — after some term or formula (in the same line), like this:

term a — Some atomic term variable

We just wrote down a term symbol a and put an in-line comment right after it.

Speaking of terms, we will now introduce terms, formulas, types, etc. that you will need for using Isabelle.

Terms We can write logical formulae and terms in the usual notation. Connectives such as $\neg$, $\vee$, $\wedge$ etc. can be typed using the backslash \ followed by the name of the sign. I.e. $\backslash$*not* for $\neg$. Note that during typing $\backslash$*not* at some point there will be a pop-up menu offering you certain auto completion suggestions that you can accept by pressing the tab key.

Some examples:

term a — atomic term as above
term $a \wedge b$ — conjunction
term $a \Longrightarrow b$ — material implication
term $\forall x.\ p\ x$ — universal quantification

In higher-order logic, formulas are just terms of a specific type, called *bool* in Isabelle. Every term above can also be seens as a formula:

prop a — atomic formula
prop $a \wedge b$ — conjunction
prop $a \Longrightarrow b$ — material implication
prop $\forall x.\ p\ x$ — universal quantification

By using the **prop** command, we can tell Isabelle to interpret the input as a formula instead of any term. In the case of the last three terms, this does not

make any difference since we use Boolean connectives. So Isabelle will figure itself out, that the symbols a, b are actually atomic formulas. However, this is not necessarily true for the first example. The symbol a could be of any type. So Isabelle will not fix its type, but instead give it a type placeholder (referred to as "type variables").

Types All terms (and also constant symbols, variables etc.) are associated a type. The type *bool* is the type of all Boolean-values objects (e.g. truth values and formulas). There are many pre-defined types in Isabelle, e.g. for sets, functions, numbers, etc. We will mainly focus on formulas and possibly individuals (objects from the universe of discourse). A type for individuals, often denoted i, does not exist in Isabelle yet. New types can be inserted at will using the **typedecl** command:

typedecl i — Create a new type i for the type of individuals

We can now play a bit around with types. The **typ** command allows us to write down types, just to try it out (as for **term**, writing down types does not change the state of the document. It is just a more sophisticated way of writing text for now). Function types are written using the function type constructor $\Rightarrow$.

typ $i \Rightarrow i$ — The type of functions from objects of type i to objects of type i

typ $i \Rightarrow bool$ — The type of a predicate on objects of type i

Terms can be assigned a specific type by the user. This is done using the :: command, written is postfix notation. This can be useful if you want to restrict the type of a term, a constant symbol or a variable and hence forbid Isabelle to figure the most general type on its own.

term a — a is a variable of some type
term $a :: i$ — a is a variable of type i
term $p :: i \Rightarrow bool$ — p is a predicate

Constants New atomic symbols can be defined using the **consts** keyword. You need to specify the type of the constant explicitly, using the :: operator, just like above:

consts $a :: bool$
$b :: bool$

You can list as many constant symbols as you want. Note that this will change the state of the system (unlike commands such as **typ**, **term** and **prop** that just allowed us to scribble a little bit around.

From now on, there exist atomic symbols (atomic propositions) a and b.

Using the constants above, we can write down more complex formulas:

prop $a \wedge b$
prop $(a \wedge b) \longleftrightarrow (\neg(\neg a \vee \neg b))$
prop $\neg\neg a$

1.2 Exercises

Exercise 1. Before we start proving logical formulae, we become acquainted with the basic logical connectives, the quanti- fiers and the remaining components of a logical formula. To that end, please give appropriate formalizations of the following expressions stated in natural language. You may freely choose appropriate names for variables and further identifiers.

- "The ship is huge and it is blue."
- "I'm sad if the sun does not shine."
- "Either it's raining or it is not."
- "I'm only going if she is going!"
- "Everyone loves chocolate or ice cream."
- "There is somebody who loves ice cream and loves chocolate as well."
- "Everyone has got someone to play with."
- "Nobody has somebody to play with if they are all mean."
- "Cats have the same annoying properties as dogs."

Solutions to Ex. 1

- "The ship is huge and it is blue."

Introduce constants for ship, huge and blue of appropriate type:

consts *ship* :: *i*
huge :: $i \Rightarrow$ *bool*
blue :: $i \Rightarrow$ *bool*

Now we can express the sentence as

prop ⟨*huge ship* $\wedge$ *blue ship*⟩

- ...

2 Proving first theorems

2.1 Hilbert Calculus for Classical Logic

Technical remark at the beginning: In order to distinguish our connectives from the usual logical connectives of the Isabelle/HOL system, we use **bold**-face written versions of them. You may use the abbreviations at the top of the document (in the theory file) to write things down, e.g. if you start writing "dis..." (the abbreviation is "disj"), Isabelle should suggest the autocompletion for $\vee$, etc.

typedecl σ — Introduce new type for syntactical formulae (propositions).

For our classical propositional language, we introduce two primitive symbols: Implication and negation. The others can be defined in terms of these two.

consts *PLimpl* :: $\sigma \Rightarrow \sigma \Rightarrow \sigma$ (**infixr** $\rightarrow$ *49*)
PLnot :: $\sigma \Rightarrow \sigma$ ($\neg$- [*52*]*53*)

consts *PLatomicProp* :: σ

definition *PLdisj* :: $\sigma \Rightarrow \sigma \Rightarrow \sigma$ (**infixr** $\vee$ *50*) **where** $a \vee b \equiv \neg a \rightarrow b$
definition *PLconj* :: $\sigma \Rightarrow \sigma \Rightarrow \sigma$ (**infixr** $\wedge$ *51*) **where** $a \wedge b \equiv \neg(\neg a \vee \neg b)$
definition *PLequi* :: $\sigma \Rightarrow \sigma \Rightarrow \sigma$ (**infix** $\leftrightarrow$ *48*) **where** $a \leftrightarrow b \equiv (a \rightarrow b) \wedge (b \rightarrow a)$
definition *PLtop* :: σ ($\top$) **where** $\top \equiv$ *PLatomicProp* $\vee \neg$*PLatomicProp*
definition *PLbot* :: σ ($\bot$) **where** $\bot \equiv \neg\top$

Next we define the notion of syntactical derivability and consequence:

consts *derivable* :: $\sigma \Rightarrow$ *bool* ($\vdash$ - *40*)
definition *consequence* :: $\sigma \Rightarrow \sigma \Rightarrow$ *bool* (- $\vdash$ - *40*) **where**
$A \vdash B \equiv\ \vdash (A \rightarrow B)$

We can now axiomatize the derivability relation using a Hilbert-style system:

axiomatization where
A2: $\vdash A \rightarrow (B \rightarrow A)$ **and**
A3: $\vdash (A \rightarrow (B \rightarrow C)) \rightarrow ((A \rightarrow B) \rightarrow (A \rightarrow C))$ **and**
A4: $\vdash (\neg A \rightarrow \neg B) \rightarrow (B \rightarrow A)$ **and**
MP: $\vdash (A \rightarrow B) \Longrightarrow\ \vdash A \Longrightarrow\ \vdash B$

Proof example. Now we are ready to proof first simple theorems:

lemma $\vdash A \rightarrow A$
proof –
have *1*: $\vdash (A \rightarrow ((B \rightarrow A) \rightarrow A)) \rightarrow ((A \rightarrow (B \rightarrow A)) \rightarrow (A \rightarrow A))$ **by** (*rule A3*[*of* - $B \rightarrow A$ *A*])
have *2*: $\vdash A \rightarrow ((B \rightarrow A) \rightarrow A)$ **by** (*rule A2*[*of* - $B \rightarrow A$])
from *1 2* **have** *3*: $\vdash (A \rightarrow (B \rightarrow A)) \rightarrow (A \rightarrow A)$ **by** (*rule MP*)
have *4*: $\vdash A \rightarrow (B \rightarrow A)$ **by** (*rule A2*[*of* - -])
from *3 4* **have** $\vdash A \rightarrow A$ **by** (*rule MP*)

then show *?thesis* **.**
qed

The same proof a little bit nicer with syntactic sugar:

lemma $\vdash A \rightarrow A$
proof –
have $\vdash (A \rightarrow ((B \rightarrow A) \rightarrow A)) \rightarrow ((A \rightarrow (B \rightarrow A)) \rightarrow (A \rightarrow A))$ **by** (*rule A3*[*of* - $B \rightarrow A$ A])
moreover have $\vdash A \rightarrow ((B \rightarrow A) \rightarrow A)$ **by** (*rule A2*[*of* - $B \rightarrow A$])
ultimately have $\vdash (A \rightarrow (B \rightarrow A)) \rightarrow (A \rightarrow A)$ **by** (*rule MP*)
moreover have $\vdash A \rightarrow (B \rightarrow A)$ **by** (*rule A2*[*of* - -])
ultimately show $\vdash A \rightarrow A$ **by** (*rule MP*)
qed

2.2 Exercises

Exercise 2. Prove one of the following statements by giving an explicit proof within the given Hilbert calculus. Please make sure that every inference step in your proof is fine-grained and annotated with the respective calculus rule name.

Hint: Find a proof first by pen-and-paper work and then formalize it within Isabelle.

- $\vdash \neg(F \rightarrow F) \rightarrow G$
- $\vdash \neg\neg F \rightarrow F$

We will later see that many proofs can in fact be done almost automatically by Isabelle, see e.g. the example from further above:

lemma *PL1*: $\vdash A \rightarrow A$ **using** *A2 A3 MP* **by** *blast*
lemma *PL2*: $\vdash \neg(F \rightarrow F) \rightarrow G$ **by** (*metis A2 A3 A4 MP*)
lemma *PL3*: $\vdash \neg\neg F \rightarrow F$ **by** (*metis A2 A3 A4 MP*)

Also, to make things look a bit nicer, we define a compound strategy *PL* (for "Propositional Logic") that applies an internal automated theorem prover with the respective axioms for propositional logic (A2, A3, A4 and MP)

method *PL* **declares** *add* = (*metis A2 A3 A4 MP add*)

In the following, we can simply use *by PL* for proving propositional tautologies. However, as proofs can be arbitrarily complicated, this method may fail for difficult formulas.

3 Hilbert proofs for MDL

We augment the language PL with the new connectives of MDL:

consts *ob* :: $\sigma \Rightarrow \sigma$ ($\bigcirc$- *[52]53*) — New atomic connective for obligation

The other new deontic logic connective can be defined as usual:

definition *perm* :: $\sigma \Rightarrow \sigma$ (**P**- *[52]53*) **where P** $a \equiv \neg(\bigcirc(\neg a))$
definition *forbidden* :: $\sigma \Rightarrow \sigma$ (**F**- *[52]53*) **where F** $a \equiv \bigcirc(\neg a)$

Next, we additionally augment the axiomatization of our proof system for PL with the rules of the system D for MDL:

axiomatization where
K: $\vdash \bigcirc(A \to B) \to (\bigcirc A \to \bigcirc B)$ **and**
D: $\vdash \bigcirc A \to \mathbf{P}A$ **and**
NEC: $\vdash A \Longrightarrow \vdash (\bigcirc A)$

3.1 MDL Proof Examples

lemma $\vdash \bigcirc(p \wedge q) \to \bigcirc p$
proof –
have *1*: $\vdash (p \wedge q) \to p$ **by** (*PL add*: *PLconj-def PLdisj-def*)
from *1* **have** *2*: $\vdash \bigcirc((p \wedge q) \to p)$ **by** (*rule NEC*)
have *3*: $\vdash \bigcirc((p \wedge q) \to p) \to \bigcirc(p \wedge q) \to \bigcirc p$ **by** (*rule K*)
from *3 2* **show** $\vdash \bigcirc(p \wedge q) \to \bigcirc p$ **by** (*rule MP*)
qed

Note that, although we have the necessitation rule $\vdash\ ?A \Longrightarrow\ \vdash \bigcirc ?A$, the formula $A \to \bigcirc A$ is not generally valid:

lemma $\vdash a \to \bigcirc a$ **nitpick**[*user-axioms*,*expect=genuine*] **oops**

3.2 Exercises

Exercise 2. Prove the following statement by giving an explicit proof within the given Hilbert calculus. Please make sure that every inference step in your proof is fine-grained and annotated with the respective calculus rule name. You may use the general *PL* method for inferring propositional tautologies.

Hint: Reuse your proof from the previous exercise sheet and formalize it within Isabelle.

- $\vdash \neg\bigcirc\bot$

4 MDL Reasoning – A Semantical Approach

Direct automation of proof calculi such as natural deduction (ND) or Hilbert-style systems is usually unfeasible in practice. The calculi do not provide reasonable proof guidance for theorem proving systems and, thus, perform poorly. Popular calculi well-suited for automation are resolution, superposition, tableaux-based systems, connection calculi and many more. However, implementing such calculi is hard work and proving them correct can also be a large task.

Another approach is to utilize the expressivity of Isabelle's underlying higher-order logic (HOL) to encode MDL's semantics into HOL and then use already existing automation methods for HOL for reasoning within MDL.

The embedding explicitly encodes the Kripke-style semantics of MDL (Modal logic D) as follows:

typedecl i — type for possible worlds
type-synonym $\sigma = (i \Rightarrow bool)$ — propositions are lifted to predicates on worlds

locale $MDL =$
  fixes
    $r :: i \Rightarrow i \Rightarrow bool$ (**infixr** r 70) — the accessibility relation
  assumes
    $seriality$: $\forall w.\ \exists v.\ w\ r\ v$ — the usual assumption for MDL
begin

In the following, the definitions of all logical connectives are given with respect to their semantics.

abbreviation $mnot :: \sigma \Rightarrow \sigma$ ($\neg$-[52]53)
 where $\neg\varphi \equiv \lambda w.\ \neg\varphi(w)$
abbreviation $mand :: \sigma \Rightarrow \sigma \Rightarrow \sigma$ (**infixr**$\wedge 51$)
 where $\varphi \wedge \psi \equiv \lambda w.\ \varphi(w) \wedge \psi(w)$
abbreviation $mor :: \sigma \Rightarrow \sigma \Rightarrow \sigma$ (**infixr**$\vee 50$)
 where $\varphi \vee \psi \equiv \lambda w.\ \varphi(w) \vee \psi(w)$
abbreviation $mimp :: \sigma \Rightarrow \sigma \Rightarrow \sigma$ (**infixr**$\rightarrow 49$)
 where $\varphi \rightarrow \psi \equiv \lambda w.\ \varphi(w) \longrightarrow \psi(w)$
abbreviation $mequ :: \sigma \Rightarrow \sigma \Rightarrow \sigma$ (**infixr**$\equiv 48$)
 where $\varphi \equiv \psi \equiv \lambda w.\ \varphi(w) \longleftrightarrow \psi(w)$
abbreviation $mobligatory :: \sigma \Rightarrow \sigma$ ($\bigcirc$)
 where $\bigcirc\ \varphi \equiv \lambda w.\ \forall v.\ \ w\ r\ v \longrightarrow \varphi(v)$
abbreviation $mforbidden :: \sigma \Rightarrow \sigma$ (F)
 where $F\ \varphi \equiv \bigcirc\ (\neg\ \varphi)$
abbreviation $mpermitted :: \sigma \Rightarrow \sigma$ (P)
 where $P\ \varphi \equiv \neg\ (\bigcirc\ (\neg\ \varphi))$

The meta-logical notion valid is used to ground formulas of MDL to truth-values within HOL (i.e. Isabelle). It's definition is straight-forward: A MDL formula is (globally) valid iff it holds in every possible world.

abbreviation *valid* :: $\sigma \Rightarrow$ *bool* ($\lfloor$-$\rfloor$[7]*8*)
where $\lfloor p \rfloor \equiv \forall w.\ p\ w$

We can also define local validity as validity in a certain given world w:

abbreviation *validInCW* :: $\sigma \Rightarrow i \Rightarrow$ *bool* ($\lfloor$-$\rfloor$-[7]*8*)
where $\lfloor p \rfloor_w \equiv p\ w$
end

Now we already have everything we need for reasoning within MDL. Using the definitions/abbreviations above, formulas that are inputted are being internally unfolded to HOL formulas and we can use every proof strategy of Isabelle to tackle them.

4.1 Some properties of MDL

We can easily verify the basic properties of MDL:

lemma (**in** *MDL*) *D*:
$\lfloor \neg\ ((\bigcirc\varphi) \wedge (\bigcirc(\neg\varphi))) \rfloor$ **using** *seriality* **by** *smt*
lemma (**in** *MDL*) *K*: $\lfloor ((\bigcirc\varphi) \wedge (\bigcirc(\varphi \rightarrow \psi)) \rightarrow (\bigcirc\psi)) \rfloor$ **by** *simp*
lemma (**in** *MDL*) *NEC*: $\lfloor \varphi \rfloor \Longrightarrow \lfloor \bigcirc\varphi \rfloor$ **by** *simp* — $\Longrightarrow$ is meta-level implication
lemma (**in** *MDL*) *RM*: $\lfloor \varphi \rightarrow \psi \rfloor \Longrightarrow \lfloor (\bigcirc\varphi) \rightarrow (\bigcirc\psi) \rfloor$ **by** *simp*

We can also reason about MDL in this setting, e.g. for verifying that the axiom D corresponds to serial frames:

lemma (**in** *MDL*) *D-equivalent-to-seriality*:
$\lfloor \neg\ ((\bigcirc\varphi) \wedge (\bigcirc(\neg\varphi))) \rfloor \equiv (\forall w.\ \exists v.\ w\ r\ v)$ **by** *smt*

We can check for consistency of our theory at any time:

lemma (**in** *MDL*) *True* **nitpick** [*satisfy*,*user-axioms*,*expect=genuine*] **oops**
— Consistency is confirmed by Nitpick

And we can get explicit (counter-) models generated for formulas that do not hold in general.

lemma (**in** *MDL*) *MC*: $\lfloor \varphi \rightarrow (\bigcirc\varphi) \rfloor$ — Modal Collapse
nitpick [*user-axioms*,*expect=genuine*] **oops**
— Counter model by Nitpick, that is, Modal Collapse for O does not hold

Examples from the exercises We define short hands for the both relation properties:

abbreviation *transitive*
where *transitive* $R \equiv \forall w\ v\ u.\ ((R\ w\ v) \wedge (R\ v\ u)) \longrightarrow R\ w\ u$
abbreviation *euclidean*
where *euclidean* $R \equiv \forall\ s\ t\ u.\ ((R\ s\ t) \wedge (R\ s\ u)) \longrightarrow R\ t\ u$

Exercise 1 Does it hold that the formula $\bigcirc\ \varphi \rightarrow \bigcirc(\bigcirc\ \varphi)$ is valid if r is not transitive?

lemma (**in** *MDL*)
  assumes ¬ (*transitive* (*r*))
  shows $\lfloor\bigcirc\ \varphi \rightarrow \bigcirc(\bigcirc\ \varphi)\rfloor$
  nitpick[*expect=genuine*, *atoms = s t*, *card i = 2*]
  oops

No, there is a counter-model provided by nitpick. It The counter model can directly be reconstructed from nitpick's answer.

Exercise 2 Prove that $\bigcirc\ \varphi \rightarrow \bigcirc\ \psi$ whenever $\varphi \rightarrow \psi$.

lemma (**in** *MDL*) *ROM*: $\lfloor\varphi \rightarrow \psi\rfloor \Longrightarrow \lfloor\bigcirc\ \varphi \rightarrow \bigcirc\ \psi\rfloor$ **by** *simp*

Exercise 3 Does it hold that the formula $\neg\bigcirc\ \varphi \rightarrow \bigcirc(\neg\bigcirc\ \varphi)$ is valid if r is euclidean?

lemma (**in** *MDL*) *3a*:
  assumes *euclidean* (*r*)
  shows $\lfloor\neg\bigcirc\ \varphi \rightarrow \bigcirc(\neg\bigcirc\ \varphi)\rfloor$
  nitpick[*expect=none*]
  sledgehammer
  using *assms* **by** *blast*

Yes, nitpick does not find a counter-model and sledgehammer suggests a proof in under 5ms.

lemma (**in** *MDL*) *3b*:
  assumes ¬ *euclidean* (*r*)
  shows $\lfloor\neg\bigcirc\ \varphi \rightarrow \bigcirc(\neg\bigcirc\ \varphi)\rfloor$
  nitpick[*expect=genuine*,*atoms = s t*]
  oops

On the contrary, if r is not euclidean, then the conjecture does not hold any more. Nitpick gives a minimal counter-model with two worlds s and t.

4.2 Chisholm's Paradox in MDL

Let's use the automation of MDL to assess Chisholm's paradox with automated reasoning mechanisms:

The set-up. We define a locale that gives raise to the vocabulary (and possibly additional assumptions) of an experiment scenario.

locale *Chisholm* = *MDL* +
  fixes — We fix local assumptions to our experiment.
    go :: σ **and** — both from Chisholm's paradox

tell :: σ **and**
kill :: σ **and**
w :: *i* — current world
begin

Define the axioms of the paradox as shorthands D1, D2, D3 and D4, and, for D2 and D3, both the wide and narrow interpretation.

It is obligatory to go and help.

abbreviation *D1* **where** $D1 \equiv \lfloor \bigcirc\ go \rfloor_w$ — everything local

If you go, you must tell your neighbor.

abbreviation *D2-wide* ($D2^w$) **where** $D2^w \equiv \lfloor \bigcirc\ (go \rightarrow tell) \rfloor_w$
abbreviation *D2-narrow* ($D2^n$) **where** $D2^n \equiv \lfloor go \rightarrow \bigcirc tell \rfloor_w$

If you do not go, do not tell your neighbor.

abbreviation *D3-wide* ($D3^w$) **where** $D3^w \equiv \lfloor \bigcirc(\ \neg go \rightarrow \neg tell\ ) \rfloor_w$
abbreviation *D3-narrow* ($D3^n$) **where** $D3^n \equiv \lfloor (\neg go) \rightarrow (\bigcirc\ (\neg tell)) \rfloor_w$

You do not go.

abbreviation *D4* **where** $D4 \equiv \lfloor \neg\ go \rfloor_w$

As can be seen above, we have defined two variants of D2 and D3, referred to as narrow- and wide-scope interpretations. We select the concrete D2/D3 by ...

abbreviation *D2* **where** $D2 \equiv D2^w$
abbreviation *D3* **where** $D3 \equiv D3^n$

We can access the definitions directly or just change to above two lines if we want to change the interpretations for our experiments.

You should kill the boss. Just as an example statement that we really do not want to be true.

abbreviation *D5* **where** $D5 \equiv \lfloor \bigcirc\ kill \rfloor_w$
end

The experiments. Now we can start assessing the concrete examples:

Inconsistency. We can prove falsity from the assumptions D1, D2, D3 and D4.

theorem (**in** *Chisholm*)
  assumes *D1* **and** *D2* **and** *D3* **and** *D4*
  shows *False*
using *seriality assms* **by** *fastforce*

This implies that the assumptions are inconsistent and we can prove anything, e.g. that we should kill our Boss:

theorem (**in** *Chisholm*)
  assumes *D1* **and** *D2* **and** *D3* **and** *D4*
  shows *D5*
  sledgehammer
  using *assms seriality* **by** *blast*

Sledgehammer finds a proof for this in less than 5ms.

Logical independence We can also assess logical independence. Depending on the choice of narrow/wide scope interpretations, either the theory will be inconsistent or logically dependent.

lemma (**in** *Chisholm*)
  assumes *D2* **and** *D3* **and** *D4*
  shows *D1* **nitpick**[*user-axioms*, *expect=genuine*, *show-all*] **oops**

lemma (**in** *Chisholm*)
  assumes *D1* **and** *D3* **and** *D4*
  shows *D2* **nitpick**[*user-axioms*, *expect=genuine*, *show-all*] **oops**

lemma (**in** *Chisholm*)
  assumes *D1* **and** *D2* **and** *D4*
  shows *D3* **nitpick**[*user-axioms*, *expect=genuine*, *show-all*] **oops**

lemma (**in** *Chisholm*)
  assumes *D1* **and** *D2* **and** *D3*
  shows *D4* **nitpick**[*user-axioms*, *expect=genuine*, *show-all*] **oops**

Currently, the theory is logically independent (but inconsistent), as certified by nitpick (giving counter-models to the conjecture of logical dependence). These results of course change if D2 and D3 are chosen with other scope.

5 Forrester's Paradox

Just as above, we define a local scope for the experiments with Forrester's paradox:

locale *Forrester* = *MDL* +
  fixes
    red :: σ **and**
    scarlet :: σ **and** — both from Forrester's paradox
    w :: *i* — current world
  assumes
    scarlet-implies-red: $\lfloor scarlet \rightarrow red \rfloor$
begin

You ought not dress red.

abbreviation *F1*
  where $F1 \equiv \lfloor \bigcirc(\neg red) \rfloor_w$

If you dress red, you ought dress scarlet

abbreviation *F2-narrow* ($F2^n$)
  where $F2^n \equiv \lfloor (red \rightarrow \bigcirc scarlet) \rfloor_w$
abbreviation *F2-wide* ($F2^w$)
  where $F2^w \equiv \lfloor \bigcirc(red \rightarrow scarlet) \rfloor_w$

You dress red.

abbreviation *F3*
  where $F3 \equiv \lfloor red \rfloor_w$

abbreviation *F2*
  where $F2 \equiv F2^n$
end

lemma (**in** *Forrester*)
  assumes *F1* **and** *F2* **and** *F3*
  shows *False*
  nitpick
  using *assms scarlet-implies-red seriality* **by** *blast*

Again, the theory is inconsistent.

Logical independence can again be assessed for different scope interpretations:

lemma (**in** *Forrester*)
  assumes *F1* **and** *F2*
  shows *F3*
  nitpick oops

lemma (**in** *Forrester*)
  assumes *F1* **and** *F3*
  shows *F2*
  nitpick oops

lemma (**in** *Forrester*)
  assumes *F2* **and** *F3*
  shows *F1*
  nitpick oops

6 About using Cups.

locale *Cup* = *MDL* +
fixes — We fix local assumptions to our experiment.
useCup :: σ **and** — Using the cup
putInDishwasher :: σ **and** — Putting the cup in the dishwasher
w :: *i* — current world

assumes $\lfloor$ *putInDishwasher* $\rightarrow$ *useCup* $\rfloor$ — Implicit: If I put a cup in the dishwasher; I'm practically using it.
begin

You ought not use non-stock cups.

abbreviation *F1* **where** $F1 \equiv \lfloor \bigcirc(\neg useCup) \rfloor$

If you use non-stock cups, you ought to put them into the dishwasher

abbreviation *F2* (*F2*) **where** $F2 \equiv \lfloor (useCup \rightarrow \bigcirc putInDishwasher) \rfloor$

I use a non-stock cup.

abbreviation *F3* **where** $F3 \equiv \lfloor useCup \rfloor_w$

lemma
assumes *F1* **and** *F2* **and** *F3*
shows *False*
using *Cup.axioms*(*2*) *Cup-axioms Cup-axioms-def D assms*(*1*) *assms*(*2*) *assms*(*3*)
by *fastforce*

As a consequence, we see that we get in a crazy situation (Falsity is true!) if using non-stock cups.

end
