# Supplementary material for: LogiKEy workbench: Deontic logics, logic combinations and expressive ethical and legal reasoning (Isabelle/HOL dataset)
Source: Data Brief. 2020 Oct 15;33:106409. doi: 10.1016/j.dib.2020.106409 (PMC7586073; doi:10.1016/j.dib.2020.106409)
Supplement: Supplementary file 1 [file mmc1.zip › 2020-DataInBrief-Data/IOL_out2.html]

xml version="1.0" encoding="utf-8"?


Theory IOL\_out2 (Isabelle2019: June 2019)


# Theory IOL\_out2

theory IOL\_out2  
imports Main

```
theory IOL_out2 imports Main      
begin
  typedecl i ― ‹type for possible worlds ›  
  type_synonym τ = "(i⇒bool)"
  consts r :: "i⇒i⇒bool" (infixr "r" 70)(* relation for a modal logic K *)
  abbreviation knot :: "τ⇒τ" ("❙¬_"[52]53) where "❙¬φ ≡ λw. ¬φ(w) " 
  abbreviation kor :: "τ⇒τ⇒τ" (infixr "❙∨"50) where "φ❙∨ψ ≡ λw. φ(w) ∨ ψ(w)" 
  abbreviation kand :: "τ⇒τ⇒τ" (infixr "❙∧"51) where "φ❙∧ψ ≡ λw. φ(w) ∧ ψ(w)" 
  abbreviation kimp :: "τ⇒τ⇒τ" (infixr "❙⟶" 49) where "φ❙⟶ψ ≡ λw. φ(w) ⟶ ψ(w)"
  abbreviation kvalid :: "τ⇒bool" ("⌊_⌋" [8]109) where "⌊p⌋ ≡ ∀w. p(w)"
  abbreviation kbox :: "τ⇒τ" ("❙□⇩k") where " ❙□⇩k φ ≡ λw. ∀v. w r v ⟶ φ(v)"
  abbreviation kdiamond :: "τ⇒τ" ("❙◇⇩k") where " ❙◇⇩k φ ≡ λw. ∃v. w r v ⟶ φ(v)"
  abbreviation ktrue  :: "τ" ("❙⊤") where "❙⊤ ≡ λw. True" 
  abbreviation kfalse :: "τ" ("❙⊥") where "❙⊥ ≡ λw. False" 

(*Idea:  x ∈ out2(G,A) iff G⇧□∪A ⊢⇩K❙□x ∧ x ∈ Cn(G(L))  *)  
  
locale TestExample =        (* OR example:  G = {(a,e),(b,e)}, e ∈ out2(G,{a ∨ b}) *)
  fixes a::τ and b::τ and e::τ  
 begin
 (* G⇧□∪{a ∨ b} ⊢⇩K □e *)
 lemma "⌊((a ❙⟶❙□⇩ke) ❙∧ (b❙⟶❙□⇩ke) ❙∧ (a ❙∨ b)) ❙⟶ ❙□⇩ke  ⌋" by auto
 (* e ∈ Cn(G(L)) *)
 lemma " ⌊ (e ❙∧ e) ❙⟶ e ⌋"  by simp 
 end

(*Drink and Drive Example*)
datatype indiv= Ali | Paul| Child
consts  Kill::"indiv⇒τ" Hurt::"indiv⇒τ" Drive_carefully::"indiv⇒τ" Stay::"indiv⇒τ"
               Drunk::"indiv⇒τ" Jump::"indiv⇒τ" Drive::"indiv⇒τ"

locale PaulContext =
  assumes
  (* Norms *)
  A0: "⌊ (❙⊤ ❙⟶ ❙□⇩k (❙¬ Kill Child ❙∧ ❙¬ Hurt Child )) ⌋" and
  A1: "⌊  (❙⊤ ❙⟶ ❙□⇩k ( Drive_carefully Paul ) )⌋" and
  A2: "⌊( (❙¬Drive_carefully Paul) ❙⟶❙□⇩k(Stay Paul ) ) ⌋"and
  (* Input set *) 
  A3: "⌊Drunk Paul⌋" and
  A4: "⌊Drive Paul⌋"  and
  A5: "⌊Jump Child⌋" and
  A6: "⌊Drunk Paul ❙⟶ ❙¬ Drive_carefully Paul⌋" and
  A7:"⌊((❙¬Drive_carefully Paul) ❙∧ Drive Paul ❙∧ Jump Child )❙⟶ ((Kill Child ❙∨ Hurt Child))⌋"
  begin
  lemma "⌊❙□⇩k(Stay Paul)⌋"   using A2 A3 A6 by auto 
  lemma "⌊❙□⇩k(Drive_carefully Paul)❙∧(❙¬Drive_carefully Paul) ⌋"  using A1 A3 A6 by simp
  lemma "⌊❙□⇩k(❙¬Kill Child ❙∧ ❙¬Hurt Child ) ❙∧ (Kill Child ❙∨ Hurt Child) ⌋"  using A0 A3 A4 A5 A6 A7 by simp
  lemma True  nitpick [satisfy,user_axioms,show_all,expect=genuine] oops (*consistency*)
  end

locale AliContext =
  assumes
  (* Norms *)
  A0: "⌊❙⊤ ❙⟶ ❙□⇩k(❙¬ Kill Child ❙∧ ❙¬ Hurt Child)⌋" and
  A1: "⌊❙⊤ ❙⟶ ❙□⇩k(Drive_carefully Ali)⌋" and
  A2: "⌊❙¬ Drive_carefully Ali ❙⟶ ❙□⇩k(Stay Ali)⌋"and
  (* Input set *)
  A3: "⌊Drunk Ali⌋" and
  A4: "⌊Drive Ali⌋" and
  A6: "⌊Drunk Ali ❙⟶ ❙¬ Drive_carefully Ali⌋" and
  A7: "⌊(❙¬ Drive_carefully Ali ❙∧ Drive Ali ❙∧ Jump Child) ❙⟶ (Kill Child ❙∨ Hurt Child)⌋"
  begin
  lemma "⌊❙□⇩k(Stay Ali)⌋"   using A2 A3 A6 by simp
  lemma "⌊❙□⇩k(Drive_carefully Ali) ❙∧ ❙¬ Drive_carefully Ali⌋"  using A1 A3 A6 by simp
  lemma"⌊❙□⇩k(❙¬Kill Child ❙∧ ❙¬Hurt Child) ❙∧ (Kill Child ❙∨ Hurt Child)⌋" nitpick [user_axioms] oops (*countermodel*)
  lemma True  nitpick [satisfy,user_axioms,show_all,expect=genuine] oops (*consistency*)
  end

end
```
