# Supplementary figures and images for: LogiKEy workbench: Deontic logics, logic combinations and expressive ethical and legal reasoning (Isabelle/HOL dataset)
Source: Data Brief. 2020 Oct 15;33:106409. doi: 10.1016/j.dib.2020.106409 (PMC7586073; doi:10.1016/j.dib.2020.106409)

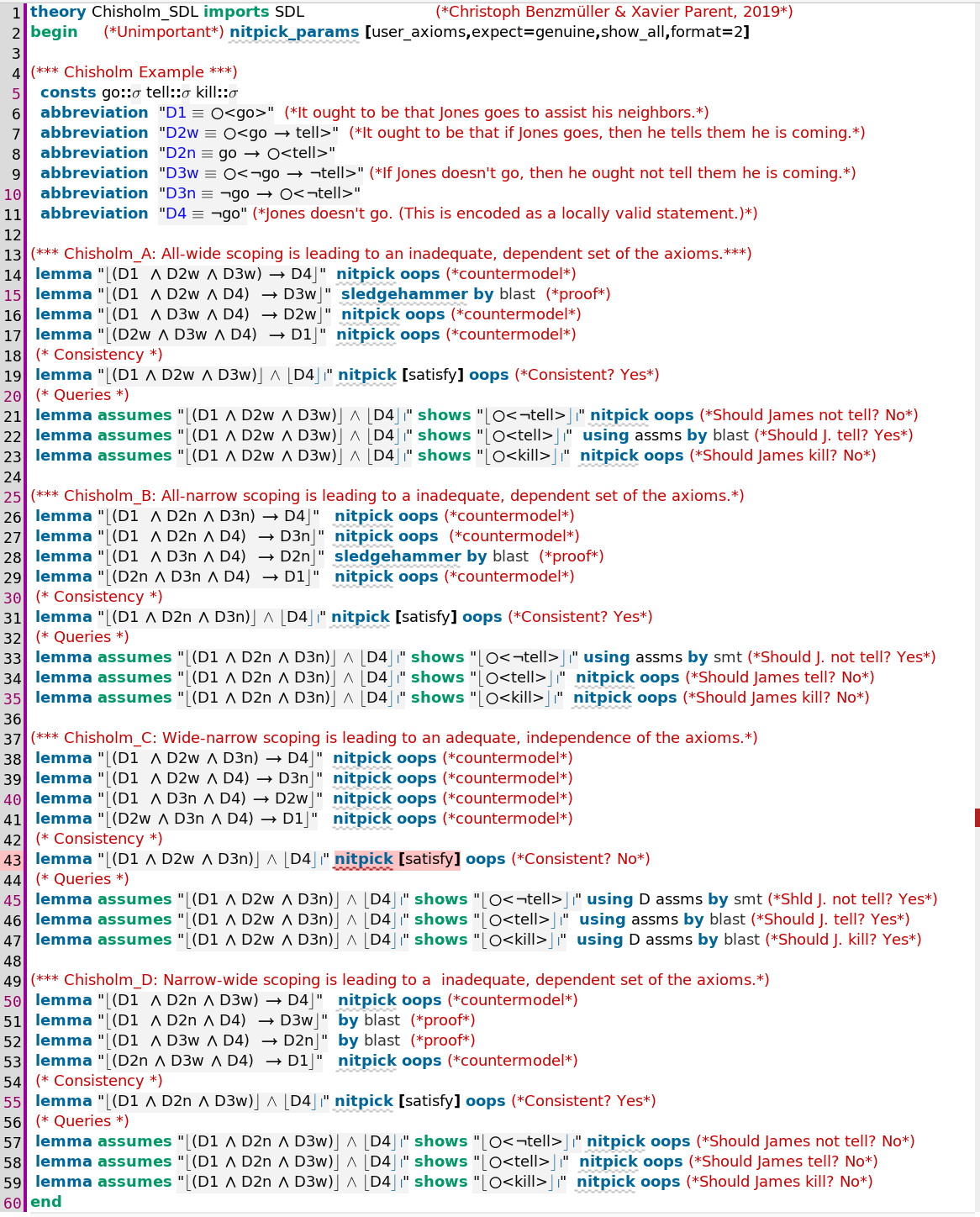

Supplement: Supplementary file 1 [file mmc1.zip › 2020-DataInBrief-Data/Chisholm_SDL.png]

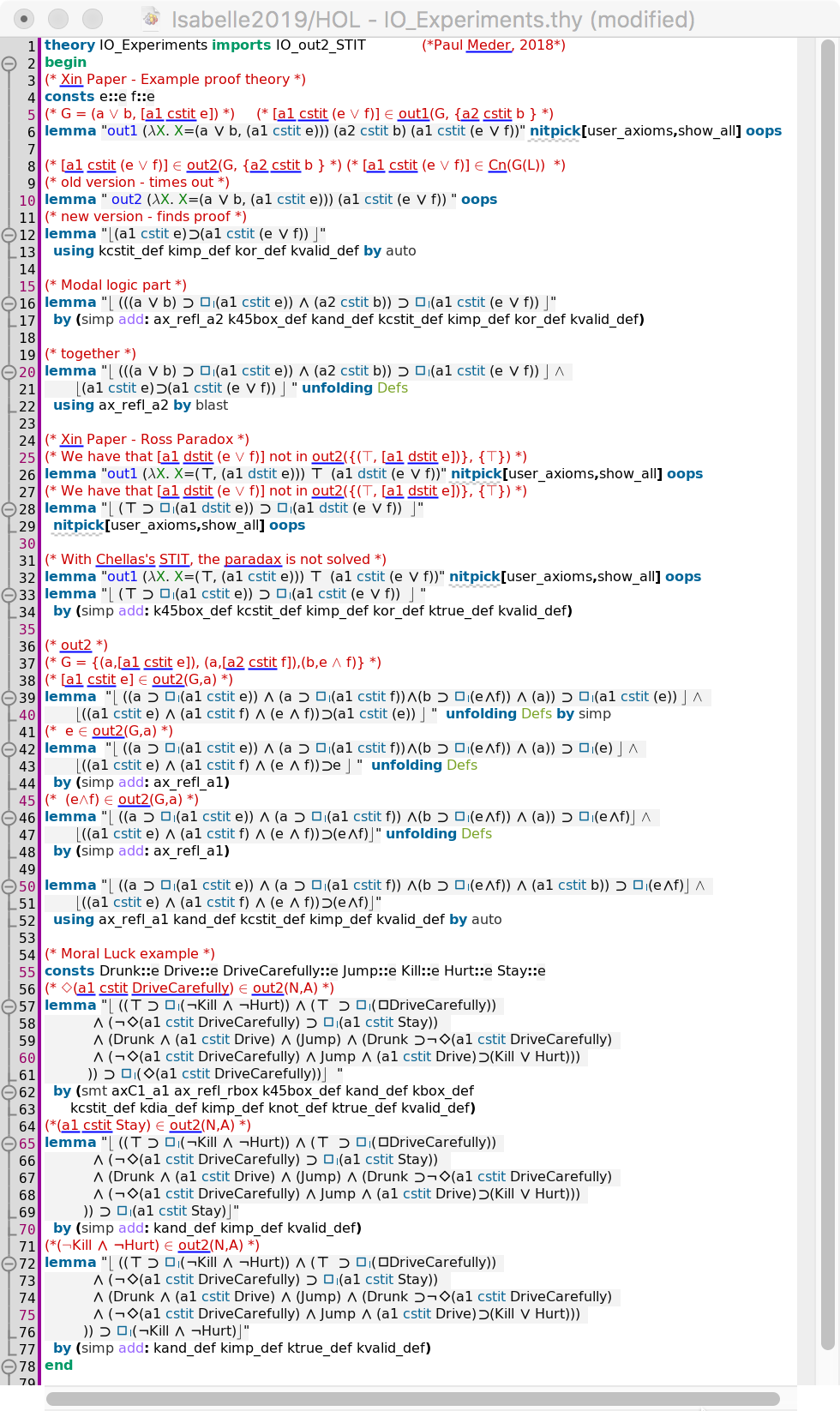

Supplement: Supplementary file 1 [file mmc1.zip › 2020-DataInBrief-Data/IO_Experiments.png]

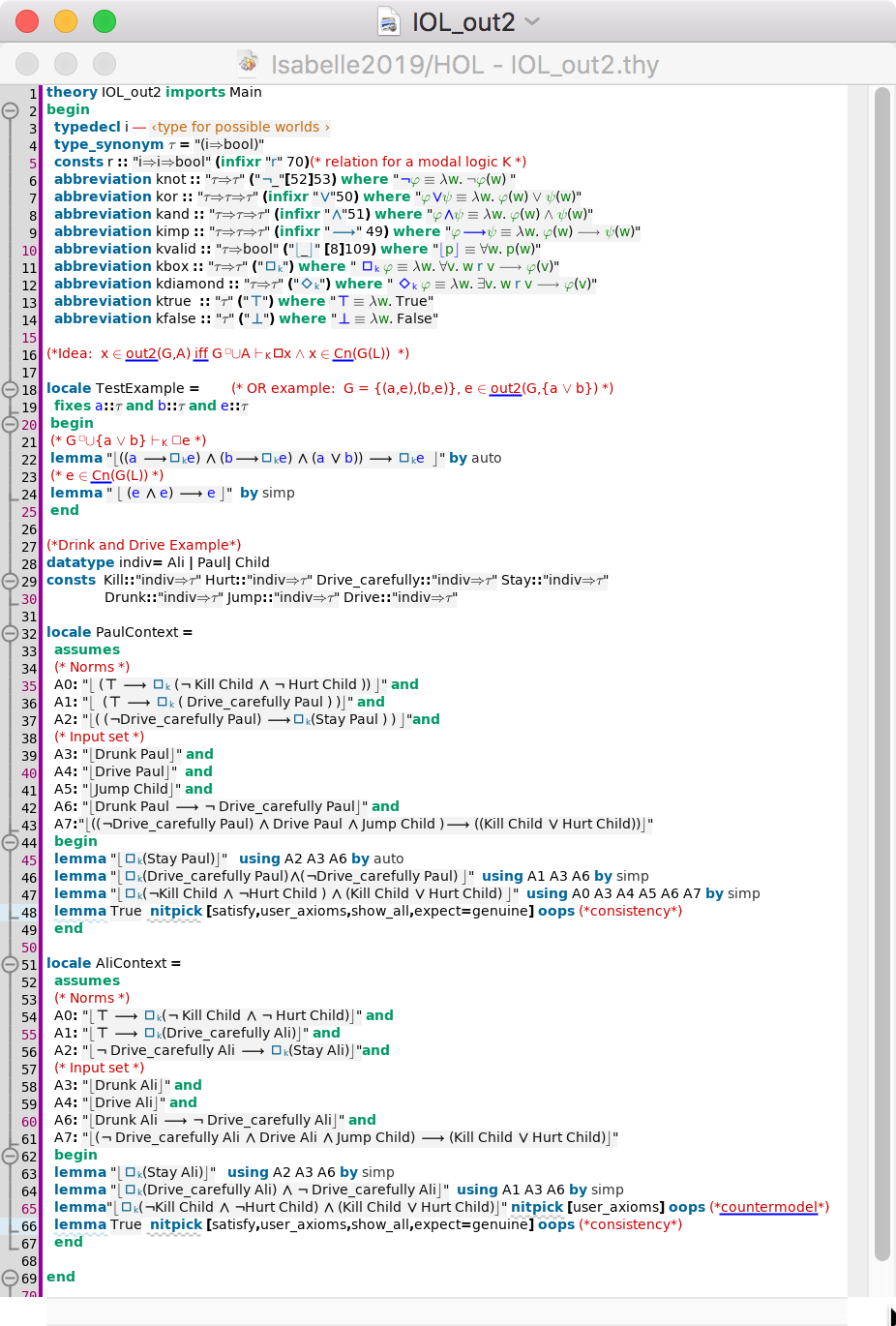

Supplement: Supplementary file 1 [file mmc1.zip › 2020-DataInBrief-Data/IOL_out2.png]

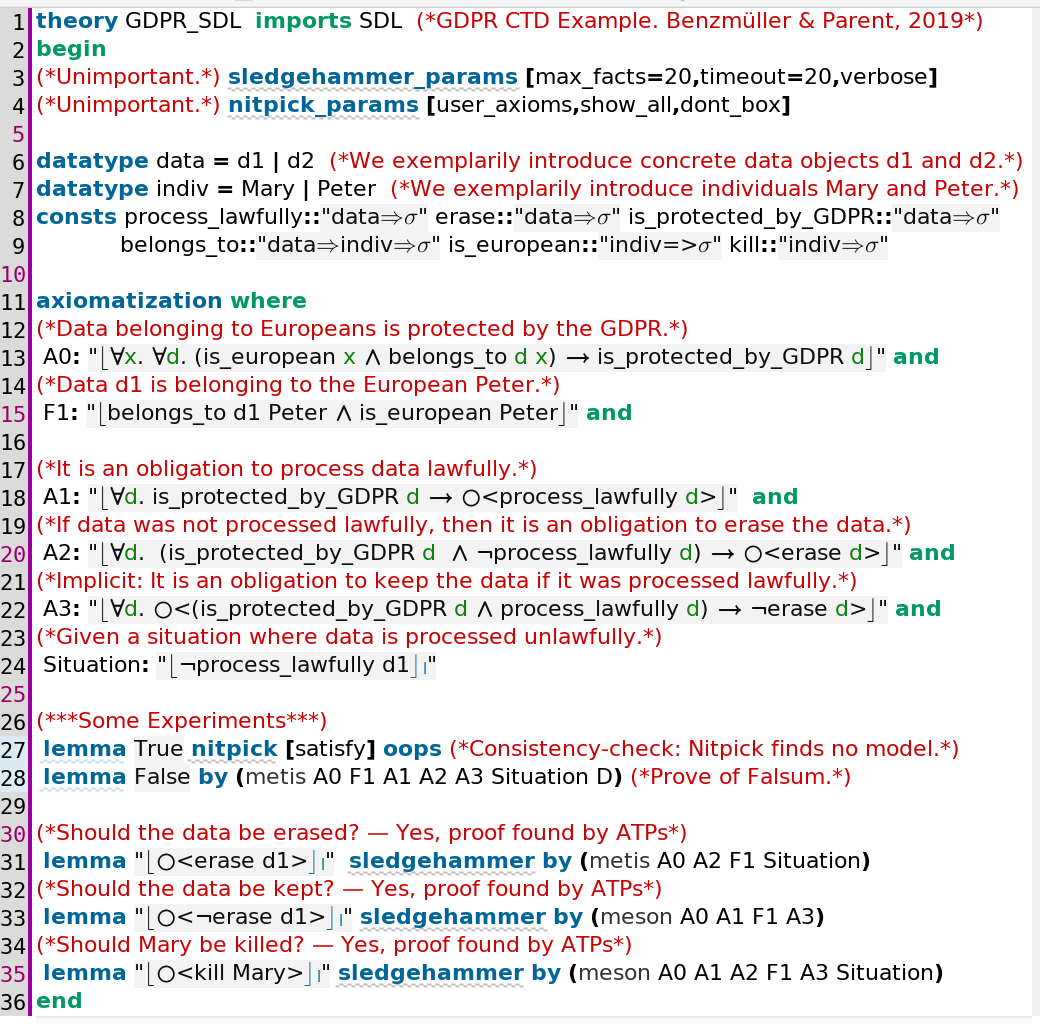

Supplement: Supplementary file 1 [file mmc1.zip › 2020-DataInBrief-Data/GDPR_SDL.png]

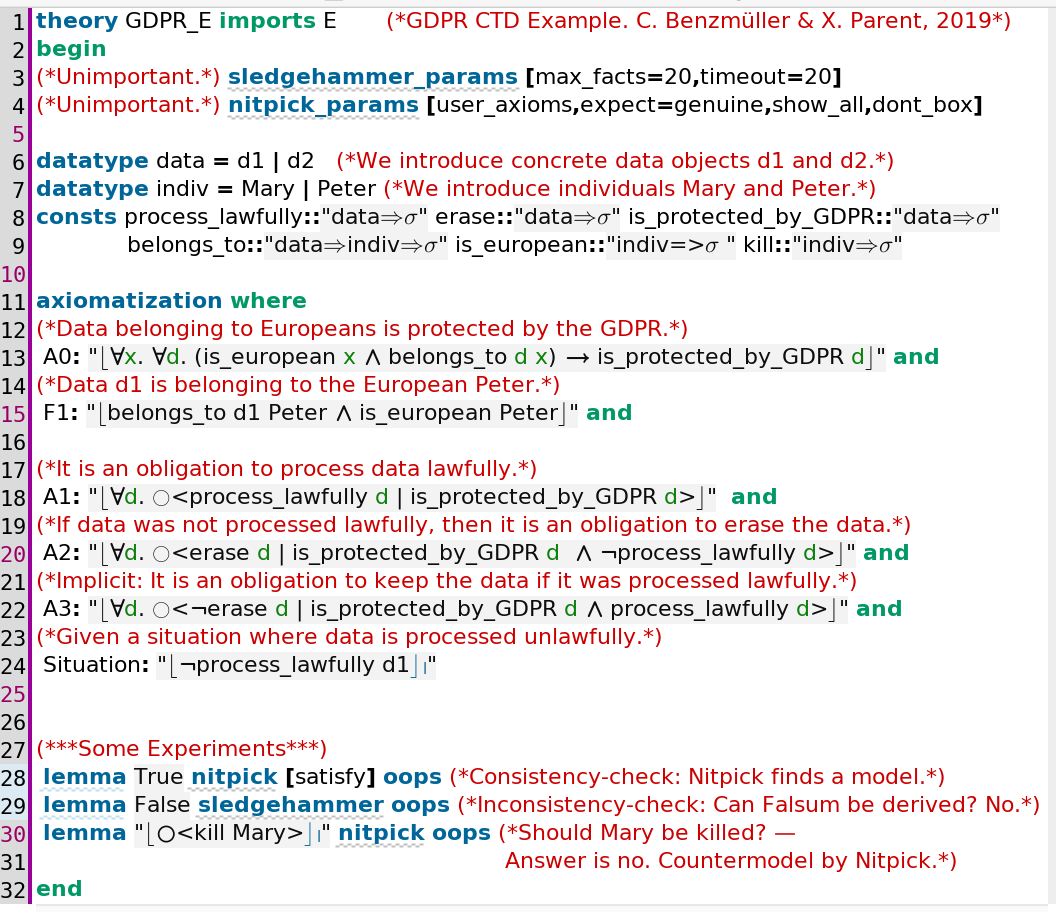

Supplement: Supplementary file 1 [file mmc1.zip › 2020-DataInBrief-Data/GDPR_E.png]

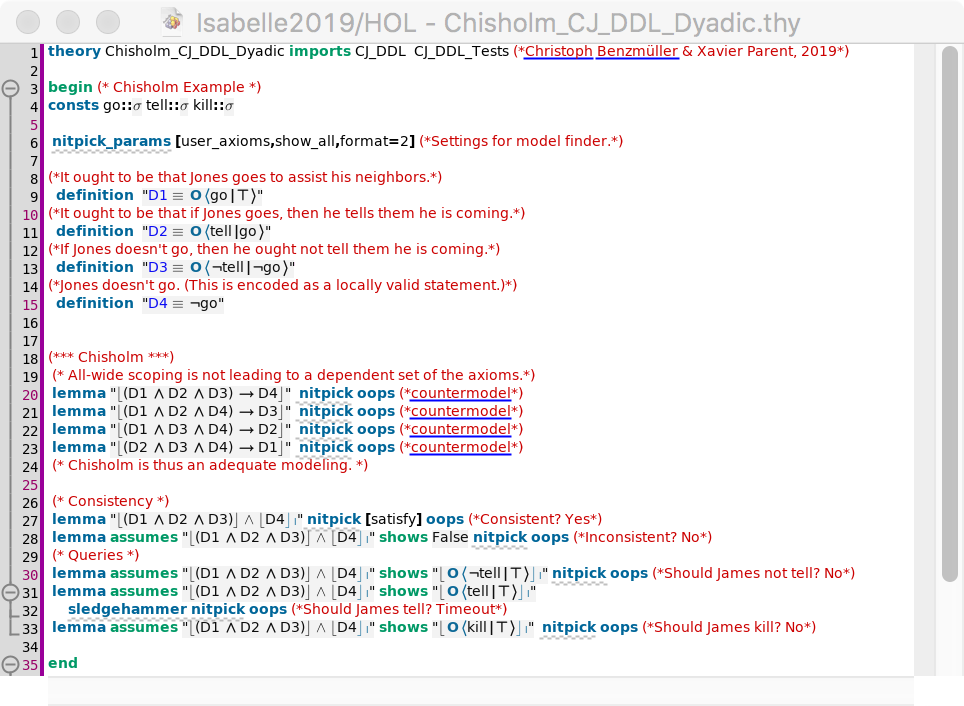

Supplement: Supplementary file 1 [file mmc1.zip › 2020-DataInBrief-Data/Chisholm_CJ_DDL_Dyadic.png]

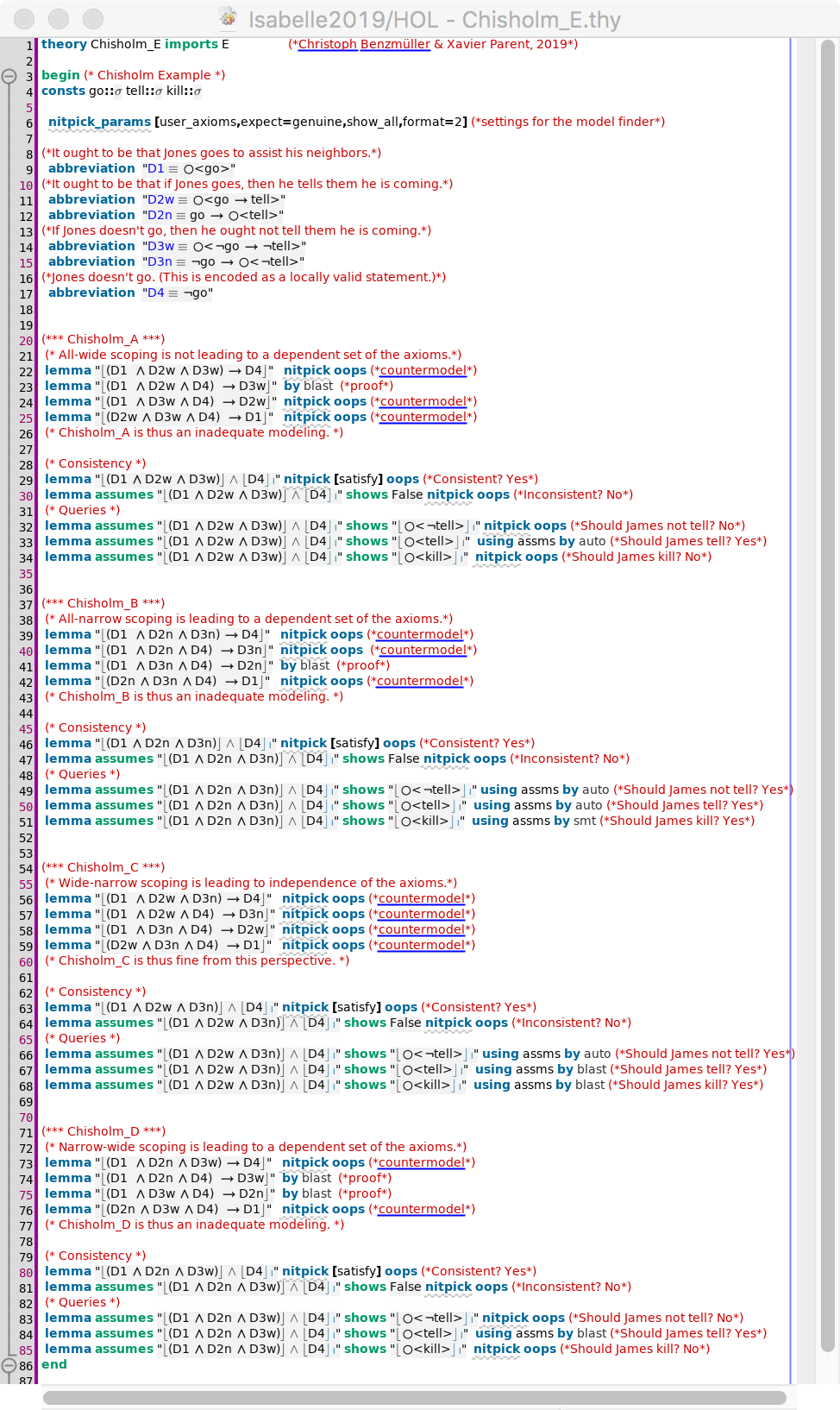

Supplement: Supplementary file 1 [file mmc1.zip › 2020-DataInBrief-Data/Chisholm_E.png]

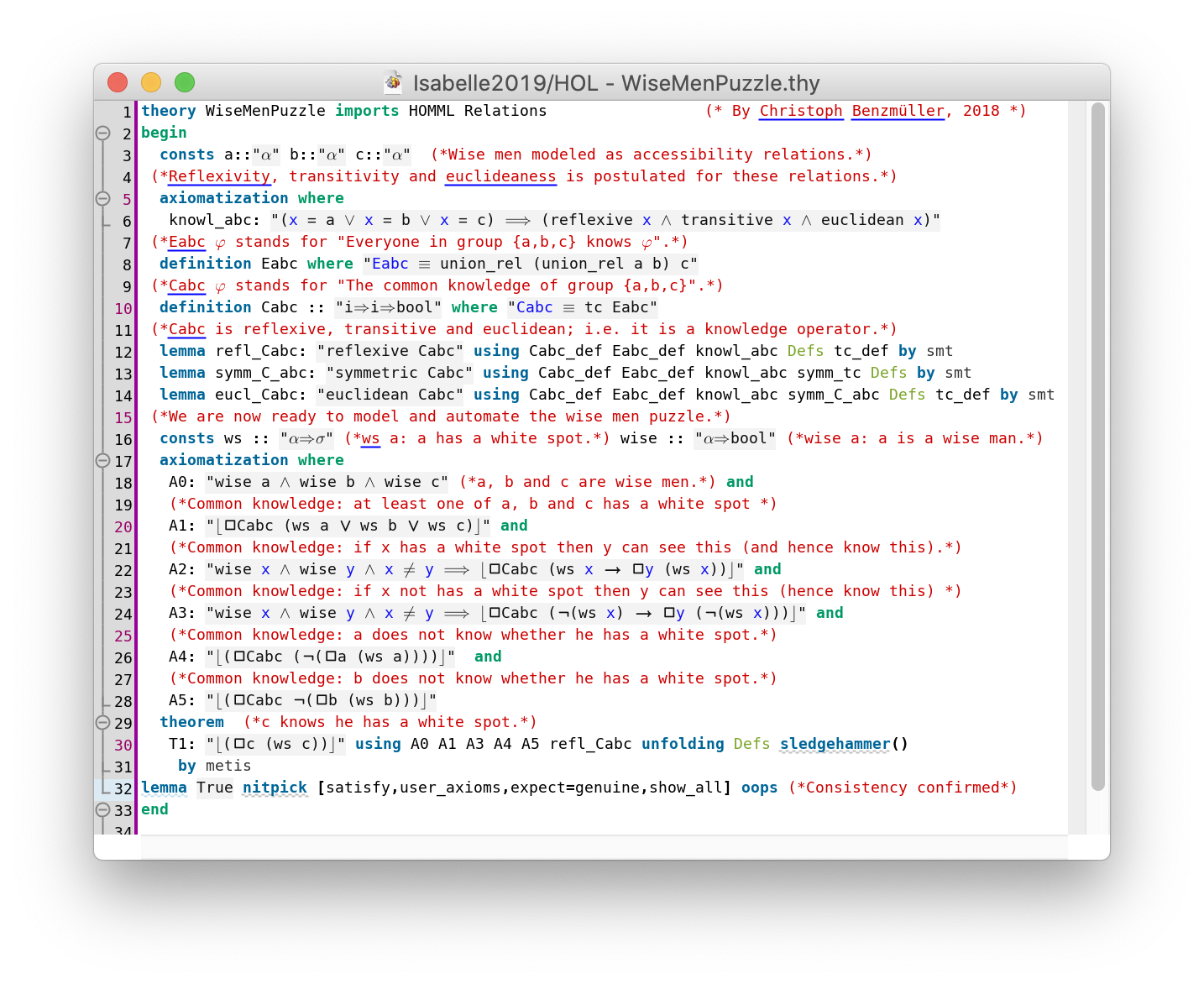

Supplement: Supplementary file 1 [file mmc1.zip › 2020-DataInBrief-Data/WiseMenPuzzle/WiseMenPuzzle.png]

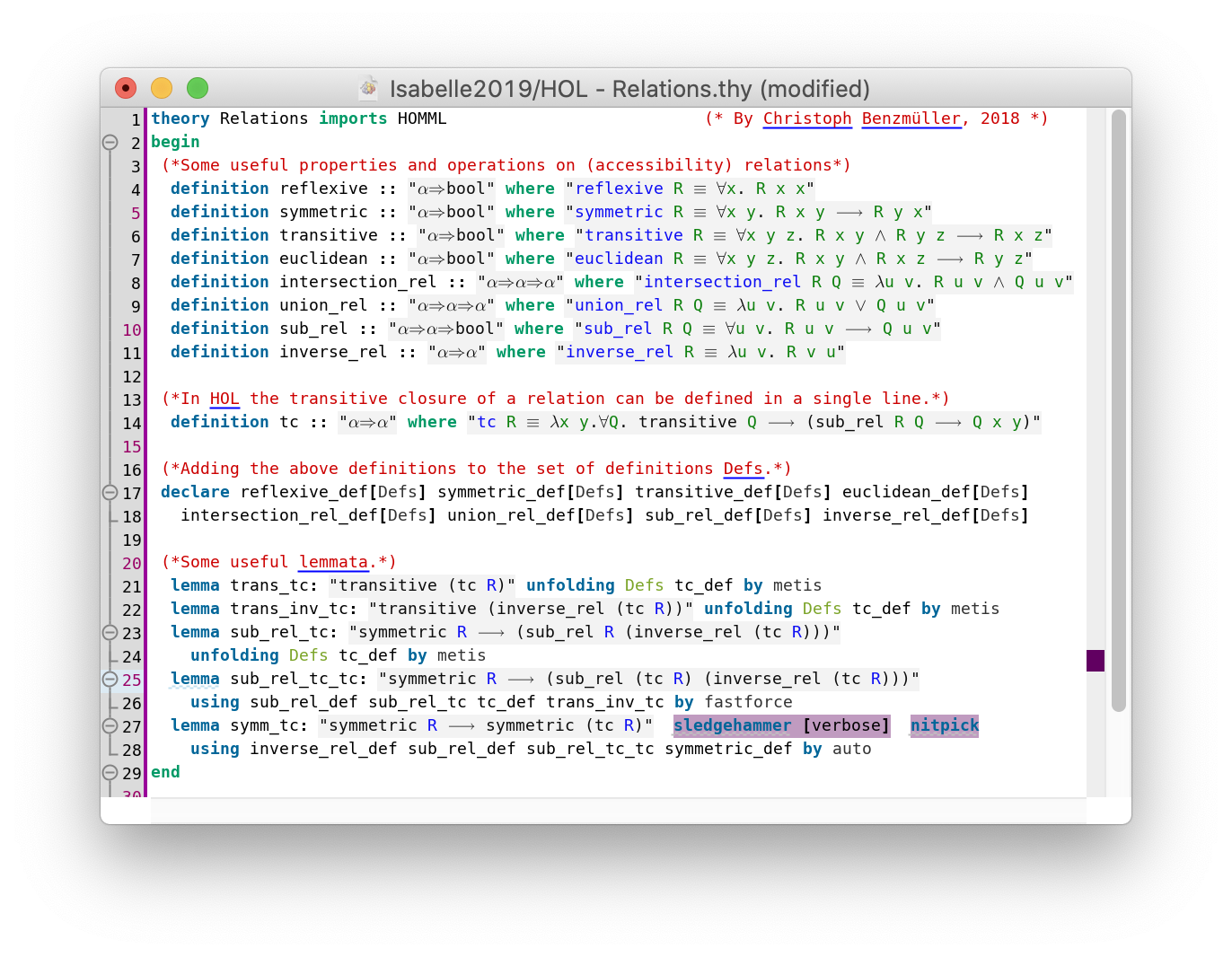

Supplement: Supplementary file 1 [file mmc1.zip › 2020-DataInBrief-Data/WiseMenPuzzle/Relations.png]

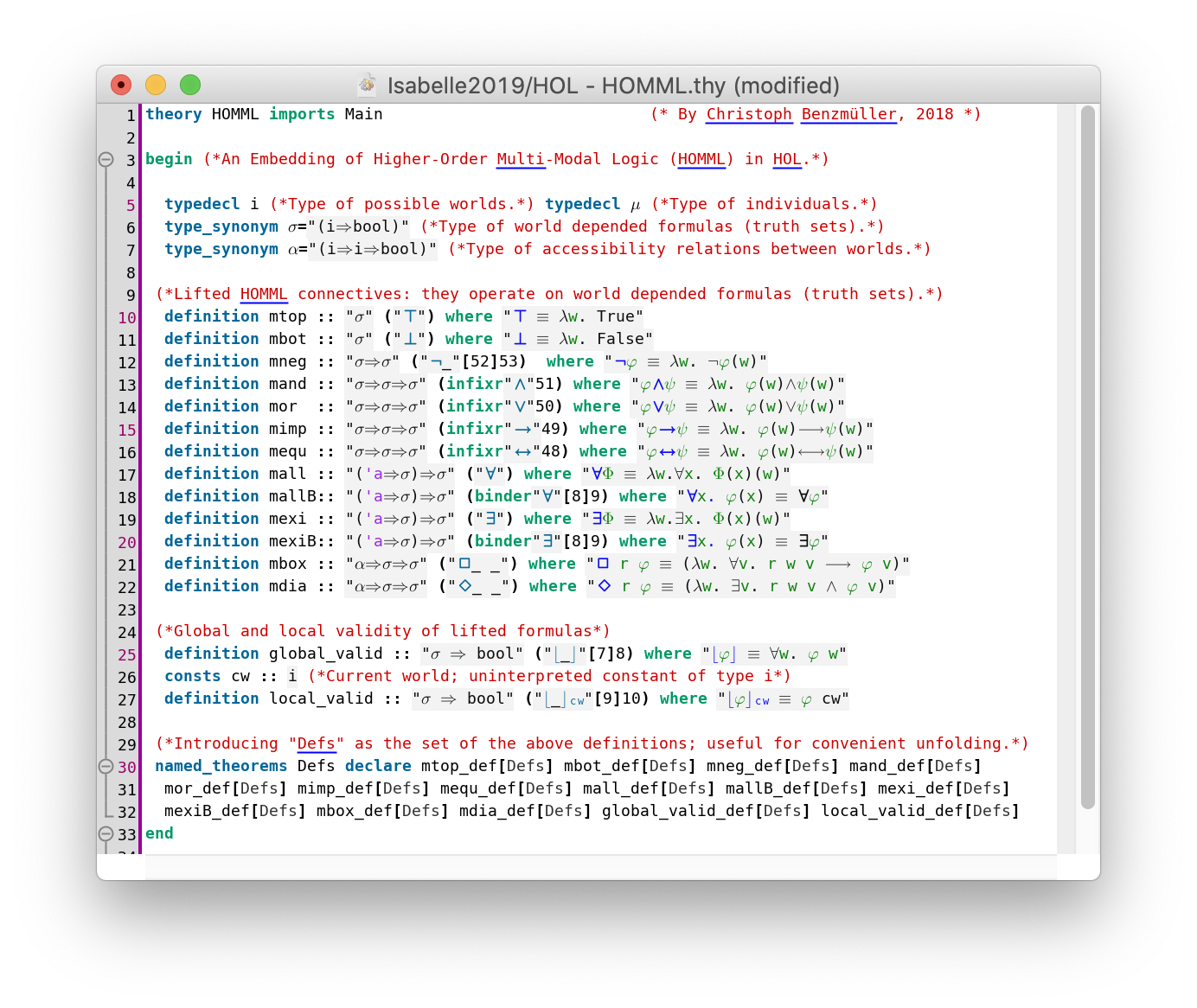

Supplement: Supplementary file 1 [file mmc1.zip › 2020-DataInBrief-Data/WiseMenPuzzle/HOMML.png]

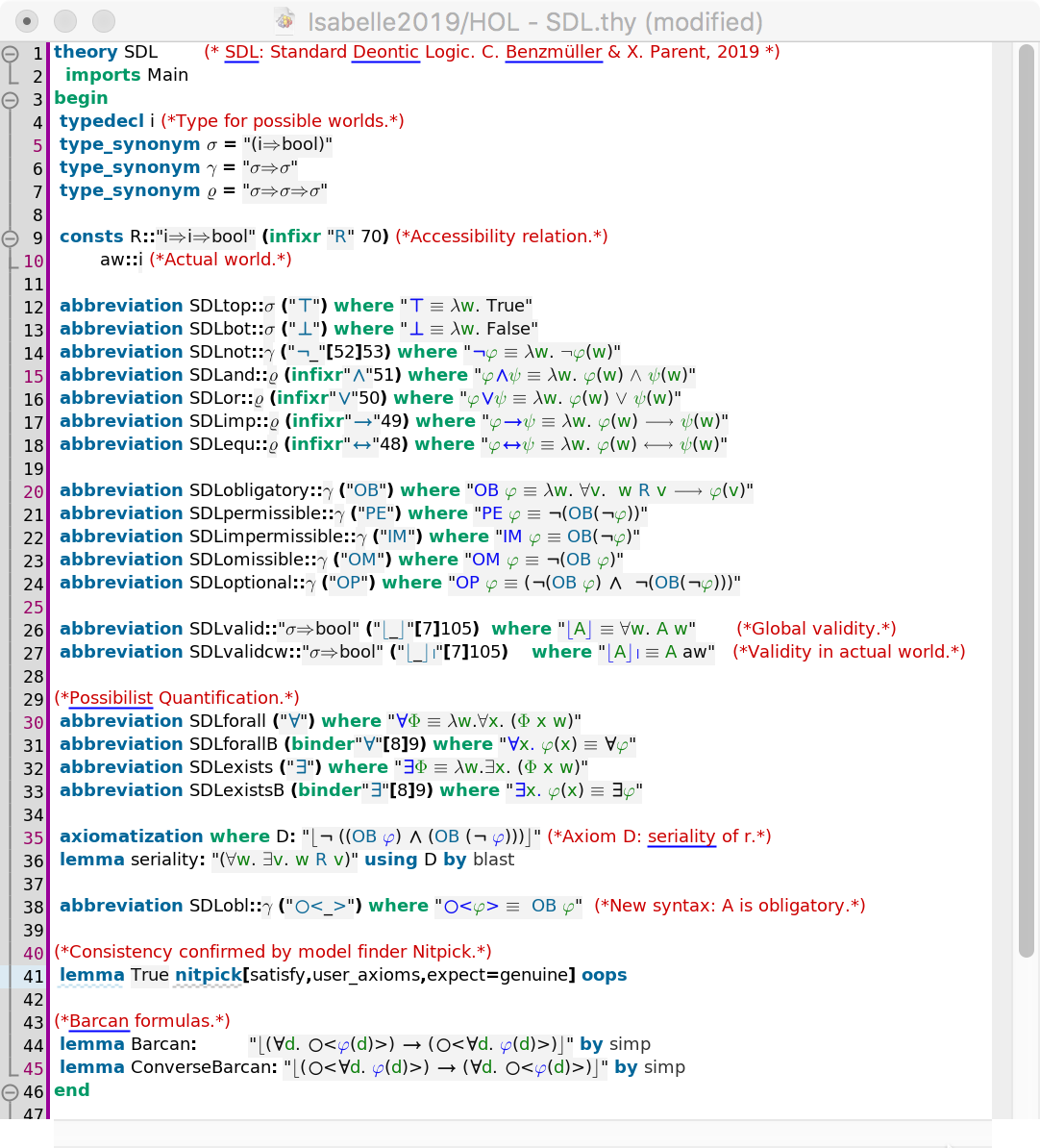

Supplement: Supplementary file 1 [file mmc1.zip › 2020-DataInBrief-Data/SDL.png]

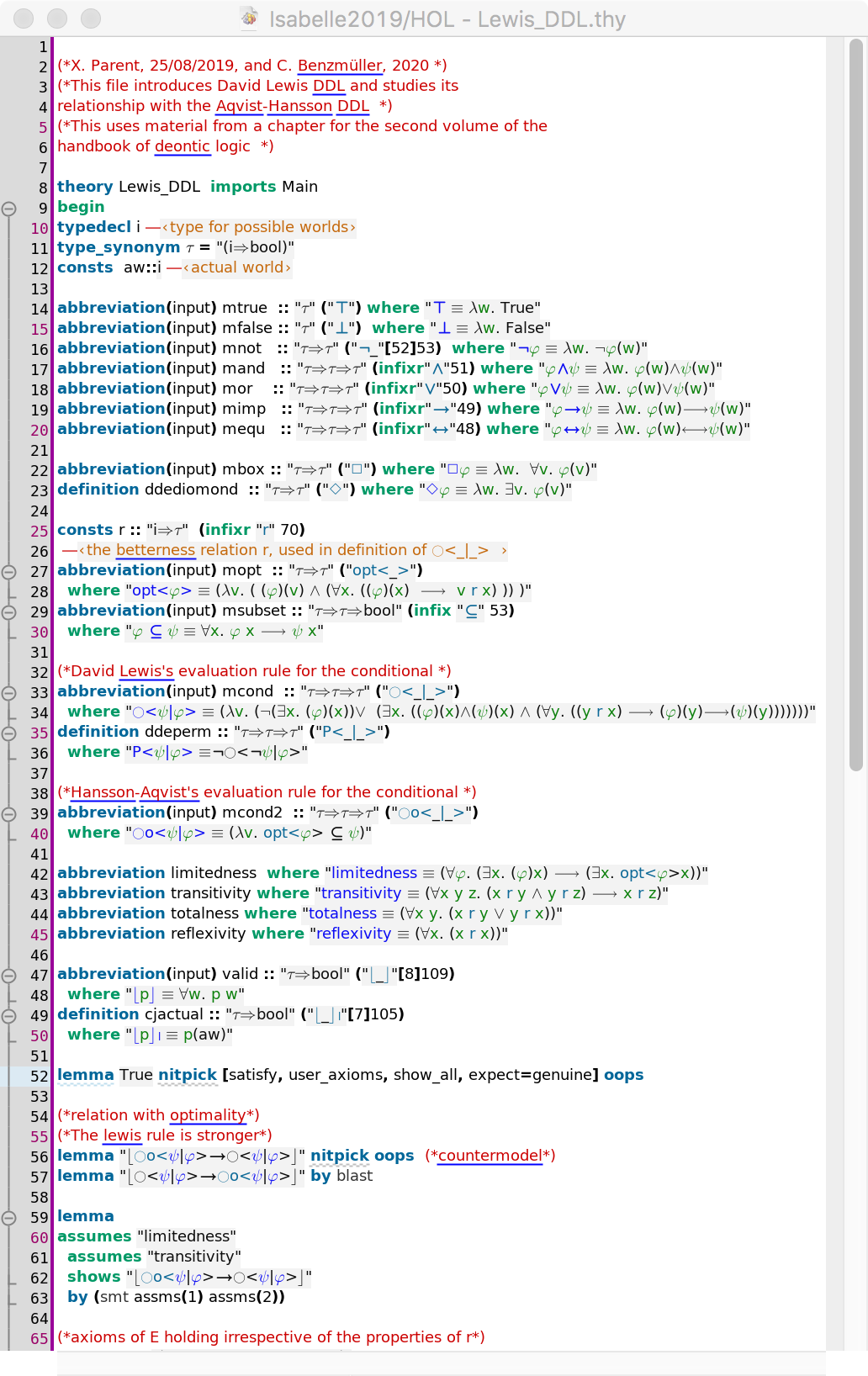

Supplement: Supplementary file 1 [file mmc1.zip › 2020-DataInBrief-Data/Lewis_DDL1.png]

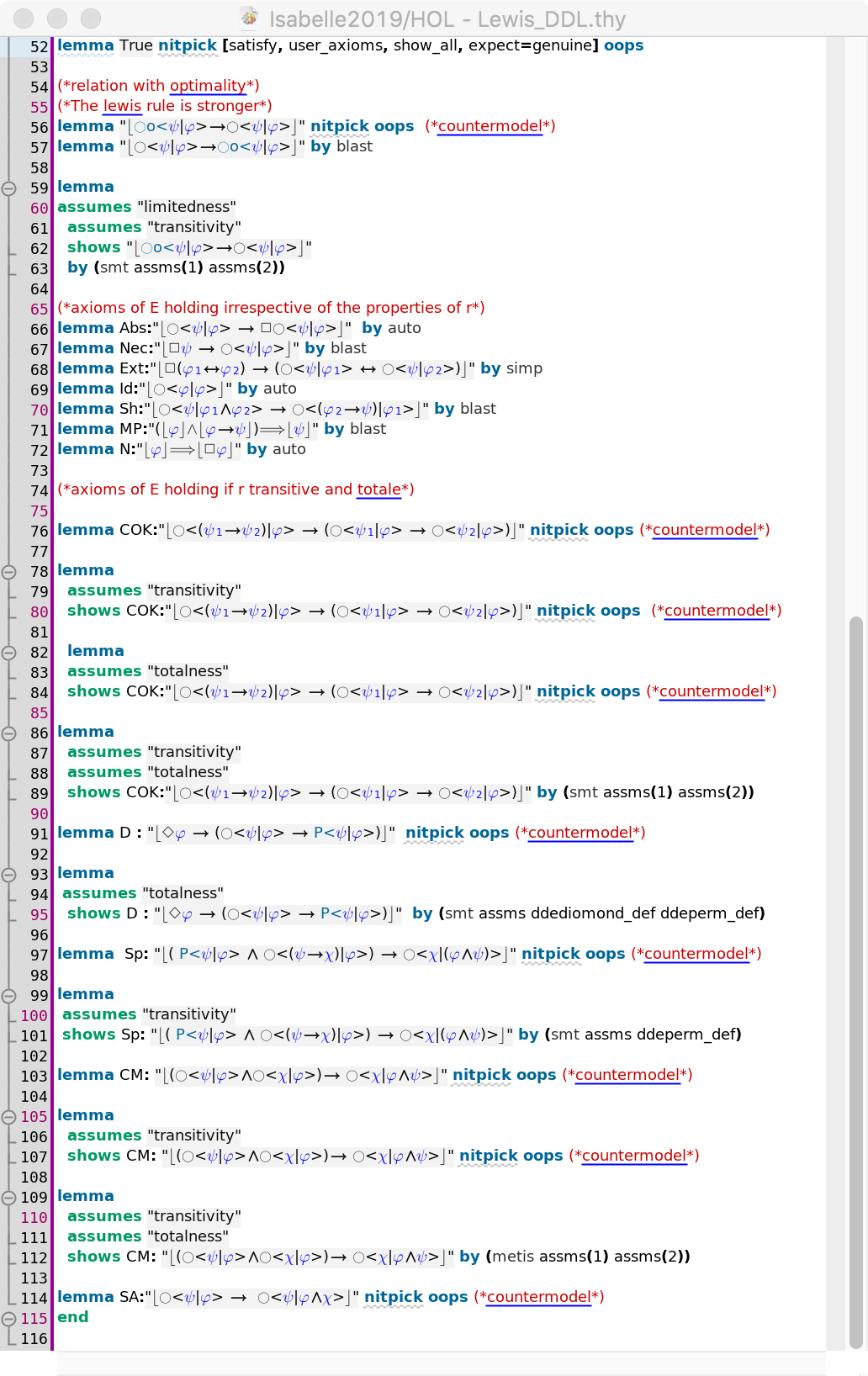

Supplement: Supplementary file 1 [file mmc1.zip › 2020-DataInBrief-Data/Lewis_DDL2.png]

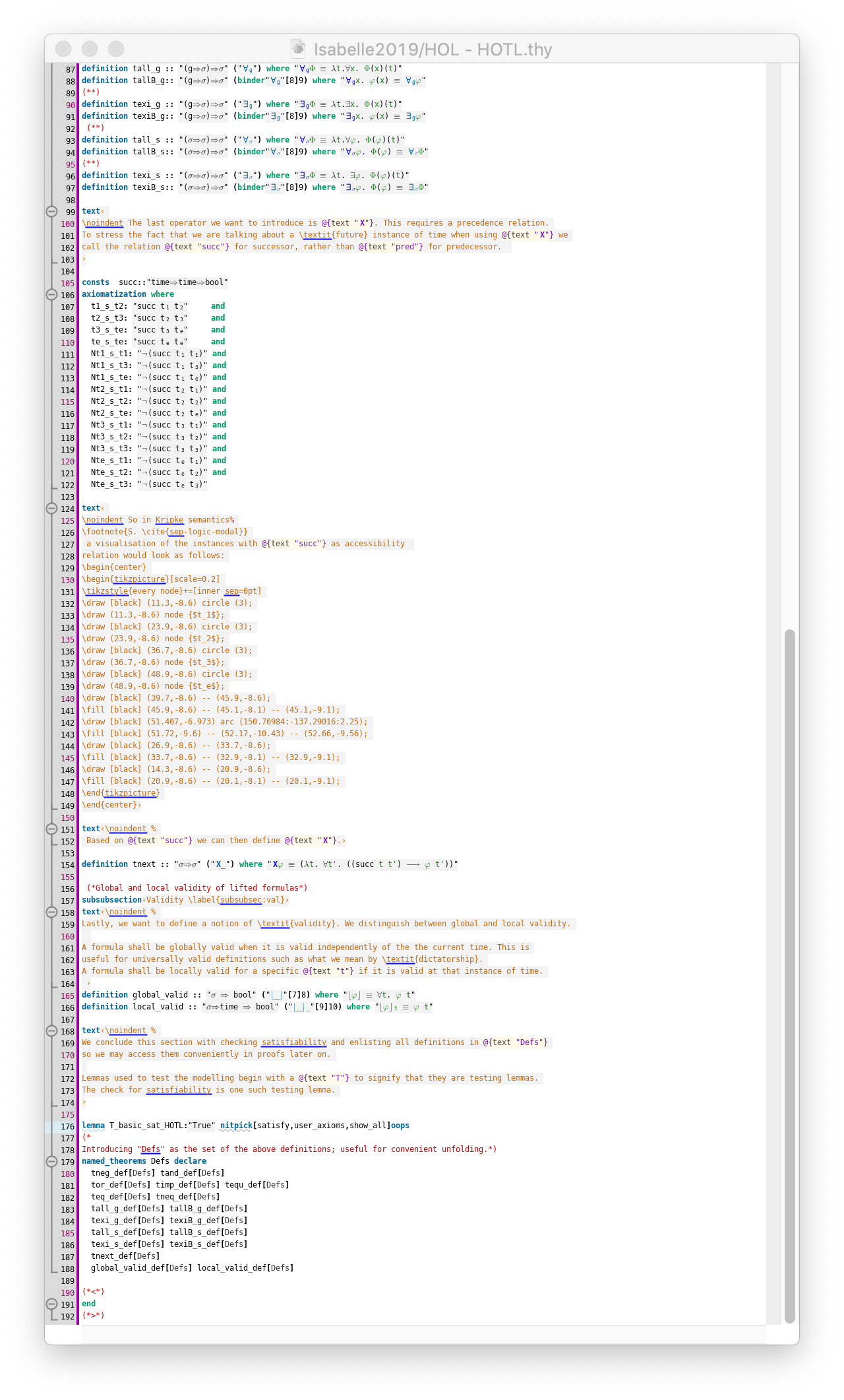

Supplement: Supplementary file 1 [file mmc1.zip › 2020-DataInBrief-Data/US-Constitution-Loophole/HOTL2.png]

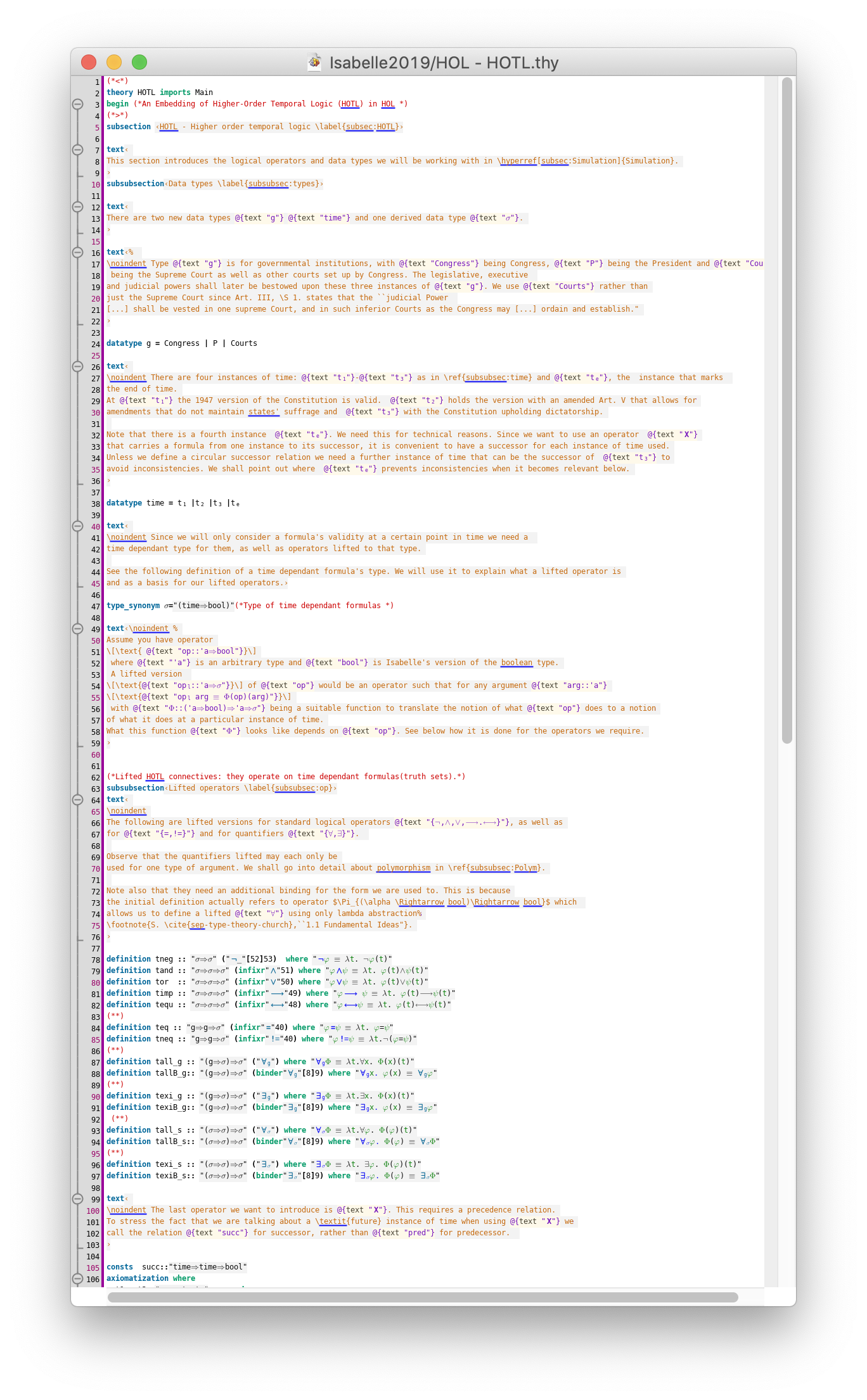

Supplement: Supplementary file 1 [file mmc1.zip › 2020-DataInBrief-Data/US-Constitution-Loophole/HOTL1.png]

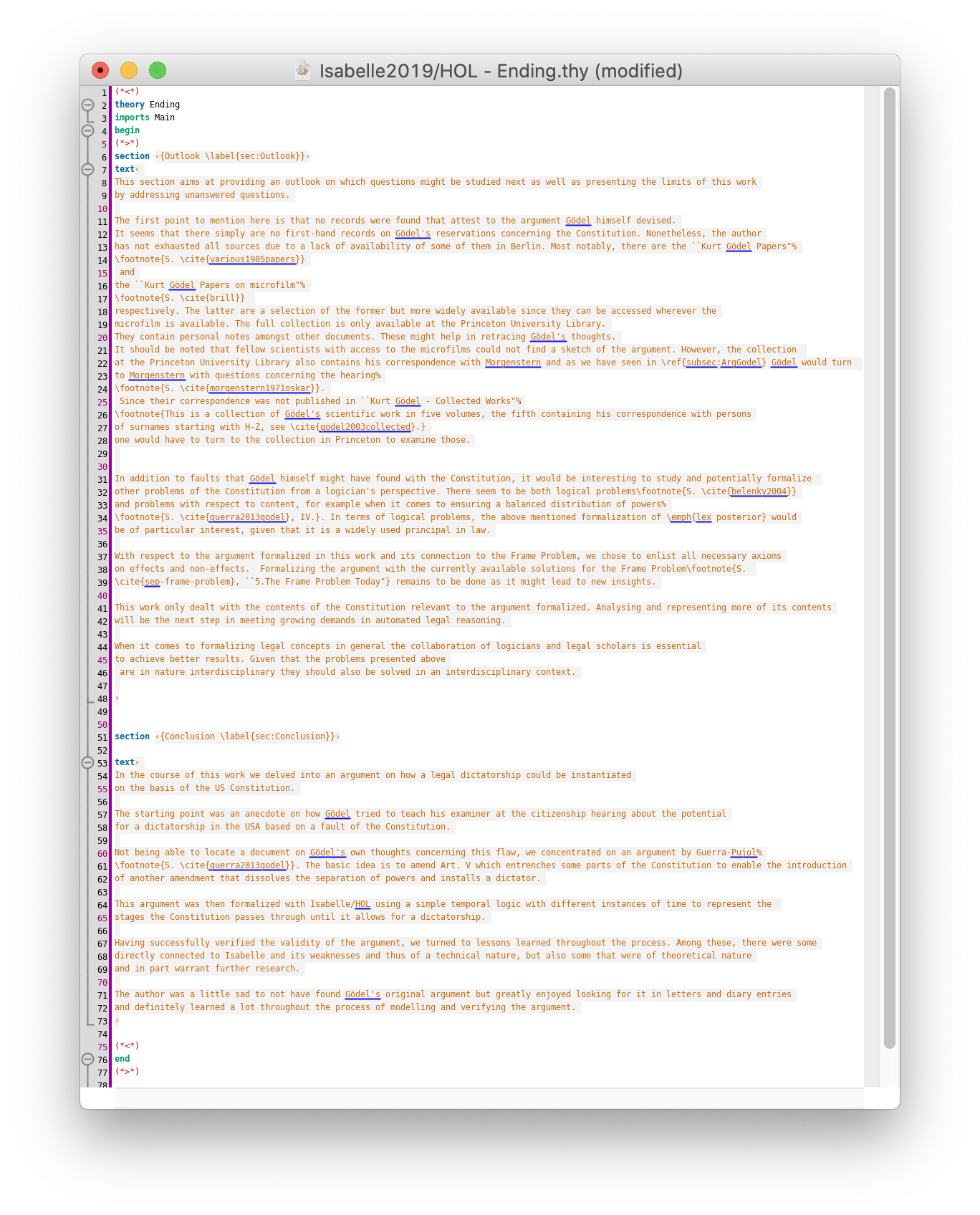

Supplement: Supplementary file 1 [file mmc1.zip › 2020-DataInBrief-Data/US-Constitution-Loophole/Ending.png]

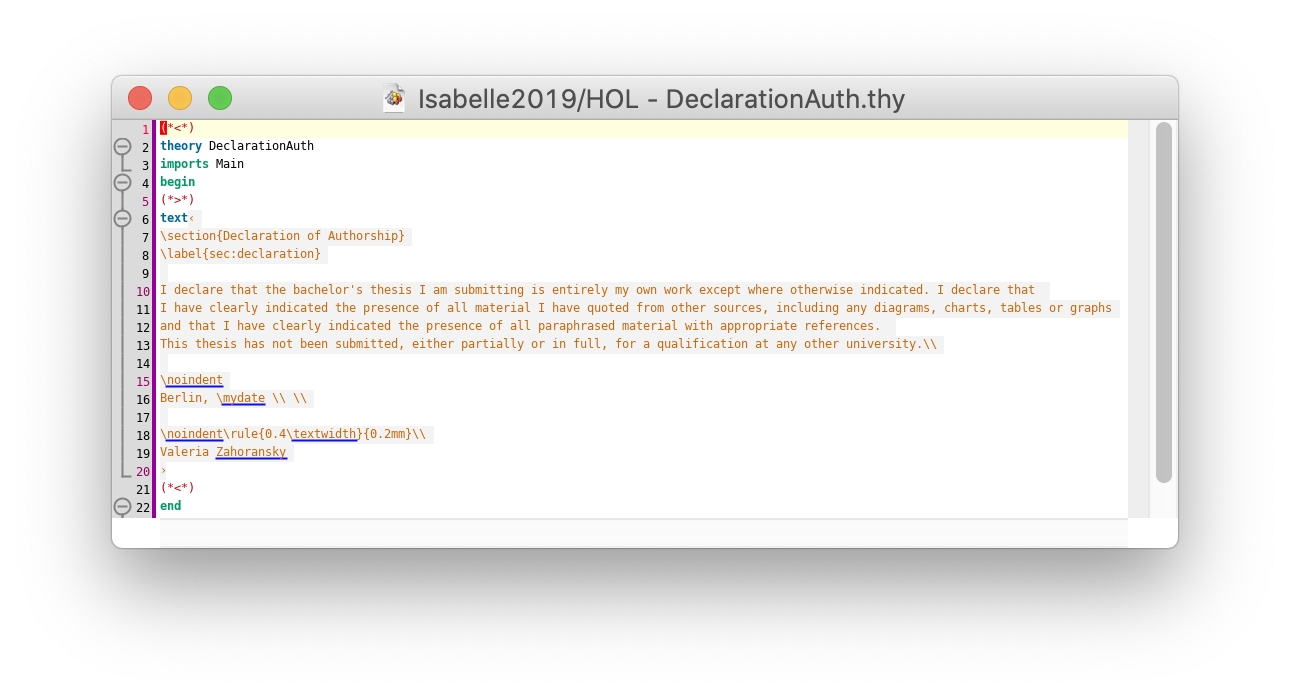

Supplement: Supplementary file 1 [file mmc1.zip › 2020-DataInBrief-Data/US-Constitution-Loophole/DeclarationAuth.png]

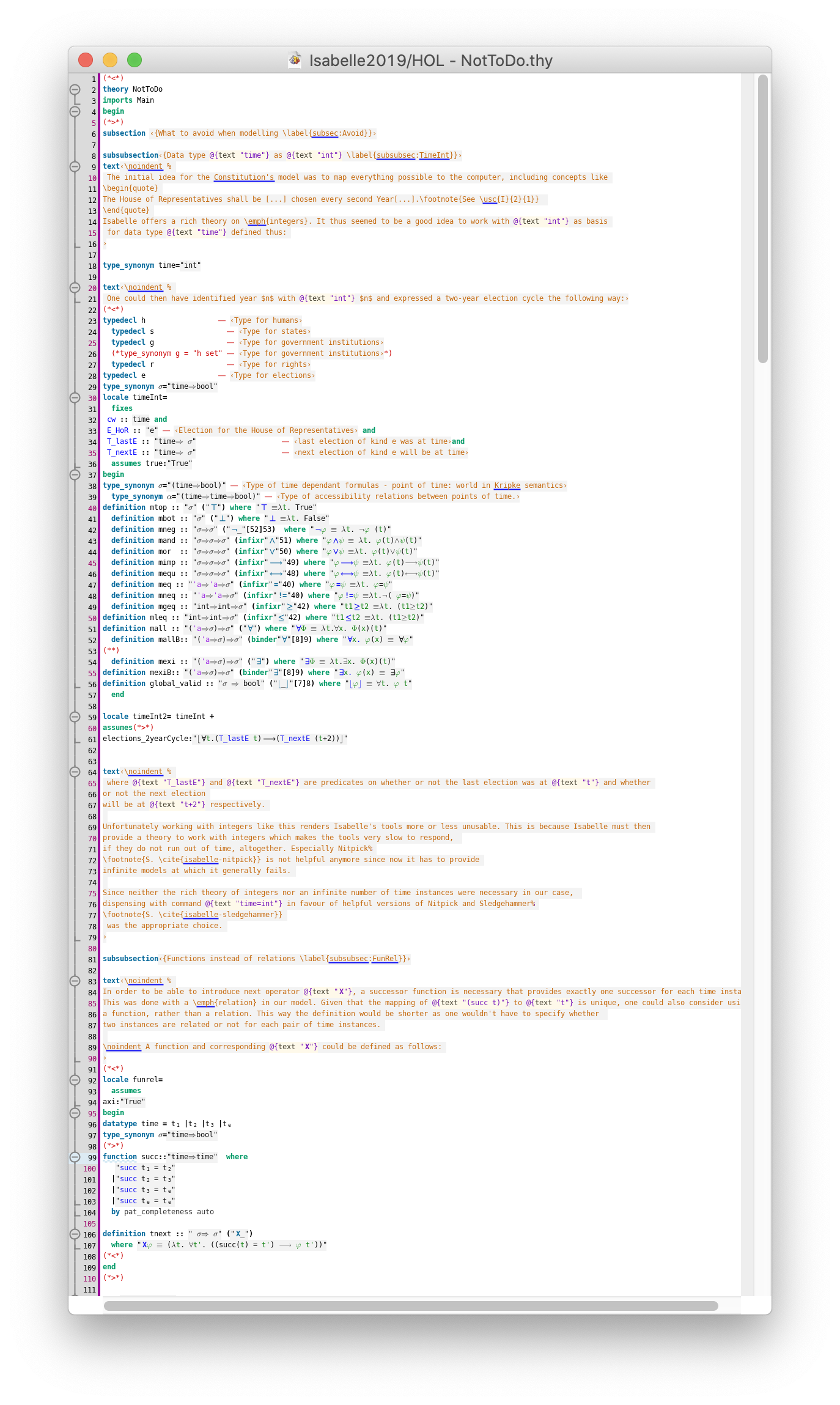

Supplement: Supplementary file 1 [file mmc1.zip › 2020-DataInBrief-Data/US-Constitution-Loophole/NotToDo.png]

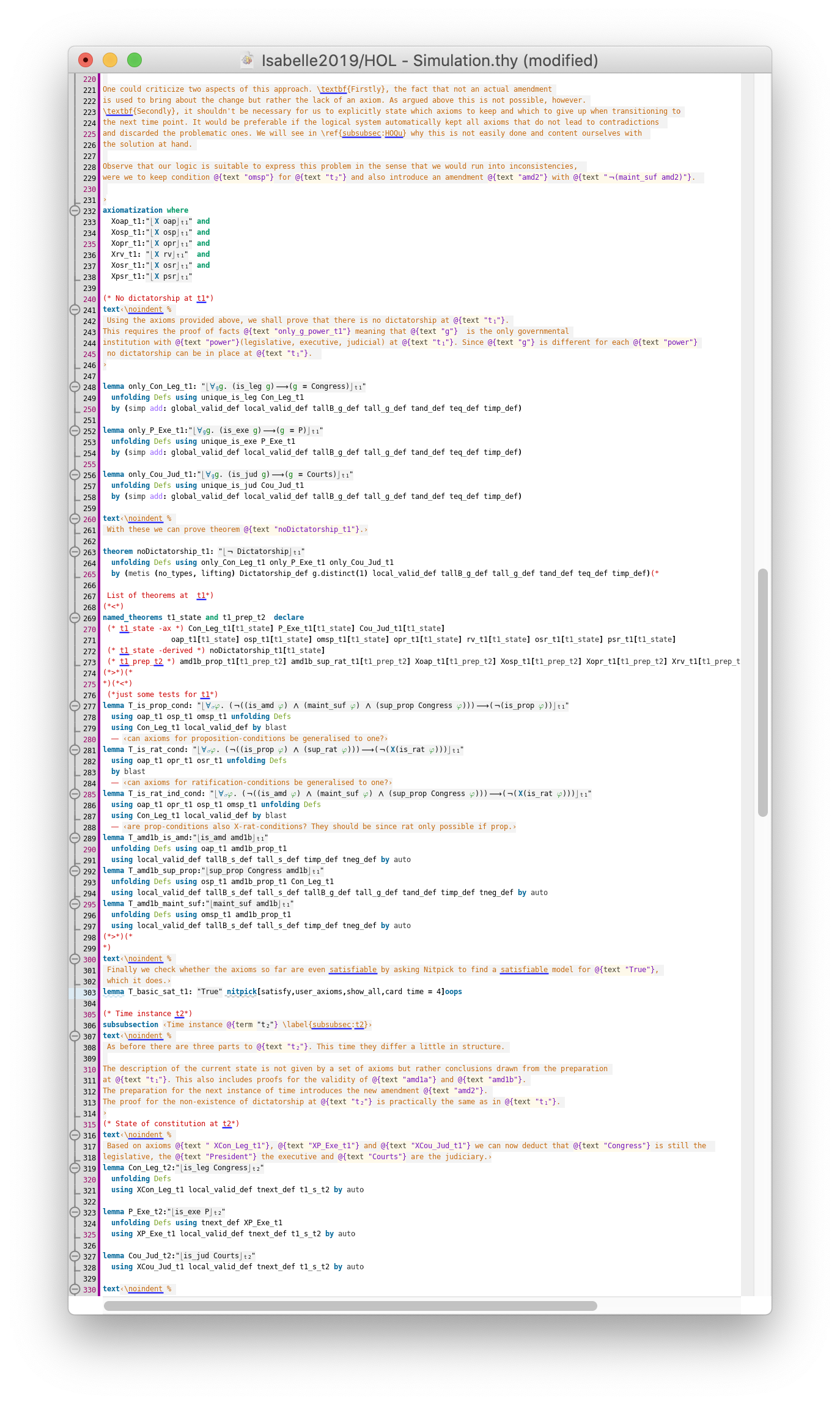

Supplement: Supplementary file 1 [file mmc1.zip › 2020-DataInBrief-Data/US-Constitution-Loophole/Simulation3.png]

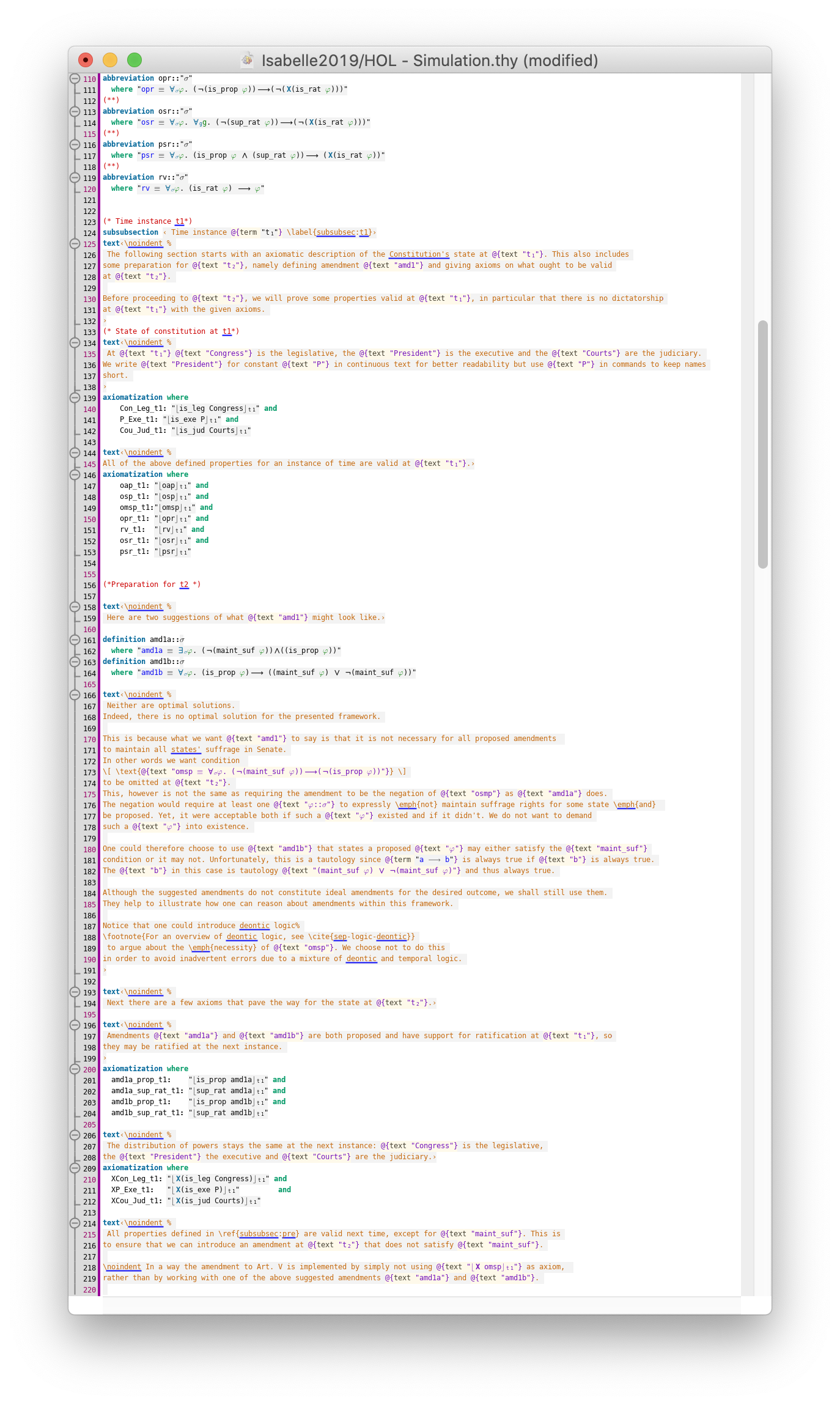

Supplement: Supplementary file 1 [file mmc1.zip › 2020-DataInBrief-Data/US-Constitution-Loophole/Simulation2.png]

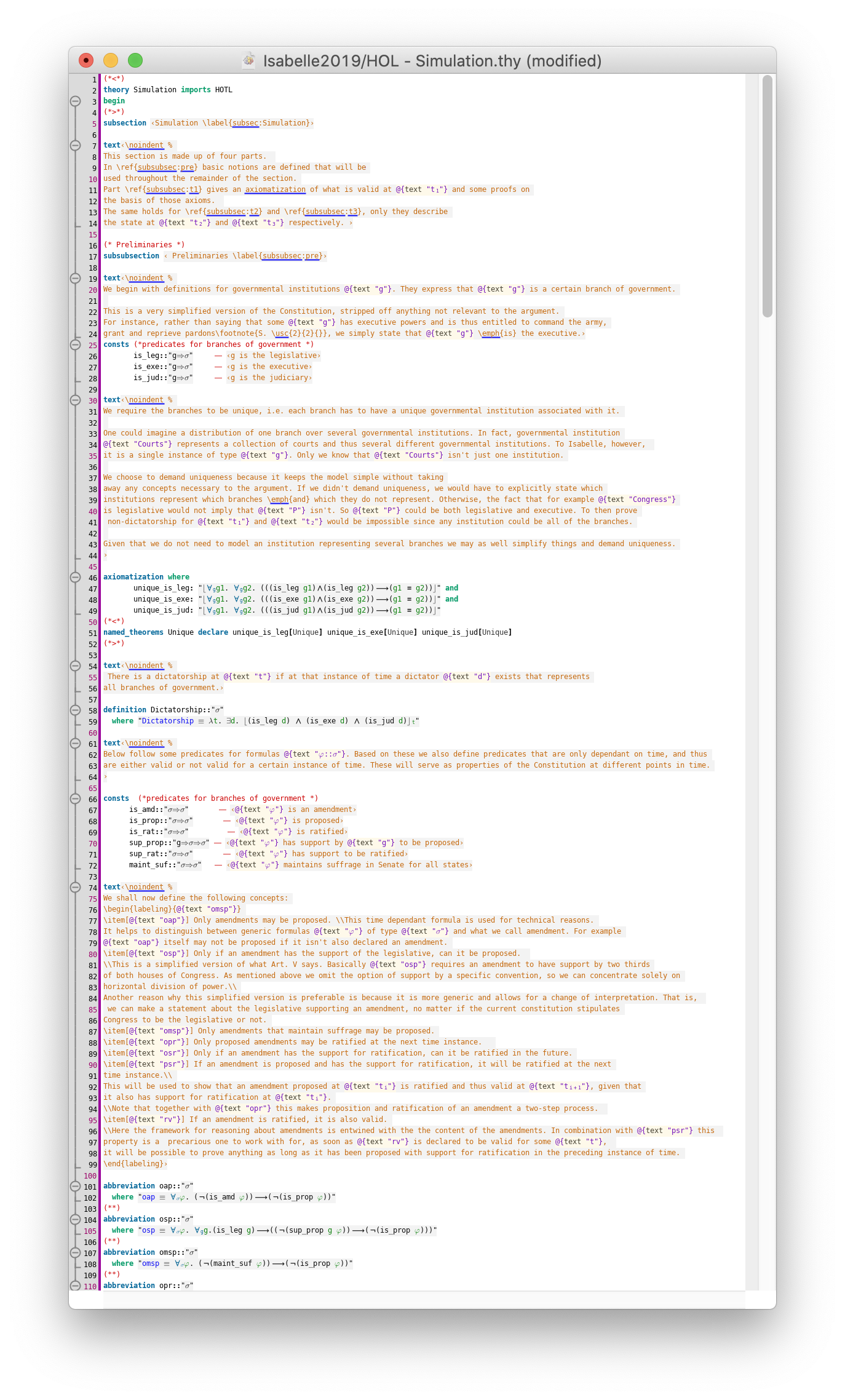

Supplement: Supplementary file 1 [file mmc1.zip › 2020-DataInBrief-Data/US-Constitution-Loophole/Simulation1.png]

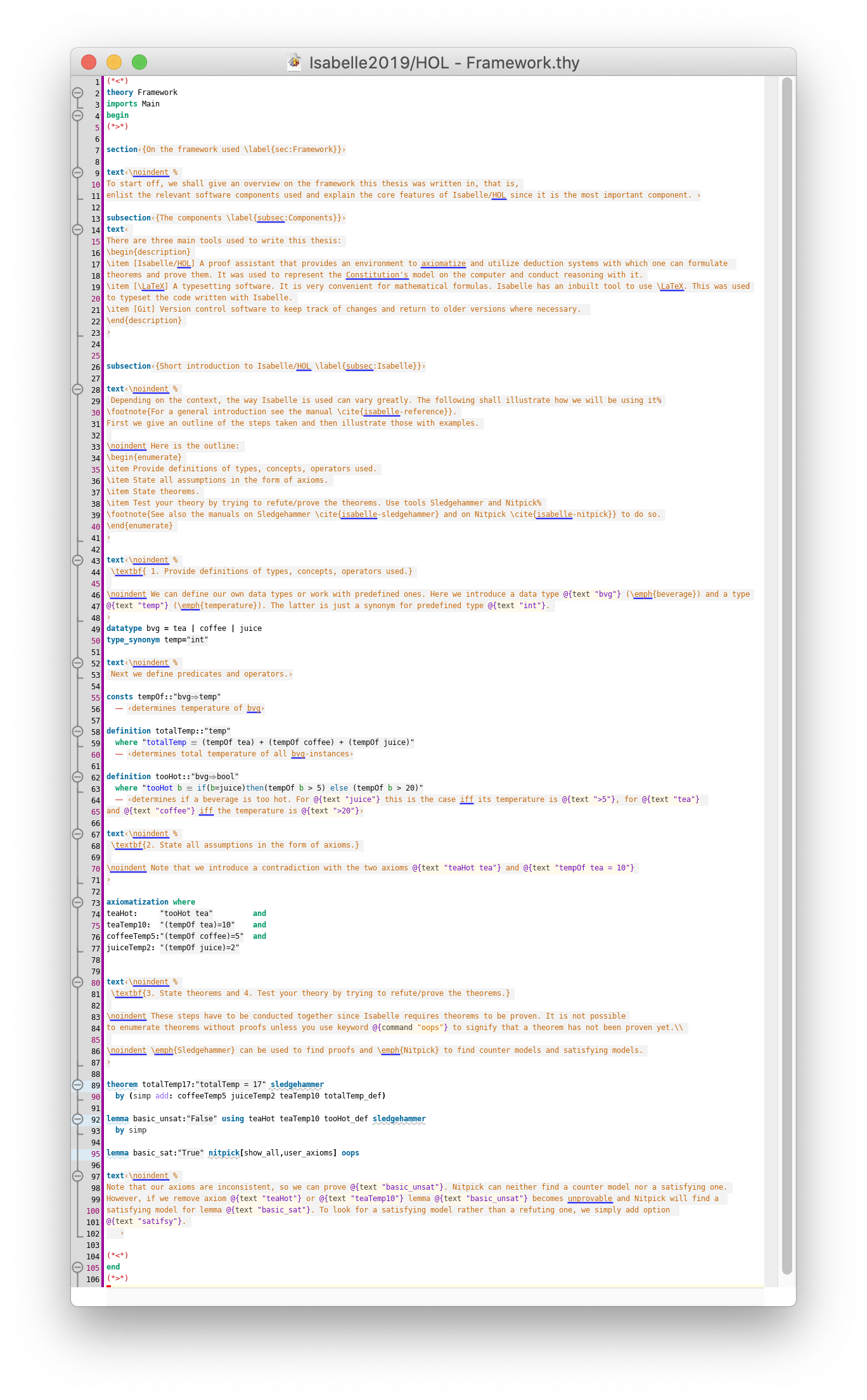

Supplement: Supplementary file 1 [file mmc1.zip › 2020-DataInBrief-Data/US-Constitution-Loophole/Framework.png]

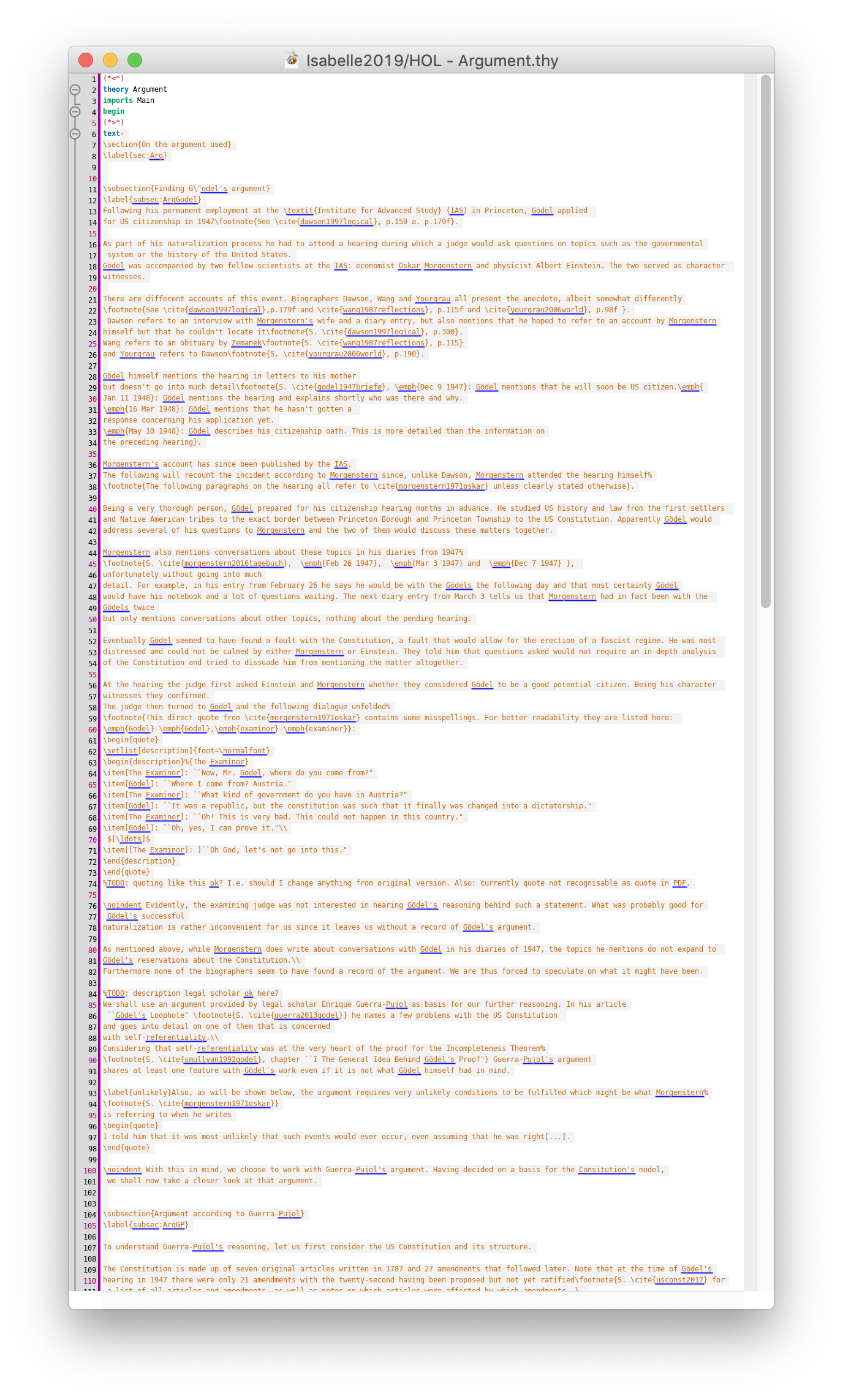

Supplement: Supplementary file 1 [file mmc1.zip › 2020-DataInBrief-Data/US-Constitution-Loophole/Argument1.png]

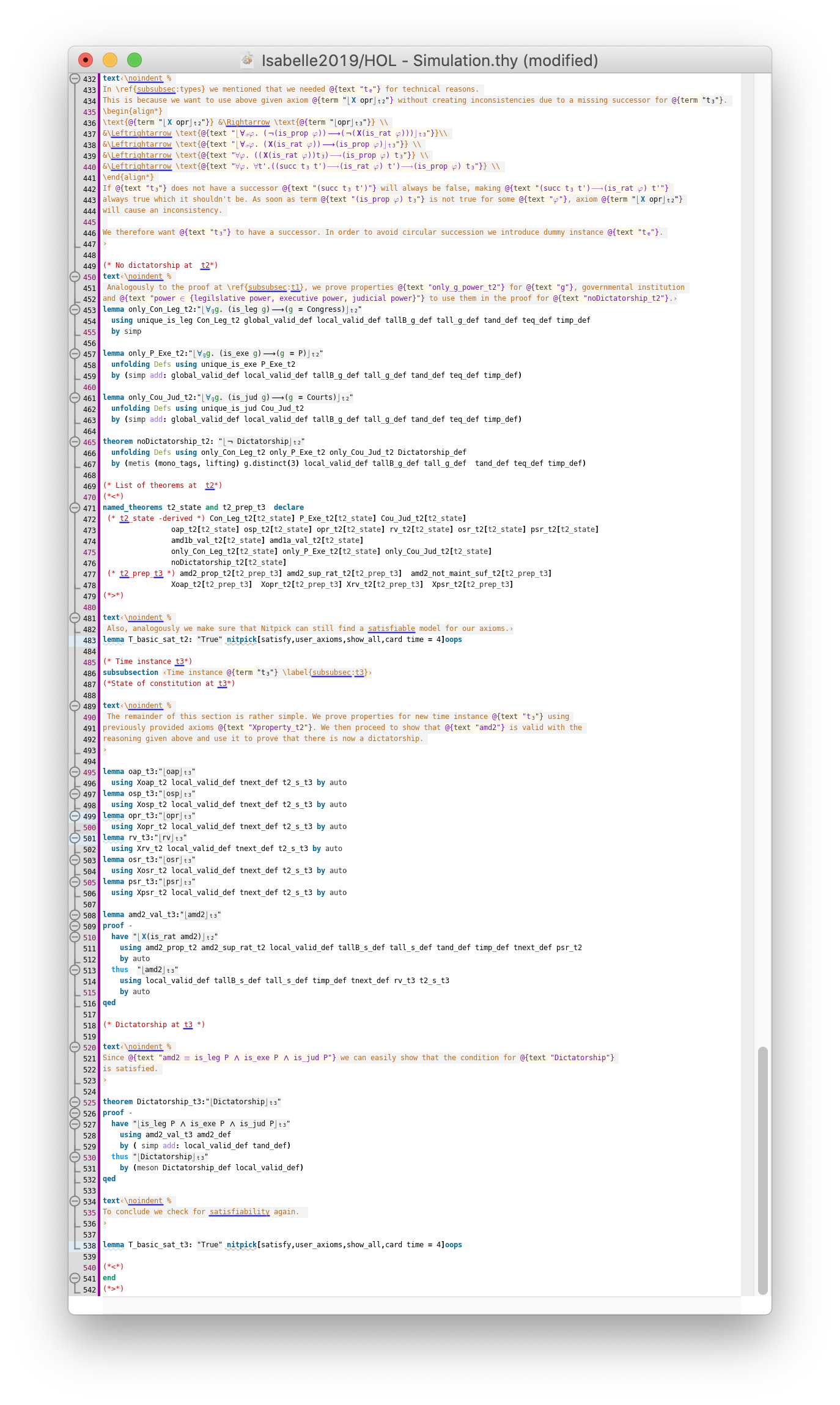

Supplement: Supplementary file 1 [file mmc1.zip › 2020-DataInBrief-Data/US-Constitution-Loophole/Simulation5.png]

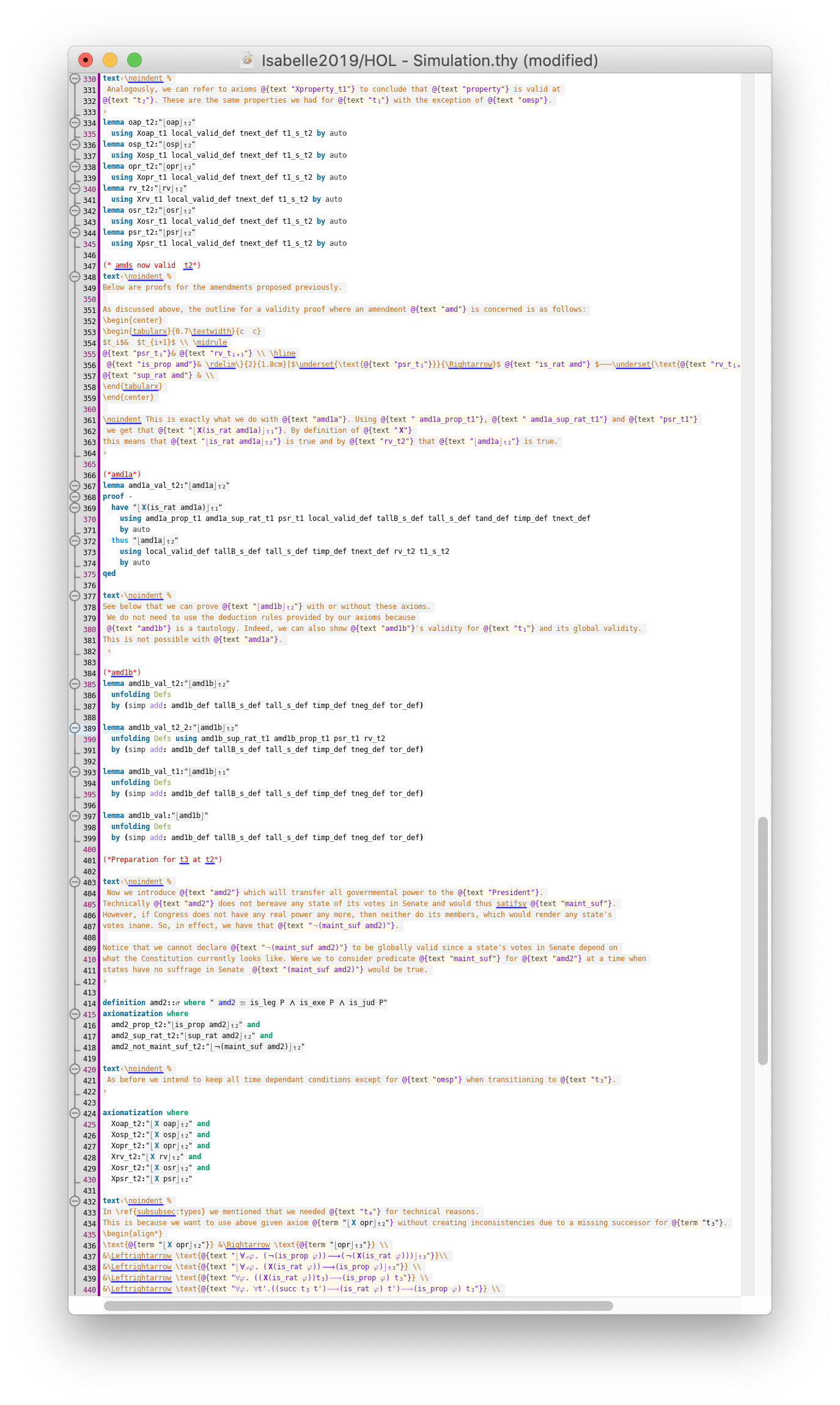

Supplement: Supplementary file 1 [file mmc1.zip › 2020-DataInBrief-Data/US-Constitution-Loophole/Simulation4.png]

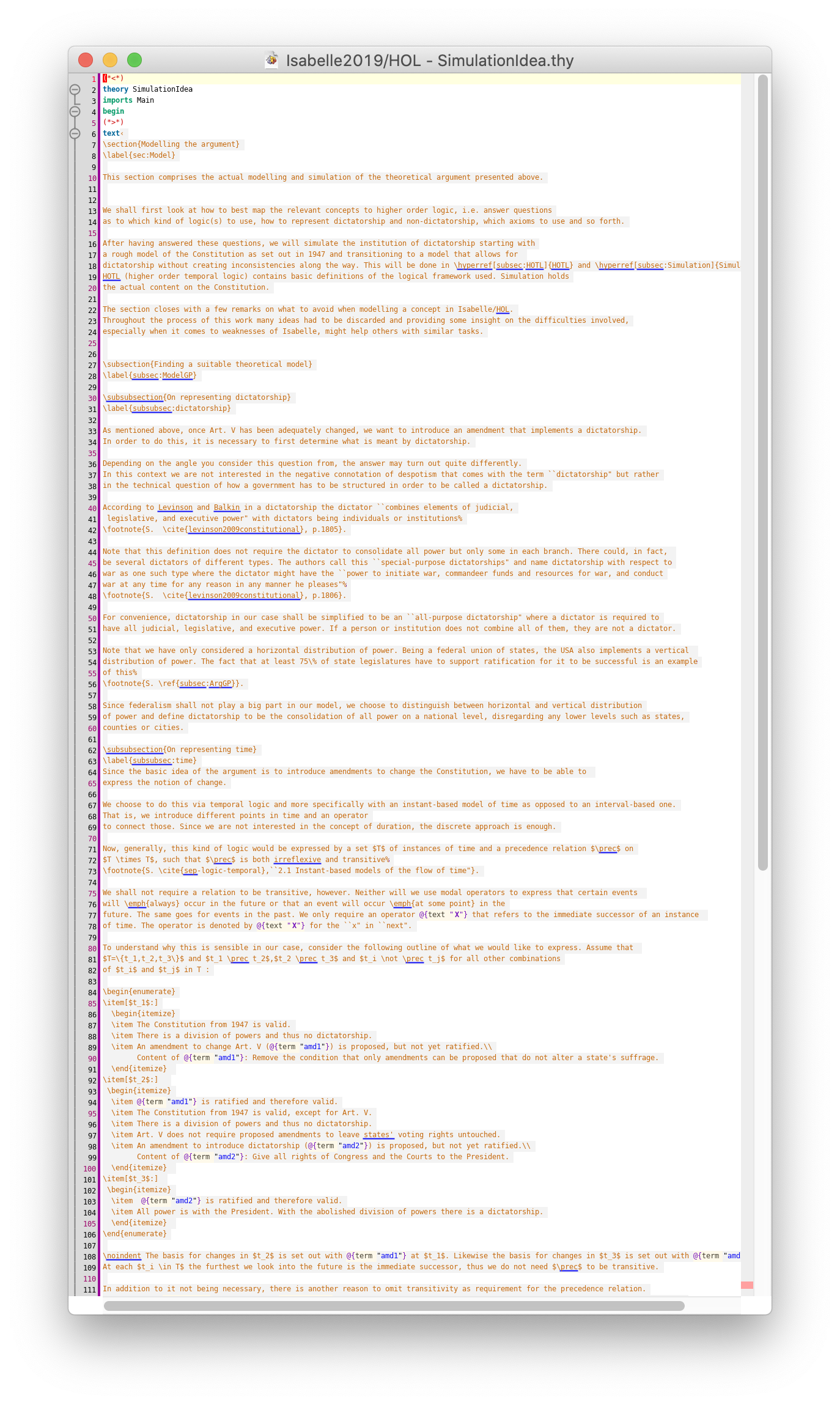

Supplement: Supplementary file 1 [file mmc1.zip › 2020-DataInBrief-Data/US-Constitution-Loophole/SimulationIdea1.png]

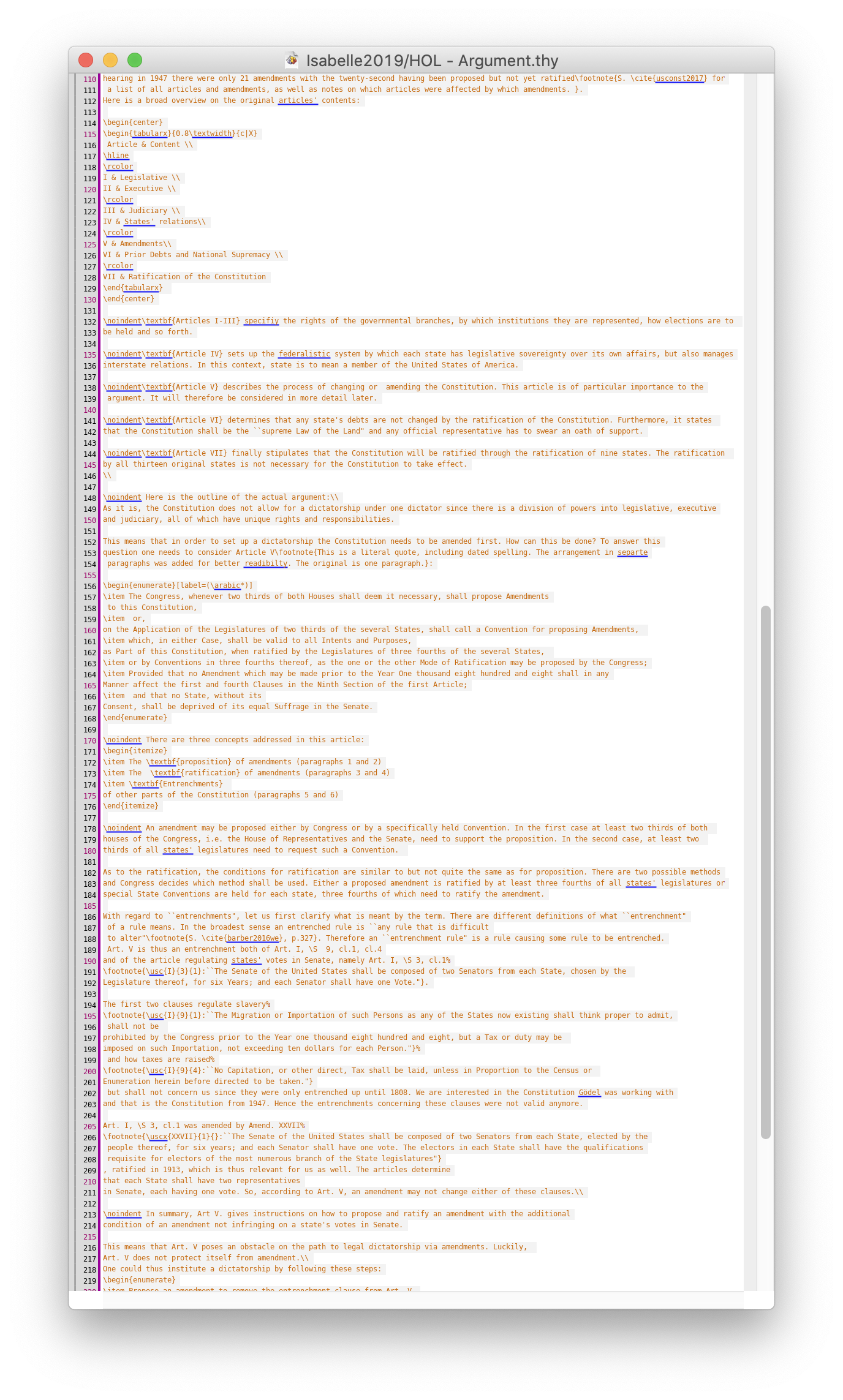

Supplement: Supplementary file 1 [file mmc1.zip › 2020-DataInBrief-Data/US-Constitution-Loophole/Argument2.png]

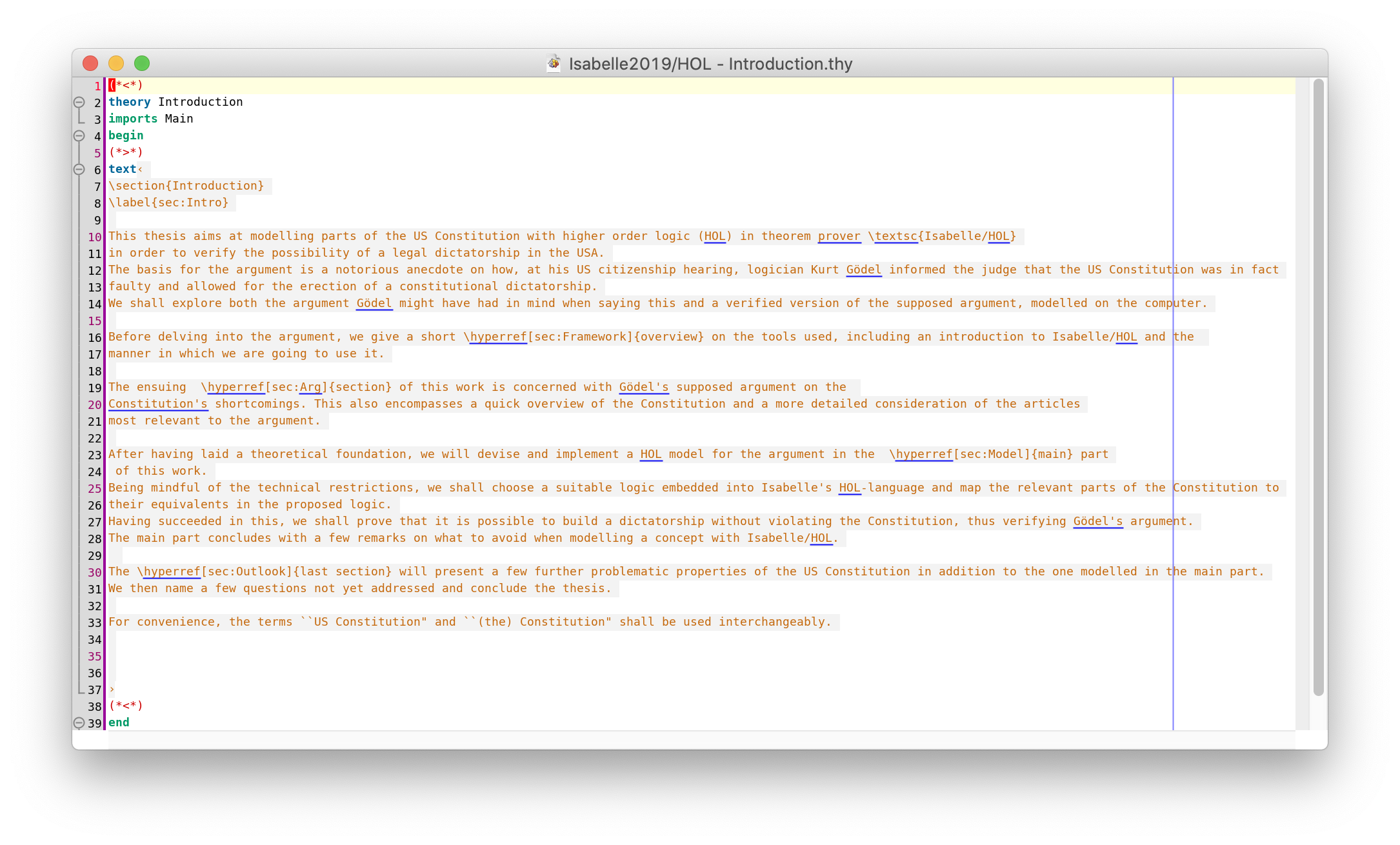

Supplement: Supplementary file 1 [file mmc1.zip › 2020-DataInBrief-Data/US-Constitution-Loophole/Introduction.png]

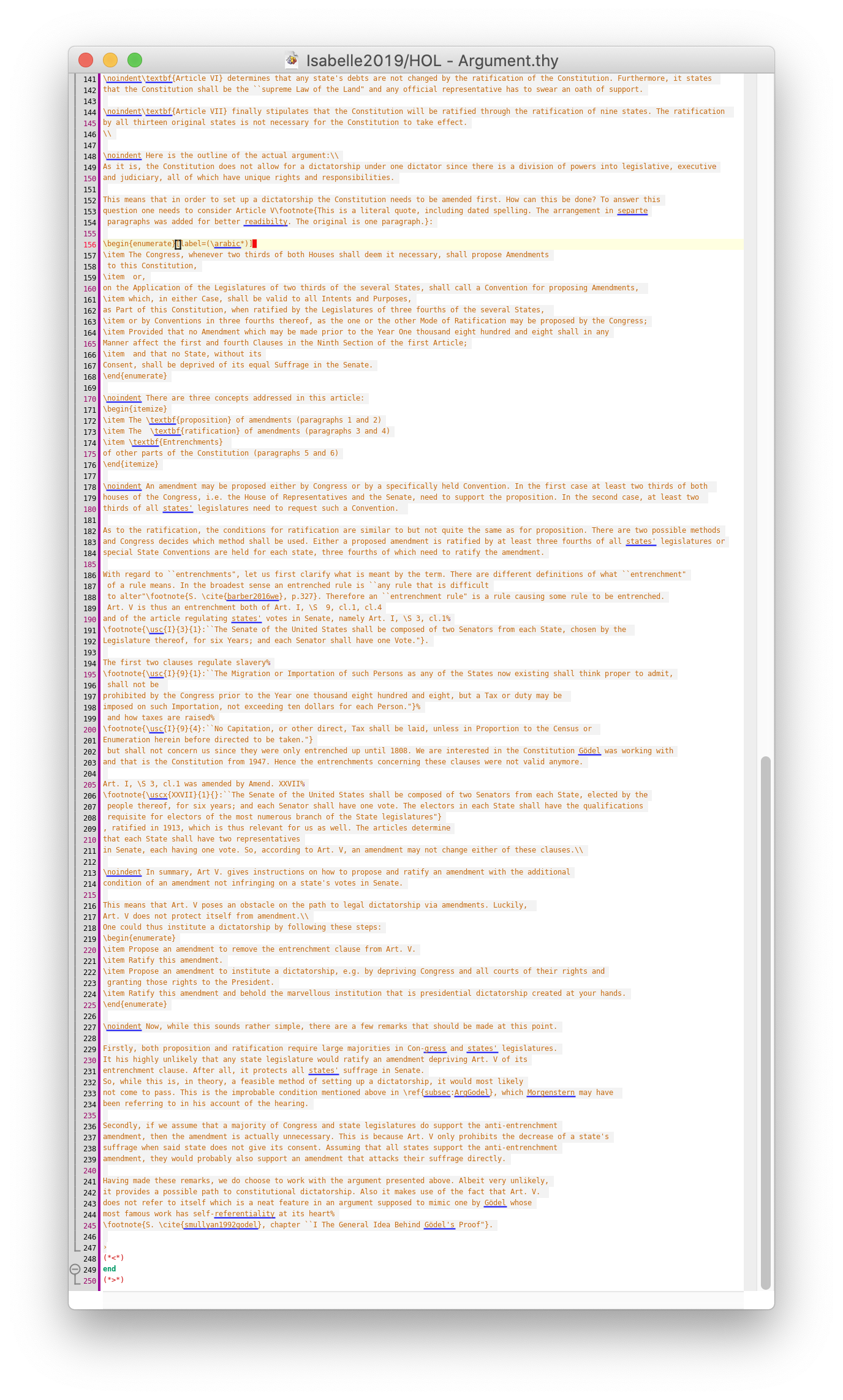

Supplement: Supplementary file 1 [file mmc1.zip › 2020-DataInBrief-Data/US-Constitution-Loophole/Argument3.png]

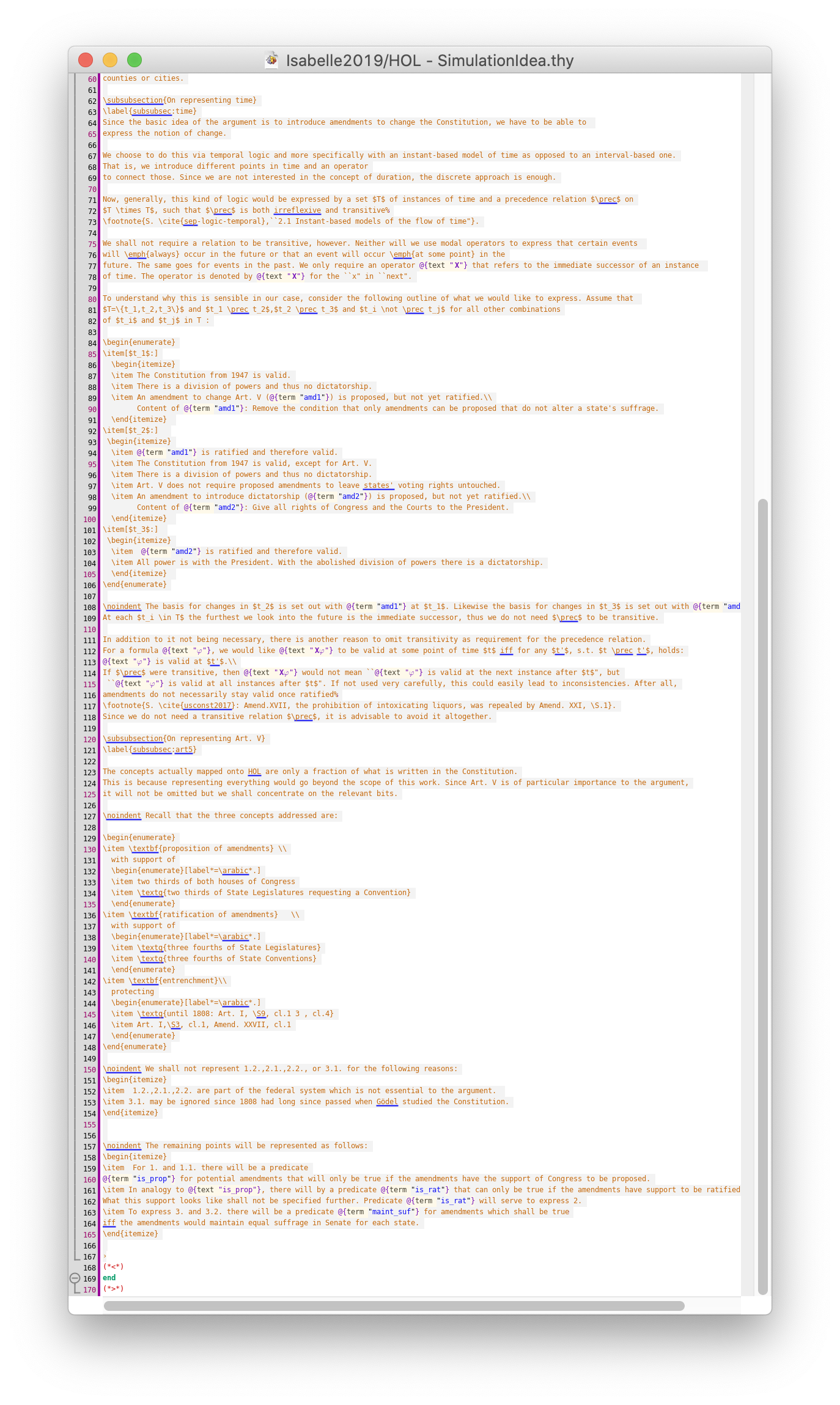

Supplement: Supplementary file 1 [file mmc1.zip › 2020-DataInBrief-Data/US-Constitution-Loophole/SimulationIdea2.png]

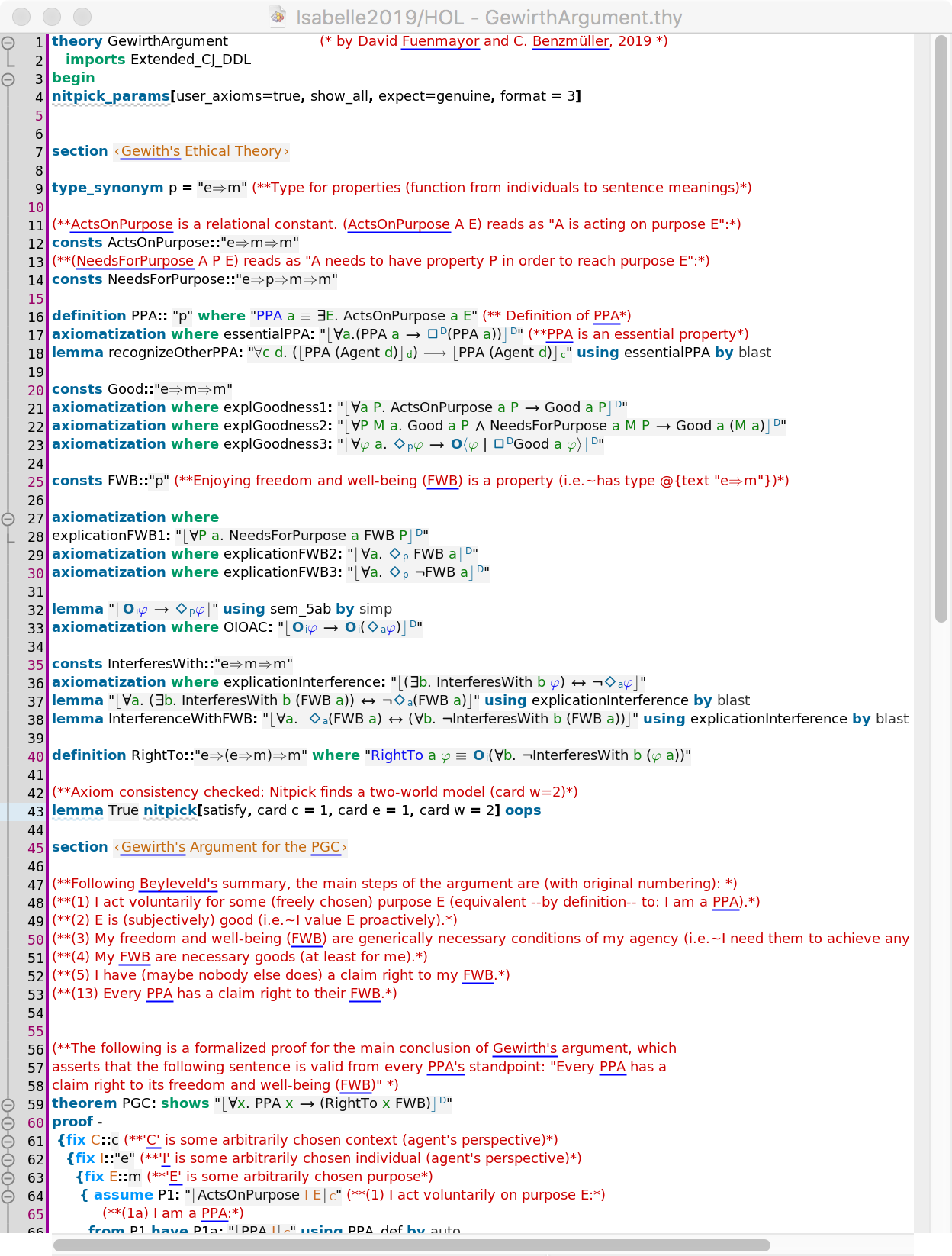

Supplement: Supplementary file 1 [file mmc1.zip › 2020-DataInBrief-Data/GewirthArgument1.png]

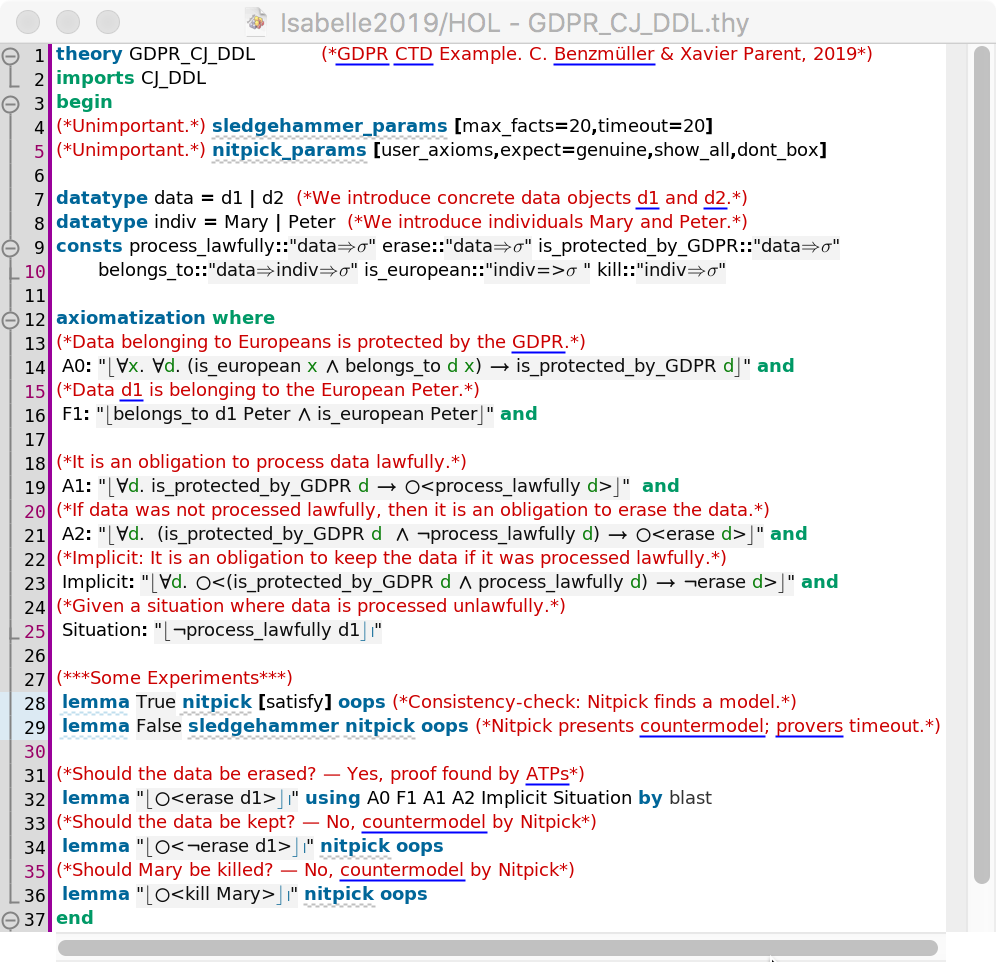

Supplement: Supplementary file 1 [file mmc1.zip › 2020-DataInBrief-Data/GDPR_CJ_DDL.png]

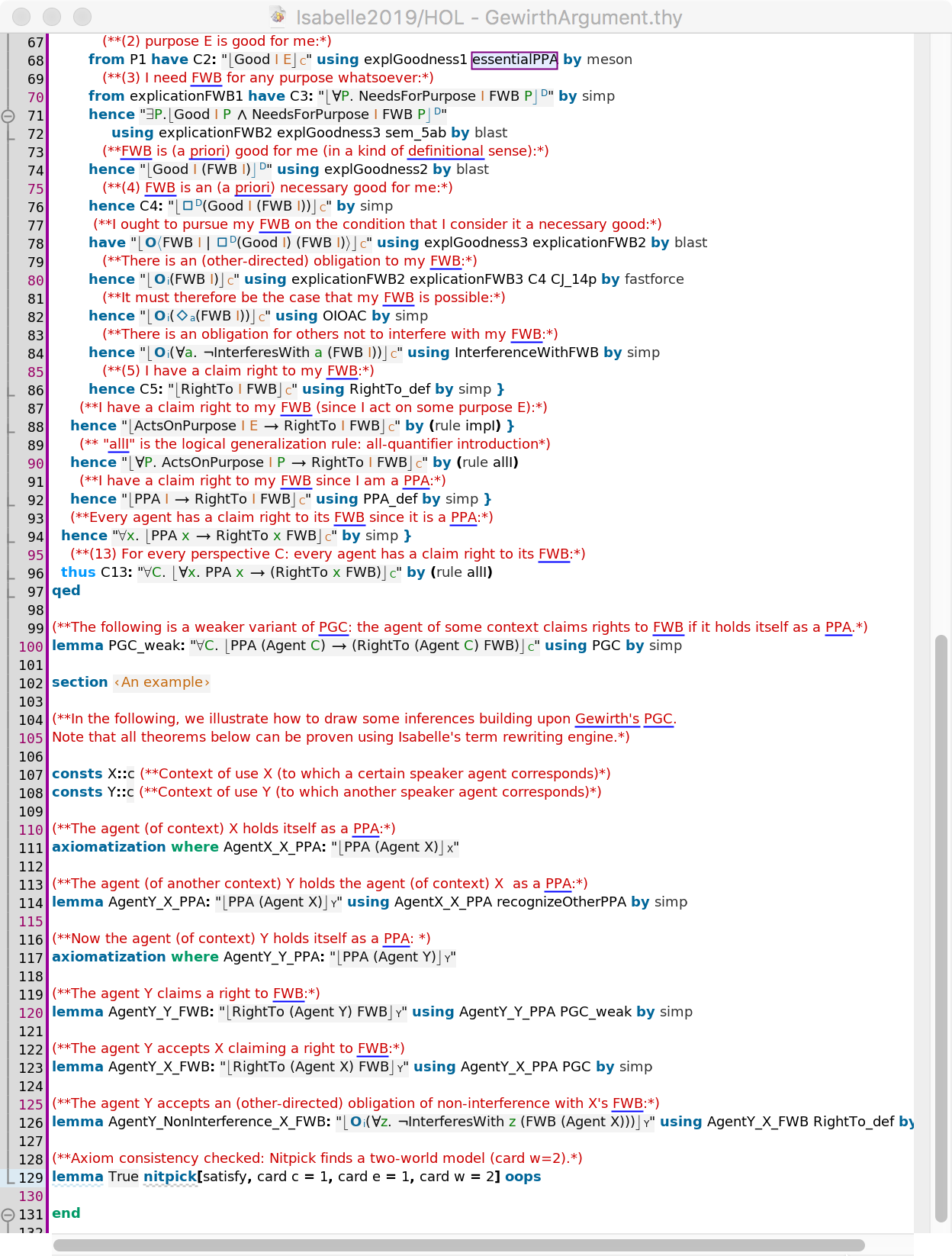

Supplement: Supplementary file 1 [file mmc1.zip › 2020-DataInBrief-Data/GewirthArgument2.png]

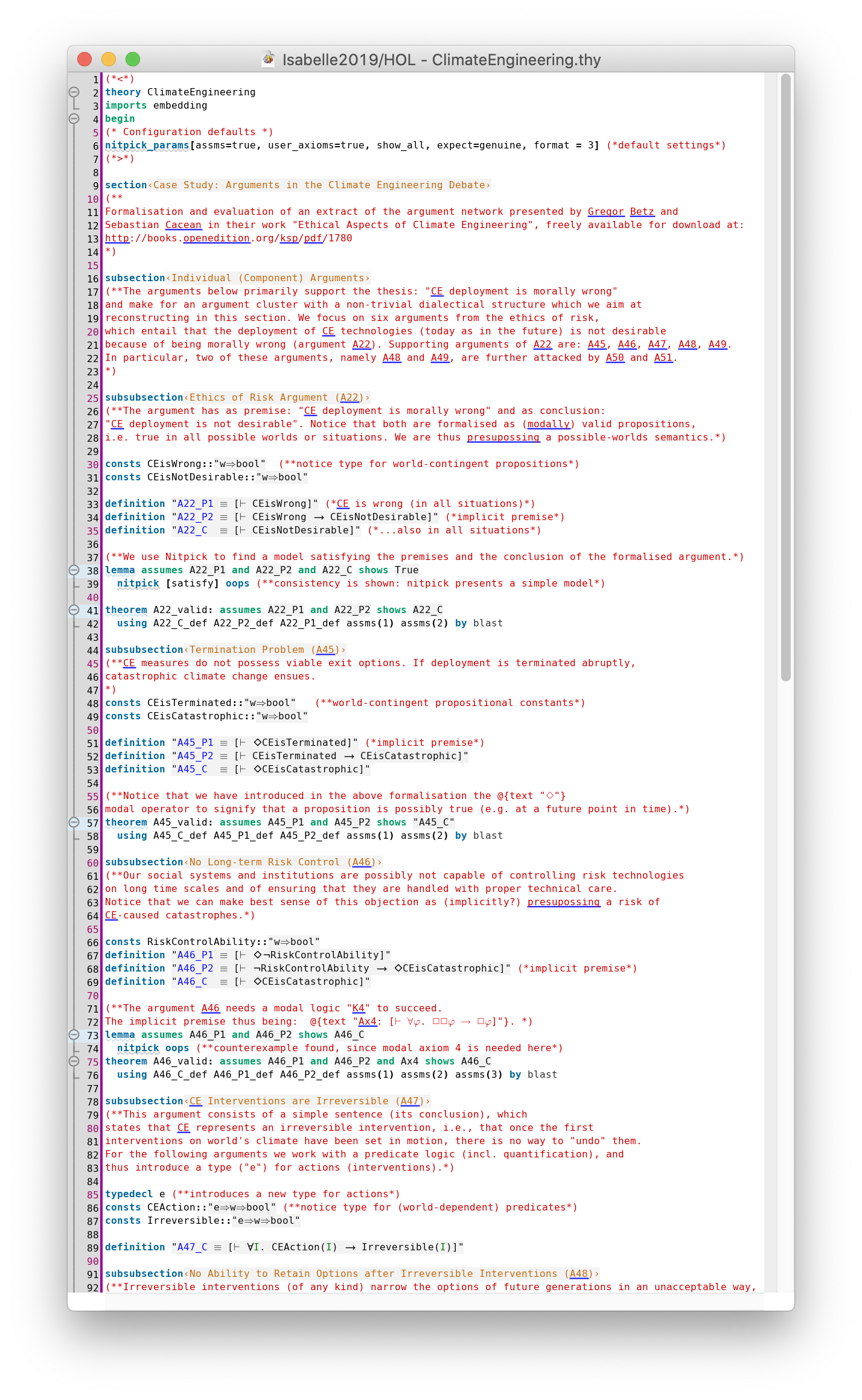

Supplement: Supplementary file 1 [file mmc1.zip › 2020-DataInBrief-Data/Climate-Engineering/ClimateEngineering1.png]

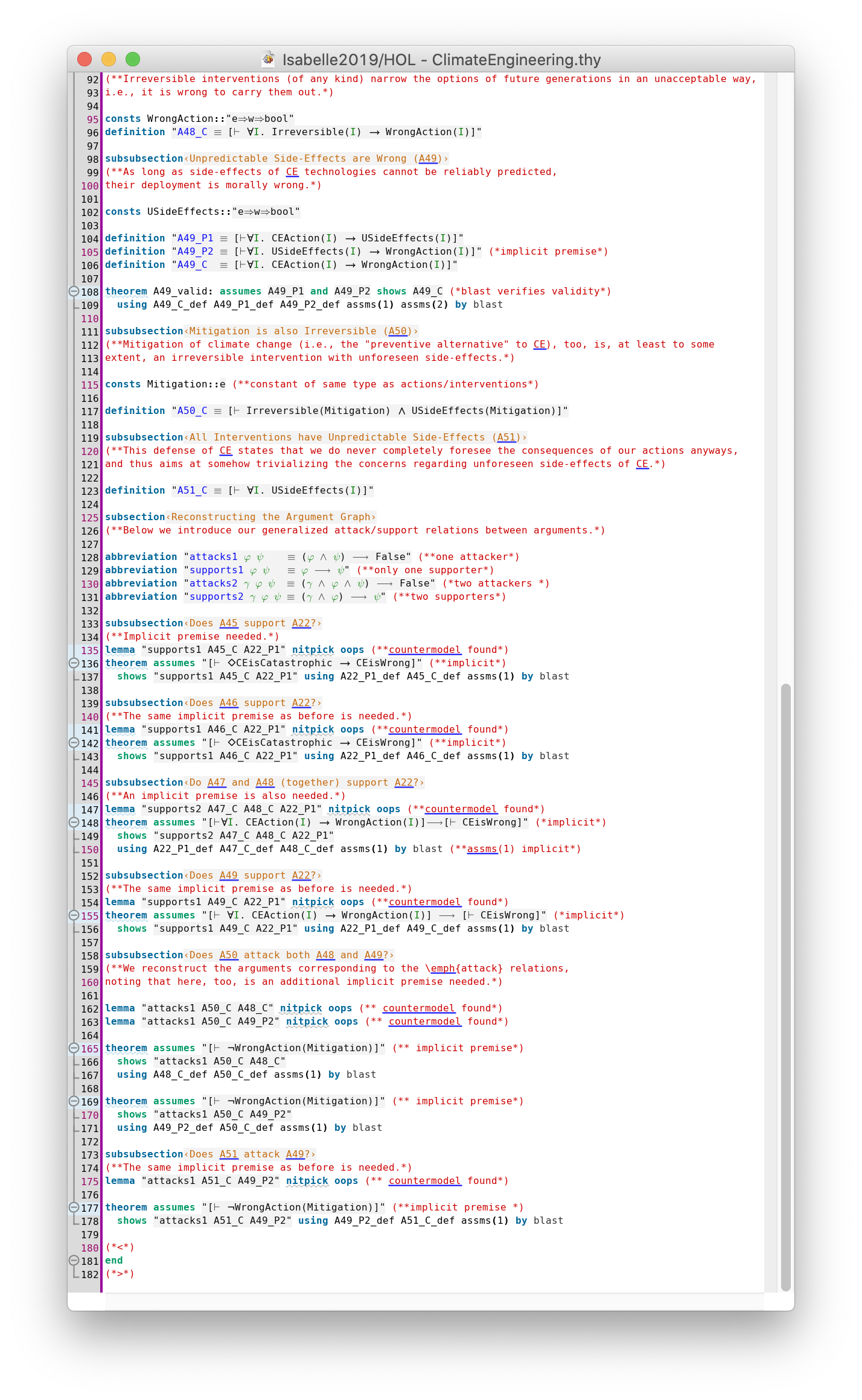

Supplement: Supplementary file 1 [file mmc1.zip › 2020-DataInBrief-Data/Climate-Engineering/ClimateEngineering2.png]

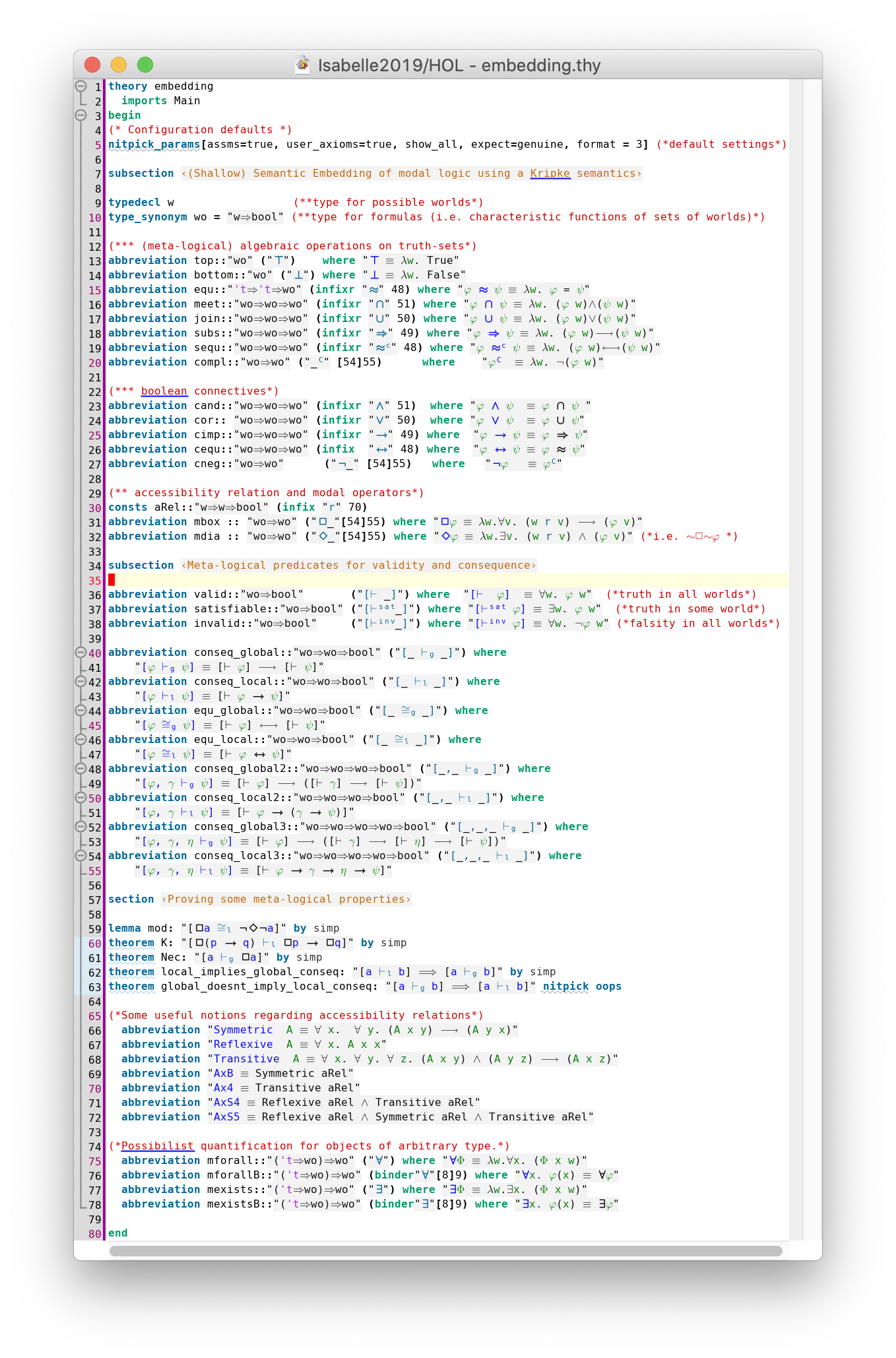

Supplement: Supplementary file 1 [file mmc1.zip › 2020-DataInBrief-Data/Climate-Engineering/embedding.png]

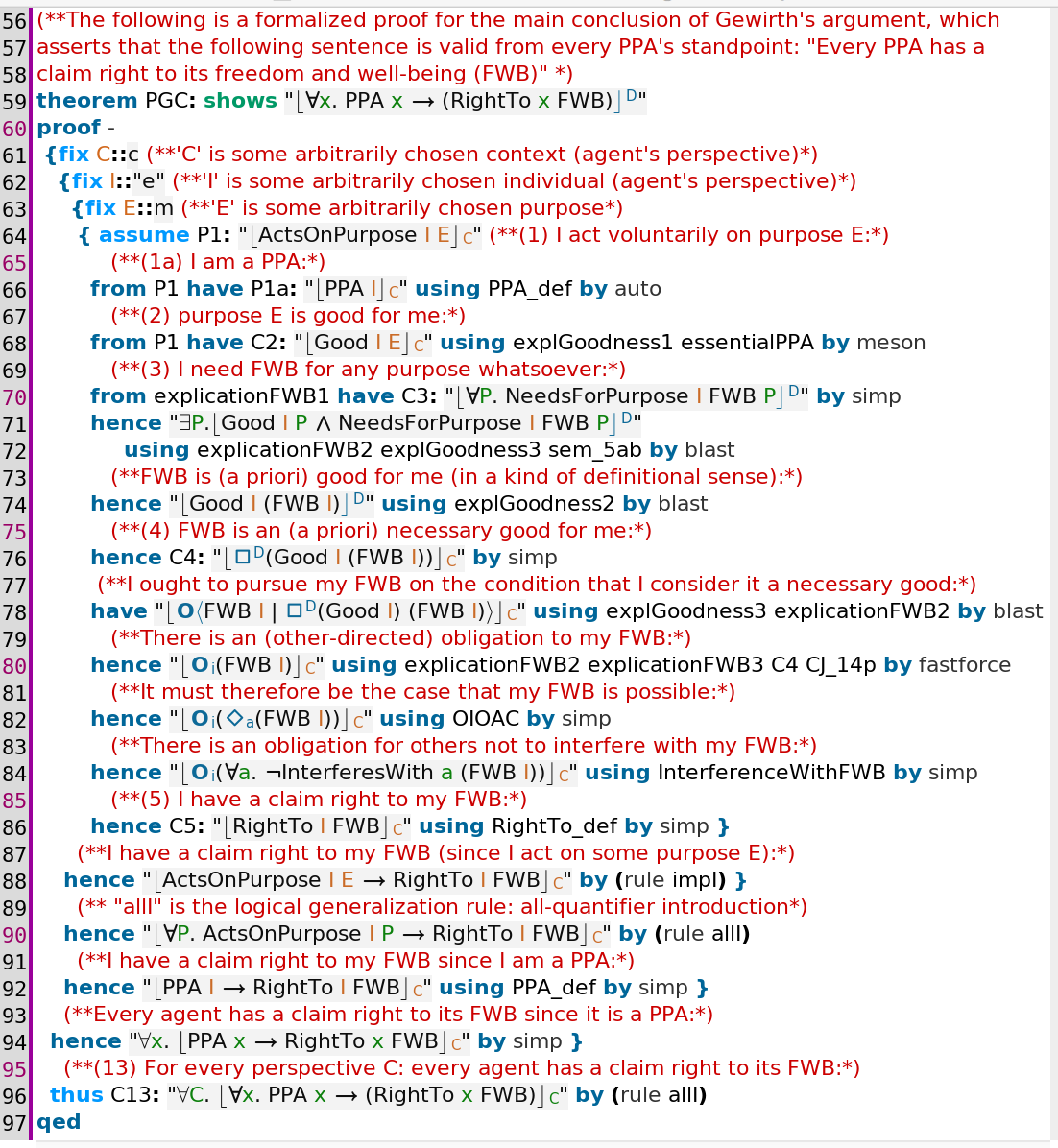

Supplement: Supplementary file 1 [file mmc1.zip › 2020-DataInBrief-Data/GewirthArgument.png]

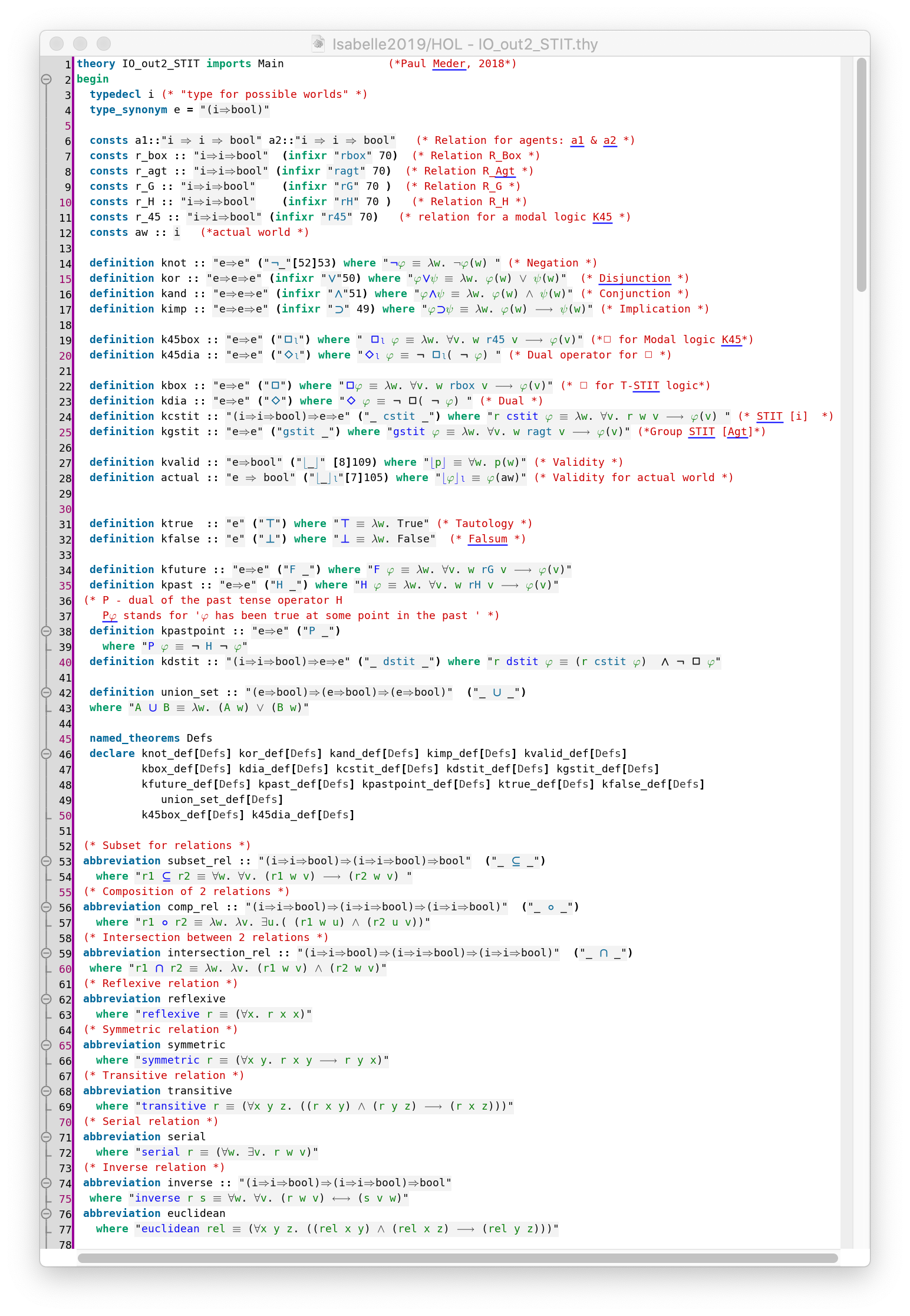

Supplement: Supplementary file 1 [file mmc1.zip › 2020-DataInBrief-Data/IO_out2_STIT1.png]

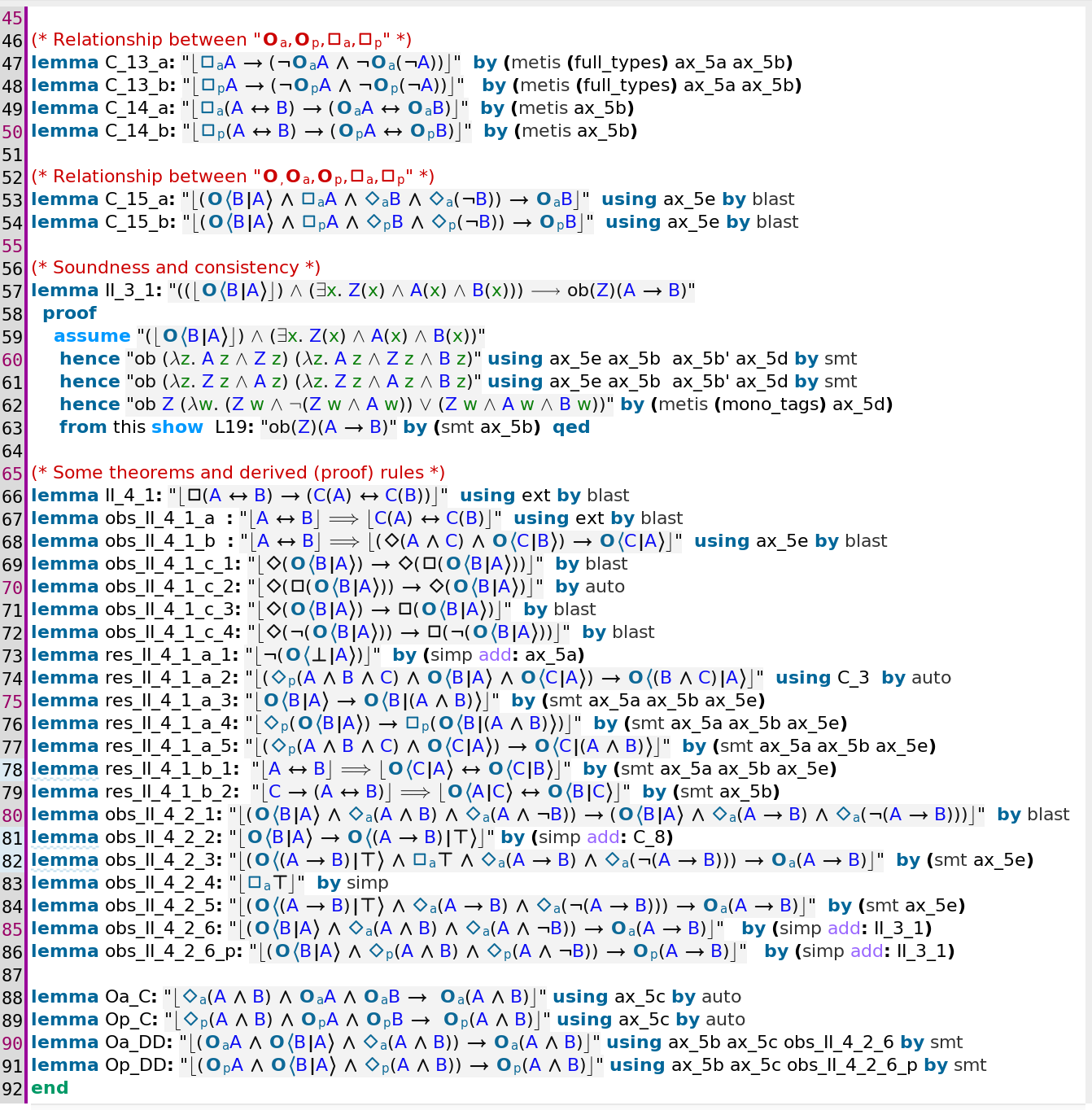

Supplement: Supplementary file 1 [file mmc1.zip › 2020-DataInBrief-Data/CJ_DDL_Tests2.png]

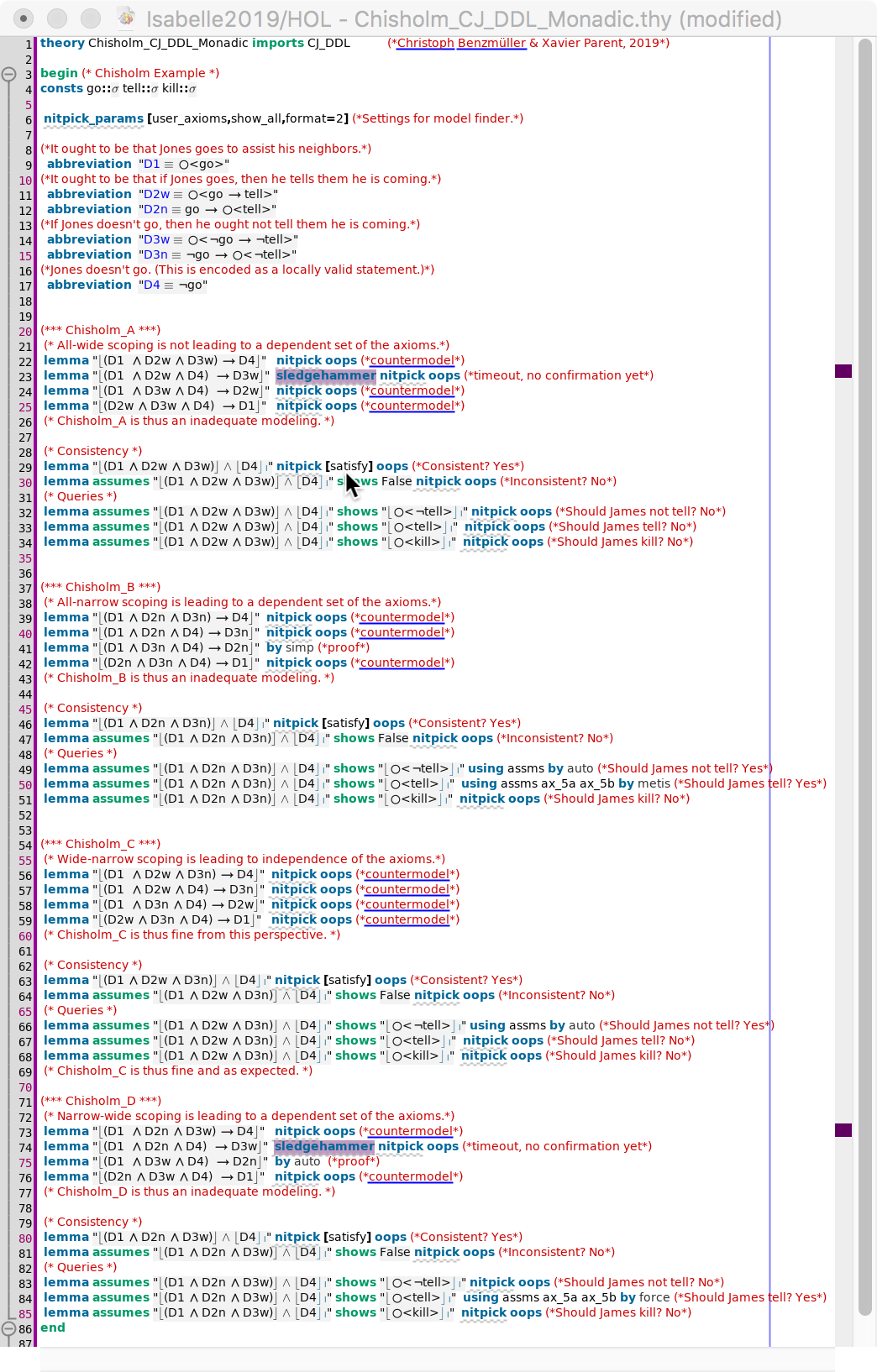

Supplement: Supplementary file 1 [file mmc1.zip › 2020-DataInBrief-Data/Chisholm_CJ_DDL_Monadic.png]

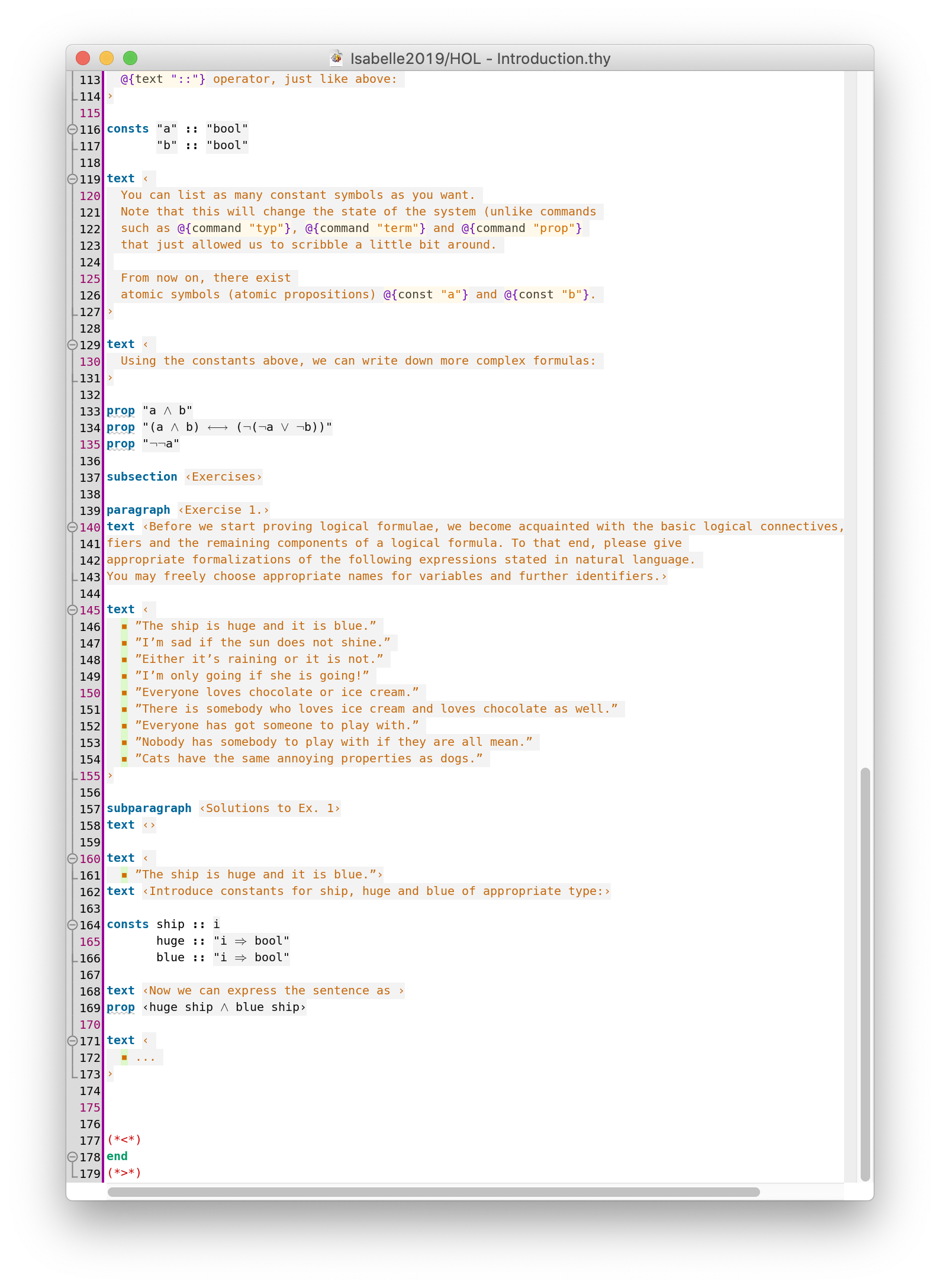

Supplement: Supplementary file 1 [file mmc1.zip › 2020-DataInBrief-Data/Course-Material-1/Introduction3.png]

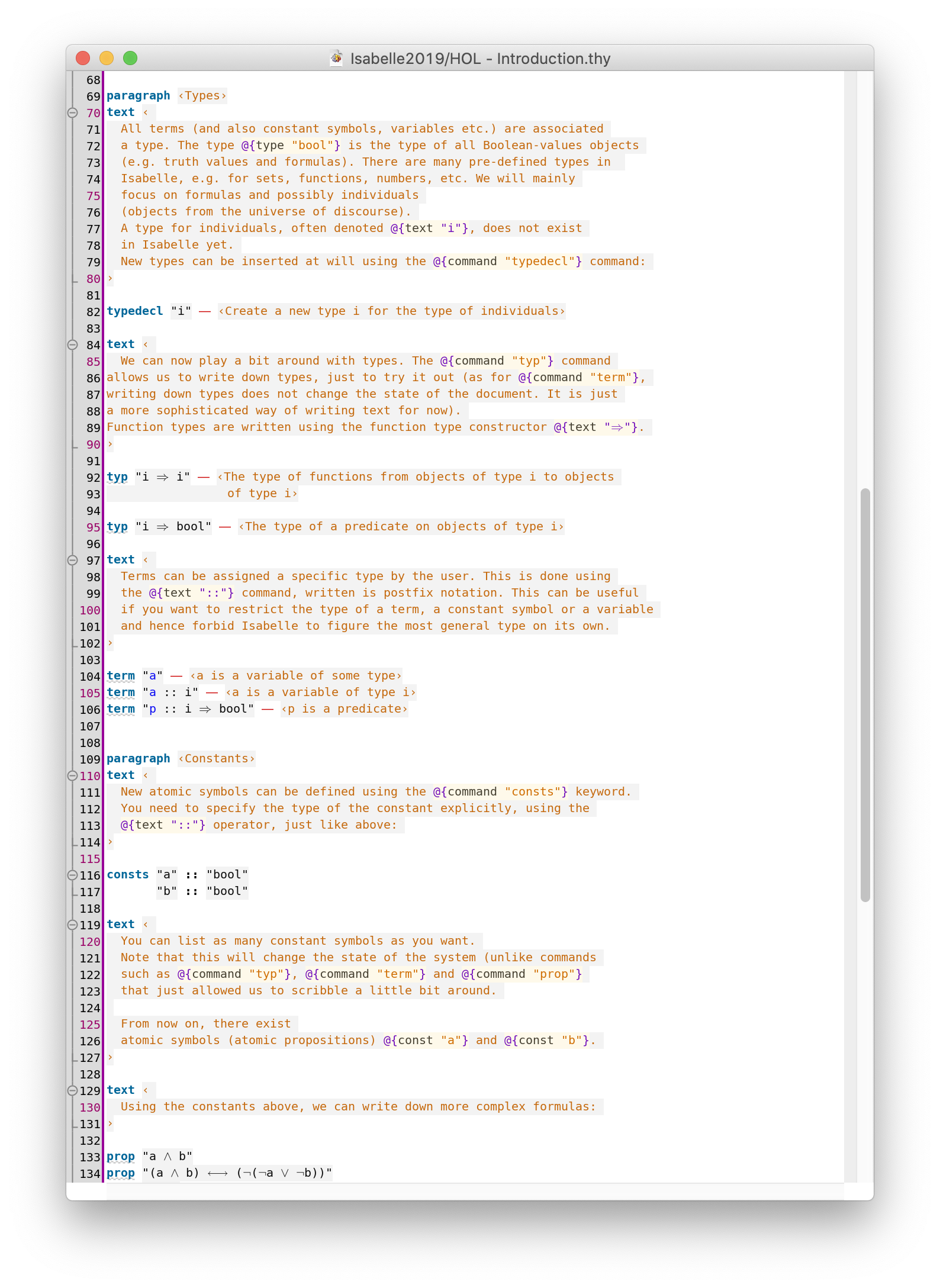

Supplement: Supplementary file 1 [file mmc1.zip › 2020-DataInBrief-Data/Course-Material-1/Introduction2.png]

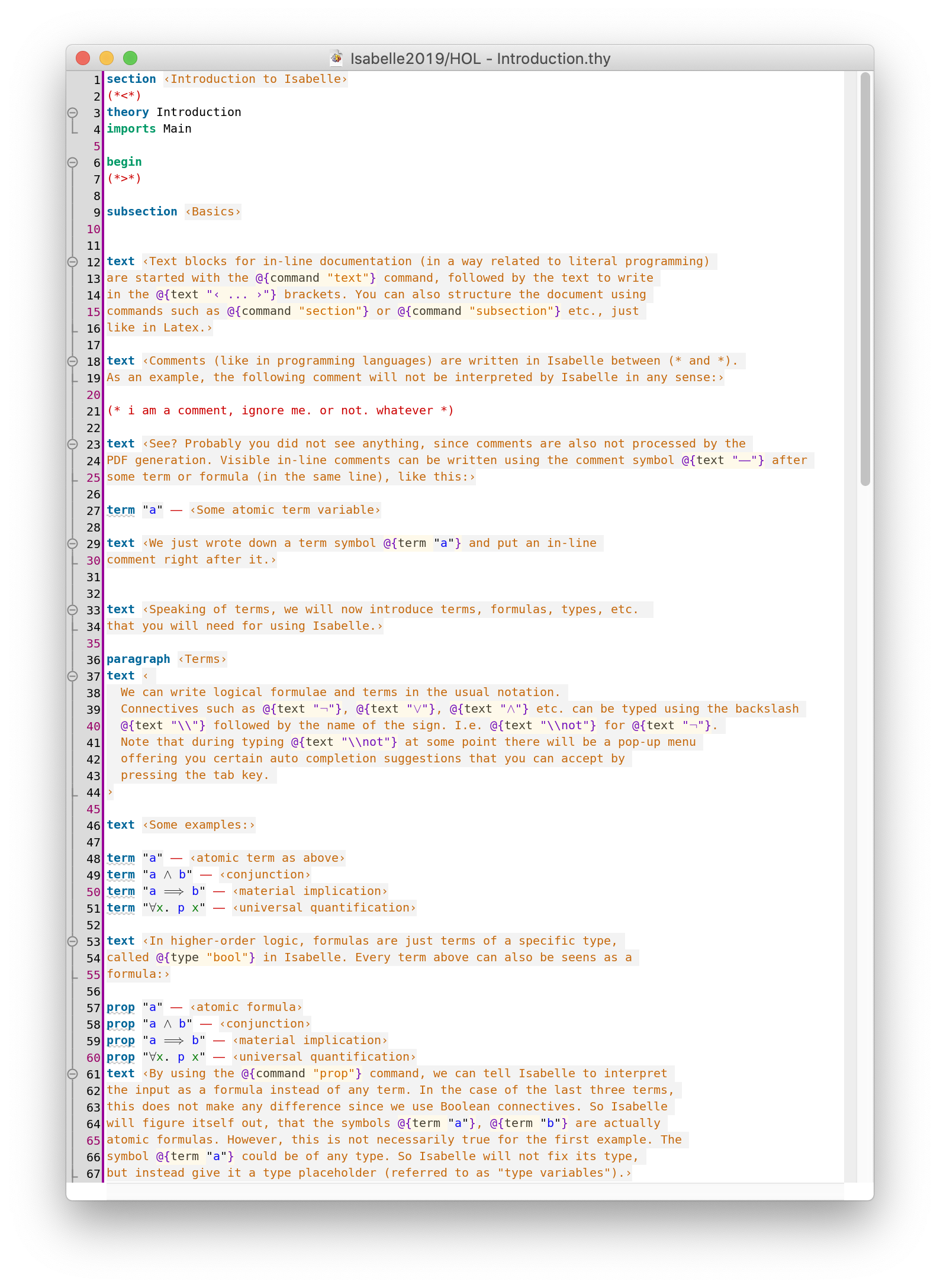

Supplement: Supplementary file 1 [file mmc1.zip › 2020-DataInBrief-Data/Course-Material-1/Introduction1.png]

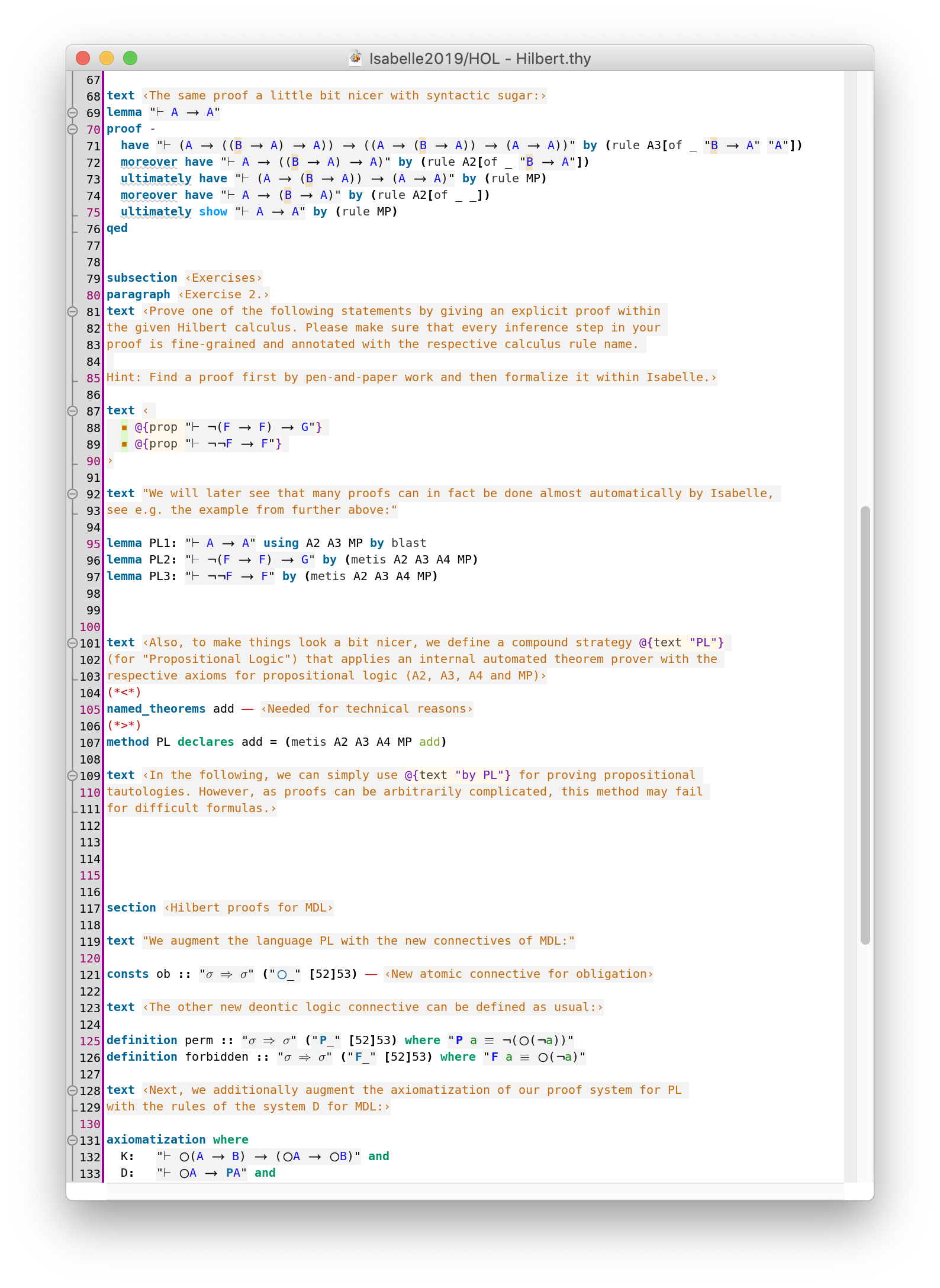

Supplement: Supplementary file 1 [file mmc1.zip › 2020-DataInBrief-Data/Course-Material-1/Hilbert2.png]

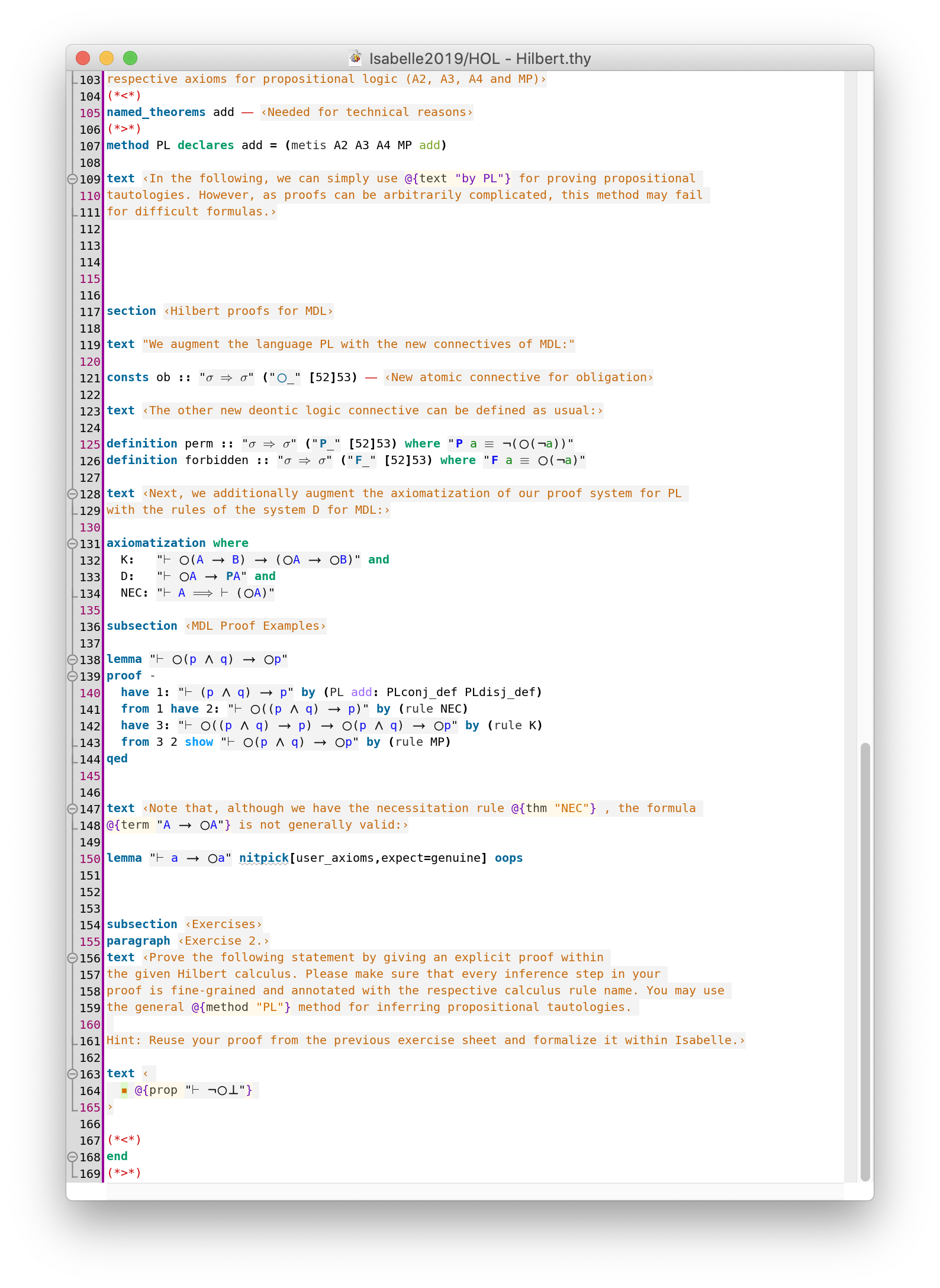

Supplement: Supplementary file 1 [file mmc1.zip › 2020-DataInBrief-Data/Course-Material-1/Hilbert3.png]

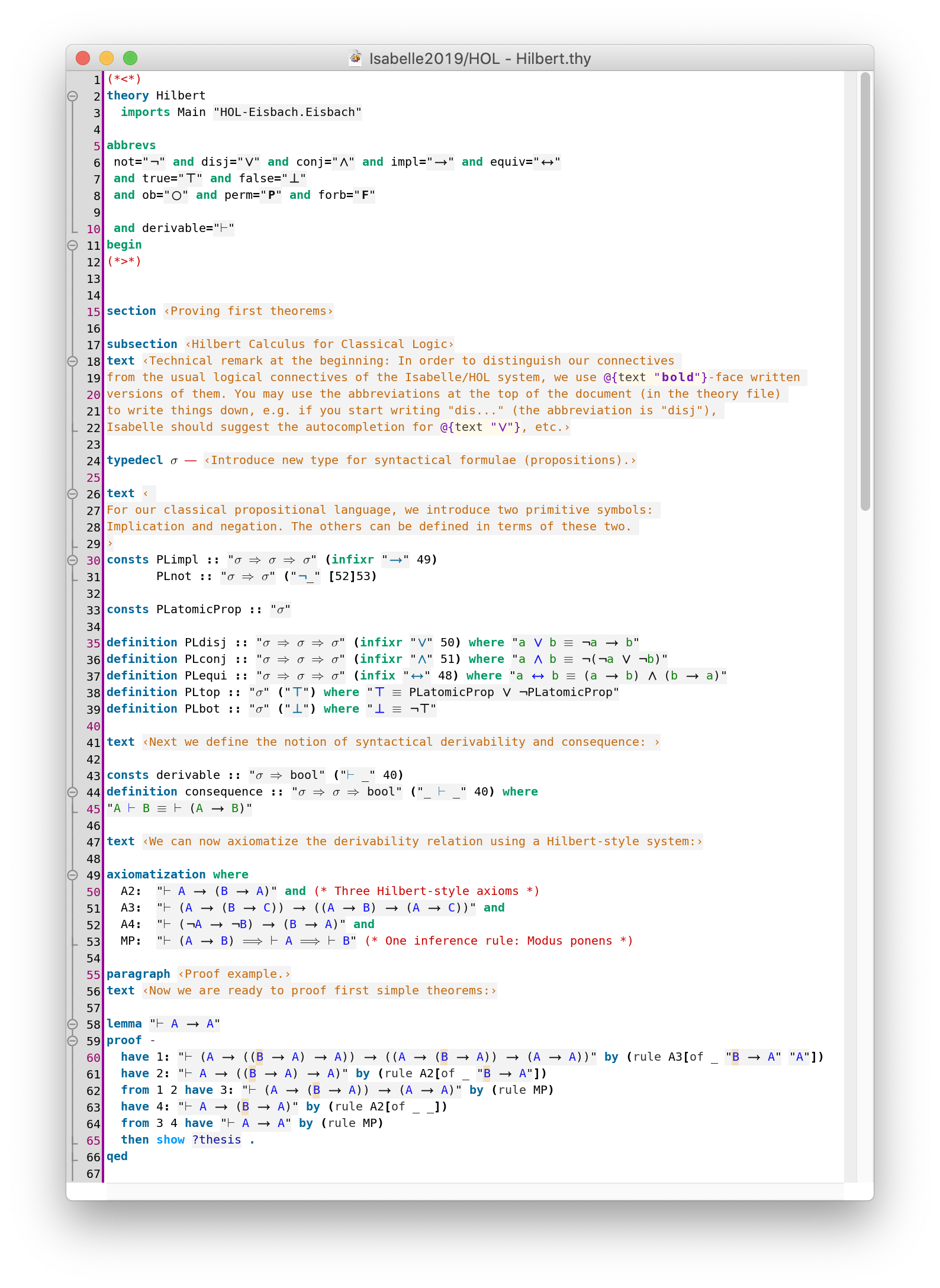

Supplement: Supplementary file 1 [file mmc1.zip › 2020-DataInBrief-Data/Course-Material-1/Hilbert1.png]

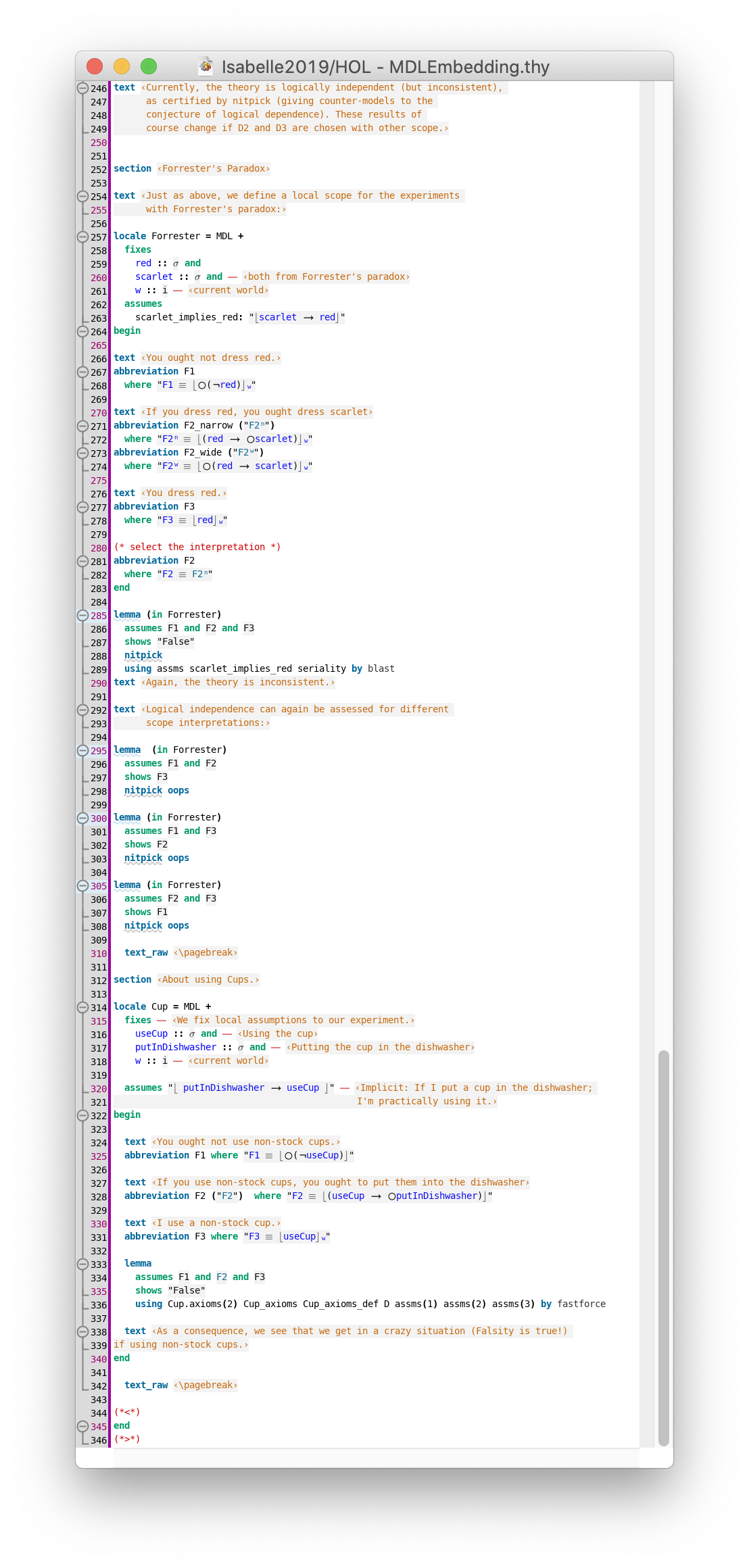

Supplement: Supplementary file 1 [file mmc1.zip › 2020-DataInBrief-Data/Course-Material-1/MDLEmbedding4.png]

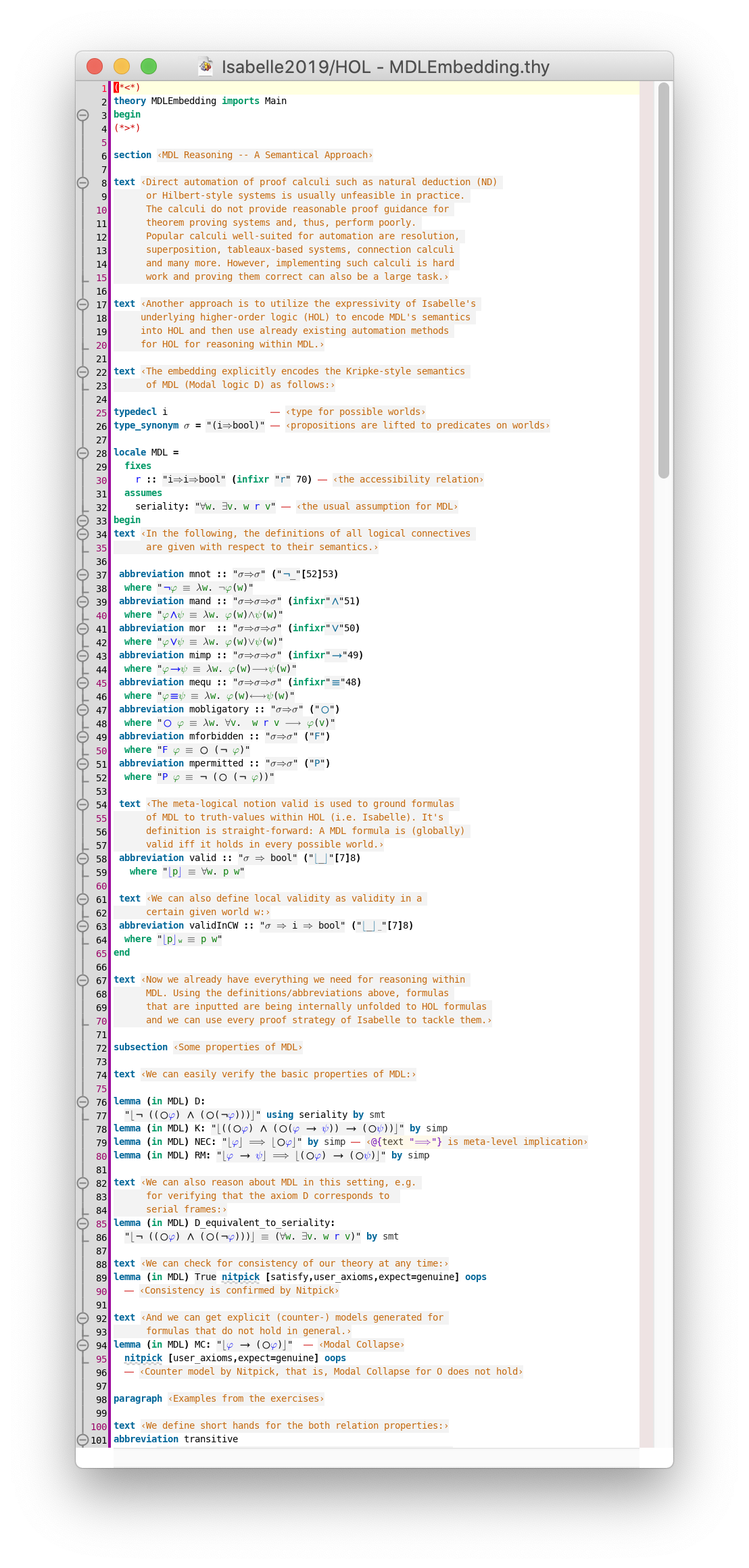

Supplement: Supplementary file 1 [file mmc1.zip › 2020-DataInBrief-Data/Course-Material-1/MDLEmbedding1.png]

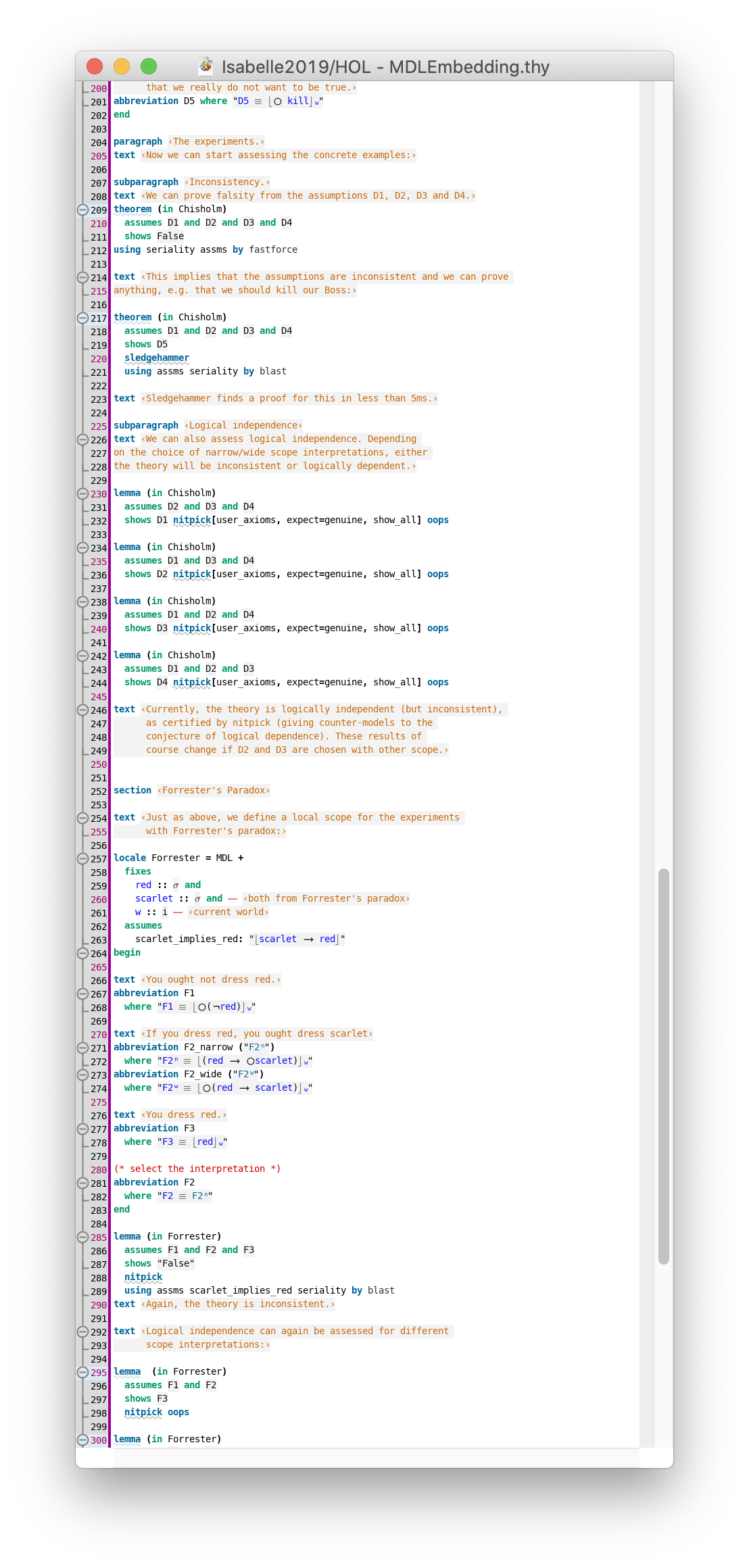

Supplement: Supplementary file 1 [file mmc1.zip › 2020-DataInBrief-Data/Course-Material-1/MDLEmbedding3.png]

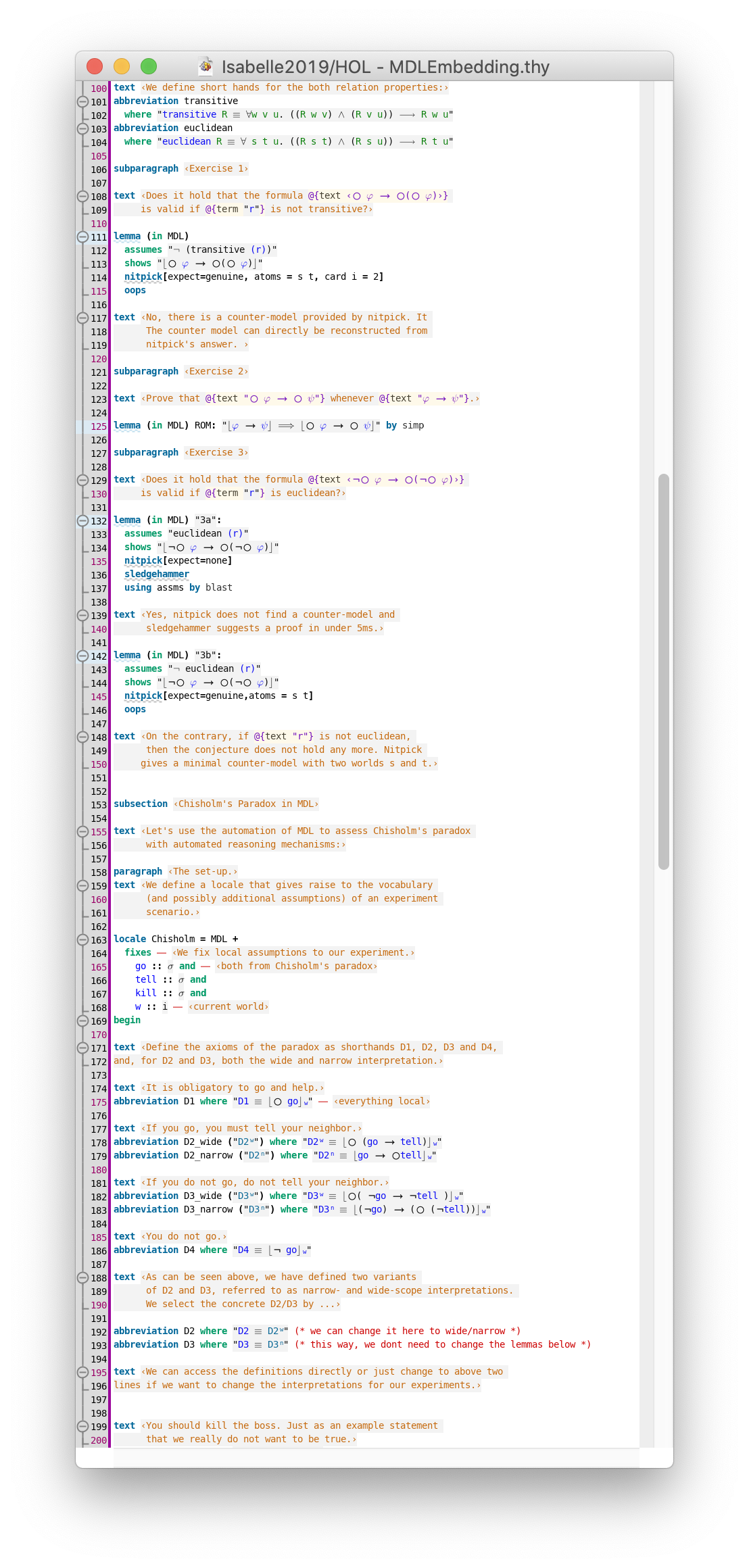

Supplement: Supplementary file 1 [file mmc1.zip › 2020-DataInBrief-Data/Course-Material-1/MDLEmbedding2.png]

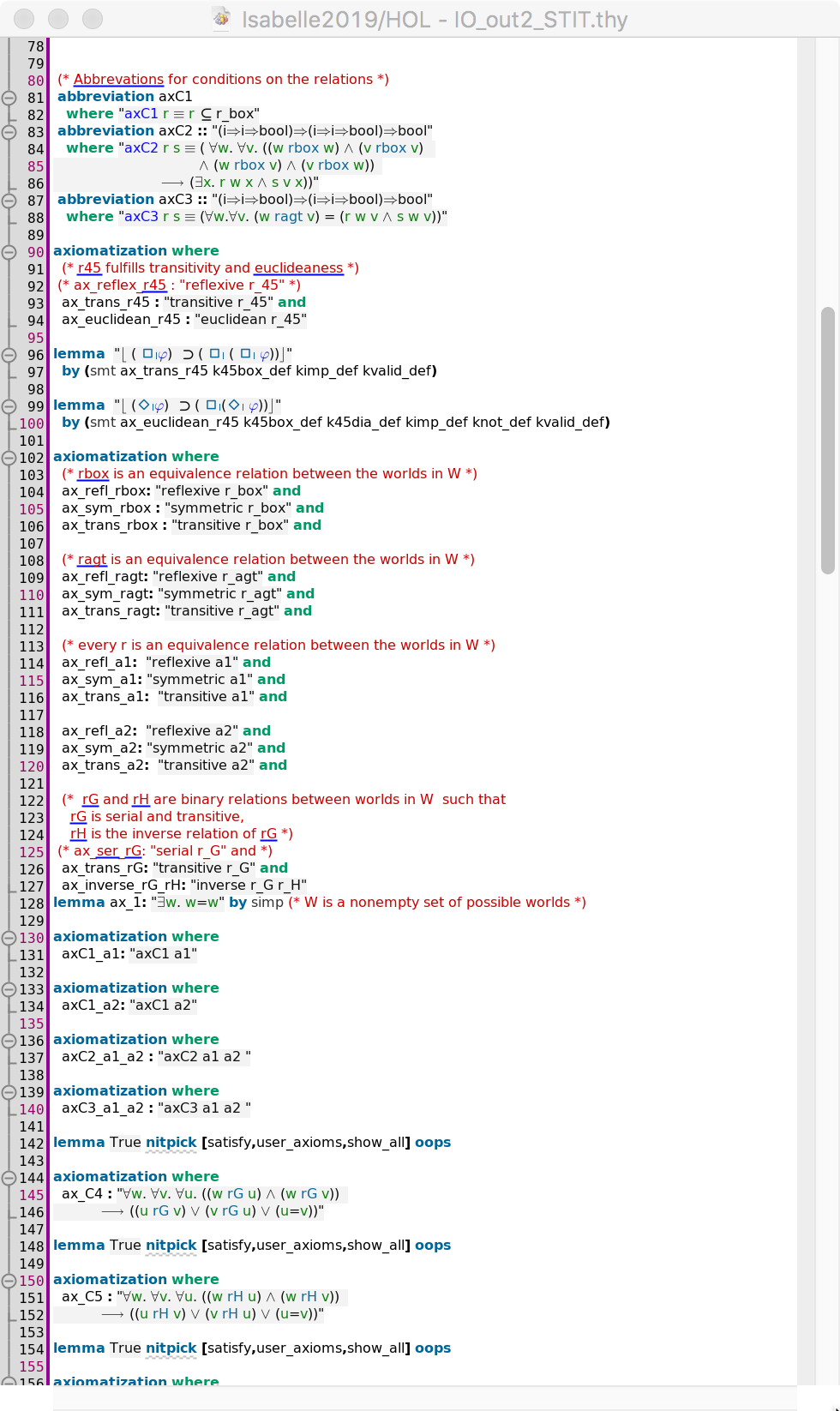

Supplement: Supplementary file 1 [file mmc1.zip › 2020-DataInBrief-Data/IO_out2_STIT2.png]

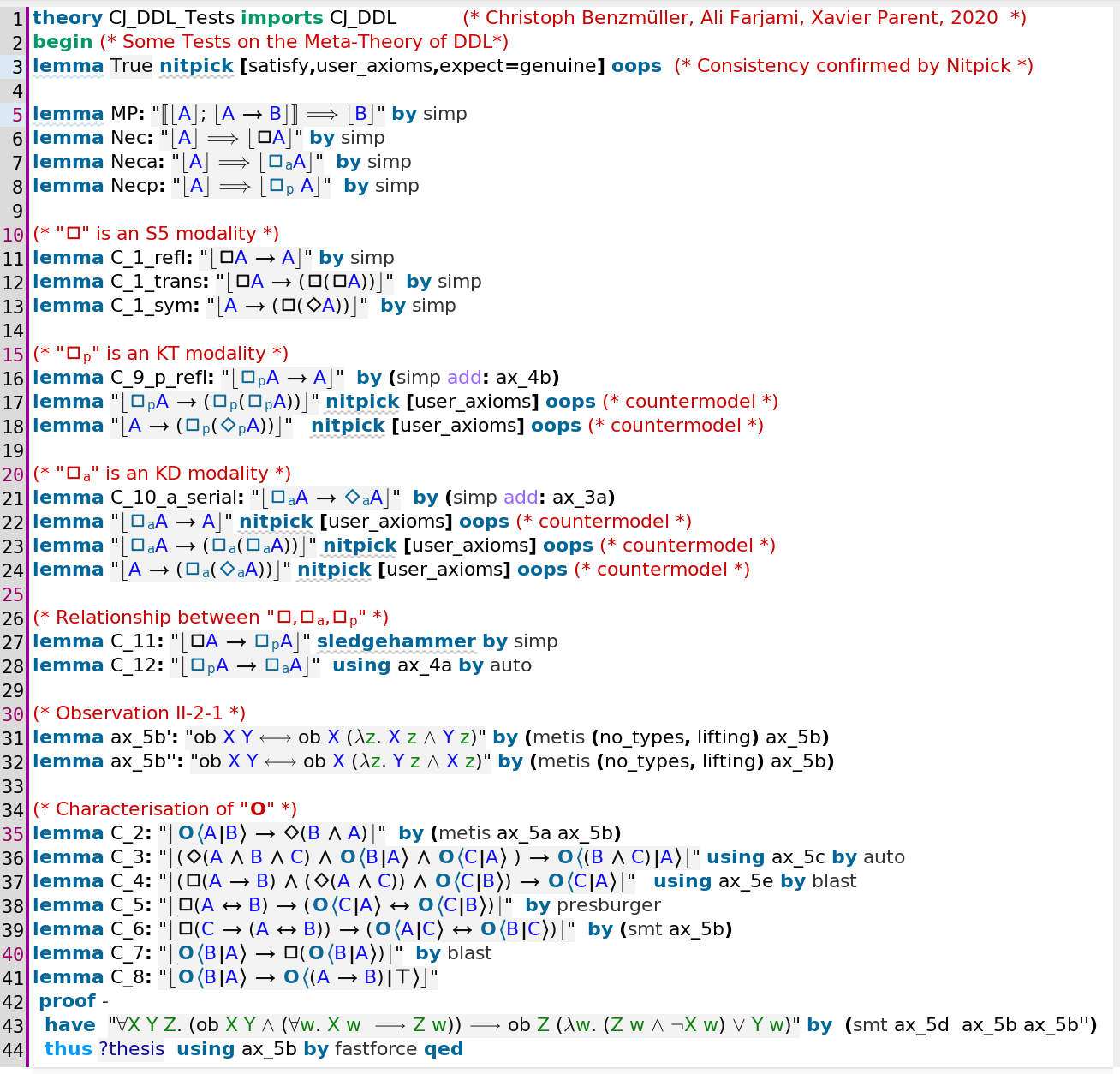

Supplement: Supplementary file 1 [file mmc1.zip › 2020-DataInBrief-Data/CJ_DDL_Tests1.png]

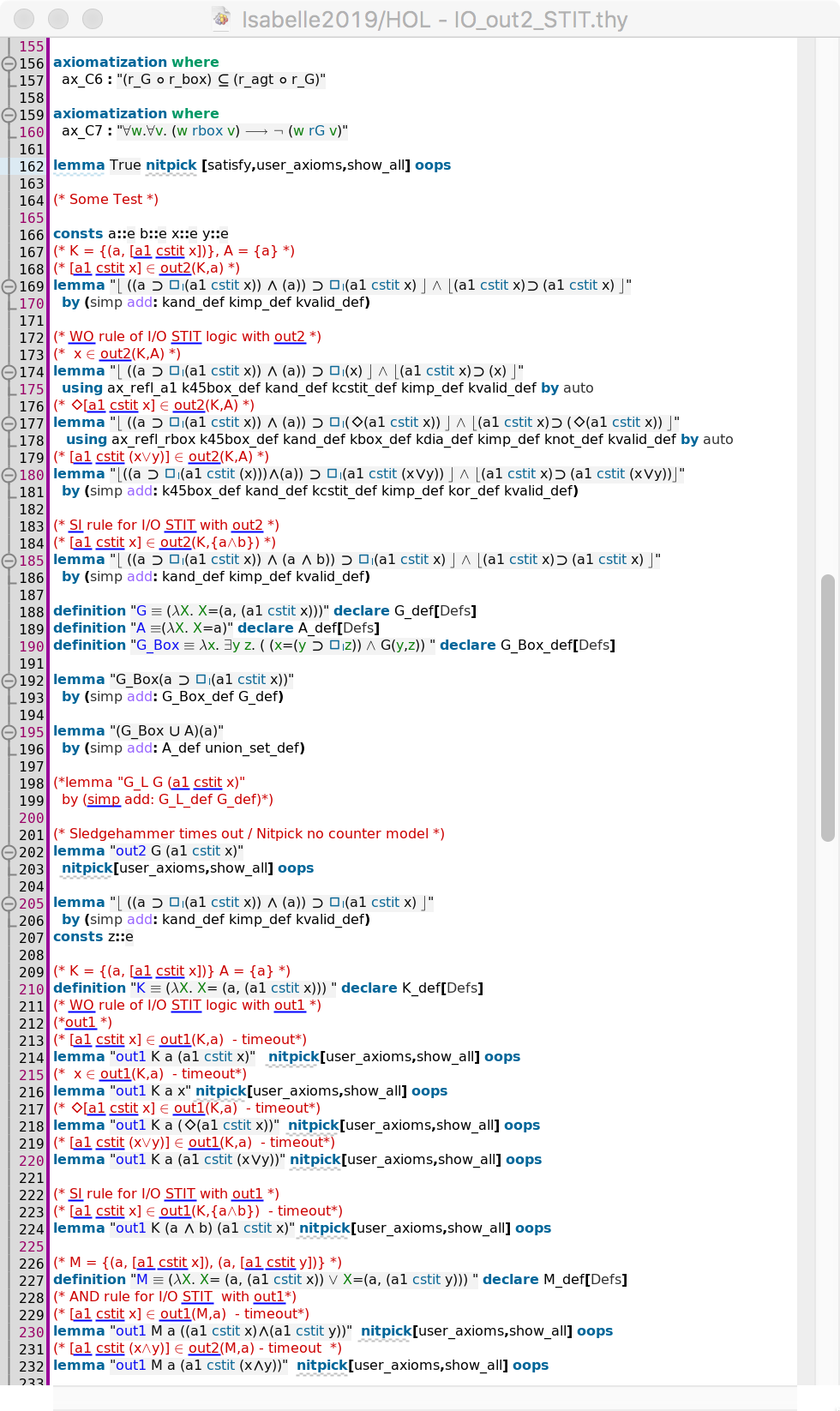

Supplement: Supplementary file 1 [file mmc1.zip › 2020-DataInBrief-Data/IO_out2_STIT3.png]

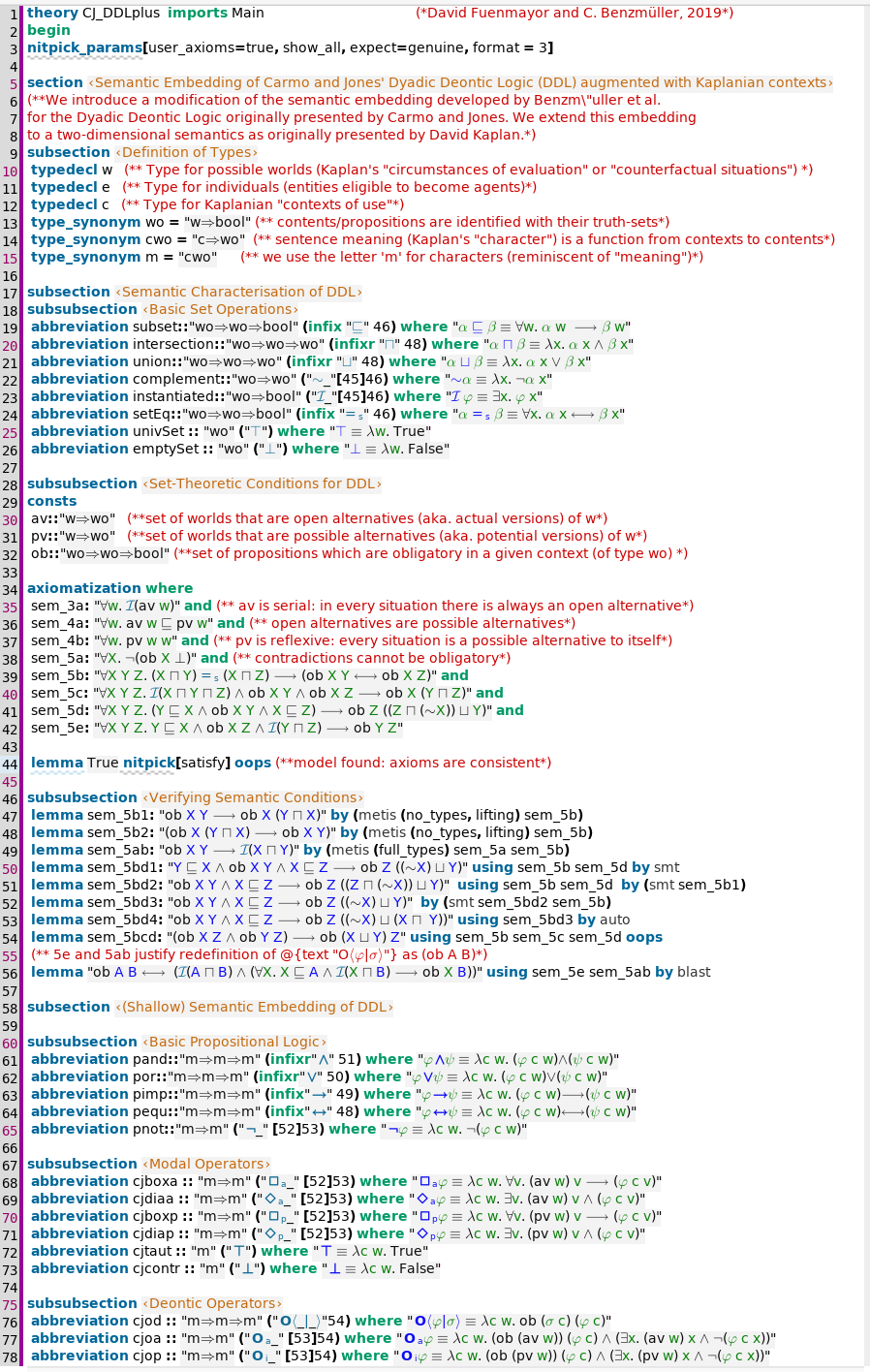

Supplement: Supplementary file 1 [file mmc1.zip › 2020-DataInBrief-Data/CJ_DDLplus1.png]

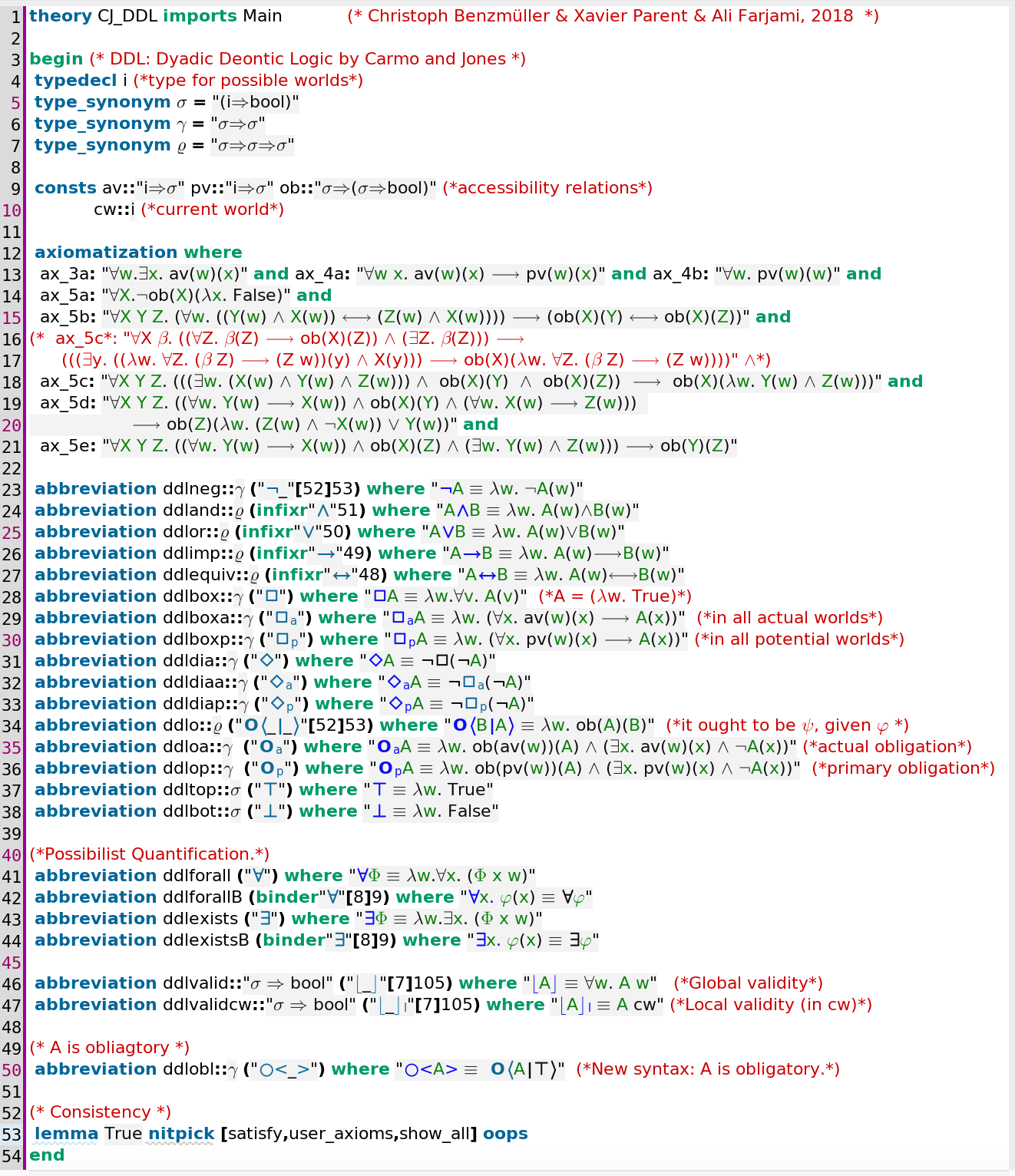

Supplement: Supplementary file 1 [file mmc1.zip › 2020-DataInBrief-Data/CJ_DDL.png]

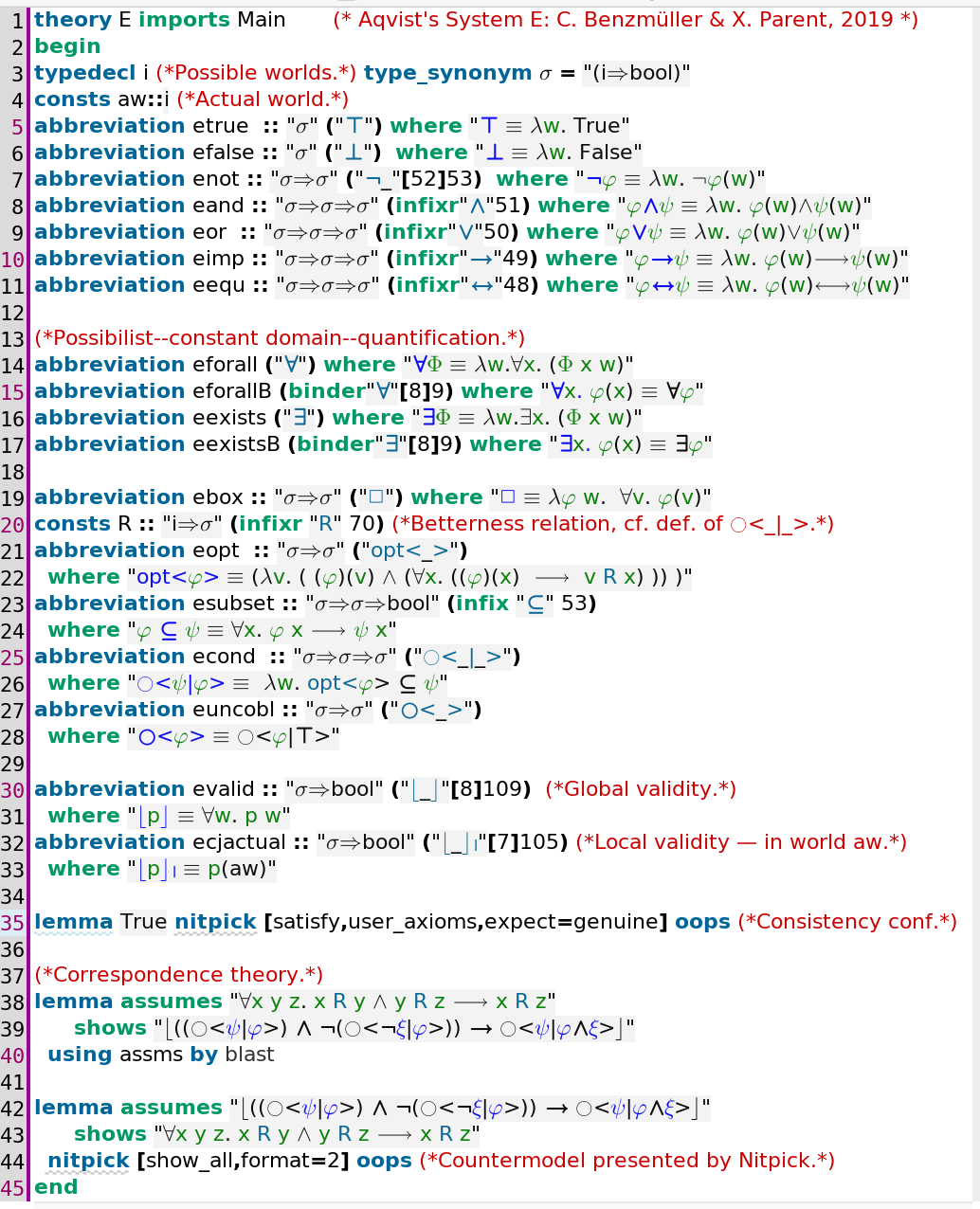

Supplement: Supplementary file 1 [file mmc1.zip › 2020-DataInBrief-Data/E.png]

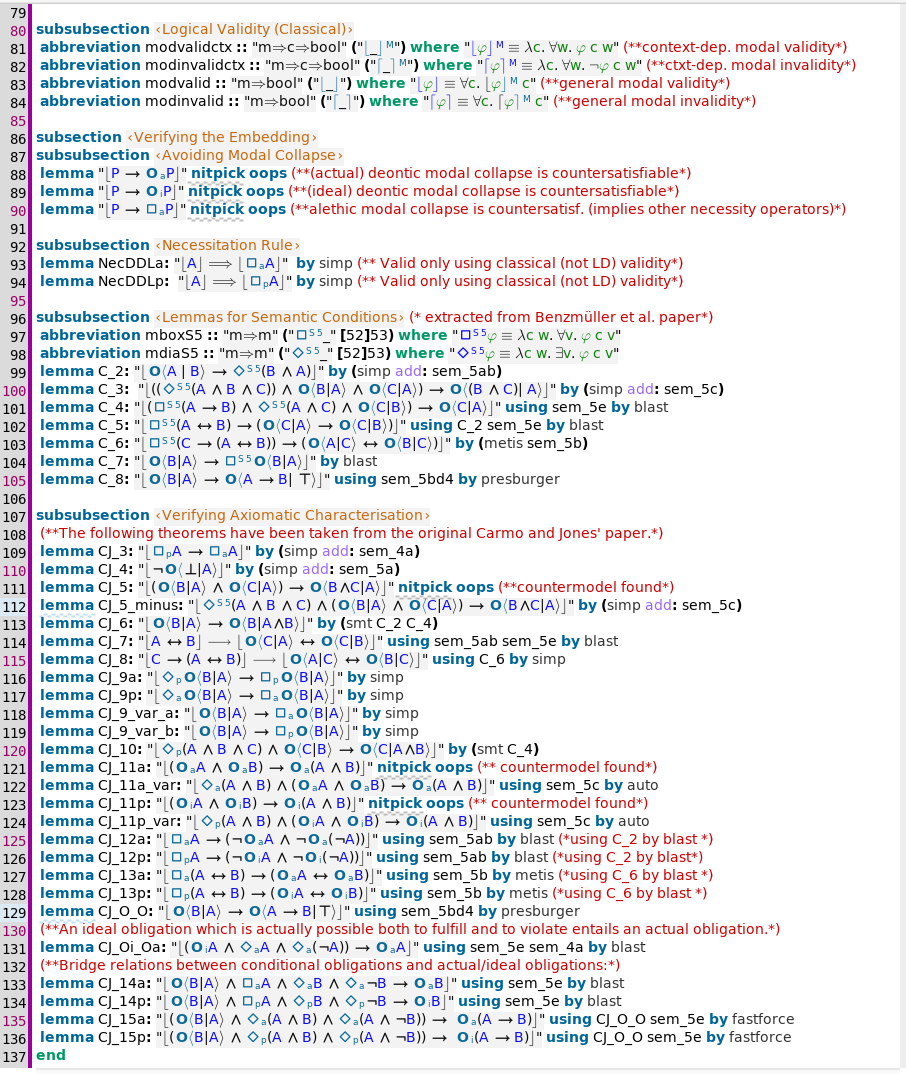

Supplement: Supplementary file 1 [file mmc1.zip › 2020-DataInBrief-Data/CJ_DDLplus2.png]

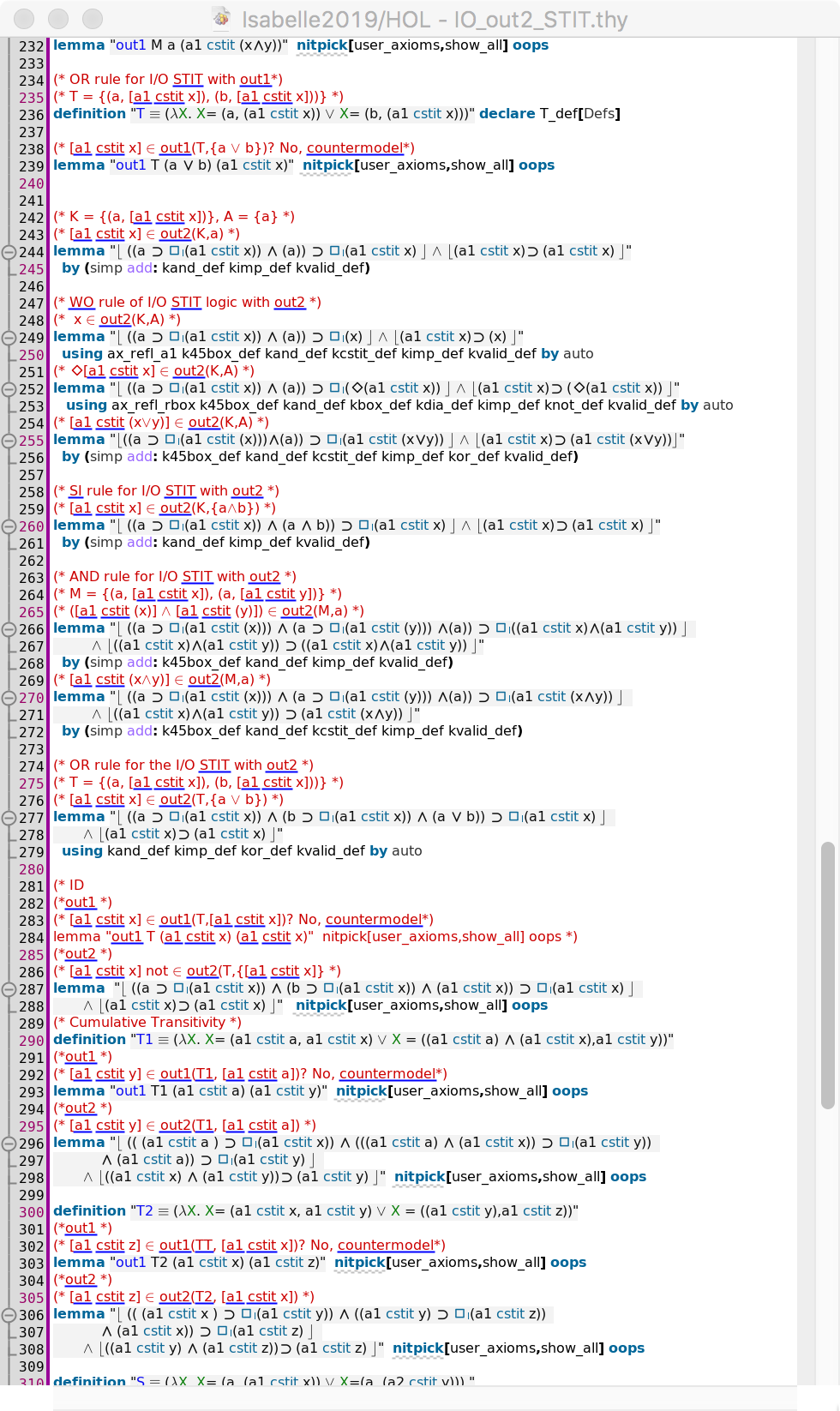

Supplement: Supplementary file 1 [file mmc1.zip › 2020-DataInBrief-Data/IO_out2_STIT4.png]

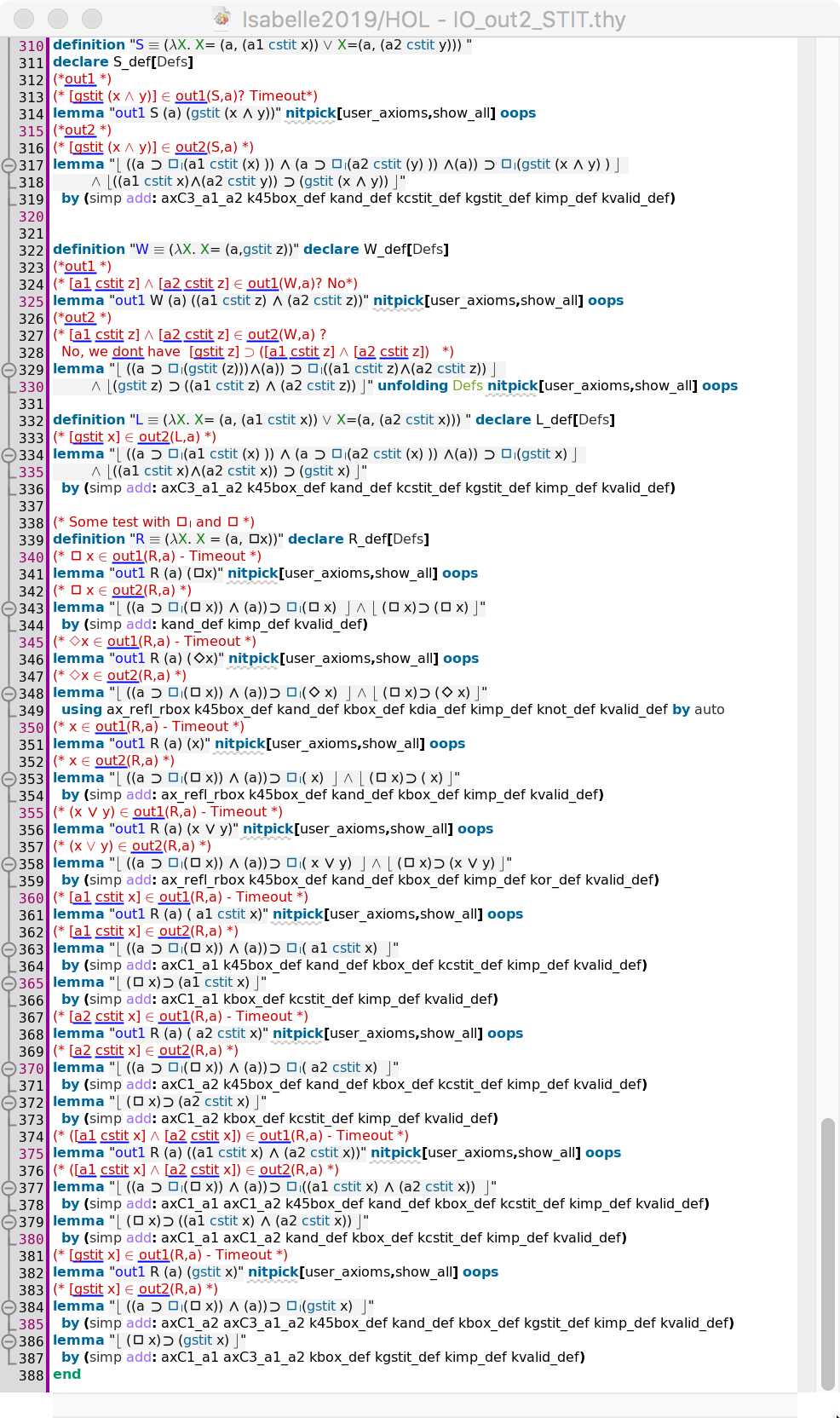

Supplement: Supplementary file 1 [file mmc1.zip › 2020-DataInBrief-Data/IO_out2_STIT5.png]
